# Supplementary material for: Trichoderma reesei complete genome sequence, repeat-induced point mutation, and partitioning of CAZyme gene clusters
Source: Biotechnol Biofuels. 2017 Jul 3;10:170. doi: 10.1186/s13068-017-0825-x (PMC5496416; doi:10.1186/s13068-017-0825-x)
Supplement: Supplementary file 1 — Additional file 1: Table A1. Preliminary assembly results obtained by the Hierarchical Genome Assembly Process (HGAP 3.0). Table A2. Error corrections of the seven PacBio unitigs using the Illimina-MiSeq reads. Table A3. Characteristics and assembly of the seven QM6a chromosomes. Table A4. Mapping of all trimmed paired-end Illumina MiSeq reads to three QM6a genome drafts. Table A5. The MiSeq reads mapped to the QM6a-v2.0 draft genome. Table A6. Sixteen false predicted genes in QM6a-v2.0. Table A7. The QM6a-v2.0 draft genome compared to the complete QM6a genome sequence. Table A8. The HiC draft genome compared to the complete QM6a genome sequence. Table A9. PCR primers. Table A10. The complete QM6a genome sequence versus the RUT-C30-v1.0 draft genome. Table A11. Gene Ontology of some newly-predicted genes. Table A12. Sequencing quality of 18 different fungal genomes. Table A13. RIP indices of various sequences in QM6a and Neurospora crassa. Table A14. Repeat-induced C-to-T mutations observed in the hph alleles in all F1 progeny. Table A15. Saccharomyces cerevisiae Ty elements by chromosome. Table A16. Transposable elements in 12 well-assembled fungal genomes. Table A17. Repetitive sequences in different chromosomal regions. Table A18. Repetitive sequences in seven QM6a chromosomes. Table A19. Partitioning of four gene clusters by the AT-rich islands. The TPM values in glucose (48 hrs), in straw (24 hrs) and in straw (24hrs) then glucose (5 hrs) are shown [24]. [file 13068_2017_825_MOESM1_ESM.pdf]

Additional File 1: Tables A1-A19

Table A1. Preliminary assembly results obtained by the Hierarchical Genome Assembly Process (HGAP 3.0).

Machine code	Name	Reference length	Total read count	Single reads	Reads in pairs	Average coverage
unitig_0	ChI	6835650	1360943	39449	1321494	30.08
unitig_1	ChII	6234469	1243789	36849	1206940	30.22
unitig_2	ChIII	5311312	1057562	31106	1026456	30.11
unitig_3	ChIV	4556699	905754	27232	878522	30.17
unitig_4	ChV	4159848	831575	24745	806830	30.27
unitig_5	ChVI	4000298	881286	27762	853524	33.90
unitig_6	ChVII	3823361	767891	23139	744752	30.61
unitig_7	Mitochondria	69528	346655	13383	333272	905.58
unitig_8	-	1870	0	0	0	0.00

Table A2. Error corrections of the seven PacBio unitigs using the Illimina-MiSeq reads

Query	Subject (raw sequence)	Identity (%)	Alignment length	Mismatch	Gap*	Query start	Query end	Subject start	Subject end	E value	Bit score
ChI	unitig_0_quiver	100	6835815	0	171	1	6835803	1	6835650	0	1.26 E+07
ChII	unitig_1_quiver	100	6234669	3	207	1	6234656	6234469	1	0	1.15 E+07
ChIII	unitig_2_quiver	100	5311458	0	148	1	5311445	1	5311312	0	9.81 E+06
ChIV	unitig_3_quiver	100	4556842	1	145	1	4556834	4556699	1	0	8.41 E+06
ChV	unitig_4_quiver	100	4159971	0	127	1	4159965	1	4159848	0	7.68 E+06
ChVI	unitig_5_quiver	100	4000397	3	105	1	4000387	4000298	1	0	7.39 E+06
ChVII	unitig_6_quiver	100	3823444	0	89	1	3823438	1	3823361	0	7.06 E+06

Table A3. Characteristics and assembly of the seven QM6a chromosomes

Chromosome	Size (bp)	GC (%)[1]	N[2]	Centromere[3]	[4]Telomeric repeats		L telomere + subtelomere[5]	Genes
					L	R	R telomere + subtelomere[5]	
I	6835803	52.01	0	3104000-3303000<br>(199000 bp)	1	13	0-7000<br>6825000-6835803	2149
II	6234656	51.35	0	1940500-2103000<br>(162500 bp)	14	6	0-500<br>6216000-6234656	1977
III	5311445	51.81	0	1745000-1905500<br>(176500 bp)	9	8	1-3500<br>5309000-5311445	1580
IV	4556834	50.96	0	1482500-1659000<br>(176500 bp)	12	7	1-20500<br>4552000-4556834	1443
V	4159965	51.27	0	1164500-1329000<br>(164500 bp)	10	11	1-1000<br>4148500-4159965	1307
VI	4000387	48.09	0	1825500-2034000<br>(208500 bp)	11	9	1-87500<br>3976500-4000387	1211
VII	3823438	50.98	0	1741000-1914000<br>(173000 bp)	7	14	1-23000<br>3816500-3823438	1209
Overall	34922528	51.07	0	1260500 bp	61	71	~258 kb	10876

1. The EMBOSS geecee tool (http://www.bioinformatics.nl/cgi-bin/emboss/geecee) was used to calculate the fractional GC content using a 500 bp sliding window.
2. Number of unresolved bases (N).
3. Centromeres were manually identified as the longest AT-rich block in each chromosome.

4. The telomeric repeats were identified as $(TTAGGG)_n$ at 3′ termini and the complementary sequence $(CCCTAA)_n$ at 5′ termini.
5. Subtelomeres were manually identified as the AT-rich block next to the telomeric repeats.

Table A4. Mapping of all trimmed paired-end Illumina MiSeq reads to three QM6a genome drafts

	v2.0[1] + Mitochrondria[2]		HiC[3]+ Mitochrondria[2]		PacBio raw result	
	count	% bases	count	% bases	count	% bases
References	88	-	12	-	8	-
Mapped reads	7,294,461	98.59%	7,285,215	98.47%	7,395,455	99.96%
Not mapped reads	104,125	1.41%	113,371	1.53%	3,131	0.04%
Reads in pairs	7,080,076	95.69%	6,753,770	91.28%	7,171,790	96.93%
Broken paired reads	214,385	2.90%	531,445	7.18%	223,665	3.02%
Total reads	7,398,586	100.00%	7,398,586	100.00%	7,398,586	100.00%

1. *Trichoderma reesei* QM6a-v2.0 (http://genome.jgi.doe.gov/Trire2/Trire2.home.html)

2. *Trichoderma reesei* complete mitochondrial genome [21].

3.QM6a-HiC assembly [17].

Table A5. The MiSeq reads mapped to the QM6a-v2.0 draft genome

Name	Reference length	Total read count	Single reads	Reads in pairs	Average coverage	Consensus length
Scaffold 1	3756989	778879	23603	755276	31.33222828	3751557
Scaffold 2	2007204	399479	8407	391072	29.84180432	2002082
Scaffold 3	1910749	376320	4074	372246	29.668183	1898164
Scaffold 4	1832615	359186	4880	354306	29.184915	1822980
Scaffold 5	1729360	347709	4673	343036	30.43065643	1726474
Scaffold 6	1455714	290207	2405	287802	30.17369827	1455401
Scaffold 7	1429972	288547	5795	282752	30.43010982	1429872
Scaffold 8	1408331	280709	5431	275278	29.75190775	1408106
Scaffold 9	1219543	311352	2464	308888	37.77733052	1216036
Scaffold 10	1156739	230207	1669	228538	29.75775088	1156419
Scaffold 11	1155933	225443	2655	222788	28.94535064	1155842
Scaffold 12	1022062	215248	9120	206128	31.75998129	1019838
Scaffold 13	891309	172151	1457	170694	28.64029758	890217
Scaffold 14	861070	172440	3598	168842	29.85852602	859039
Scaffold 15	837556	169792	3244	166548	30.78107971	837372
Scaffold 16	824923	165617	2793	162824	30.00676548	824417
Scaffold 17	797352	158547	4487	154060	29.64783559	797185
Scaffold 18	685578	136527	3031	133496	29.73504109	685364
Scaffold 19	663018	131812	1064	130748	30.12435861	662962
Scaffold 20	629213	122814	1428	121386	28.86360898	626721

Scaffold 21	576034	111937	1271	110666	28.86420072	575844
Scaffold 22	541456	108482	1252	107230	30.66683535	541325
Scaffold 23	512080	99631	1475	98156	28.84010311	511978
Scaffold 24	501049	100300	2226	98074	30.33847388	500986
Scaffold 25	439677	88846	1684	87162	30.63345365	439206
Scaffold 26	433400	86099	1159	84940	30.19074296	431280
Scaffold 27	433262	96722	4300	92422	34.2017509	433222
Scaffold 28	407093	84301	3129	81172	31.2420528	405857
Scaffold 29	382182	80172	1936	78236	32.54712415	382148
Scaffold 30	247268	50812	730	50082	31.51634259	247188
Scaffold 31	231272	45921	619	45302	30.46748417	226648
Scaffold 32	230370	47815	1053	46762	31.27029561	230059
Scaffold 33	207997	43645	1105	42540	32.71151507	207899
Scaffold 34	166473	38237	3145	35092	34.48770672	166446
Scaffold 35	152537	35761	2597	33164	36.97032195	150501
Scaffold 36	136855	27507	581	26926	30.39967118	136761
Scaffold 37	132540	27363	519	26844	30.73817715	132508
Scaffold 38	125035	28857	1945	26912	36.85574439	124008
Scaffold 39	105148	22133	1173	20960	31.57468521	105081
Scaffold 40	89857	19116	372	18744	32.37009916	89854
Scaffold 41	80626	16633	705	15928	31.53837472	80496
Scaffold 42	78584	17094	700	16394	33.69913723	78541

Scaffold 43	74996	14949	957	13992	30.46809163	73881
Scaffold 44	66247	16942	2616	14326	39.61809591	66237
Scaffold 45	65952	18681	1997	16684	44.12766861	64692
Scaffold 46	62252	15266	1602	13664	38.90530425	62246
Scaffold 47	50543	14610	1216	13394	50.77391528	49994
Scaffold 48	48367	9919	657	9262	30.80112473	48306
Scaffold 49	46304	11527	1517	10010	39.36132947	45776
Scaffold 50	45663	10008	404	9604	34.86509866	45626
Scaffold 51	43169	32538	9536	23002	130.1951864	43109
Scaffold 52	41083	16933	3379	13554	73.07180586	41083
Scaffold 53	36593	9104	730	8374	40.45333807	36573
Scaffold 54	34758	7137	837	6300	31.74892111	33759
Scaffold 55	33670	23093	10163	12930	118.0482922	32018
Scaffold 56	32194	12046	1670	10376	66.1353979	32200
Scaffold 57	25756	12030	3680	8350	81.41869856	25756
Scaffold 58	21040	9119	2513	6606	74.88003802	21035
Scaffold 59	18517	10742	4434	6308	98.98212453	17650
Scaffold 60	15714	19592	7532	12060	217.7283951	15693
Scaffold 61	15406	14786	6920	7866	160.9715695	15349
Scaffold 62	15337	5129	1283	3846	54.49070874	15190
Scaffold 63	14539	4461	293	4168	44.50794415	13966
Scaffold 64	14482	5823	751	5072	69.3937992	14484

Scaffold 65	12580	7622	2968	4654	104.3219396	12577
#Scaffold 66	11200	2	2	0	0.005892857	36
#Scaffold 67	11021	1	1	0	0.004446058	44
Scaffold 68	10734	4261	1033	3228	70.2005776	10703
Scaffold 69	10696	4029	1463	2566	64.10620793	10684
Scaffold 70	8513	2550	590	1960	50.93363092	7888
#Scaffold 71	6846	0	0	0	0	0
Scaffold 72	6811	3905	1873	2032	98.17868154	6449
Scaffold 73	6421	2875	561	2314	77.07382028	6294
Scaffold 74	5890	2476	528	1948	71.07317487	5890
#Scaffold 75	5683	0	0	0	0	0
#Scaffold 76	5459	0	0	0	0	0
Scaffold 77	5371	2164	964	1200	68.47533048	4577
Scaffold 78	5154	2303	267	2036	76.03259604	5111
Scaffold 79	4691	2419	973	1446	88.64101471	4693
Scaffold 80	4619	2422	822	1600	90.62740853	4609
#Scaffold 81	4614	0	0	0	0	0
#Scaffold 82	4370	0	0	0	0	0
#Scaffold 83	3796	0	0	0	0	0
Scaffold 84	3468	6003	3207	2796	290.5282584	3467
#Scaffold 85	3089	0	0	0	0	0
#Scaffold 86	3000	0	0	0	0	0

Scaffold 87	2158	344	64	280	25.8897127	1738

Ten highlighted rows in yellow are scaffolds with no or very rare Miseq reads. These ten scaffolds (highlighted in yellow background) are not genuine *Trichoderma reesei* sequences.

Table A6. Sixteen false predicted genes in QM6a-v2.0

False gene Id in QM6a-v2.0	Scaffold
34413	76
42942	66
43199	66
43302	67
43392	71
43427	83
71146	66
71154	67
71166	67
71167	67
71177	71
71180	71
71192	81
71196	82
71212	85
112695	71

Table A7. The QM6a-v2.0 draft genome compared to the complete QM6a genome sequence

Query QM6a-v2.0	Query Start	Query End	Subject (Pac-bio)	Subject Start	Subject End	Identity (%)	Alignment length	Mismatch	Gap	E value	Bit Score
Scaffold 01	2981794	3756980	ChIV	2269495	1494308	99.99	775200	31	14	0	1.43E+06
Scaffold 01	1	89682	ChV	1696274	1785953	100	89683	0	4	0	1.66E+05
Scaffold 01	89656	863422	ChV	1785955	2559723	100	773777	0	11	0	1.43E+06
Scaffold 01	863409	2198922	ChV	2559736	3895277	99.99	1335561	1	39	0	2.47E+06
Scaffold 01	2199005	2454056	ChV	3896247	4151299	100	255054	0	3	0	4.71E+05
Scaffold 01	2455057	2462279	ChV	4152681	4159904	97.76	7224	161	1	0	12441
Scaffold 02	1436220	2007204	ChIII	2474752	1903747	99.99	571014	2	16	0	1.05E+06
Scaffold 02	153060	1432008	ChIII	3753988	2475067	99.98	1278976	161	42	0	2.36E+06
Scaffold 02	18	98415	ChIII	3850718	3752318	99.99	98402	1	5	0	1.82E+05
Scaffold 03	1253342	1910705	ChVII	2569158	1911735	99.99	657435	2	17	0	1.21E+06
Scaffold 03	1212271	1240208	ChVII	2602928	2574992	100	27938	0	1	0	51585
Scaffold 03	944321	1212286	ChVII	2871061	2603086	99.99	267980	4	8	0	4.95E+05
Scaffold 03	872295	943320	ChVII	2947081	2876056	100	71027	0	2	0	1.31E+05
Scaffold 03	9	872321	ChVII	3819365	2947079	99.99	872332	29	36	0	1.61E+06
Scaffold 04	1	248983	ChI	3301251	3550226	99.99	248986	1	13	0	4.60E+05
Scaffold 04	250135	579158	ChI	3550676	3879695	99.99	329032	0	14	0	6.08E+05
Scaffold 04	585034	586070	ChI	3880113	3881162	98.38	1050	4	13	0	1838
Scaffold 04	587107	1445500	ChI	3881872	4740265	99.99	858416	51	24	0	1.59E+06

Scaffold 04	1446608	1715045	ChI	4740291	5008732	99.99	268448	4	11	0	4.96E+05
Scaffold 04	1715112	1832600	ChI	5010361	5127850	99.98	117496	15	13	0	2.17E+05
Scaffold 05	1584135	1587517	ChI	5240985	5237599	91.97	3389	264	7	0	4745
Scaffold 05	205745	1583084	ChI	6621901	5244544	100	1377382	0	36	0	2.54E+06
Scaffold 05	15	205616	ChI	6827910	6622305	99.99	205612	13	15	0	3.80E+05
Scaffold 06	395026	1455096	ChIII	4916432	3856362	100	1060093	9	24	0	1.96E+06
Scaffold 06	37	395041	ChIII	5311445	4916445	100	395007	0	6	0	7.29E+05
Scaffold 07	13	1427322	ChI	1429714	2392	99.98	1427344	203	25	0	2.63E+06
Scaffold 08	1053069	1408331	ChII	3080452	2725183	99.99	355275	3	11	0	6.56E+05
Scaffold 08	6	1052998	ChII	4134013	3081015	99.99	1053018	13	38	0	1.94E+06
Scaffold 09	17	115994	ChVI	576856	692848	99.98	115995	0	6	0	2.14E+05
Scaffold 09	119147	1042634	ChVI	692957	1616421	99.99	923507	12	21	0	1.71E+06
Scaffold 09	1033894	1219543	ChVI	1670076	1855727	99.99	185655	4	5	0	3.43E+05
Scaffold 10	1	1156739	ChII	4292787	5449533	100	1156769	2	29	0	2.14E+06
Scaffold 11	1	1155933	ChIII	1652434	496497	100	1155955	7	31	0	2.13E+06
Scaffold 12	163575	1006991	ChI	2282403	1438963	99.99	843456	7	25	0	1.56E+06
Scaffold 12	18	162524	ChI	2447043	2284531	99.99	162514	2	8	0	3.00E+05
Scaffold 13	475	891309	ChIV	444699	1335509	99.98	890869	85	61	0	1.64E+06
Scaffold 14	17	1094	ChII	1240863	1241943	99.72	1081	0	3	0	1977
Scaffold 14	3019	861051	ChII	1243227	2101277	99.99	858084	2	26	0	1.58E+06
Scaffold 15	60	837532	ChVI	3916298	3078804	99.99	837519	3	25	0	1.55E+06
Scaffold 16	1	824923	ChVII	1743563	918618	99.99	824972	1	34	0	1.52E+06

Scaffold 17	139528	797352	ChIV	3729932	3072119	99.99	657835	9	21	0	1.22E+06
Scaffold 17	1	139544	ChIV	3869643	3730103	100	139544	0	2	0	2.58E+05
Scaffold 18	26	685551	ChV	481252	1166792	99.99	685562	14	32	0	1.27E+06
Scaffold 19	1	663001	ChIV	3875645	4538646	100	663015	0	20	0	1.22E+06
Scaffold 20	1	271906	ChIV	2272004	2543904	100	271907	2	6	0	5.02E+05
Scaffold 20	272901	373184	ChIV	2543830	2644124	99.9	100310	61	14	0	1.85E+05
Scaffold 20	373174	495487	ChIV	2644138	2766438	99.98	122316	4	8	0	2.26E+05
Scaffold 20	496665	629213	ChIV	2766550	2899095	99.99	132553	1	7	0	2.45E+05
Scaffold 21	22053	575900	ChI	3083432	2529544	99.99	553901	8	19	0	1.02E+06
Scaffold 21	1	22127	ChI	3105595	3083469	99.99	22128	1	2	0	40845
Scaffold 22	1	541433	ChVI	562824	21385	100	541447	2	13	0	1.00E+06
Scaffold 23	1	512050	ChII	718129	1230222	99.99	512100	4	19	0	9.45E+05
Scaffold 24	1	501044	ChVII	913527	412469	99.99	501065	16	14	0	9.25E+05
Scaffold 25	105	439637	ChII	5923339	5483794	99.97	439562	72	33	0	8.11E+05
Scaffold 26	432691	433400	ChII	2115039	2114332	99.3	710	3	2	0	1282
Scaffold 26	20385	431690	ChII	2526411	2115095	99.99	411323	0	12	0	7.59E+05
Scaffold 26	32	19384	ChII	2548436	2529084	99.96	19355	4	4	0	35695
Scaffold 27	1	433246	ChII	95884	529128	100	433252	4	12	0	8.00E+05
Scaffold 28	33	365854	ChV	1693349	1327548	99.99	365824	19	24	0	6.75E+05
Scaffold 29	1	382182	ChVII	402515	20321	99.99	382200	2	6	0	7.06E+05
Scaffold 30	43	247244	ChV	72510	319712	99.99	247215	3	10	0	4.56E+05
Scaffold 31	166	223996	ChII	6010832	6234656	99.99	223831	7	6	0	4.13E+05

Scaffold 32	17	230085	ChIII	491885	261817	100	230071	6	4	0	4.25E+05
Scaffold 33	1	207997	ChIV	7850	215854	100	208005	2	2	0	3.84E+05
Scaffold 34	1	166446	ChII	2554512	2720961	99.99	166452	3	7	0	3.07E+05
Scaffold 35	17	140563	ChIII	103439	243986	100	140548	1	1	0	2.60E+05
Scaffold 36	780	136855	ChII	533368	669445	99.99	136083	1	8	0	2.51E+05
Scaffold 37	1	132499	ChVI	2306227	2173720	99.99	132508	0	7	0	2.45E+05
Scaffold 38	1	21574	ChIV	251449	273007	99.65	21577	55	16	0	39404
Scaffold 38	23524	125035	ChIV	275089	376603	100	101515	1	3	0	1.87E+05
Scaffold 39	1	105113	ChVI	1968909	1863790	99.98	105121	11	9	0	1.94E+05
Scaffold 40	1	89857	ChIII	1657010	1746866	100	89859	0	3	0	1.66E+05
Scaffold 41	15	80502	ChII	5927577	6008066	99.87	80492	102	6	0	1.48E+05
Scaffold 42	19	78571	ChV	328068	406622	99.98	78561	4	14	0	1.45E+05
Scaffold 43	1875	74206	ChI	2524820	2452489	99.96	72334	24	4	0	1.33E+05
Scaffold 43	50	1644	ChI	2526535	2524939	96.99	1597	46	2	0	2684
Scaffold 44	13	66247	ChVI	3007139	3073375	100	66237	0	2	0	1.22E+05
Scaffold 45	64748	65900	ChIII	1137	1	94.98	1156	36	22	0	1794
Scaffold 45	21	63776	ChIII	65620	1862	100	63759	0	3	0	1.18E+05
Scaffold 46	58	62252	ChV	1	62189	99.98	62198	1	5	0	1.15E+05
Scaffold 47	152	50227	ChVI	3919670	3969742	99.98	50076	8	3	0	92409
Scaffold 48	33	48338	ChI	5186239	5234545	99.99	48307	4	1	0	89177
Scaffold 49	35	45832	ChI	5181011	5135208	99.92	45812	16	19	0	84369
Scaffold 50	30	45639	ChVI	2306547	2352160	99.98	45615	2	5	0	84186

Scaffold 51	49	43169	ChVI	2045780	2088899	100	43121	1	1	0	79617
Scaffold 52	1	41083	ChVII	1796669	1837755	99.99	41087	0	4	0	75848
Scaffold 53	3	36569	ChV	479121	442553	99.92	36570	27	4	0	67357
Scaffold 54	11	33733	ChII	707620	673896	99.99	33726	0	4	0	62255
Scaffold 55	1	20483	ChI	3182462	3202948	99.91	20490	9	9	0	37731
Scaffold 55	22088	33645	ChI	3204646	3216205	99.01	11564	105	10	0	20709
Scaffold 56	1	32194	ChIV	3014570	2982377	100	32194	0	0	0	59452
Scaffold 57	1	25756	ChIII	1805296	1831051	100	25756	0	0	0	47563
Scaffold 58	1	21040	ChII	4209062	4188023	99.98	21042	0	3	0	38832
Scaffold 59	1	17951	ChII	4210705	4228621	99.34	17956	74	39	0	32465
Scaffold 60	1	15688	ChV	1251827	1267517	99.97	15691	1	3	0	28951
Scaffold 61	44	15386	ChV	1251849	1236505	99.97	15346	0	4	0	28313
Scaffold 62	1	15193	ChVI	2941717	2956909	99.94	15195	5	4	0	28007
Scaffold 63	182	14539	ChVI	3997931	3983604	98.34	14378	169	39	0	25163
Scaffold 64	18	14482	ChIV	4556834	4542370	99.99	14466	0	2	0	26701
Scaffold 65	1	12580	ChIV	2981768	2969190	99.97	12581	1	3	0	23207
Scaffold 68	27	10716	ChII	4241937	4231238	99.74	10703	12	8	0	19595
Scaffold 69	16	10696	ChII	91115	80434	99.99	10682	0	1	0	19719
Scaffold 70	470	8459	ChII	5459934	5467924	98.44	8014	78	31	0	14102
Scaffold 72	1	6707	ChIII	67505	74189	97.75	6716	111	30	0	11529
Scaffold 73	163	6407	ChIV	224854	218612	99.57	6250	15	12	0	11382
Scaffold 74	1	5890	ChV	439630	433741	100	5890	0	0	0	10877

Scaffold 77	4238	5371	ChI	3228500	3227364	98.77	1138	9	5	0	2021
Scaffold 77	307	3744	ChI	3231975	3228535	99.85	3441	2	3	0	6324
Scaffold 78	1	5108	ChVI	2939112	2934003	99.88	5111	2	3	0	9402
Scaffold 79	1	4691	ChIII	1859056	1854364	99.96	4693	0	2	0	8654
Scaffold 80	1	4603	ChI	3218928	3223538	99.78	4611	2	7	0	8453
Scaffold 84	1	3468	ChVI	2028172	2031639	100	3468	0	0	0	6405
Scaffold 87	5	2158	ChV	4159965	4157812	98.61	2154	30	0	0	3812

Table A8. The HiC draft genome compared to the complete QM6a genome sequence

Query (HiC)	Query Start	Query End	Subject (Pac-bio)	Subject Start	Subject End	Identity (%)	Alignment length	Mismatch	Gap	E value	Bit Score	Assembly error
HiC10	1	1422573	ChI	7125	1429714	100	1422605	10	18	0	2.63E+06	-
HiC 10	1433628	2277068	ChI	1438963	2282403	99.99	843456	31	24	0	1.56E+06	-
HiC 10	2278119	2440625	ChI	2284531	2447043	99.99	162514	2	8	0	3.00E+05	-
HiC 10	2445779	2513193	ChI	2457404	2524820	100	67417	0	2	0	1.25E+05	-
HiC 10	2515068	3066258	ChI	2532227	3083432	99.99	551218	26	16	0	1.02E+06	-
HiC 10	3066184	3088311	ChI	3083469	3105596	99.99	22129	1	2	0	40847	-
HiC 10	3088307	3108793	ChI	3182458	3202948	99.9	20494	10	9	0	37733	-
HiC 10	3110398	3116725	ChI	3204646	3210978	99.7	6334	12	7	0	11585	-
HiC 10	3116725	3365707	ChI	3301251	3550226	99.99	248986	1	13	0	4.60E+05	-
HiC 10	3366859	3695882	ChI	3550676	3879695	99.99	329032	0	14	0	6.08E+05	-
HiC 10	3701758	3702794	ChI	3880113	3881162	98.38	1050	4	13	0	1838	-
HiC 10	3703831	4562224	ChI	3881872	4740265	99.99	858416	51	24	0	1.59E+06	-
HiC 10	4563332	4831769	ChI	4740291	5008732	99.99	268448	4	11	0	4.96E+05	-
HiC 10	4831836	4948352	ChI	5010361	5126873	99.99	116518	0	6	0	2.15E+05	-
HiC 10	4948824	4994621	ChI	5135208	5181011	99.92	45812	16	19	0	84369	-
HiC 10	4994688	5040681	ChI	5186239	5232232	99.99	45994	3	0	0	84919	-
HiC 10	5048796	5052178	ChI	5237599	5240985	91.97	3389	264	7	0	4745	-
HiC 10	5053229	6430568	ChI	5244544	6621901	100	1377382	0	36	0	2.54E+06	-

HiC 10	6430697	6636298	ChI	6622305	6827910	99.99	205612	13	15	0	3.80E+05	-
HiC 9	5928542	5968595	ChII	55579	15524	100	40056	0	2	0	73957	-
HiC 9	5915688	5919039	ChII	82911	86262	100	3352	0	0	0	6191	Inversion
HiC 9	5919055	5923910	ChII	91115	86260	99.96	4857	0	2	0	8957	-
HiC 9	5484478	5915687	ChII	527092	95884	100	431214	0	8	0	7.96E+05	-
HiC 9	5350265	5483698	ChII	666803	533368	99.99	133441	1	8	0	2.46E+05	-
HiC 9	5315519	5349241	ChII	707620	673896	99.99	33726	0	4	0	62255	-
HiC 9	5275922	5315508	ChII	757713	718129	99.99	39587	1	2	0	73085	-
HiC 9	4803429	5275891	ChII	1230222	757744	99.99	472483	3	16	0	8.72E+05	-
HiC 9	4802335	4803412	ChII	1241943	1240863	99.72	1081	0	3	0	1977	-
HiC 9	3942358	3947436	ChII	2096198	2101277	99.98	5080	0	1	0	9374	Inversion
HiC 9	3947455	4800410	ChII	2096199	1243227	99.99	853006	2	25	0	1.58E+06	-
HiC 9	3941649	3942358	ChII	2115039	2114332	99.3	710	3	2	0	1282	-
HiC 9	3529343	3940648	ChII	2526411	2115095	99.99	411323	0	12	0	7.59E+05	-
HiC 9	3508990	3528342	ChII	2548436	2529084	99.96	19355	4	4	0	35695	-
HiC 9	3344167	3508958	ChII	2719306	2554512	100	164797	1	6	0	3.04E+05	-
HiC 9	2988907	3344169	ChII	3080452	2725183	99.99	355275	3	11	0	6.56E+05	-
HiC 9	1935844	2988836	ChII	4134013	3081015	99.99	1053018	13	38	0	1.94E+06	-
HiC 9	1911172	1917607	ChII	4202628	4209063	100	6436	0	0	0	11886	Inversion
HiC 9	1917606	1935557	ChII	4210704	4228621	99.34	17957	74	39	0	32467	Inversion
HiC 9	1906549	1911172	ChII	4231238	4235862	99.61	4628	11	7	0	8440	Inversion
HiC 9	1900432	1906504	ChII	4235855	4241937	99.82	6083	1	2	0	11164	Inversion

HiC 9	743699	1900437	ChII	5449533	4292787	100	1156769	2	29	0	2.14E+06	-
HiC 9	739167	743703	ChII	5459934	5464455	99.38	4537	13	1	0	8209	Inversion
HiC 9	299366	738699	ChII	5923339	5483994	99.97	439362	72	32	0	8.11E+05	-
HiC 9	223235	299247	ChII	6003591	5927577	99.99	76016	0	4	0	1.40E+05	-
HiC 9	1	223069	ChII	6233895	6010832	99.99	223069	7	5	0	4.12E+05	-
HiC 11	5112367	5121959	ChIII	1862	11454	100	9593	0	0	0	17716	Inversion
HiC 11	5056839	5111002	ChIII	65620	11454	99.99	54167	0	3	0	1.00E+05	-
HiC 11	5121959	5127350	ChIII	67505	72896	100	5392	0	0	0	9958	Inversion
HiC 11	4916256	5056802	ChIII	243986	103439	100	140548	1	1	0	2.60E+05	-
HiC 11	4676319	4904286	ChIII	491885	263918	100	227970	1	4	0	4.21E+05	-
HiC 11	4670462	4676302	ChIII	498691	504531	100	5841	0	0	0	10787	Inversion
HiC 11	3522563	4670462	ChIII	1652436	504532	100	1147922	7	31	0	2.12E+06	-
HiC 11	3435458	3522565	ChIII	1744116	1657009	100	87110	0	3	0	1.61E+05	-
HiC 11	3432708	3435457	ChIII	1744117	1746866	100	2750	0	0	0	5079	Inversion
HiC 2	1	4555	ChIII	1823888	1828443	99.98	4556	0	1	0	8407	Insertion
HiC 2	4545	23144	ChIII	1823894	1805296	99.99	18600	1	1	0	34335	Insertion<br>Inversion
HiC 1	1	4048	ChIII	1858403	1854358	99.95	4048	0	1	0	7463	Insertion
HiC 1	4041	4691	ChIII	1858404	1859056	99.69	653	0	2	0	1194	Insertion
HiC 11	3403179	3418102	ChIII	1903746	1918669	100	14924	0	0	0	27560	Inversion
HiC 11	2847118	3403179	ChIII	2474752	1918670	99.99	556091	2	16	0	1.03E+06	-
HiC 11	1563958	2842906	ChIII	3753988	2475067	99.98	1278976	161	42	0	2.36E+06	-

HiC 11	1455732	1554129	ChIII	3850718	3752318	99.99	98402	1	5	0	1.82E+05	-
HiC 11	1455147	1455716	ChIII	3854999	3854430	99.65	570	2	0	0	1042	-
HiC 11	395026	1455096	ChIII	4916432	3856362	100	1060093	9	24	0	1.96E+06	-
HiC 11	37	395041	ChIII	5311445	4916445	100	395007	0	6	0	7.29E+05	-
HiC 7	1	201094	ChIV	7850	208951	100	201102	2	2	0	3.71E+05	-
HiC 7	201095	206879	ChIV	214735	208951	100	5785	0	0	0	10683	Inversion
HiC 7	206878	228452	ChIV	251448	273007	99.65	21578	55	16	0	39406	-
HiC 7	230402	331913	ChIV	275089	376603	100	101515	1	3	0	1.87E+05	-
HiC 7	331913	376733	ChIV	391732	436553	100	44822	0	1	0	82764	-
HiC 7	377204	1268038	ChIV	444699	1335509	99.98	890869	85	61	0	1.64E+06	-
HiC 7	1268039	1328616	ChIV	1342054	1402637	99.99	60584	0	1	0	1.12E+05	-
HiC 7	1330065	1399458	ChIV	1402890	1472283	100	69394	0	0	0	1.28E+05	-
HiC 7	1399455	1410556	ChIV	1505409	1494308	99.75	11102	28	0	0	20347	Inversion
HiC 7	1410567	2171658	ChIV	1505408	2266498	100	761103	1	13	0	1.41E+06	-
HiC 7	2171659	2443564	ChIV	2272004	2543904	100	271907	2	6	0	5.02E+05	-
HiC 7	2444559	2544842	ChIV	2543830	2644124	99.9	100310	61	14	0	1.85E+05	-
HiC 7	2544832	2667145	ChIV	2644138	2766438	99.98	122316	4	8	0	2.26E+05	-
HiC 7	2668323	2800878	ChIV	2766550	2899101	99.99	132560	2	8	0	2.45E+05	-
HiC 7	2841304	2853883	ChIV	2969190	2981768	99.97	12581	1	3	0	23207	-
HiC 7	2800864	2814136	ChIV	2996155	2982882	99.98	13274	2	1	0	24495	Inversion
HiC 7	2814133	2832556	ChIV	3014570	2996147	100	18424	0	0	0	34023	Inversion
HiC 7	2832557	2841303	ChIV	3072119	3080864	99.99	8747	0	1	0	16146	-

HiC 7	2853883	3502961	ChIV	3080864	3729932	99.99	649089	9	20	0	1.20E+06	-
HiC 7	3502945	3642495	ChIV	3730103	3869650	100	139551	1	2	0	2.58E+05	-
HiC 7	3642475	4305489	ChIV	3875633	4538646	100	663029	0	22	0	1.22E+06	-
HiC 7	4307810	4319988	ChIV	4542370	4554547	99.99	12179	0	1	0	22484	-
HiC 7	4305507	4307792	ChIV	4554548	4556834	99.96	2287	0	1	0	4217	-
HiC contig6	3925902	3987055	ChV	61146	1	99.98	61155	0	3	0	1.13E+05	-
HiC 6	3678658	3925859	ChV	319712	72510	99.99	247215	3	10	0	4.56E+05	-
HiC 6	3612592	3678615	ChV	394093	328068	99.99	66027	0	4	0	1.22E+05	-
HiC 6	3606224	3612592	ChV	394093	400460	99.98	6369	0	1	0	11755	Inversion
HiC 6	3602099	3606231	ChV	435506	439637	99.95	4133	1	1	0	7620	Inversion
HiC 6	3592561	3602099	ChV	442553	452092	99.7	9541	26	3	0	17455	Inversion
HiC 6	3565505	3592536	ChV	479126	452093	99.99	27034	1	2	0	49904	-
HiC 6	2879956	3565481	ChV	1166792	481252	99.99	685562	14	32	0	1.27E+06	-
HiC 6	2870206	2879928	ChV	1236505	1246230	99.97	9726	0	3	0	17941	-
HiC 6	2864566	2870185	ChV	1251849	1246231	99.98	5620	0	1	0	10371	-
HiC 6	2848835	2864522	ChV	1267517	1251827	99.97	15691	1	3	0	28951	-
HiC 6	2486437	2848808	ChV	1693349	1330981	100	362374	2	7	0	6.69E+05	-
HiC 6	2396723	2486405	ChV	1785953	1696273	100	89684	0	4	0	1.66E+05	-
HiC 6	1622983	2396749	ChV	2559723	1785955	100	773777	0	11	0	1.43E+06	-
HiC 6	287483	1622996	ChV	3895277	2559736	99.99	1335561	1	39	0	2.47E+06	-
HiC 6	32349	287400	ChV	4151299	3896247	100	255054	0	3	0	4.71E+05	-

HiC 6	7574	12044	ChV	4159330	4154769	78.36	4682	682	145	0	2728	Inversion
HiC 6	24126	31348	ChV	4159904	4152681	97.76	7224	161	1	0	12441	Inversion
HiC 4	3110893	3652325	ChVI	562824	21385	100	541447	2	13	0	1.00E+06	-
HiC 4	2994899	3110876	ChVI	692848	576856	99.98	115995	0	6	0	2.14E+05	-
HiC 4	1	23597	ChVI	1592825	1616421	99.99	23597	3	0	0	43559	rDNA
HiC 4	2040802	2064398	ChVI	1592825	1616421	99.99	23597	3	0	0	43559	rDNA
HiC 4	2066329	2089925	ChVI	1592825	1616421	99.99	23597	3	0	0	43559	rDNA
HiC 4	2091853	2991746	ChVI	1592828	692957	99.99	899914	9	22	0	1.66E+06	-
HiC 4	1991678	2015274	ChVI	1616421	1592825	99.99	23597	3	0	0	43559	rDNA
HiC 4	2017205	2040801	ChVI	1616421	1592825	99.99	23597	3	0	0	43559	rDNA
HiC 4	14857	25527	ChVI	1670076	1680746	99.98	10671	2	0	0	19695	rDNA
HiC 4	2055658	2066329	ChVI	1670076	1680747	99.98	10672	2	0	0	19697	rDNA
HiC 4	2081185	2091855	ChVI	1670076	1680746	99.98	10671	2	0	0	19695	rDNA
HiC 4	2015274	2025945	ChVI	1680747	1670076	99.98	10672	2	0	0	19697	rDNA
HiC 4	1815444	2000418	ChVI	1855052	1670076	99.99	184980	4	5	0	3.42E+05	-
HiC 4	1710348	1815450	ChVI	1968909	1863802	99.98	105109	10	7	0	1.94E+05	-
HiC 4	1668237	1710299	ChVI	2087841	2045780	100	42063	1	1	0	77663	-
HiC 4	1535719	1668217	ChVI	2306227	2173720	99.99	132508	0	7	0	2.45E+05	-
HiC 4	1492668	1535689	ChVI	2349567	2306547	99.99	43022	2	1	0	79429	-
HiC 4	998818	1492670	ChVI	2892561	2398713	99.99	493855	23	8	0	9.12E+05	-
HiC 3	1	1122	ChVI	2935405	2934284	100	1122	0	0	0	2073	Insertion
HiC 3	1109	4830	ChVI	2939126	2935406	99.87	3722	4	1	0	6844	Insertion

HiC 4	979073	994265	ChVI	2956909	2941717	99.94	15195	5	4	0	28007	-
HiC 4	912671	978916	ChVI	3073386	3007139	99.99	66249	0	4	0	1.22E+05	-
HiC 4	75185	912683	ChVI	3916298	3078802	99.99	837521	27	24	0	1.55E+06	-
HiC 4	25679	75125	ChVI	3919670	3969113	99.99	49447	1	3	0	91286	Inversion
HiC 0	3881	14358	ChVI	3987479	3997931	97.77	10498	169	34	0	18029	Insertion
HiC 0	1	3883	ChVI	3987480	3983603	99.87	3883	0	5	0	7138	Insertion
HiC 5	3642624	3654434	ChVII	20467	32277	100	11811	0	0	0	21811	-
HiC 5	3272399	3642623	ChVII	402515	32278	99.99	370243	2	6	0	6.84E+05	-
HiC 5	2773931	3272398	ChVII	913527	415046	99.99	498487	2	11	0	9.20E+05	-
HiC 5	1951833	2773936	ChVII	1743563	921437	99.99	822153	2	34	0	1.52E+06	-
HiC 5	1910749	1951832	ChVII	1837756	1796669	99.99	41088	0	4	0	75850	-
HiC 5	1253342	1910728	ChVII	2569158	1911735	99.99	657435	25	16	0	1.21E+06	-
HiC 5	1212271	1240208	ChVII	2602928	2574992	100	27938	0	1	0	51585	-
HiC 5	944321	1212286	ChVII	2871061	2603086	99.99	267980	4	8	0	4.95E+05	-
HiC 5	872295	943320	ChVII	2947081	2876056	100	71027	0	2	0	1.31E+05	-
HiC 5	9	872321	ChVII	3819365	2947079	99.99	872332	29	36	0	1.61E+06	-

#The HiC sequences with inversion errors are highlighted in green, whereas those with insertion errors are in orange.

Table A9. PCR primers

Name	Nucleotide sequence (5'→3')	Length	Applications
PA9442	GAGCCCTAACCCTCCGTTAC	2977 bp	After *Xma*I restriction enzyme, the 5' fragment (524 bp) was purified and used as the 25S rDNA probe
PA9443	CGAAGCAGAATTCGGTAAGC		
PA8205	AACAATATGCGTCACCGAGT	539 bp	The DNA probe for *mus53*
PA8206	ACGGAGGCTCAATATCGCA		
PA7959	GATGTAGGAGGGCGTGGATA	494 bp	The DNA probe for all *hgh* cassettes and Sanger's sequencing
PA7960	ACATTGTTGGAGCCGAAATC		
PA8965	TCTCCGCATTCCGATGGAT	3248 bp	PCR amplification of the *hgh* cassettes from all *blr1***Δ**::*hph* alleles
PA8963	GCGATCCGACGCCACATC		
PA9373	CAAGATGCAAGCGGATAACA	4107 bp	PCR amplification of the *hgh* cassettes from all *ku70***Δ**::*hph* alleles
PA9374	GGGTCAATCTCCCTCTGTGA		
PA9395	AGAGACGGGGCAAAGGAG	4063 bp	PCR amplification of the *hgh* cassettes from all *env1***Δ**::*hph* alleles
PA9396	GCATCAGAAGAGACTGGGATT		
PA6157	CTTAGGGTCCAGGAAGTTCT	495 bp	PCR amplification of the *blr1*gene
PA6158	CAACATCTTTGACGAACTCA		
PA6040	CAGGAGACTCCAAGTTTGATC	549 bp	PCR amplification of the *ku70* gene
PA6041	TGCTGCGCTTCTTGAATC		
PA6176	CCCGTAGAATTGTTTGAAAG	1205 bp	PCR amplification of the *env1* gene
PA6177	CCAAGCTCTAAACGTAAGTCA		

Table A10. The complete QM6a genome sequence versus the RUT-C30-v1.0 draft genome

RUT-C30	R-start	R-end	QM6a	Q-Start	Q-end	Identity (%)	Length	Mismatch	Gap	E value	Bit Score
scaffold_1	1	55105	ChV	1698834	1753947	99.98	55114	1	2	0	1.02E+05
scaffold_1	56076	76260	ChV	1755903	1776099	99.94	20197	0	1	0	37220
scaffold_1	76298	76463	ChV	1775881	1776046	90.64	171	6	5	3.0E-53	219
scaffold_1	76463	80706	ChV	1776163	1780406	100	4244	0	0	0	7838
scaffold_1	80757	86278	ChV	1780412	1785933	100	5522	0	0	0	10198
scaffold_1	86312	115685	ChV	1785981	1815354	100	29374	0	0	0	54244
scaffold_1	115853	231340	ChV	1815473	1930966	99.99	115494	4	2	0	2.13E+05
scaffold_1	231798	662947	ChV	1931394	2362552	99.99	431165	3	12	0	7.96E+05
scaffold_1	664233	665902	ChV	2364083	2365752	100	1670	0	0	0	3085
scaffold_1	665899	780773	ChV	2365808	2480687	99.99	114880	11	2	0	2.12E+05
scaffold_1	780761	859776	ChV	2480702	2559718	100	79017	0	1	0	1.46E+05
scaffold_1	859853	1016965	ChV	2559746	2716878	99.97	157142	2	13	0	2.90E+05
scaffold_1	1018564	1178886	ChV	2719408	2879756	99.98	160350	4	5	0	2.96E+05
scaffold_1	1178895	1292480	ChV	2879818	2993389	99.99	113587	0	2	0	2.10E+05
scaffold_1	1292520	1300476	ChV	2993401	3001357	100	7957	0	0	0	14694
scaffold_1	1300922	1407901	ChV	3001840	3108809	99.99	106980	1	1	0	1.98E+05
scaffold_1	1408158	1414351	ChV	3110000	3116193	100	6194	0	0	0	11439
scaffold_1	1414572	1705125	ChV	3116549	3407127	99.99	290580	2	7	0	5.36E+05

scaffold_1	1705341	1968671	ChV	3407428	3670788	99.98	263375	5	7	0	4.86E+05
scaffold_1	1968673	2138285	ChV	3670902	3840554	99.97	169655	4	7	0	3.13E+05
scaffold_1	2138265	2190386	ChV	3840593	3892716	99.99	52124	1	2	0	96237
scaffold_1	2195088	2317751	ChV	3899067	4021751	99.98	122685	2	2	0	2.26E+05
scaffold_1	2317689	2446284	ChV	4021749	4150345	100	128597	0	1	0	2.38E+05
scaffold_1	2446286	2565929	ChII	1940624	1820978	99.99	119648	1	3	0	2.21E+05
scaffold_1	2566007	2575275	ChII	1820956	1811688	100	9269	0	0	0	17117
scaffold_1	2578068	2597581	ChII	1806857	1787344	99.99	19514	1	0	0	36031
scaffold_1	2597626	2779669	ChII	1787302	1605254	99.99	182053	1	8	0	3.36E+05
scaffold_1	2779663	3042312	ChII	1605235	1342582	99.98	262672	6	9	0	4.85E+05
scaffold_1	3042479	3334638	ChII	1342559	1050383	99.99	292185	1	7	0	5.39E+05
scaffold_1	3334636	3597120	ChII	1050292	787788	99.99	262507	2	7	0	4.85E+05
scaffold_2	1	210017	ChIII	1905226	2115232	99.98	210032	3	4	0	3.88E+05
scaffold_2	210058	246378	ChIII	2115283	2151604	100	36322	0	1	0	67067
scaffold_2	246380	374670	ChIII	2151652	2279977	99.95	128329	25	9	0	2.37E+05
scaffold_2	376353	408724	ChIII	2282372	2314744	99.99	32373	2	1	0	59766
scaffold_2	408715	504755	ChIII	2314772	2410818	99.99	96047	1	4	0	1.77E+05
scaffold_2	506926	508298	ChIII	2414204	2415576	100	1373	0	0	0	2536
scaffold_2	508316	567852	ChIII	2415628	2475164	100	59537	0	0	0	1.10E+05
scaffold_2	567899	570334	ChIII	2475168	2477603	100	2436	0	0	0	4499
scaffold_2	570336	736357	ChIII	2477679	2643721	99.98	166046	3	6	0	3.06E+05
scaffold_2	736402	764162	ChIII	2643732	2671491	100	27761	0	1	0	51258

scaffold_2	764217	882041	ChIII	2671470	2789292	99.99	117827	3	6	0	2.18E+05
scaffold_2	882266	912782	ChIII	2789888	2820408	99.99	30521	0	3	0	56336
scaffold_2	914646	1032028	ChIII	2823207	2940596	99.98	117395	3	7	0	2.17E+05
scaffold_2	1032068	1032101	ChI	3578706	3578673	100	34	0	0	1E-06	63.9
scaffold_2	1032103	1111437	ChIII	2940567	3019911	99.99	79345	0	1	0	1.47E+05
scaffold_2	1111439	1208738	ChIII	3020328	3117629	100	97302	1	1	0	1.80E+05
scaffold_2	1208737	1253647	ChIII	3120105	3165023	99.97	44920	2	2	0	82878
scaffold_2	1254102	1256922	ChIII	3165651	3168471	100	2821	0	0	0	5210
scaffold_2	1256983	1259978	ChIII	3168489	3171484	100	2996	0	0	0	5533
scaffold_2	1260036	1448753	ChIII	3171649	3360368	100	188720	2	1	0	3.49E+05
scaffold_2	1448754	1692281	ChI	4499631	4256042	99.97	243591	2	7	0	4.49E+05
scaffold_2	1693957	1904572	ChI	4253685	4043035	99.98	210651	3	5	0	3.89E+05
scaffold_2	1904570	1952107	ChI	4499631	4547167	99.99	47540	2	5	0	87746
scaffold_2	1951983	2139374	ChI	4547081	4734492	99.96	187432	10	10	0	3.46E+05
scaffold_2	2139421	2156750	ChI	4734623	4751949	99.91	17330	12	1	0	31938
scaffold_2	2160517	2207517	ChI	4757101	4804121	99.96	47021	1	3	0	86699
scaffold_2	2207522	2298194	ChI	4804253	4894947	99.96	90698	6	8	0	1.67E+05
scaffold_2	2298166	2352765	ChI	4895103	4949702	100	54600	1	0	0	1.01E+05
scaffold_2	2352529	2391655	ChI	4949544	4988664	99.94	39128	14	4	0	72129
scaffold_2	2391579	2410084	ChI	4988704	5007215	99.96	18512	1	1	0	34143
scaffold_2	2410086	2523293	ChI	5012101	5125305	99.99	113212	3	7	0	2.09E+05
scaffold_3	1	2028	ChVII	1913726	1915753	100	2028	0	0	0	3746

scaffold_3	2525	131693	ChVII	1916335	2045541	99.97	129207	5	1	0	2.38E+05
scaffold_3	136148	412272	ChVII	2053926	2330053	99.99	276131	11	5	0	5.10E+05
scaffold_3	413517	612815	ChVII	2332097	2531405	99.97	199336	3	11	0	3.68E+05
scaffold_3	612824	648085	ChVII	2531484	2566745	100	35262	0	0	0	65117
scaffold_3	657030	683343	ChVII	2576596	2602923	99.94	26328	1	2	0	48525
scaffold_3	683441	948588	ChVII	2603093	2868276	99.98	265190	11	7	0	4.89E+05
scaffold_3	955298	1024356	ChVII	2877983	2947054	99.98	69073	1	3	0	1.28E+05
scaffold_3	1024489	1026477	ChVII	2947280	2949268	99.95	1989	1	0	0	3670
scaffold_3	1026464	1557207	ChVII	2949281	3480033	99.98	530786	54	16	0	9.80E+05
scaffold_3	1557252	1894325	ChVII	3480043	3817123	100	337081	2	3	0	6.22E+05
scaffold_4	1	76011	ChI	6825097	6749072	99.98	76026	0	1	0	1.40E+05
scaffold_4	76051	200211	ChI	6748978	6624817	99.98	124173	8	3	0	2.29E+05
scaffold_4	203907	307319	ChI	6619943	6516523	99.99	103422	1	2	0	1.91E+05
scaffold_4	307316	357057	ChI	6516573	6466833	99.98	49742	8	1	0	91807
scaffold_4	357037	378622	ChI	6466826	6445243	99.99	21586	1	1	0	39846
scaffold_4	378514	468887	ChI	6445392	6355017	99.99	90378	1	6	0	1.67E+05
scaffold_4	470181	545559	ChI	6353014	6277644	99.99	75379	1	2	0	1.39E+05
scaffold_4	545429	620507	ChI	6277797	6202724	99.96	75092	1	8	0	1.39E+05
scaffold_4	622532	703344	ChI	6199903	6119083	99.99	80821	1	5	0	1.49E+05
scaffold_4	707018	737409	ChI	6115310	6084905	99.92	30408	6	5	0	56008
scaffold_4	737391	1036838	ChI	6084322	5784828	99.98	299498	6	10	0	5.53E+05
scaffold_4	1037008	1573239	ChI	5784849	5248569	99.98	536296	15	13	0	9.90E+05

scaffold_5	1	17006	ChIII	5309379	5292374	100	17006	0	0	0	31405
scaffold_5	17876	282639	ChIII	5291312	5026533	99.99	264780	2	5	0	4.89E+05
scaffold_5	282488	317939	ChIII	5026558	4991101	99.96	35458	7	3	0	65402
scaffold_5	317913	392539	ChIII	4991082	4916450	99.99	74633	1	2	0	1.38E+05
scaffold_5	393274	609174	ChIII	4915677	4699743	99.98	215936	8	7	0	3.99E+05
scaffold_5	609185	609414	ChVI	3395813	3395583	99.13	231	1	1	1E-112	414
scaffold_5	609416	853383	ChIII	4699760	4455756	99.98	244011	3	9	0	4.50E+05
scaffold_5	859319	963876	ChIII	4448343	4343786	100	104558	0	0	0	1.93E+05
scaffold_5	963948	1072922	ChIII	4343801	4234826	100	108976	2	1	0	2.01E+05
scaffold_5	1072941	1341181	ChIII	4234838	3966548	99.98	268292	3	7	0	4.95E+05
scaffold_5	1341188	1448619	ChIII	3966564	3859112	99.98	107453	4	5	0	1.98E+05
scaffold_6	1	78102	ChI	6745	84846	99.99	78102	5	0	0	1.44E+05
scaffold_6	82487	130688	ChI	90295	138496	100	48202	0	0	0	89013
scaffold_6	130979	378615	ChI	138856	386485	100	247639	1	2	0	4.57E+05
scaffold_6	378677	402678	ChI	386509	410522	99.89	24014	14	1	0	44215
scaffold_6	402705	644238	ChI	410501	652052	99.99	241552	0	4	0	4.46E+05
scaffold_6	644591	784955	ChI	652872	793255	99.98	140385	2	7	0	2.59E+05
scaffold_6	785011	1029114	ChI	793376	1037522	99.98	244148	4	7	0	4.51E+05
scaffold_6	1029256	1030256	ChI	1037523	1038523	100	1001	0	0	0	1849
scaffold_6	1030258	1043259	ChI	1038569	1051570	99.95	13002	7	0	0	23974
scaffold_6	1043153	1271823	ChI	1051380	1280054	99.98	228694	10	4	0	4.22E+05
scaffold_6	1273675	1278588	ChI	1282725	1287638	100	4914	0	0	0	9075

scaffold_6	1278762	1289232	ChI	1287916	1298386	100	10471	0	0	0	19337
scaffold_6	1291097	1417722	ChI	1301052	1427679	100	126628	2	1	0	2.34E+05
scaffold_7	1	182722	ChII	3508412	3325655	99.98	182758	9	5	0	3.37E+05
scaffold_7	182715	351256	ChII	3325609	3157049	99.98	168566	9	8	0	3.11E+05
scaffold_7	353095	454102	ChII	3151389	3050385	99.99	101011	5	6	0	1.86E+05
scaffold_7	455822	858893	ChII	3047278	2644181	99.99	403104	11	11	0	7.44E+05
scaffold_7	858882	932353	ChII	2643455	2569983	99.99	73476	2	6	0	1.36E+05
scaffold_7	933445	1398895	ChII	2568127	2102615	99.98	465518	25	17	0	8.59E+05
scaffold_8	1	18064	ChIII	1650553	1632481	99.95	18073	0	1	0	33316
scaffold_8	19898	377041	ChIII	1630178	1272970	99.97	357216	23	13	0	6.59E+05
scaffold_8	380356	486831	ChIII	1268807	1162321	99.99	106487	0	1	0	1.97E+05
scaffold_8	486949	669368	ChIII	1162314	979917	99.97	182435	3	7	0	3.37E+05
scaffold_8	669139	858711	ChIII	980212	790599	99.96	189622	19	17	0	3.50E+05
scaffold_8	858576	1151275	ChIII	790795	498070	99.97	292740	30	17	0	5.40E+05
scaffold_9	1	10499	ChI	3302934	3313445	99.87	10512	1	1	0	19324
scaffold_9	10558	58888	ChI	3313579	3361929	99.95	48351	2	2	0	89151
scaffold_9	58932	240579	ChI	3362221	3543908	99.97	181693	2	15	0	3.35E+05
scaffold_9	245439	565925	ChI	3554909	3875451	99.97	320555	16	22	0	5.91E+05
scaffold_9	565723	572213	ChI	3885363	3891853	99.92	6491	5	0	0	11960
scaffold_9	572304	723484	ChI	3891840	4043042	99.97	151203	24	3	0	2.79E+05
scaffold_9	723485	731801	ChVI	3577081	3585397	100	8317	0	0	0	15359
scaffold_9	731803	733053	ChVI	3585518	3586768	100	1251	0	0	0	2311

scaffold_9	733757	800675	ChVI	3587445	3654363	100	66919	0	0	0	1.24E+05
scaffold_9	802355	808092	ChVI	3656936	3662673	100	5738	0	0	0	10597
scaffold_9	810782	950370	ChVI	3665982	3805593	99.98	139614	5	6	0	2.58E+05
scaffold_9	950363	952996	ChII	3521814	3524467	92.92	2655	166	6	0	3843
scaffold_9	950363	1121722	ChVI	3805696	3977084	99.97	171391	15	10	0	3.16E+05
scaffold_10	1	256104	ChVI	3421213	3165092	99.97	256142	13	14	0	4.73E+05
scaffold_10	257064	259591	ChVI	3163550	3161023	100	2528	0	0	0	4669
scaffold_10	262951	291202	ChVI	3156583	3128333	100	28252	0	1	0	52165
scaffold_10	292956	458495	ChVI	3125846	2960266	99.96	165591	7	7	0	3.05E+05
scaffold_10	458497	476449	ChVI	2960230	2942279	99.99	17953	0	1	0	33146
scaffold_10	476443	482039	ChVI	2942260	2936664	100	5597	0	0	0	10336
scaffold_10	483607	553982	ChVI	2933832	2863447	99.98	70386	1	2	0	1.30E+05
scaffold_10	554097	795227	ChVI	2863246	2622096	99.99	241153	2	6	0	4.45E+05
scaffold_10	795262	797348	ChVI	2622076	2619990	99.95	2087	1	0	0	3849
scaffold_10	797365	988014	ChVI	2620030	2429353	99.97	190680	18	6	0	3.52E+05
scaffold_10	988059	1023047	ChVI	2429337	2394349	100	34989	0	0	0	64613
scaffold_11	1	35414	ChI	1441371	1476786	99.81	35425	47	17	0	65029
scaffold_11	35191	36304	ChI	1476645	1477766	98.58	1123	6	4	0	1978
scaffold_11	35812	91730	ChI	1477202	1533089	99.79	55937	49	38	0	1.03E+05
scaffold_11	91751	117371	ChI	1533176	1558798	99.99	25623	1	2	0	47301
scaffold_11	117396	544192	ChI	1558769	1985598	99.97	426868	38	23	0	7.87E+05
scaffold_11	544194	711360	ChI	1985469	2152637	100	167170	3	3	0	3.09E+05

scaffold_11	711567	834344	ChI	2152744	2275518	99.96	122794	9	4	0	2.27E+05
scaffold_11	834430	839430	ChI	2275497	2280496	99.98	5001	0	1	0	9228
scaffold_11	841419	1000085	ChI	2286157	2444824	100	158669	1	3	0	2.93E+05
scaffold_12	1	113790	ChIV	3223444	3337221	99.97	113800	7	5	0	2.10E+05
scaffold_12	113792	211547	ChIV	3337255	3435047	99.96	97794	5	7	0	1.80E+05
scaffold_12	211678	213346	ChIV	3435090	3436758	100	1669	0	0	0	3083
scaffold_12	214116	275380	ChIV	3438928	3500201	99.98	61275	1	4	0	1.13E+05
scaffold_12	275371	316079	ChIV	3500402	3541110	99.99	40709	3	0	0	75161
scaffold_12	316081	490259	ChIV	3541176	3715382	99.97	174213	5	15	0	3.21E+05
scaffold_12	490261	490387	ChV	906766	906892	98.43	127	2	0	2E-55	224
scaffold_12	490353	737073	ChIV	3715415	3962173	99.98	246763	9	11	0	4.55E+05
scaffold_12	737119	882497	ChIV	3962191	4107598	99.98	145408	1	3	0	2.68E+05
scaffold_13	1	33530	ChII	4989900	4956364	99.97	33537	2	2	0	61878
scaffold_13	33515	172290	ChII	4956355	4817546	99.97	138813	6	5	0	2.56E+05
scaffold_13	172197	185586	ChII	4817710	4804322	99.99	13390	1	1	0	24714
scaffold_13	185864	451261	ChII	4803992	4538554	99.98	265440	3	7	0	4.90E+05
scaffold_13	451334	471361	ChII	4538507	4518480	100	20028	1	0	0	36982
scaffold_13	471355	609778	ChII	4518462	4380011	99.97	138454	3	5	0	2.56E+05
scaffold_13	609823	617432	ChII	4380030	4372421	100	7610	0	0	0	14054
scaffold_13	621602	670727	ChII	4366677	4317543	99.98	49136	1	3	0	90660
scaffold_13	670783	733467	ChII	4317533	4254850	100	62686	0	2	0	1.16E+05
scaffold_13	733469	772749	ChII	4254811	4215533	99.99	39282	0	2	0	72515

scaffold_13	772830	852609	ChII	4215509	4135730	100	79780	0	0	0	1.47E+05
scaffold_14	1	2973	ChVII	1741071	1738099	100	2973	0	0	0	5491
scaffold_14	3178	155825	ChVII	1737805	1585167	99.97	152653	30	5	0	2.82E+05
scaffold_14	156221	169120	ChVII	1576389	1563487	99.98	12903	0	3	0	23808
scaffold_14	170120	206024	ChVII	1562341	1526434	99.98	35910	0	4	0	66268
scaffold_14	199972	201452	ChVI	354376	355856	72.02	1519	349	62	8E-103	381
scaffold_14	206062	230886	ChVII	1526434	1501598	99.94	24838	1	3	0	45774
scaffold_14	231566	465628	ChVII	1500917	1266870	99.97	234086	11	7	0	4.32E+05
scaffold_14	465660	468847	ChVII	1266837	1263650	100	3188	0	0	0	5888
scaffold_14	469017	490702	ChVII	1263387	1241700	99.87	21690	23	5	0	39888
scaffold_14	490704	565163	ChVII	1241908	1167440	99.99	74469	1	1	0	1.38E+05
scaffold_14	565165	608896	ChVII	1167410	1123680	100	43732	0	1	0	80751
scaffold_14	608898	724533	ChVII	1123174	1007512	99.97	115665	5	9	0	2.13E+05
scaffold_14	724504	791338	ChVII	1007563	940730	100	66835	1	1	0	1.23E+05
scaffold_14	791310	791808	ChVII	940712	940214	98.6	500	5	2	0	883
scaffold_14	793986	808780	ChVII	936076	921282	99.99	14795	2	0	0	27311
scaffold_15	1	12807	ChIV	1482752	1469947	99.97	12807	3	1	0	23629
scaffold_15	12800	64430	ChIV	1469841	1418208	99.99	51634	0	2	0	95330
scaffold_15	66017	125191	ChIV	1415792	1356642	99.93	59180	5	4	0	1.09E+05
scaffold_15	125519	208068	ChIV	1356309	1273760	100	82550	0	0	0	1.52E+05
scaffold_15	209693	362064	ChIV	1271264	1118823	99.93	152443	33	19	0	2.81E+05
scaffold_15	362042	362813	ChIV	1118786	1118016	99.87	772	0	1	0	1419

scaffold_15	363112	399482	ChIV	1117669	1081312	99.94	36375	0	2	0	67036
scaffold_15	399515	419232	ChIV	1081335	1061619	99.99	19718	0	1	0	36405
scaffold_15	419344	506591	ChIV	1061214	973986	99.96	87257	2	5	0	1.61E+05
scaffold_15	508007	586444	ChIV	971366	892917	99.98	78451	0	4	0	1.45E+05
scaffold_15	586549	652317	ChIV	892897	827145	99.97	65771	3	3	0	1.21E+05
scaffold_15	652695	790617	ChIV	826714	688779	99.99	137936	1	3	0	2.55E+05
scaffold_16	1	281838	ChIV	3060147	2778285	99.98	281880	8	7	0	5.20E+05
scaffold_16	281798	577251	ChIV	2778399	2482933	99.99	295470	21	10	0	5.45E+05
scaffold_16	575408	582570	ChIV	1636615	1643790	80.06	7196	13	44	0	5291
scaffold_16	577239	633395	ChIV	2482876	2426719	99.99	56158	2	1	0	1.04E+05
scaffold_16	633494	701249	ChIV	2426402	2358652	99.94	67759	29	6	0	1.25E+05
scaffold_16	701251	771230	ChIV	2358865	2288884	99.99	69985	0	4	0	1.29E+05
scaffold_17	1	65236	ChVI	919186	853945	99.99	65243	0	3	0	1.20E+05
scaffold_17	65438	364445	ChVI	853803	554802	100	299010	3	7	0	5.52E+05
scaffold_17	364586	568555	ChVI	554576	350589	99.97	203998	27	10	0	3.76E+05
scaffold_17	569863	747696	ChVI	348489	170634	99.98	177857	3	2	0	3.28E+05
scaffold_17	747697	747802	ChVI	85584	85479	100	106	0	0	3E-47	196
scaffold_18	1	65640	ChV	1164688	1099036	99.98	65653	1	1	0	1.21E+05
scaffold_18	65695	263429	ChV	1099015	901275	99.98	197761	3	7	0	3.65E+05
scaffold_18	269116	323246	ChV	896393	842266	99.96	54140	1	3	0	99836
scaffold_18	323209	332385	ChV	842234	833058	99.99	9177	1	0	0	16942
scaffold_18	332436	375226	ChV	833053	790263	100	42791	1	0	0	79017

scaffold_18	375365	377954	ChV	790250	787661	100	2590	0	0	0	4783
scaffold_18	379657	389067	ChV	785053	775643	99.99	9411	1	0	0	17374
scaffold_18	389083	393125	ChV	775518	771475	99.98	4044	0	1	0	7461
scaffold_18	393127	394032	ChII	786869	787775	99.89	907	0	1	0	1668
scaffold_18	394021	445511	ChV	771472	719966	99.96	51508	1	4	0	94998
scaffold_18	445803	682269	ChV	719464	482968	99.98	236500	6	7	0	4.37E+05
scaffold_19	1	387877	ChIV	678731	290834	99.98	387924	3	15	0	7.16E+05
scaffold_19	387983	574769	ChIV	290806	104019	100	186790	1	3	0	3.45E+05
scaffold_19	574771	658193	ChIV	103605	20160	99.97	83446	1	1	0	1.54E+05
scaffold_19	658175	660971	ChVI	58619	55826	99.79	2798	1	2	0	5129
scaffold_20	1	55793	ChIV	1658844	1714650	99.97	55807	1	1	0	1.03E+05
scaffold_20	55887	61263	ChIV	1714747	1720123	99.96	5377	2	0	0	9919
scaffold_20	61475	62516	ChII	2569183	2570224	91.55	1042	88	0	0	1437
scaffold_20	61475	130764	ChIV	1720972	1790274	99.98	69304	1	3	0	1.28E+05
scaffold_20	130897	139072	ChIV	1790429	1798604	99.9	8180	0	3	0	15055
scaffold_20	139015	140976	ChIV	1798493	1800454	100	1962	0	0	0	3624
scaffold_20	141150	352629	ChIV	1800441	2011968	99.97	211537	6	9	0	3.90E+05
scaffold_20	353296	615917	ChIV	2012715	2275333	100	262624	5	5	0	4.85E+05
scaffold_20	615919	622596	ChIV	2275390	2282067	100	6678	0	0	0	12333
scaffold_21	1	16587	ChI	3104263	3087676	99.99	16588	1	1	0	30622
scaffold_21	16583	20663	ChI	3087596	3083516	100	4081	0	0	0	7537
scaffold_21	20712	180327	ChI	3083415	2923799	100	159617	2	1	0	2.95E+05

scaffold_21	180418	564944	ChI	2923717	2539083	99.96	384641	15	16	0	7.10E+05
scaffold_21	565250	571177	ChI	2537760	2531833	100	5928	0	0	0	10948
scaffold_22	1	166071	ChII	5007022	5173109	99.98	166089	19	4	0	3.07E+05
scaffold_22	166114	200921	ChII	5173143	5207959	99.96	34818	2	4	0	64218
scaffold_22	201061	224662	ChII	5207958	5231558	100	23602	0	1	0	43578
scaffold_22	224615	307108	ChII	5231432	5313922	99.99	82494	4	3	0	1.52E+05
scaffold_22	307134	335236	ChII	5313903	5342005	100	28103	1	0	0	51891
scaffold_22	335272	354651	ChII	5342030	5361412	99.98	19383	1	3	0	35770
scaffold_22	354636	507801	ChII	5361470	5514638	100	153170	2	4	0	2.83E+05
scaffold_23	1	41598	ChVII	911444	869849	99.93	41611	2	4	0	76650
scaffold_23	41645	496651	ChVII	869872	414821	99.99	455054	7	9	0	8.40E+05
scaffold_24	1	491864	ChVI	1602038	1110160	99.99	491896	6	11	0	9.08E+05
scaffold_25	1	1178	ChIV	4168141	4166964	100	1178	0	0	0	2176
scaffold_25	1914	3265	ChII	4117781	4116430	100	1352	0	0	0	2497
scaffold_25	3319	61175	ChII	4116408	4058552	100	57857	1	0	0	1.07E+05
scaffold_25	61256	389513	ChII	4058466	3730176	99.99	328295	5	12	0	6.06E+05
scaffold_25	389466	409395	ChII	3730308	3710381	99.95	19930	8	1	0	36747
scaffold_25	414905	429282	ChII	3702615	3688238	100	14378	0	0	0	26552
scaffold_26	1	76892	ChII	5707150	5784055	99.98	76906	0	1	0	1.42E+05
scaffold_26	77018	89127	ChII	5784075	5796190	99.95	12116	0	1	0	22336
scaffold_26	96617	193209	ChII	5808082	5904677	99.99	96596	5	2	0	1.78E+05
scaffold_26	193207	206414	ChII	5904637	5917845	99.99	13209	0	1	0	24386

scaffold_26	206428	228830	ChII	5917817	5940219	100	22403	1	0	0	41366
scaffold_26	230356	424381	ChII	5942474	6136512	99.98	194040	23	4	0	3.58E+05
scaffold_27	3	150046	ChII	741831	591774	99.99	150059	3	2	0	2.77E+05
scaffold_27	151452	301075	ChII	587497	437875	100	149625	3	3	0	2.76E+05
scaffold_27	302325	368978	ChII	436424	369771	100	66654	0	0	0	1.23E+05
scaffold_27	368952	423763	ChII	369365	314536	99.95	54833	4	7	0	1.01E+05
scaffold_28	1	57471	ChV	761	58237	99.99	57477	1	1	0	1.06E+05
scaffold_28	57473	78753	ChV	1328621	1349900	100	21281	0	1	0	39292
scaffold_28	79106	418400	ChV	1351470	1690798	99.96	339371	16	13	0	6.26E+05
scaffold_29	1	677	ChI	5187197	5187873	100	677	0	0	0	1251
scaffold_29	677	96559	ChIII	3360368	3456236	99.92	95896	33	10	0	1.77E+05
scaffold_29	96281	167567	ChIII	3455874	3527169	99.92	71307	23	10	0	1.31E+05
scaffold_29	167430	389225	ChIII	3526986	3748779	99.99	221802	5	8	0	4.10E+05
scaffold_30	1	155110	ChVII	22764	177904	99.98	155142	4	4	0	2.86E+05
scaffold_30	156170	219732	ChVII	179724	243286	100	63564	0	2	0	1.17E+05
scaffold_30	229520	374706	ChVII	254590	399776	100	145188	1	2	0	2.68E+05
scaffold_31	1	243404	ChV	317356	73975	99.98	243409	18	4	0	4.49E+05
scaffold_32	1	225946	ChIII	263756	489681	99.99	225949	2	3	0	4.17E+05
scaffold_33	1	25980	ChII	46	26024	100	25980	0	1	0	47969
scaffold_33	26912	219820	ChII	28181	221097	100	192917	1	2	0	3.56E+05
scaffold_34	1	91428	ChIV	4542920	4451474	99.98	91447	1	2	0	1.69E+05
scaffold_34	93349	197531	ChIV	4448365	4344140	99.96	104226	1	2	0	1.92E+05

scaffold_35	1	1361	ChVI	3440953	3442327	98.98	1375	0	1	0	2449
scaffold_35	2834	3236	ChVI	350639	351041	98.51	403	6	0	0	712
scaffold_35	2867	16098	ChVI	3444777	3458008	99.89	13232	15	0	0	24352
scaffold_35	17803	134867	ChVI	3459955	3577042	99.98	117089	2	4	0	2.16E+05
scaffold_35	134868	134991	ChI	5187873	5187996	100	124	0	0	7.0E-58	230
scaffold_35	135845	141342	ChI	5189314	5194811	100	5498	0	0	0	10154
scaffold_35	142686	159103	ChI	5196451	5212868	99.98	16418	3	0	0	30304
scaffold_35	159058	178894	ChI	5212739	5232575	99.97	19837	5	0	0	36607
scaffold_36	1	89545	ChVI	2146824	2236384	99.97	89564	5	5	0	1.65E+05
scaffold_36	89565	165123	ChVI	2236531	2312086	99.96	75566	11	4	0	1.39E+05
scaffold_36	166591	171881	ChVI	2314508	2319798	100	5291	0	0	0	9771
scaffold_37	1	2800	ChII	3515167	3517966	100	2800	0	0	0	5171
scaffold_37	3103	6368	ChII	3518080	3521345	100	3266	0	0	0	6032
scaffold_37	7508	161369	ChII	3523781	3677633	99.99	153866	3	3	0	2.84E+05
scaffold_38	1	140130	ChIV	3215166	3075016	99.97	140162	3	5	0	2.59E+05
scaffold_39	1	134967	ChIII	240700	105737	100	134967	3	1	0	2.49E+05
scaffold_39	135759	136837	ChVII	2331733	2330655	99.54	1079	5	0	0	1965
scaffold_40	1	19970	ChII	5567455	5587423	99.99	19970	0	1	0	36871
scaffold_40	20730	132228	ChII	5588821	5700348	99.97	111529	2	6	0	2.06E+05
scaffold_41	1	129422	ChVI	1696184	1825620	99.99	129437	2	2	0	2.39E+05
scaffold_42	1	29352	ChVI	2033891	2063267	99.91	29377	1	1	0	54083
scaffold_42	29354	100072	ChVI	2063320	2134038	100	70719	3	0	0	1.31E+05

scaffold_43	1	97962	ChIV	4279286	4181325	100	97962	0	0	0	1.81E+05
scaffold_44	1	93284	ChIII	3754485	3847766	99.99	93284	8	2	0	1.72E+05
scaffold_45	1	4061	ChIII	1657816	1661876	100	4061	0	0	0	7500
scaffold_45	4117	87508	ChIII	1661886	1745280	100	83395	0	2	0	1.54E+05
scaffold_46	1	13796	ChII	230762	244554	99.97	13796	1	2	0	25453
scaffold_46	13774	73864	ChII	244561	304650	100	60091	0	1	0	1.11E+05
scaffold_47	1	1095	ChV	400490	399396	98.08	1095	21	0	0	1906
scaffold_47	1409	41406	ChV	399317	359321	99.94	40005	11	8	0	73719
scaffold_47	41121	46262	ChV	359415	354270	99.36	5156	9	11	0	9319
scaffold_47	48080	69695	ChV	351648	330031	99.99	21618	0	1	0	39909
scaffold_48	1	69203	ChI	2454978	2524184	99.99	69207	3	1	0	1.28E+05
scaffold_49	1	62064	ChVI	991635	929573	100	62064	0	1	0	1.15E+05
scaffold_50	1	59456	ChIII	2792	62248	100	59457	0	1	0	1.10E+05
scaffold_51	1	48764	ChIV	4116910	4165673	100	48764	1	0	0	90045
scaffold_52	1	40667	ChVI	1650609	1691275	100	40667	0	0	0	75098
scaffold_53	1	8251	ChI	5137666	5145916	100	8251	0	0	0	15237
scaffold_53	10161	39108	ChI	5148355	5177305	99.98	28951	3	1	0	53428
scaffold_54	1	34869	ChII	6216469	6181601	100	34869	1	0	0	64386
scaffold_55	1	30664	ChV	477242	446579	100	30664	0	0	0	56626
scaffold_56	1	29153	ChII	5559261	5530107	99.99	29155	0	1	0	53827
scaffold_57	1	14827	ChIV	1573568	1558742	100	14827	0	0	0	27381
scaffold_57	18483	18514	ChIV	4489563	4489532	100	32	0	0	1.0E-07	60.2

scaffold_58	1	1948	ChVII	1	1948	100	1948	0	0	0	3598
scaffold_58	2491	13676	ChVII	2586	13746	93.21	11241	628	40	0	16406
scaffold_59	1	12354	ChVI	32351	44704	100	12354	0	0	0	22814
scaffold_60	1	10705	ChVI	1045581	1056285	100	10705	0	0	0	19769
scaffold_61	1	4772	ChIV	9022	4251	100	4772	0	0	0	8813
scaffold_61	5424	9278	ChIV	3896	42	100	3855	0	0	0	7119
scaffold_62	1	9246	ChVI	6493	15738	99.99	9246	1	0	0	17069
scaffold_63	1	9044	ChIV	4302410	4293367	100	9044	0	0	0	16702
scaffold_64	1	1197	ChV	1240022	1241218	100	1197	0	0	0	2211
scaffold_64	3788	8018	ChV	1244202	1248432	100	4231	0	0	0	7814
scaffold_65	1	4250	ChI	3189637	3193886	99.91	4250	4	0	0	7827
scaffold_65	4859	7194	ChI	3194196	3196531	99.96	2336	1	0	0	4309
scaffold_66	1	1957	ChV	1263931	1261978	99.59	1957	5	3	0	3568
scaffold_66	1929	7115	ChV	1261886	1256700	100	5187	0	0	0	9579
scaffold_67	1	7103	ChVI	51882	44780	100	7103	0	0	0	13117
scaffold_68	1	6317	ChII	6145812	6152128	100	6317	0	0	0	11666
scaffold_69	1	5706	ChIII	1826533	1820828	100	5706	0	0	0	10538
scaffold_70	1	1334	ChV	1243592	1242259	100	1334	0	0	0	2464
scaffold_70	941	1334	ChV	1224975	1224580	92.68	396	27	1	1.0E-161	569
scaffold_70	4289	5243	ChV	1238657	1237703	99.58	956	2	2	0	1742
scaffold_71	1	4847	ChVI	73986	78832	100	4847	0	0	0	8951
scaffold_72	1	1267	ChVI	1936145	1937411	100	1267	0	0	0	2340

scaffold_72	2577	4426	ChVI	1938626	1940475	100	1850	0	0	0	3417
scaffold_73	1	4134	ChVI	1926403	1922271	99.98	4134	0	1	0	7627
scaffold_74	1	3905	ChVI	62338	66242	100	3905	0	0	0	7212
scaffold_75	1	3608	ChIV	4288810	4285203	99.97	3608	1	0	0	6660
scaffold_76	1	3432	ChIII	72302	68871	100	3432	0	0	0	6338
scaffold_77	1	3397	ChV	355823	359219	100	3397	0	0	0	6274
scaffold_78	1	3113	ChVI	69373	66261	100	3113	0	0	0	5749
scaffold_79	1	3079	ChVI	58726	61804	100	3079	0	0	0	5686
scaffold_80	1	3005	ChVI	1943380	1946384	100	3005	0	0	0	5550
scaffold_81	1	2769	ChII	1997868	2000636	100	2769	0	0	0	5114
scaffold_82	1	2758	ChII	770092	772849	100	2758	0	0	0	5094
scaffold_83	1	2705	ChII	6230125	6232829	100	2705	0	0	0	4996
scaffold_84	1	2680	ChVI	1949664	1952343	100	2680	0	0	0	4950
scaffold_85	1	2617	ChVI	1064630	1067246	100	2617	0	0	0	4833
scaffold_86	1	2614	ChVII	1826527	1829140	100	2614	0	0	0	4828
scaffold_87	1	2571	ChI	4751948	4754518	100	2571	0	0	0	4748
scaffold_88	1	2511	ChVII	1831990	1834500	100	2511	0	0	0	4638
scaffold_89	1	2447	ChVI	1921910	1919464	100	2447	0	0	0	4519
scaffold_90	1	2407	ChVI	54194	51788	100	2407	0	0	0	4446
scaffold_92	1	2222	ChVII	1829716	1831937	100	2222	0	0	0	4104
scaffold_93	1	2179	ChII	2031709	2033887	100	2179	0	0	0	4024
scaffold_94	1	2113	ChI	3546631	3544519	100	2113	0	0	0	3903

scaffold_95	1	2058	ChVII	1808487	1810544	100	2058	0	0	0	3801
scaffold_96	1	2013	ChI	3180538	3178526	99.95	2013	1	0	0	3712
scaffold_97	1	1953	ChIII	1819615	1817663	100	1953	0	0	0	3607
scaffold_98	1	1907	ChI	3187819	3185913	99.58	1907	8	0	0	3478
scaffold_99	1	1897	ChIV	4174695	4172799	99.58	1897	8	0	0	3459
scaffold_100	1	1871	ChII	1808731	1806861	100	1871	0	0	0	3456
scaffold_101	18	1829	ChIV	4556834	4555023	100	1812	0	0	0	3347
scaffold_102	1	1835	ChVI	1928490	1930324	100	1835	0	0	0	3389
scaffold_104	1	1135	ChI	6831440	6830330	84.02	1158	115	28	0	1050
scaffold_104	1138	1664	ChVI	49223	49745	88.43	527	57	4	0	632
scaffold_105	1	1634	ChVII	1823580	1821947	100	1634	0	0	0	3018
scaffold_106	1	1590	ChVI	4528	2939	100	1590	0	0	0	2937
scaffold_107	1	1565	ChVI	3665906	3664342	100	1565	0	0	0	2891
scaffold_108	1	1561	ChVII	244674	246234	100	1561	0	0	0	2883
scaffold_109	1	1556	ChII	5801873	5800318	100	1556	0	0	0	2874
scaffold_110	1	1542	ChV	4159929	4158388	100	1542	0	0	0	2848
scaffold_112	1	1520	ChII	6234450	6232931	100	1520	0	0	0	2808
scaffold_113	1	1503	ChIV	4110849	4109347	100	1503	0	0	0	2776
scaffold_114	1	1448	ChV	414945	416392	100	1448	0	0	0	2675
scaffold_115	1	1439	ChII	1997089	1995651	100	1439	0	0	0	2658
scaffold_116	1	1430	ChI	6354444	6353010	99.58	1435	1	1	0	2612
scaffold_117	7	1381	ChII	6227239	6228613	98.47	1375	21	0	0	2423

scaffold_118	1	1373	ChVI	3160669	3159297	100	1373	0	0	0	2536
scaffold_119	1	1364	ChVI	1013526	1014889	100	1364	0	0	0	2519
scaffold_120	1	1361	ChI	5237745	5239105	99.85	1361	2	0	0	2503
scaffold_121	1	1354	ChVI	5008	6361	100	1354	0	0	0	2501
scaffold_122	1	1305	ChVI	3998487	3999791	100	1305	0	0	0	2410
scaffold_123	1	1302	ChI	3551990	3550689	100	1302	0	0	0	2405
scaffold_124	1	1295	ChVI	1010667	1011961	96.37	1295	47	0	0	2132
scaffold_125	1	1294	ChII	2003067	2001774	100	1294	0	0	0	2390
scaffold_126	1	1294	ChII	2028132	2029422	98.38	1294	18	3	0	2270
scaffold_127	1	1274	ChVII	1820648	1821921	100	1274	0	0	0	2353
scaffold_128	1	1273	ChVII	2048122	2046850	100	1273	0	0	0	2351
scaffold_129	1	1249	ChVII	1798386	1797138	100	1249	0	0	0	2307
scaffold_130	1	1228	ChII	1809969	1808742	100	1228	0	0	0	2268
scaffold_131	1	1227	ChII	5799358	5798132	100	1227	0	0	0	2266
scaffold_133	1	1219	ChVII	2875916	2877134	100	1219	0	0	0	2252
scaffold_134	1	1214	ChVI	3997280	3996067	100	1214	0	0	0	2242
scaffold_135	1	1205	ChV	1754356	1755560	100	1205	0	0	0	2226
scaffold_138	1	1198	ChII	1995267	1994070	100	1198	0	0	0	2213
scaffold_139	1	1189	ChII	1810497	1811685	100	1189	0	0	0	2196
scaffold_140	1	1185	ChIV	4545164	4546348	100	1185	0	0	0	2189
scaffold_141	1	1184	ChII	2013021	2014204	100	1184	0	0	0	2187
scaffold_142	5	407	ChI	3219761	3219360	97.03	404	9	3	0	676

scaffold_142	123	1170	ChVI	1933389	1932342	98.85	1048	12	0	0	1869
scaffold_143	1	1170	ChVI	3656405	3655236	100	1170	0	0	0	2161
scaffold_144	1	1168	ChII	6229052	6230219	100	1168	0	0	0	2158
scaffold_146	1	1166	ChVI	54695	55860	100	1166	0	0	0	2154
scaffold_147	1	1164	ChVI	1955591	1956754	100	1164	0	0	0	2150
scaffold_148	1	1146	ChVI	1913392	1914537	100	1146	0	0	0	2117
scaffold_149	1	1146	ChIV	3437764	3438909	100	1146	0	0	0	2117
scaffold_150	1	1129	ChII	2010177	2011305	100	1129	0	0	0	2085
scaffold_151	1	1127	ChII	2011794	2012920	100	1127	0	0	0	2082
scaffold_152	1	1115	ChVII	1800803	1799689	100	1115	0	0	0	2060
scaffold_153	1	1106	ChVII	12704	13809	100	1106	0	0	0	2043
scaffold_154	1	1106	ChII	588339	589444	100	1106	0	0	0	2043
scaffold_155	1	1104	ChIV	1548306	1549409	100	1104	0	0	0	2039
scaffold_156	1	1104	ChVII	11468	10365	100	1104	0	0	0	2039
scaffold_157	1	1099	ChV	438818	437720	100	1099	0	0	0	2030
scaffold_158	1	1099	ChIV	4333371	4334469	100	1099	0	0	0	2030
scaffold_159	1	1098	ChI	3547634	3546537	100	1098	0	0	0	2028
scaffold_160	2	406	ChI	3243807	3243403	99.01	405	4	0	0	726
scaffold_160	403	1084	ChV	59437	60119	99.12	683	5	1	0	1227
scaffold_161	29	1080	ChI	6835803	6834752	100	1052	0	0	0	1943
scaffold_162	1	1075	ChII	2030489	2031563	100	1075	0	0	0	1986
scaffold_163	1	1058	ChI	3552953	3551896	100	1058	0	0	0	1954

scaffold_164	1	1056	ChVI	1926512	1927567	100	1056	0	0	0	1951
scaffold_165	1	119	ChII	3047061	3047179	97.48	119	3	0	2E-52	204
scaffold_165	92	1056	ChII	3522801	3523765	100	965	0	0	0	1783
scaffold_166	1	1052	ChII	5800412	5799361	100	1052	0	0	0	1943
scaffold_167	1	1042	ChV	435915	436956	100	1042	0	0	0	1925
scaffold_168	1	1041	ChI	3228994	3230034	100	1041	0	0	0	1923
scaffold_169	1	1029	ChVI	70887	71915	100	1029	0	0	0	1901
scaffold_170	1	1026	ChIII	4449123	4450148	100	1026	0	0	0	1895
scaffold_171	1	1016	ChII	2001766	2000751	100	1016	0	0	0	1877
scaffold_172	1	997	ChIII	1808981	1809977	100	997	0	0	0	1842
scaffold_173	1	994	ChII	2017802	2018795	100	994	0	0	0	1836
scaffold_174	1	993	ChVII	1578218	1577226	100	993	0	0	0	1834
scaffold_175	1	982	ChIII	1813156	1812175	100	982	0	0	0	1814
scaffold_176	1	981	ChV	1273030	1274010	100	981	0	0	0	1812
scaffold_177	1	970	ChIV	9438	10407	100	970	0	0	0	1792
scaffold_178	1	969	ChVI	1931568	1930600	100	969	0	0	0	1790
scaffold_179	1	965	ChIV	1544796	1543832	100	965	0	0	0	1783
scaffold_180	1	954	ChI	3180996	3181949	100	954	0	0	0	1762
scaffold_181	1	924	ChI	327	1250	100	924	0	0	0	1707
scaffold_182	11	886	ChVI	1947008	1947883	99.66	876	3	0	0	1602

The RTU-C30 sequences with short ectopic insertions are highlighted in green.

Table A11. Gene Ontology of some newly-predicted QM6a genes (Additional File: Table B3)

**Biological process**	**Gene number**	**Known genes**
ADP ribosylation	1	*sec7*, *arl3*
Autophagy	1	*atg11*
CAZyme	3	TrC0724C (Glycosyl transferase family35)<br>*chs6* (chitin synthase 6)<br>TrE1217W (glycosylhydrolase 7-2)
Cell cycle control	2	*cdc4*, *cdc15*
Cell wall		*acw8*,*acw9*
Chromatin and histone modification	4	*eaf3, hda3, hta1*, *clr4*, *rsc3*
Cytoskeleton	4	*myo1*, *kip2*, *klp2*, *rai14*, *mlp2*
Genome integrity	10	*dna2*, *ddi1*, *dnl4*, *rad50*, *rad51*, *rad57*, *srs2*, *pif1*, *rrm3*, *hrq1*, *slx4*, *smc1*
Iron sulfur cluster assembly	1	*isa2*
Mitochondria	5	*cor1*, *cox23*, *cox24*, *isd11*, *mam33*, *mdn1*, *rsm27*, *tom7*
RNA splicing or processing	3	*sap62*, *msl5*, *usb1*, *exo2/xrn1*
Meiotic silencing by unpaired DNA	1	*sad1* (RNA-dependent RNA polymerase)
Nucleotide metabolism	1	*hnt1*
Oxidoreduction	4	*adh5* (TrA0261), Trb1207W, TrE0197C, TrE0242C,
Protein degradation	4	*apc11*, *cdc14*, *das1*, *elc1*, *nas6*, *outb1*, *rai14*, *tul1*, *udf1*, TrB1864C (ubiquitin conjugating enzyme)
Protein kinase	1	*ck1a*

Protein palmitoylation	2	*akr1, akr2*
Transcription factors	2	*cad1, cat8, cup9, eaf1, mot1,*
Translation	9	*rpl13, rpl37a,*
Transport	7	*TrB1425C, hxt2*
rDNA	23	
snRNA	2	
tRNA	70	

Table A12. Sequencing quality of 18 different fungal genomes*

Species	Phylum	Strain	Database	GeneBank	Contigs	N (bp)	N (%)
Trichoderma reesei	Ascomycota	QM6a	-	-	7	0	0%
Neurospora crassa	Ascomycota	OR74A	NCBI	GCA_000182925.2	20	40775	0.1%
Penicillium chrysogenum	Ascomycota	P2niaD18	NCBI	GCA_000710275.1	4	1300	0%
Mycosphaerella graminicola	Ascomycota	IPO323	JGI	GCA_000219625.1	21	5997	0.02%
Fusarium fujikuroi	Ascomycota	IMI 58289	NCBI	GCA_900079805.1	12	23886	0.05%
Aspergillus nidulans	Ascomycota	FGSC A4	NCBI	GCA_000011425.1	8	10467	0.04%
Saccharomyces cerevisiae	Ascomycota	s288c	NCBI	GCA_000146045.2	16	0	0%
Schizosaccharomyces pombe	Ascomycota	972h-	NCBI	GCA_000002945.2	3	402	0%
Candida glabrata	Ascomycota	CBS138	NCBI	GCA_000002545.2	12	300	0%
Ustilago maydis	Basidiomycota	521	NCBI	GCA_000328475.2	23	22700	0.11%
Coprinopsis cinerea	Basidiomycota	Okayama7#130	NCBI	GCA_000182895.1	94	34	0%
Cryptococcus neoformans	Basidiomycota	JEC21	NCBI	GCA_000091045.1	14	1860	0.01%
Leptosphaeria maculans	Ascomycota	v23.1.3	JGI	GCA_000230375.1	41	1128152	2.51%
Sporisorium reilianum	Basidiomycota	SRZ2	NCBI	GCA_000230245.1	23	336654	1.84%
Flammulina velutipes	Basidiomycota	KACC42780	NCBI	GCA_000633125.1	11	374554	1.05%
Puccinia graminis	Basidiomycota	CRL 75-36-700-3	JGI	GCA_000149925.1	392	7123336	8.04%
Melampsora laricis-populina	Basidiomycota	98AG31	JGI	GCA_000204055.1	462	3446329	3.41%
Rhodotorula graminis	Basidiomycota	WP1	JGI	GCA_001329695.1	26	236450	1.13%

* The faSize program (https://github.com/ENCODE-DCC/kentUtils/tree/master/src/utils/faSize) was used to calculate the overall number and percentage of unresolved bases (N) in each fungal genome. The genome sequences with >1.1% N are low-quality (marked in red).

Table A13. RIP indices of various sequences in QM6a and *Neurospora crassa*

Organism	Sequences	TpA/ApT	(CpA+TpG)/(ApC+GpT)
QM6a	*MAT 1-2-1*	0.37	1.84
	All predicted genes	0.53 ± 0.36	1.32 ± 0.19
	Whole genome	0.79	1.26
	Large rDNA tandem repeats (n = 9)	1.02	1.05
	5S rDNA repeats (n = 39)	1.27 ± 0.30	0.90 ± 0.12
	AT-rich blocks (≥3 kb; n = 167)	1.48 ± 0.10	0.43 ± 0.26
	AT-rich blocks (2<L<3 kb; n = 8)	1.37 ± 0.36	0.74 ± 0.39
	AT-rich blocks (1<L≤2 kb; n = 53)	0.95 ± 0.29	1.14 ± 0.25
	AT-rich blocks (1 kb; n = 276)	0.83 ± 0.21	1.24 ± 0.19
	AT-rich blocks (0.5 kb; n = 1845)	0.79 ± 0.20	1.27 ± 0.20
	gyspy LTR (n = 9)	1.61 ± 0.11	0.30 ± 0.18
	Copia LTR (n = 6)	1.48 ± 0.12	0.57 ± 0.25
	MULE-MuDR (n = 21)	1.30 ± 0.40	0.81 ± 0.42
N. crassa	*MFa* (AF397732.1)	0.57	1.27
	All predicted genes	0.65 ± 0.13	1.2 ± 0.11
	Whole genome (v12 OR74)	0.91	1.1

Table A14. Repeat-induced C-to-T mutations observed in the *hph* alleles in all F1 progeny

Sequence	(CA<->TA) + (TG<->TA)	(CG<->TG) + (CG<->CA)	(CC<->TC) + (GG<->GA)	(CT<->TT) + (AG<->AA)	CpA (%)	Cp (%)	CpC (%)	Cp (%)
*blr1*Δ (*MAT1-1*; F0)	0	0	0	0	0	0	0	0
*blr1*Δ (*MAT1-2*; F0)	0	0	0	0	0	0	0	0
*blr1*Δ x *blr1*Δ (F1 #1)	27	16	0	0	62.8	37.2	0	0
*blr1*Δ x *blr1*Δ (F1 #2)	27	16	0	0	62.8	37.2	0	0
*blr1*Δ x *blr1*Δ (F1 #3)	26	19	0	1	56.5	41.3	0	2.2
*blr1*Δ x *blr1*Δ (F1 #4)	26	19	0	1	56.5	41.3	0	2.2
*blr1*Δ x *blr1* (F1 #4)	22	19	0	1	52.4	45.2	0	2.4
*env1*Δ (*MAT1-1*; F0)	0	0	0	0	0	0	0	0
*env1*Δ (*MAT1-2*; F0)	0	0	0	0	0	0	0	0
*blr1*Δ x *env1*Δ (F1 #1, *blr1* Δ*::hph*)	7	11	0	1	36.8	57.9	0	5.3
*blr1*Δ x *env1*Δ (F1 #2, *blr1* Δ*::hph*)	7	11	0	1	36.8	57.9	0	5.3
*blr1*Δ x *env1*Δ (F1 #1, *env1*Δ*::hph*)	0	0	0	0	0	0	0	0
*blr1*Δ x *env1*Δ (F1 #4, *env1*Δ*::hph*)	0	0	0	0	0	0	0	0
*env1*Δ x *env1*Δ (F1 #1)	0	0	0	0	0	0	0	0
*env1*Δ x *env1*Δ (F1 #2)	0	0	0	0	0	0	0	0
*env1*Δ x *env1*Δ (F1 #3)	0	0	0	0	0	0	0	0
*env1*Δ x *env1*Δ (F1 #4)	0	0	0	0	0	0	0	0
*ku70*Δ (*MAT1-1*; F0)[1]	0	0	0	0	0	0	0	0

*ku70*Δ (*MAT1-2*; F0) [1]	0	0	0	0	0	0	0	0
*env1*Δ x *ku70*Δ (F1 #1, *env1*Δ*::hph*)	0	0	0	0	0	0	0	0
*env1*Δ x *ku70*Δ (F1 #2, *env1*Δ*::hph*)	0	0	0	0	0	0	0	0
*env1*Δ x *ku70*Δ (F1 #2, *ku70*Δ*::hph*)	0	0	0	0	0	0	0	0
*env1*Δ x *ku70*Δ (F1 #3, *ku70*Δ*::hph*)	0	0	0	0	0	0	0	0
*ku70*Δ x *ku70* (F1 #3)	0	0	0	0	0	0	0	0
*ku70*Δ x *ku70* (F1 #4)	0	0	0	0	0	0	0	0
*ku70*Δ x *ku70*Δ (F1 #1)	0	0	0	0	0	0	0	0
*ku70*Δ x *ku70*Δ (F1 #2)	0	0	0	0	0	0	0	0
*ku70*Δ x *ku70*Δ (F1 #3)	0	0	0	0	0	0	0	0
*ku70*Δ x *ku70*Δ (F1 #4)	0	0	0	0	0	0	0	0
*ku70*Δ *env1*Δ (*MAT1-1*; F0; *ku70*Δ*::hph*)	0	0	0	0	0	0	0	0
*ku70*Δ *env1*Δ (*MAT1-1*; F0; env1Δ*::hph*)	0	0	0	0	0	0	0	0
*ku70*Δ *env1*Δ x *ku70 env1* (F1 #3, *ku70*Δ*::hph*)	22	24	0	0	47.8	52.2	0	0
*ku70*Δ *env1*Δ x *ku70 env1* (F1 #4, *ku70*Δ*::hph*)	22	24	0	0	47.8	52.2	0	0
*ku70*Δ *env1*Δ x *ku70 env1* (F1 #2, *env1*Δ*::hph*)	23	18	0	0	56.1	43.9	0	0
*ku70*Δ *env1*Δ x *ku70 env1* (F1 #3, *env1*Δ*::hph*)	23	18	0	0	56.1	43.9	0	0

1. The *hph* alleles in the parental (F0) *ku70*Δ or *ku70*Δ *env1*Δ strains carried one CA to TA and four CC to TC point mutations. These four C-to-T point mutations in *ku70*Δ*::hph* were not included in the comparison of RIP sequence preference.

Table A15. *Saccharomyces cerevisiae* Ty elements by chromosome.

Chromosome number	Number of insertions (# of insertion ≥ 5kb)					Total Ty insertions on chromosomes
	Ty1	Ty2	Ty3	Ty4	Ty5	
I	5 (1)	1	1	0	0	5
II	8 (2)	3 (1)	1	2	0	16
III	8	4 (1)	1	1	2 (1)	23
IV	20 (5)	6 (3)	9	3	0	36
V	11 (2)	9	3	3	2	30
VI	7	3 (1)	0	1	0	13
VII	18 (3)	6 (2)	7 (1)	4	1	41
VIII	8 (1)	4	3	3 (1)	1	20
IX	4	2	2(1)	1	0	7
X	13 (2)	5	1	4 (1)	0	23
XI	9	1	2	1	1	13
XII	18 (4)	4 (2)	5	3	0	27
XIII	15 (4)	3	0	4	0	23
XIV	5 (2)	2 (1)	4	3	0	10
XV	18 (2)	4 (2)	5	2	0	27
XVI	14 (4)	4	4	3 (1)	0	25
Overall	181 (32)	61	48 (2)	38 (3)	7 (1)	335

Full chromosome sequences were obtained from the *Saccharomyces* Genome Database. (SGD). The Ty elements were identified by RepeatMasker (http:www.repeatmasker.org/) against the Malvid section of the MIPS repeat database (mipsREdat_9.3p). RepeatMasker results

were filtered to retain the high-confidence hits (length >= 140, Smith–Waterman local similarity scores >= 450). It should be noted that the final results are close to those published previously [65, 66].

Table A16. Transposable elements in 12 well-assembled fungal genomes

Species	Genome size (bp)	Repetitive sequences (bp)	Overall	Class I (retrotransposons)							Class II (transposons)					
				Tad1-LINE	*I*-LINE	*Jockey*-LINE	other LINEs	*Copi*-LTR	*Gypsy*-LTR	other LTRs	*CMC-EnSpm*	*hAT*	*MULE-MuDR*	*TcMar -Fot1*	*PIF-Harbinger-like*	Others
			copy number													
Trichoderma reesei	34922528	42035	70	0	0	4	14	8	10	4	6	0	21	0	0	3
Fusarium fujikuroi	43832314	330124	286	0	1	0	2	19	150	4	3	1	1	79	0	26
Neurospora crassa	41037538	795049	441	267	10	0	5	6	124	5	1	1	1	13	0	8
Mycosphaerella graminicola	39686251	792433	695	142	0	0	1	101	417	12	3	0	0	14	0	5
Penicillium chrysogenum	32498524	258859	273	54	3	0	2	42	42	4	1	17	3	50	0	55
Aspergillus nidulans	29828291	736219	791	17	1	66	1	126	178	115	2	104	36	76	0	69
Saccharomyces cerevisiae	12071326	396333	361	0	3	0	4	287	48	5	2	3	0	0	0	9
Schizosaccharomyces pombe	12571820	127770	218	0	0	0	0	1	198	10	1	0	0	0	0	8
Candida glabrata	11123113	21966	40	0	2	0	1	4	14	7	0	2	0	0	0	10
Ustilago maydis	19643891	188065	166	0	4	0	3	137	4	6	1	0	2	0	0	9
Cryptococcus neoformans	19051922	801159	645	0	4	0	41	55	321	147	0	0	2	0	62	13
Coprinopsis cinerea	36294355	1516981	624	1	0	0	4	128	445	14	12	5	1	0	0	14

Table A17. Repetitive sequences in different chromosomal regions

Chromosome regions		Subtelomere		Centromere		Long AT-rich block (≥3 kb)		Short AT-rich block (05-3 kb)		Not AT-rich regions		Overall	
		Base pairs (copy number)											
Class I	LINE	187 bp	1	148 bp	1	0	0	0	0	4887 bp	16	5222 bp	18
	copia-LTR	0	0	0	0	6171 bp	6	0	0	1597 bp	2	7768 bp	8
	Gypsy-LTR	2683 bp	3	346 bp	1	4399 bp	5	0	0	171 bp	1	7599 bp	10
	others LTRs	0	0	0	0	621 bp	1	0	0	1110 bp	3	1731 bp	4
Class II	*CMC-EnSpm*	621 bp	2	536 bp	3	724 bp	1	0	0	0	0	1881 bp	6
	MULE-MuDR	0	0	6646 bp	10	1500 bp	2	184 bp	1	5880 bp	8	14210 bp	21
	Other	0	0	0	0	0	0	0	0	1500 bp	2	1500 bp	3
rDNA (28S-5.8S-18S)		0	0	0	0	0	0	0	0	50004 bp	9	50004 bp	9
Satellite (+5S rDNA)		267 bp	2	362 bp	2	0	0	748 bp	6	4147 bp	33	5524 bp	43
Simple repeats		0	0	1825bp	4	0	0	0	0	976 bp	5	2801 bp	9
Overall		3758 bp	8	9863 bp	21	13415 bp	15	923 bp	7	70272 bp	79	98240 bp	130

Table A18. Repetitive sequences in seven QM6a chromosomes

QM6a chromosomes		I	II	III	IV	V	VI	VII	Overall
		Base pairs (copy number)							
Class I	LINE	807 (4)	1486 (4)	2063 (7)	148 (1)	718 (2)	0 (0)	0 (0)	5222 (18)
	Copia-LTR	666 (2)	4270 (3)	0 (0)	1202 (1)	1630 (2)	0 (0)	0 (0)	7768 (8)
	Gypsy-LTR	171 (1)	4489 (5)	282 (1)	1001 (1)	0 (0)	1656 (2)	0 (0)	7599 (10)
	other LTRs	0 (0)	893 (2)	0 (0)	621 (1)	217 (1)	0 (0)	0 (0)	1731 (4)
Class II	*CMC-EnSpm*	534 (2)	0 (0)	0 (0)	0 (0)	724 (1)	623 (3)	0 (0)	1881 (6)
	MULE-MuDR	725 (2)	2991 (4)	3416 (6)	2165 (3)	1996 (3)	0 (0)	2917 (3)	14210 (21)
	Other transposons	521 (1)	0 (0)	0 (0)	0 (0)	979 (1)	0 (0)	0 (0)	1500 (2)
Satellite (w/o 5S rDNA)		207 (1)	0 (0)	267 (2)	0 (0)	0 (0)	155 (1)	0 (0)	629 (4)
5S rDNA		127 (1)	1008 (8)	507 (4)	373 (3)	124 (1)	2131 (17)	625 (5)	4895 (39)
Simple repeats		0 (0)	780 (2)	849 (2)	189 (1)	0 (0)	0 (0)	983 (4)	2801 (9)
18S-5.8S-26S rDNA		0 (0)	0 (0)	0 (0)	0 (0)	0 (0)	50004 (9)	0 (0)	50004 (9)
Overall		3758 (14)	15917 (28)	7384 (22)	5699 (11)	6388 (11)	56693 (32)	4525 (12)	98240 (130)
Percentage in each chromosome		0.05%	0.26%	0.14%	0.13%	0.15%	1.42%	0.12%	0.29%

Table A19. Partitioning of four gene clusters by AT-rich blocks.

Gene cluster	Systematic name	Start	Stop	Length (bp)	Gene function	QM6a ID	Glucose (48 hrs)			Straw (24 hrs)			Straw (24 hrs) Glucose (5 hrs)	
							I	II	III	I	II	III	I	II
A	AT-block	6186000	6186500	500										
	TrB1966W	6187413	6188558	1146	Hypothetical protein	111717	169.8	114.4	133.0	235.8	254.4	281.1	27.1	51.0
	TrB1967C	6188844	6190301	1458	GH30 endo-β-1,4-xylanase	69276	2.2	0.0	0.0	165.4	166.4	155.6	1.3	0.0
	TrB1968W	6192320	6193759	1440	Glucuronoyl esterase *cip2*	123940	0.0	0.8	0.9	692.1	573.7	693.6	1.3	2.1
	AT-block	6194500	6195000	500										
	TrB1969W	6195357	6196133	777	β-1,4-glucuronan lyase *trgL*	69189	0.0	0.0	0.0	1.3	0.0	0.0	2.4	0.0
	AT-block	6196000	6196500	500										
	TrB1970W	6197486	6198336	851	Hypothetical protein	69181	1.4	4.9	1.7	8.3	7.4	11.4	0.0	7.9
	TrB1971C	6198686	6201772	3087	GH2 β-mannosidase	69245	0.4	0.0	0.0	74.3	84.9	71.3	0.6	0.0
	AT-block	6202500	6203000	500										
	TrB1972C	6203699	6205243	1545	Nitrate transport *nit10*	111724	2.9	1.7	2.7	13.6	13.8	12.4	0.0	0.0
	AT-block	6205500	6206000	500										
	TrB1973C	6206646	6208604	1959	Iron-sulfur nitrate reductase *nit6*	69291	0.5	0.0	0.0	0.0	0.0	0.0	0.0	0.0
	TrB1974C	6208839	6209978	1140	*nit2*	69210	0.0	0.0	0.0	0.0	0.0	0.0	0.0	0.0
	TrB1975W	6211369	6214122	2754	NAD(P) nitrate reductase *nit3*	81955	0.0	0.0	0.0	1.1	0.8	0.3	0.0	0.0
	TrB1976W	6215458	6216291	834	Hypothetical protein, HFBs	111729	0.0	1.4	1.5	1.2	0.0	0.0	0.0	0.0
	AT-block	6216000	6234656	18656										
	tel2R	6216000	6234656	18656										

B	AT-block	4387500	4388000	500										
	TrD1393W	4389973	4392937	2965	*GH31*	82235	2.3	0.9	1.9	83.8	115.3	91.3	58.7	20.2
	AT-block	4393000	4394000	1000										
	TrD1394C	4393986	4396830	2845	Transcription factors	52499	10.0	5.5	15.7	24.7	18.9	19.7	7.2	9.5
	TrD1395W	4399762	4401029	1268		-	0.0	2.4	1.3	1.0	1.1	2.5	0.0	1.5
	TrD1396W	4403921	4405880	1960		70191	71.4	85.6	85.3	85.0	56.6	93.6	33.9	38.2
	AT-block	4406000	4406500	500										
	TrD1397W	4409318	4412707	3390		28353	25.0	22.5	19.3	28.5	28.9	21.0	8.8	6.2
	TrD1398C	4413809	4416429	2621	*cel3c*	82227	0.8	1.4	0.5	56.9	57.1	49.4	2.2	0.0
	TrD1399W	4420692	4421159	468		-	0.0	0.0	0.0	7.5	18.6	2.0	0.0	0.0
	AT-block	4425000	4425500	500										
	TrD1400C	4426153	4427461	1309		124084	22.7	23.8	35.3	1.9	2.1	1.6	0.0	0.0
	TrD1401C	4429434	4429847	414		-	2.9	0.0	0.0	0.0	0.0	0.0	0.0	0.0
	TrD1402W	4432911	4434484	1574		-	8.1	9.3	4.5	1.8	0.0	1.5	0.0	3.8
	TrD1403C	4437493	4439076	1584		38372	24.3	34.4	34.7	26.4	18.2	21.7	8.1	7.2
	TrD1404W	4440001	4440893	893		70175	46.2	40.8	20.7	76.4	121.6	73.0	22.7	35.4
	TrD1405C	4441023	4442127	1105		5894	3.0	7.9	6.1	12.7	8.4	6.4	3.6	4.2
	TrD1406C	4442608	4443867	1260		112147	0.0	0.0	0.0	0.0	0.0	0.0	0.0	0.0
	TrD1407W	4444617	4448064	3448		112146	0.4	1.0	1.1	1.7	2.7	0.7	7.7	2.4
	AT-block	4447500	4452000	4500										
	TrD1408C	4452403	4453208	806		124083	42.0	25.8	20.4	13.3	12.7	14.6	18.9	35.9
	TrD1409C	4453980	4454781	802		-	1.4	4.8	0.0	2.7	1.5	1.1	5.0	2.0

	AT-block	4454000	4454500	500										
	TrD1410W	4455937	4457496	1560		52489	243.6	256.6	164.1	67.7	67.8	64.1	107.7	141.6
	TrD1411C	4457706	4458923	1218	*GH28*	70186	2.5	9.5	4.1	2.5	1.8	2.0	3.0	2.3
	TrD1412C	4459530	4460950	1421	*pgx1*	112140	0.0	0.0	0.0	25.7	31.7	33.3	2.9	0.0
	AT-block	4461500	4462000	500										
	TrD1413W	4462716	4464576	1861	Transport	5890	0.7	0.0	0.0	2.0	4.9	1.6	1.2	0.9
	TrD1414C	4464845	4465393	549		70201	55.4	76.1	59.2	41.8	33.1	43.3	93.1	96.0
	AT-block	4465500	4466000	500										
	AT-block	3779500	3781000	1500										
	TrG1193W	3780974	3781788	815	*egl2/cel5* endo-β-1,4-glucanase	104060	0.0	2.8	0.0	0.0	0.0	0.0	0.0	0.0
	TrG1194C	3782185	3783386	1202		104059	0.0	0.0	0.0	0.0	0.0	0.9	0.0	0.0
	TrG1195C	3784087	3785522	1436		120312	4.0	0.0	2.0	2661.9	5857.6	3030.8	1.5	0.0
	TrG1196C	3786525	3786936	412		-	2.9	0.0	0.0	5.6	48.3	18.5	0.0	0.0
	TrG1197C	3787214	3787838	625		-	579.0	339.1	432.6	215.8	411.4	193.4	569.2	339.3
C	TrG1198W	3788216	3788979	764		-	5.8	1.7	14.4	21.5	39.9	27.1	18.4	16.4
	TrG1199W	3789733	3790810	1078		56896	60.0	65.2	44.3	23.6	15.8	27.5	46.8	97.0
	TrG1200W	3791640	3792665	1026	PI4-phosphate 5-kinase	2492	0.0	0.0	0.0	5.8	7.3	5.6	0.0	1.4
	AT-block	3793000	3793500	500										
	TrG1201W	3793759	3795158	1400		104054	0.0	0.0	0.0	0.0	0.0	0.0	0.0	0.0
	TrG1202W	3795577	3797460	1884	GH79 ß-glucuronidase	72568	0.0	0.0	0.0	0.0	0.0	0.0	0.0	0.0
	TrG1203C	3797586	3799386	1801	Permease/transporter	56289	0.0	3.0	0.0	2.6	0.7	4.2	0.0	0.0
	TrG1204W	3800146	3801264	1119	Rhamnogalacturonyl hydrolase	57179	0.0	0.0	0.0	1.8	7.6	2.9	0.0	0.0

	AT-block	3801000	3802000	1000									
	AT-block	3802500	3803000	500									
	TrG1205W	3803098	3803421	324		104050	0.0	0.0	0.0	0.0	0.0	0.0	0.0	0.0
	AT-block	3803500	3804000	500									
	TrG1206C	3805219	3806829	1611	*cbh* cellobiohydrolase	72567	2.9	4.1	6.2	4748	8696	4844	7.7	8.1
	AT-block	3807500	3809500	2000									
	AT-block	2213000	2214000	1000									
	TrE0668C	2214464	2215057	594		102500	2.4	0.0	2.9	0.0	2.5	0.0	0.0	0.0
	TrE0669C	2216491	2217596	1106	*axe1* acetyl xylan esterase	73632	3.3	2.6	1.4	3129	5083	3687	6.0	1.6
	AT-block	2217500	2218500	1000									
	AT-block	2219000	2219500	500									
D	TrE0670C	2219457	2220467	1011	*cip1*	73638	0.0	1.2	0.0	1378	1345	1551	5.8	0.0
	AT-block	2220500	2221000	500									
	TrE0671C	2221294	2222382	1089	*egl4*, β-1,6-N-acetylglucosaminyl transferase	73643	4.9	1.1	2.4	4590	3774	5037	8.8	5.5
	AT-block	2223000	2224000	1000									
	AT-block	2224500	2225000	500									
