# Supplementary material for: Trichoderma reesei complete genome sequence, repeat-induced point mutation, and partitioning of CAZyme gene clusters
Source: Biotechnol Biofuels. 2017 Jul 3;10:170. doi: 10.1186/s13068-017-0825-x (PMC5496416; doi:10.1186/s13068-017-0825-x)
Supplement: Supplementary file 4 — Additional file 4. Sequence alignments of the 24 centromeric repeats. [file 13068_2017_825_MOESM4_ESM.pdf]

Appendix A3. Sequence alignments of the 24 centromeric repeats

```
                        *        20         *        40         *        60         *        80         *       100
cen1-CR1 : ---------------------------------------------------------------------------------------------------- :    -
cen1-CR2 : ---------------------------------------------------TTACTTTTATATATATAATAAGGTATTAAAACTATTACTTATATATATT :   49
cen1-CR3 : ------------------------------AATATAACTTTTATACTTTTTCTTATTTTAATTATAAATTAAAATATTATTTAAATAAATAATATATATA :   70
cen1-CR4 : ---------------------------------------------------------------------------------------------------- :    -
cen1-CR5 : ---------------------------------------------------------------------------------------------------- :    -
cen2-CR1 : ---------------------------------------------------------------------------------------------------- :    -
cen2-CR2 : GGCGCTCTCTTGTCAGGCAAGCTCATTGGAGTTGTTTCGTTGACAGACATTCTCAATATCACTTATTAATAAAATAAAAAAAAATAAAGCAGCTATAATA :  100
cen2-CR3 : GGCGCTCTCTTGTCAGGCAAGCTCATTGGAGTTGTTTCGTTGACAGACATTCTCAATATCATACTTATACTAAAATT----------------------- :   77
cen2-CR4 : ---------------------------------------------------------------------------------------------------- :    -
cen2-CR5 : ---------------------------------------------------------------------------------------------------- :    -
cen3-CR1 : ---------------------------------------------------------------------------------------------------- :    -
cen3-CR2 : ---------------------------------------------------------------------------------------------------- :    -
cen3-CR3 : ---------------------------------------------------------------------------------------------------- :    -
cen3-CR4 : ---------------------------------------------------------------------------------------------------- :    -
cen4-CR1 : ---------------------------------------------------------------------------------------------------- :    -
cen4-CR2 : ---------------------------------------------------------------------------------------------------- :    -
cen4-CR3 : ---------------------------------------------------------------------------------------------------- :    -
cen4-CR4 : ----------------------------------------TAAAATAATTAAAATATATTTTATTAAAATAAAAAGCTTAAATAGTAATATATATAAGGT :   60
cen4-CR5 : ---------------------------------------------------------------------------------------------------- :    -
cen5-CR1 : ---------------------------------------------------------------------------------------------------- :    -
cen6-CR1 : ---------CATCCTCATAGTATCCCGGCTCATTCGAGATGACATTGCCAGGCGCCAACGGGACTTCGGTTTAATATTTTATAAATAAAATCTTAAAAAA :   91
cen7-CR1 : ---------------------------------------------------------------------------------------------------- :    -
cen7-CR2 : ---------------------------------------------------------------------------------------------------- :    -
cen7-CR3 : ---------------------------------------------------------------------------------------------------- :    -

                        *       120         *       140         *       160         *       180         *       200
cen1-CR1 : --------------------------------------------------------------------------------------------------TA :    2
cen1-CR2 : ATATAATTATAATAAGCTTATATACTTATAAAAACTTTTTAATAAAGAAATAAAAGTTTTATAAGTAGCTTAATAAGTTATAAAAGCTATATAATAAGTA :  149
cen1-CR3 : ATATTAATAAGTTTTATAAAAATAAAGTTAATATATATTTATTAAATAGCTAAAGTATTTATA---AAGTTAAATAATAAAACTTAATATTAAAAAAAGC :  167
cen1-CR4 : ---------------------------------------------------------------------------------------------------- :    -
cen1-CR5 : ---------------------------------------------------------------------------------------------------- :    -
cen2-CR1 : ---------------------------------------------------------------------------------------------------- :    -
cen2-CR2 : TAATATTAATTAAAGTAAATAATAAAAATAGTAAGTTTTTAAAAACTTATAAAAATAAATAAAAGTAATTAAATCTTTTATAAGCTATATATTAAATAAA :  200
cen2-CR3 : ---------------------------------------------------------------------------------------------------- :    -
cen2-CR4 : ---------------------------------------------------------------------------------------------------- :    -
cen2-CR5 : ---------------------------------------------------------------------------------------------------- :    -
cen3-CR1 : ---------------------------------------------------------------------------------------------------- :    -
cen3-CR2 : ---------------------------------------AATAATGTCCTCAGGCGCCGAGTCCGCATTCTGGAGCCTTATATCAGCCTCCAGTCGAGCT :   61
cen3-CR3 : -------------------------------------------TAATATAATATATATAATAAACTACTTAACTTACTTAGTATTAATTTTTATTTATAA :   57
cen3-CR4 : ---------------------------------------------------------------------------------------------------- :    -
cen4-CR1 : ---------------------------------------------------------------------------------------------------- :    -
cen4-CR2 : -----------ATAATAATATTTTTAAATAATAAAAGCTAGGTTAATATTATTAATAAAAGAATTATAAAAAATATATTATTATTACTTATTAACTTTAT :   89
cen4-CR3 : ---------------------------------------------------------------------------------------------------- :    -
cen4-CR4 : TTACTTATTAAATAAAAATAAAGCTATAAAATTATAAAATATATAATATTAAAGTTAAGTAATAAGACTTTTTATAATTAAAAAAAGCTAAAAATTAAAT :  160
cen4-CR5 : ---------------------------------------------------------------------------------------------------- :    -
cen5-CR1 : ---------------------------------------------------------------------------------------------------- :    -
cen6-CR1 : GTATAAAGGCTTTATAGCTATATTTATTAATAATATTATAATCTTTTTTATAATTATTAAAAAGTATATTAAATATTTAATAATAATATTTACTTTATTT :  191
cen7-CR1 : ---------------------------------------------------------------------------------------------------- :    -
cen7-CR2 : -------------------------TATTAATAATTTAAGATTTAATAATAAATTATAATATAAAAAGGCAGATATATTATTATAATAAAT--------- :   66
cen7-CR3 : ---------------------------------------------------------------------------------------------------- :    -
```

```
                    *       220         *       240         *       260         *       280         *       300
cen1-CR1 : ATATATTTATAAATAATAATATTATTAAAGCTAAAGCTAGTATTAATAATAATAGTAATAGTAATAGCTTTAAATTTTAATTATAATAATTATTAAAGCT :  102
cen1-CR2 : ATAAATAATATAAATAAAATAAAAAACTTAAAATACTTAAATAAAT-------------------------------------------TTATTAAAGCT :  206
cen1-CR3 : TATAATAATATTATTATATTATTTTTTTTATTATATATATATATATATATAATAGTAGGCATTTTTTAAGTTAA---------------GTGTTAAAGCT :  252
cen1-CR4 : ---------------------------------------------------------------------------------------------------- :    -
cen1-CR5 : ------------------------------------------------------------------------------------------TATTAAAGCT :   10
cen2-CR1 : ---------------------------------------------------------------------------------------------------- :    -
cen2-CR2 : GTGCTTAATATTATTAATATAT---------------------------TAAGTAAAATTACCTTTTTTAAAAA---------------TTATTAAAGCT :  258
cen2-CR3 : ------------------------------------------------------------------------------------------TATTAAAGCC :   87
cen2-CR4 : ---------------------------------------------------------------------------------------------------- :    -
cen2-CR5 : ---------------------------------------------------------------------------------------------------- :    -
cen3-CR1 : ---------------------------------------------------------------------------------------------------- :    -
cen3-CR2 : ATTAATAAAATAATT---------------------------------------------------------------------------TATTAAAGCT :   86
cen3-CR3 : TATAATAACTATATTATAATTTTATTAATAT----------------------------------------------------------TTATTAAAGCT :   99
cen3-CR4 : ----------------------------------------------------TACTTATATAAAAATTATTATA---------------TTATTAAAGCT :   33
cen4-CR1 : ---------------------------------------------------------------------------------------------------- :    -
cen4-CR2 : AATAATAAATACTTTTATTATTATATTATAAGAAATTTATAATTATTATAAATAAAAACTATAGCTTTATAATA---------------ATATTAAAGCT :  174
cen4-CR3 : ---------------------------------------------------------------------------------------------------- :    -
cen4-CR4 : ATAAAGCTATACTTATTAACTTAAAAATTAAAAAAAGGCTAAATAGTAATTAGCTAAATAATTAAATTATAATAAGCTTTTAATATAATTTATTAAAGCT :  260
cen4-CR5 : ---------------------------------------------------------------------------------------------------- :    -
cen5-CR1 : -------------TCCCCATTCCGGCCCTGCTTGAGGATGCAGACAAGGCGTTCGATACCCCCATCCACGGTCA---------------TTATTAAAGCC :   72
cen6-CR1 : ATAAATTAAAATATTATACTTTTTTTAACTAAGTTTTTTTTAATATTTTTATTAATAAAACTACTAGGCTTTTT---------------TTATTAAAGCT :  276
cen7-CR1 : ----------------------------------------------------TACTTATTAAATAAAGTATTAA---------------TTATTAAAGCT :   33
cen7-CR2 : -----------------------------------------------------------------------------------------ATATTAAAGCT :   77
cen7-CR3 : ------------------------------------------------------TAATATACTTTAAGTATTAAATCTTTTTTATAATAATCTTAAAGCT :   46

                    *       320         *       340         *       360         *       380         *       400
cen1-CR1 : AGTATATAATATAAAAAAGT-AAAAAGAGATAAAAAATAACTAAAGTTATAAAAAGTTAATATATAGTTATTAATTACTATTAAGTAATTTAAAAGTTAA :  201
cen1-CR2 : AGTATATAATATAAGGAAGT-AAAAAGAAAGAGAGAATAACTAAGGTTATAGGAAGTTAGTATATAGTTATTAATTACTACTAAGTAGTCTAAGAGTTAA :  305
cen1-CR3 : AGTATATAATATAAAAAAGT-AAAAAAAAAGAGAGGATAACTAAGGTTATAAAAAGTTAATATATAGTTATTAGTTACTACTAAGTGGTCTAAGAGTTAA :  351
cen1-CR4 : ----------------------------------------------------------------------------------AAGTGGTCTAGGAGTTAA :   18
cen1-CR5 : AGTATATAATATAAGAAAGT-AAAAAAAAAAAGAGGATAACTAAGGTTATAAAAAGTTAGTATATAGTTATTAATTATTACTAAGTGGTCTAGGAGTTAA :  109
cen2-CR1 : ---------------------------------------------------------------------------------------------------- :    -
cen2-CR2 : GGTATATAATATAAGGAAGT-AAAGAGAGAGAGAGAATAATTAAGGTTATAAAAAGTTAGTATATAGTTATTAATTACTACTAAGTGGTTTAAGAGTTAA :  357
cen2-CR3 : AGCATGTGATATAAGGAAGT-AAAAAGAGAGAGAGGATAACTAAGGTTATAGGAAGTTAGTATATAGTTATTAATTACTACCAAGTGGTCTAAGAGTTAA :  186
cen2-CR4 : ---------------------------------------------------------------------------------------------------- :    -
cen2-CR5 : ---------------------------------------------------------------------------------------------------- :    -
cen3-CR1 : ---------------------------------------------------------------------------------------------------- :    -
cen3-CR2 : AGTATATAATATAAAAAAGT-AAAAAAAAA--AAGGATAACTAAAGTTATAAAAAGTTAATATATAGCTATTAATTATTACTAAATAATATAAAAGTTAA :  183
cen3-CR3 : AGTATATAATATAAAAAAGT-AAAAAAAGAGAGAGAATAATTAAAGTTATAAAAAGTTAGTATATAATTATTAATTATTATTAAGTAGTCTAGGAGTTAA :  198
cen3-CR4 : AGTATATAATATAAAAAAGT-AAAAAGAGAGAGAAAATAATTAAGGTTATAAAAAGTTAGTATATAGCTATTAATTATTACTAAGTAGTCTAAAGGTTAA :  132
cen4-CR1 : ---------------------------------------------------------------------------------------------------- :    -
cen4-CR2 : AGTATATAATATAAAAAAGT-AAAAAGAGAAAGAGAATAACTAAAGTTATAAAAAATTAATATATAGTTATTAATTACTACTAGATAGTTTAAAAGTTAA :  273
cen4-CR3 : ---------------------------------------------------------------------------------------------------- :    -
cen4-CR4 : AGTATATAATATAAGAAAGT-AAAAAGAGAGAGAGAATAACTAAGGTTATAAAAAGTTAATATATAGTTATTAATTATTACTAAGTGGTTTAAGAGTTAA :  359
cen4-CR5 : ---------------------------------------------------------------------------------------------------- :    -
cen5-CR1 : AGCATGTAATATAAGGAAGT-AAAGAGAGA--GAGGATAACTAAGGTTATAGGAAGTTAGTATATAGTTATTAATTACTGCTAAGTAGTCTAAGAGTTAA :  169
cen6-CR1 : AGTATATAATATAAGGAAGT-AAAAAGAAAGAGAGGATAATTAAAGTTATAAAAAGTTAATATATAGTTATTAATTACTACTAAGTAGTTTAAGAGTTAA :  375
cen7-CR1 : AGTATATAATATAAAAAAGT-AAAAAGAGAGAGAGAATAACTAAGGTTATAAAAAGTTAATATATAGTTATTAATTACTATTATATAGTCTAAGAGTTAA :  132
cen7-CR2 : AGTATTTAATATAAGAAAGT-AAAAAAAAAAAGAGAATAACTAAAGTTATAAAAAGTTAGTATATAGTTATTAATTACTATTAAGTAATTTAAAAGTTAA :  176
cen7-CR3 : AGTATATAATATAAAAAAGTAAAAAAAAAAAAGAGATTAATTAAAATTATAAAAAGTTAGTATATAGTTATTAATTACTATTAAATAATTTAAAAGTTAA :  146
```

```
                         *        420         *        440         *        460         *        480         *        500
cen1-CR1 : TTATATAATTACTTTTAAAAGTAAGTGTTTATAAATAATTATATTTATTTTTAAACCTTTTTAAAATAAAAAAATAGTTTATTTTTTTTATAGCTATAAT :  301
cen1-CR2 : TTATATAATCACTTTTAAAGGTAGGTATTTATAAGTAACTATATTTATTTTTAAACCTTTTTAAAATAAAAGGATAGTTTACTTCTTTTATAGCTATAAT :  405
cen1-CR3 : TTATATAATTACTTTTAAAAGTAGGTGTTTATAAGTAACTATATTTATTTTTAAACCTCCTTAAAATAAAAAGACAGTTTACTTTTTTTATAGCTATAAT :  451
cen1-CR4 : TTATATAATCACTTCTAAAAGTAAGTGTTTATAAGTAACTGTATTTATCTTTAAACCTCCTTAAAATAAAAGGATAGTTTACTCCTTTTATAGCTATAAT :  118
cen1-CR5 : TTATATAATTACTTTTAAAAGTAGGTATTTATAAGTAACTGTATTTATTTTTAAACCTCCTTAAAATAAAAGGATAGTTTACTTTTTTTATAGCTATAAT :  209
cen2-CR1 : ---------------------------------------------------------------------------------------------------- :    -
cen2-CR2 : TTATATAATTACTTTTAAAAGTAGGTGTTTATAAGTAACTATATTTATTTTTAAACCTTTTTAAAATAAAAGGATAGTTTACTTTTTTTATAGCTATAAT :  457
cen2-CR3 : TTATATAATCACTTTTAAAGGTAGGTGTTTATAAGTGACTGTATTTATCTTTAAACCTCCTTAAAATAAAGGGATAGTTTACTCCTTTTATAACTGTAAT :  286
cen2-CR4 : ---------------------------------------------------------------------------------------------------- :    -
cen2-CR5 : ---------------------------------------------------------------------------------------------------- :    -
cen3-CR1 : ---------------------------------------------------------------------------------------------------- :    -
cen3-CR2 : TTATATAATTACTTTTAAAAGTAAGTGCTTATAAATAACTATATTTATTTTTAAACCTTTTTAAAATAAAGGGATAGTTTACTTTTTTTATAGCTATAAT :  283
cen3-CR3 : TTATATAATTACTTTTAAAAGTAAGTATTTATAAATAACTATATTTATTTTTAAACCTTTTTAAAATAAAAAAATAATTTACTTTTTTTATAATTATAAT :  298
cen3-CR4 : TTATATAATTACTTTTAAAAGTAAGTATTTATAAGTAATTATATTTATTTTTAAACCTTTTTAAAATAAAGAGATAGTTTACTTTTTTTATAGTTATAAT :  232
cen4-CR1 : ---------------------------------------------------------------------------------------------------- :    -
cen4-CR2 : TTATATAATTATTTTTAAAAGTAGGTATTTATAAGTAATTATATTTATTTTTAAACCTTTTTAAAATAAAAGGATAGTTTACTTTTTTTATAGCTATAAT :  373
cen4-CR3 : ---------------------------------------------------------------------------------------------------- :    -
cen4-CR4 : TTATATAATTACTTTTAAAAGTAGGTGTTTATAAGTAACTGTATTTATTTTTAAACCTTTTTAAAATAAAGGGATAGTTTACTTTTTTTATAGCTATAAT :  459
cen4-CR5 : ---------------------------------------------------------------------------------------------------- :    -
cen5-CR1 : TTATATAATTACTTTTAAAGGTAGGTGTTTATAAGTAACTGTATTTATTTTTAAACCTTCTTAAAATAAAGGGATAGTTTACTCCTTTTATAGCTATAAT :  269
cen6-CR1 : TTATATAATTACTTTTAAAAGTAAGTATTTATAAGTAACTATATTTATTTTTAAACCTTCTTAAAATAAAAAAATAGTTTACTTTTTTTATAGCTATAAT :  475
cen7-CR1 : TTATATAATTACTTTTAAAAGTAGGTGTTTATAAATAATTATATTTATTTTTAAACCTTTTTAAAATAAAAGGATAGTTTACTTTTTTTATAACTATAAT :  232
cen7-CR2 : TTATATAATTACTTTTAAAGGTAGGTATTTATAAATAACTATATTTATTTTTAAACCTTTTTAAAATAAAAAGATAGTTTACTTTTTTTATAGCTATAAT :  276
cen7-CR3 : TTATATAATTATTTTTAAAAATAAGTATTTATAAGTAATTATATTTATTTTTAAACCTTTTT-AAATAAAAGGATAGTTTACTTTTTTTATAGCTATAAT :  245

                         *        520         *        540         *        560         *        580         *        600
cen1-CR1 : AATATTATAAAGTTATAAGTATTTTATATTAAGGTTATTATAACCTTATATATATTTAATTTAACTTTATATTAAACTTCTTTTAAAAACTATGCCTTTT :  401
cen1-CR2 : AATACTATAAAGTTATAAGCACTTTATATTAAGGTTATTATAACCTTATATATATTTAATTTAACTTTATACTAAACTCTTTTTAAAGACTATGCCTTTT :  505
cen1-CR3 : AATACTATAAAGTTATAAGCACTTTATACTAAAGTTATTATAACCTTATATATATTTAATTTAACTTTATACTAAACTTCTTTTAAAAACTATACCTTTT :  551
cen1-CR4 : AATGCTATAAAGTTATAAGCACTTTATATTAAGGTTATTATAACCTTATATATATTTAATCTAACTTTATACTAAACTCCTTTTAAAAACTATGCCTTTT :  218
cen1-CR5 : AATACTATAAAGTTATAAGCACTTTATGCTAAGGTTATTATAACCTTATATATATTTAATTTAACTTTATATTAAACTCCTTTTAAAAACTATGCCTTTT :  309
cen2-CR1 : ---------------------------------------------------------------------------------------------------- :    -
cen2-CR2 : AGCGCTATAAAGTTATAAGCACTTTATACTAAAGTTATTATAACCTTATATATATTTAATTTAACTTTATATTAAACTCTTTTTAAAAACTATGCCTTTT :  557
cen2-CR3 : AATGCTATAAAGTTATAAGCACTTTATACTAAGGTTATTATAACCTTATATATATTTAGTCTAGCTTTATACTAAACTCCTTTTAAAAACTATGCCTTTT :  386
cen2-CR4 : ---------------------------------------------------------------------------------------------------- :    -
cen2-CR5 : ---------------------------------------------------------------------------------------------------- :    -
cen3-CR1 : ---------------------------------------------------------------------------------------------------- :    -
cen3-CR2 : ATTATTATAAAGTTATAAGCACTTTATATTAAAGTTATTATAACCTTATATATATTTAATTTAACTTTATATTAAACTTTTTTTAAAAATTATACTTTTT :  383
cen3-CR3 : AATATTATAAAGTTATAAGCACTTTATATTAAGGTTATTATAATTTTATATATATTTAATTTAATTTTATATTAAACTTTTTTTAAAAACTATGCCTTTT :  398
cen3-CR4 : AATATTATAAAATTATAAGCACTTTATATTAAAGTTATTATAACTTTATATATATTTAATTTAACTTTATATTAAACTCTTTTTAAAAACTATGCCTTTT :  332
cen4-CR1 : ---------------------------------------------------------------------------------------------------- :    -
cen4-CR2 : AATATTATAAAGTTATAAGCACTTTATATTAAGATTATTATAACTTTATATATATTTAATTTAACTTTATATTAAACTCCTTTTAAAAACTATACTTTTT :  473
cen4-CR3 : ---------------------------------------------------------------------------------------------------- :    -
cen4-CR4 : AATATTATAAAGTTATAAGCACTTTATATTAAGGTTATTATAACTTTATATATATTTAATTTAATTTTATATTAAACTCTTTTTAAAAACTATACCTTTT :  559
cen4-CR5 : ---------------------------------------------------------------------------------------------------- :    -
cen5-CR1 : AATACTATAAAGTTATAAGCACTTTATACTAAGGTTATTATAACCTTATATATATTTAATCTAACTTTATAATAAACTCCTTTTAAAAACTATGCCTTTT :  369
cen6-CR1 : AATATTATAAAATTATAAGCACTTTATATTAAGGTTATTATAATCTTATATATATTTAATTTAACTTTATATTAAACTCTTTTTAAAAGCTATGCCTTTT :  575
cen7-CR1 : AATATTATAAAGTTATAAGCACTTTATATTAAGGTTATTATAACCTTATATATATTTAATTTAACTTTATATTAAACTTTTTTTAAAAACTATGCCTTTT :  332
cen7-CR2 : AATATTATAAAGTTATAAATACTTTATATTAAGATTATTATAACTTTATATATATTTAATTTAACTTTATATTAAACTTTTTTTAAAAACTATACCTTTT :  376
cen7-CR3 : AATATTATAATATTATAAGTATTTTATATTAAGATTATTATAACCTTATATATATTTAATTTAACTTTATATTAAACTTTTTTTAAGAACTATGCCTTTT :  345
```

```
                   *        620         *        640         *        660         *        680         *        700
cen1-CR1 : TATAAAAATTATATATTATAATTTTAATAAGTTACCTTTCCTTTACTATATTATTTTAATATAATTATTTTACCTTTTATTTA-TTTTTTAGTATTTATT :  500
cen1-CR2 : TATAAGGACTATATACTATAATCTTAATAAGTTACTTTTTTTTTACTATATTATCTTAATATAATTATTCTGCCTTTTATTTA-TTTTCTAGTATTTATC :  604
cen1-CR3 : TATAAGGACTATATACTATAATCTTAATAAATTACCCTTCCTTTACTATATTATCTTAATATAATTATTCTACCTCTTATTTA-TTTTCTAGTATTTATT :  650
cen1-CR4 : TATAAAGACTATATACTATAATCTTAATAAGTTGCCCTTCCTTTACTGTATTATTTTAATATAATTATTCTGCCTTTTATTTATTTTTCTAGCATTTATC :  318
cen1-CR5 : TATAAGGACTATATACTATAATCTTAATAAGTTACTCTTCCTTTACTATATTATCTTAATATAATTATTCTACCTTTTATTTATTTTTTTAGTATTTATC :  409
cen2-CR1 : ---------------------------------------------------------------------------------------------------- :    -
cen2-CR2 : TATAAAAATTATATATTATAATTCTAATAGGTTACCCTTCTTTTACTATATTATCTTAATATAATTATTTTACTTTTTATTTATTTTTTTAATATTTACC :  657
cen2-CR3 : TATAAGAACTATATACTATAATCCTAATAAGTTGCCCTTCCTTTACTATATTATCTTAATATAATTATTCTACCTCCTATTTATTTTTCTAGTATTTACC :  486
cen2-CR4 : ---------------------------------------------------------------------------------------------------- :    -
cen2-CR5 : --------------------------------------------------------------------------------------TTTAAGTATATATA :   14
cen3-CR1 : ---------------------------------------------------------------------------------------------------- :    -
cen3-CR2 : TATAAAAACTATATATTATAATTTTAATAAGTTACTTTTTTTTTACTATATTATTTTAATATAATTATTTTATCTTTTATTTA-TTTTTTAATATTTACT :  482
cen3-CR3 : TATAAAAACTATATATTATAATTTTAATAAGTTACTTTTTTTTTATTATATTATTTTAATATAATTATTTTACTTTTTATTTA-TTTTTTAATATTTACT :  497
cen3-CR4 : TATAAAAACTATATATTATAATTTTAATAAATTACTCTTTTTTTATTATATTATTTTAATATAATTATTTTACTTTTTATTTA-TTTTTTAATATTTACT :  431
cen4-CR1 : ---------------------------------------------------------------------------------------------------- :    -
cen4-CR2 : TATAAAGACTATATATTATAATTTTAATAAATTACCTTTCTTTTACTATATTATTTTAATATAATTATTTTATTTTTTATTTATTTTTTTAATATTTATT :  573
cen4-CR3 : ---------------------------------------------------------------------------------------------------- :    -
cen4-CR4 : TATAAAGACTATATATTATAATTTTAATAAGTTACTTTTTTTTTATTATATTATTTTAATATAATTATTTTATCTTTTATTTATTTTTTTAATATTTATT :  659
cen4-CR5 : ---------------------------------------------------------------------------------------------------- :    -
cen5-CR1 : TATAAAAACTATATATTATAATCTTAATAAATTACCCTTCCTTTACTATATTATTTTAATATAAATATTCTGCCTTTTATTTA--TTTTTAGTATTTATT :  467
cen6-CR1 : TATAAAGGCTATATATTATAATCTTAATAAGTAACCTTTCTTTTATTATATTATCTTAATATAATTATTTTACCTTTTATTTATTTTTTTAGTATTTACT :  675
cen7-CR1 : TATAAAAACTATATATTATAATTTTAATAAGTTACCCTTTTTTTACTATATTATCTTAATATAATTATTTTACTTTTTATTTATTTTTTTAGTATTTACC :  432
cen7-CR2 : TATAAAAATTATATATTATAATTTTAATAAGTTGCCTTTTTTTTATTATATTATTTTAATATAATTATTTTATTTTTTATTTA-TTTTTTAATATTTACT :  475
cen7-CR3 : TATAAAGACTATATATTATAATTTTAATAAATTACCCTTTTTTTATTATATTATTTTAATATAATTATTTTACTTTTTATTTATTTTTTTAATATTTACT :  445

                   *        720         *        740         *        760         *        780         *        800
cen1-CR1 : TTTATTT-TTTTATATTTAATTAAAGCCTAGGCTTATTTAATAGTCTTTATATACTTTAAAGACTTATTATAATATAGCCTATCCTTAA--ATATATCTA :  597
cen1-CR2 : -TAGTTT-TTTTATATTTAGTTAAAGCCTAGGCTTATTTAATAATCTTTATATACTTTAAGAACTTATTATAGTATAGCCTATCCTTAA--ATATATATA :  700
cen1-CR3 : -TAATTC-TTTTATATTTAATTAAAGCCTAGGCCTATTTAATAGTCTTTATATACTTTAAGAACTTATTATAGTATAGCCTATTTTTAA--ATATATATA :  746
cen1-CR4 : -TTATTC-TTTTATATTTAATTAAAGTCTAGGCCTATTTAATAGTCTTTATATGCCTTAAGGACTTATTATAATATAGCCTATTCTTAA--ATATATATA :  414
cen1-CR5 : -TTATTC-TTTTATATTTAATTAAAATCTAGGCCTATTTAATAGTCTTTATATACTTTAAAGACTTATTATAGTATAGCCTATTTTTAA--ATATATATA :  505
cen2-CR1 : ---------------------------------------------------------------------------------------------------- :    -
cen2-CR2 : TTTATTT-TTTTATATTTAGTTAAAGTCTAGGCTTATTTAATAATCCTTATATATTATAAGGACTTATTACTATATAGCCTATTCTTAA--ATATATATA :  754
cen2-CR3 : TTTATTC-TTTTATATTTAATTAAAGTCTAGGCCTATTTAATAGTCCTTATATACTATAAGGACTTATTACTATATAGCCTATCCTTAAATATATATATA :  585
cen2-CR4 : ---------------------------------------------------------------------------------------------------- :    -
cen2-CR5 : TTTATAT--------------------------------------------------------------------------------------------- :   21
cen3-CR1 : ---------------------------------------------------------------------------------------------------- :    -
cen3-CR2 : TTTATTT-TTTTATATTTAATTAAAGCTTAAGCTTATTTAATAGTCTTTATATATTATAAAAACTTATTACTATATAGCCTATCCTTAAATATATATATA :  581
cen3-CR3 : TTTATTT-TTTTATATTTAATTAAAGCCTAGGCCTATTTAATAATTCTTATATACTTTAAGGATTTATTATAATATAGCCTGTTTTTAA--ATATATATA :  594
cen3-CR4 : TTTATTT-TTTTATATTTAGTTAAAGCCTAGGCCTATTTAATAGTTTTTATATACTATAAGGACTTATTATTATATAGCCTATTTTTAAATATATATATA :  530
cen4-CR1 : ---------------------------------------------------------------------------------------------------- :    -
cen4-CR2 : TTTATTT-TTTTATATTTAGTTAAAGCCTAGGCTTATTTAATAGTCTTTATATACCTTAAAGACTTATTATAGTATAGCCTATTTTTAA--ATATATATA :  670
cen4-CR3 : ---------------------------------------------------------------------------------------------------- :    -
cen4-CR4 : TTTATTT-TTTTATATTTAGTTAAAGCCTAGGCTTATTTAATAGTTTTTATATACTTTAAGGACTTATTATAATATAGCCTATTTTTAA--ATATATATA :  756
cen4-CR5 : ---------------------------------------------------------------------------------------------------- :    -
cen5-CR1 : TTTATTT-TTTTATATTTAGTTAAGGCCTAGGCTTATTTAATAGTCTTTATATACCTTAAAGACTTATTATAGTATAGCCTATCTTTAA--ATATATATA :  564
cen6-CR1 : TTTATTT-TTTTATATTTAATTAAAACCTAGGCTTATTTAATAATTTTTATATATTATAAAAACTTATTACTATATAGCCTATTTTTAAATATATATATA :  774
cen7-CR1 : TTTATTC-TTTTATATTTAATTAAAGCTTAGGCTTATTTAATAGTCTTTATATACTATAAGGACTTATTATTATATAGCCTATTTTTAAATATATATATA :  531
cen7-CR2 : TTTATTT-TTTTATATTTAATTAAAGCCTAGGCTTATTTAATAGTTTTTATATATTATAAAGACTTATTATTATATAGCCTATCCTTAA--ATATATATA :  572
cen7-CR3 : TTTATTTTTTTTATATTTTATAAAAGCCTAGGCTTATTTAATAGTCTTTATATACTATAAGGATTTATTATTATATAGCCTATTCTTAA--ATATATATA :  543
```

```
                       *       820         *       840         *       860         *       880         *       900
cen1-CR1 : TTTATTTTTTACTTAATTAGTTTTTAATTATAATACTTTTTTATTATTTTTATATTTTTTTATTATAATACTTTTATAAATTTATTATTTAATAGCTATT :  697
cen1-CR2 : CTTATTTTCTACTTAGTTAGTTTTTAATTATAATACTTCCTTACTATTTCTATACTTTTTTATTATAATGCCTTTATAAACTTATTACTTAAAGGCTATT :  800
cen1-CR3 : CTTATTTTCTACTTAGTTAGTTTTTAATTATAATACTTCCTTATTATTTTTATACTTTTTTATTATAATACCTTTATAAACTTATTACTTAAAGGCTATT :  846
cen1-CR4 : CTTATTTTCTACCTAGTTAGTCTTTAATTATAATACTTCCTTACTATTTTTATACTTTTTTATTATAATACCTTTATAAACTTATTACTTAAAGGCTATT :  514
cen1-CR5 : CTTATTTTCTACCTAGTTAGTCTTTAATTATAATACTTTCTTATTATTTCTATGCTTTTTTATTATAATGCCTTTATAAACTTATTACTTAAAGGCTATT :  605
cen2-CR1 : ---------------------------------------------------------------------------------------------------- :    -
cen2-CR2 : CTTATTTTCTACCTAGTTAGTCTTTAATTATAATACTTTCTTATTATTTTTATATTCTTTTATTATAATACCTTTATAAATTTATTACTTAGCAGCTATT :  854
cen2-CR3 : CTTATTTTCTACCTAGTTAGTCTTTAATTATAATACTTCCTTACTATTTCTGCATTCTTTTATTATAATACCTTTATAAATTTATTACTTAGCAGCTATT :  685
cen2-CR4 : ---------------------------------------------------------------------------------------------------- :    -
cen2-CR5 : ---------------------------------------------------------------------------------------------------- :    -
cen3-CR1 : ---------------------------------------------------------------------------------------------------- :    -
cen3-CR2 : CTTATTTTTTATTTAATTAGTTTTTAATTATAATACTTTTTTATTATTTTTATATTTTTTTATTATAATACTTTTATAAACTTATTATTTAATAGCTAAT :  681
cen3-CR3 : CTTATTTTTTATTTAATTAGTTTTTAATTATAATACTTTTTTACTTTTTTTATACTTTTTTATTATAATACTTTTATAAACTTATTATTTAATAGCTATT :  694
cen3-CR4 : CTTATTTTTTACTTAATTAATTTTTAAATATAATACTTTTTTATTATTTTTATATTTTTTTATTATAATGCCTTTATAAACTTATTATTTAATAGCTATT :  630
cen4-CR1 : ---------------------------------------------------------------------------------------------------- :    -
cen4-CR2 : CTTATTTTTTACTTAGTTAATCCTTAGTTATAATACTTTTTTACTATTTTTATACTTTTTTATTATAATGCCTTTATAAACTTATTATTTAAAGGCTATT :  770
cen4-CR3 : ---------------------------------------------------------------------------------------------------- :    -
cen4-CR4 : CTTATTTTCTACTTAATTAGTTTTTAATTATAATACTTTTTTACTATTTTTATACTTTTTTATTATAATACCTTTATAAACTTATTATTTAAAGGCTATT :  856
cen4-CR5 : ---------------------------------------------------------------------------------------------------- :    -
cen5-CR1 : CTTATTTTCTACTTAGTTAGTTTTTAATTATAATACTTTTTTACTATTTTTATACTTTTTTATTATAATGCCTTTATAAACTTATTACTTAGCAGCTATT :  664
cen6-CR1 : CTTATTTTTTACTTAATTAGTCTTTAATTATAATACTTT-TTACTATTTTTATATTTTTTTATTATAATACCTTTATAAATTTATTACTTAGTAGCTATT :  873
cen7-CR1 : CTTATTTTTTATTTAGTTAATTTTTAATTATAATACTTTTTTATTATTTTTATATTCTTTTATTATAATATTTTTATAAACTTATTATTTAGTAGCTATT :  631
cen7-CR2 : TTTATTTTTTACTTAATTAATTTTTAATTATAATACTTTTTTACTATTTTTATATTTCTTTATTATAATATTTTTATAAACTTATTACTTAGCAGTTATT :  672
cen7-CR3 : CTTATTTTTTATTTAATTAGTTTTTAATTATAATACTTTTTTATTTTTTTTATAGTTTTTTATTATAATACCTTTATAAATTTATTACTTAATAATTATT :  643

                       *       920         *       940         *       960         *       980         *      1000
cen1-CR1 : ATTTTACTTAAAAATCCTTTTATATATTACTTATAAAAATACTTTTAAAAAATACCTTAGCTTATACTAACCTTTTTAAAAAACTTTTTTAATAATATAT :  797
cen1-CR2 : ATTTTACTTAAGAATTCTCCTATATATTACTTATAAAAATACCCTTAAAAAATACCTTAGCTTATATTAATCTTTTTAAAAAACTCTTTTAATAATATAT :  900
cen1-CR3 : ATTCTACTTAAAAATTCTCTTATATATTACTTATAAAAACGCCCTTAAAAAATGCCTTAGCTTATACTAATCTTTTTAAAAAACTCTTTTAATAATATAT :  946
cen1-CR4 : ATTTTACTTAAGAATTCTCCTATATATTACCTATAAAGACGCCCTTAAAAAATACCTTAGCCTATGCTAATCTCTTTAAAAAACTTTCCTAATAATATAT :  614
cen1-CR5 : ATTTTATTTAAGAATTCTTCTATATATTACTTATAAAAATGCCTTTAAAAAATGCCTTAGCTTACACTAATCTCTTTAAAGGACTCTCTTAATAATATAT :  705
cen2-CR1 : ---------------------------------------------------------------------------------------------------- :    -
cen2-CR2 : ATTTTACTTAAGAATTCTCCTATCTATTACTTAAAAGGATGCCTTTAAAAAACACCTTAGCTTATACTAACCTTTTTAAAAAACTCTTTTAATAATATAT :  954
cen2-CR3 : ATTTTACTTAAGAATTCTCCTATCTATTACCTAAAAGAATGCCTTTAAAAAACGCCTTAGCTTATACTAACCTCCTTAAAAGACTTTTTTAATAATATAT :  785
cen2-CR4 : ---------------------------------------------------------------------------------------------------- :    -
cen2-CR5 : ---------------------------------------------------------------------------------------------------- :    -
cen3-CR1 : ---------------------------------------------------------------------------------------------------- :    -
cen3-CR2 : CTTTTACTTAAGAATTCTCCTATATATTACTTATAAAAATACTTTTAAAAAATACCTTAGCCTATATTAACCT-TTAAAAAAACTCTTTTAATAATATAT :  780
cen3-CR3 : ATTTTACTTAAGAATTCTTTTATATATTACTTATAAAAATATTTATAAAAAATACCTTAGCTTATATTAATCTTTTTAAAAAACTTTTTTAATAATATAT :  794
cen3-CR4 : ATTTTACTTAAGAATTCTTTTATATATTACTTATAAAAATACTTTTAAAAAATGCCTTAGCTTATATTAACCTTTTAAAAAAATTCTTTTAATAATATAT :  730
cen4-CR1 : ---------------------------------------------------------------------------------------------------- :    -
cen4-CR2 : ATTTTACTTAAAAATTCTCTTATATATTACTTATAAAAATACTTTTAAAAAATGCCTTAGCTTATATTAATCTTTTTAAAAAACTCTCTTAATAATATAT :  870
cen4-CR3 : ---------------------------------------------------------------------------------------------------- :    -
cen4-CR4 : ATTTTACTTAAAAATTCTTCTATATATTATTTATAAAAATGCCTTTAAAAAACGCCTTAGCTTATACTAATCTCCTTAAAAAACTCTTTTAATAATATAT :  956
cen4-CR5 : ---------------------------------------------------------------------------------------------------- :    -
cen5-CR1 : ATTCTATTTAAGAATTCTCTTATATATTACTTATAAAAATGCCTTTAAAAAACGCCTTAGCTTATACTAACCTCCTTAAAAAACTCTTTTAATAATATAT :  764
cen6-CR1 : ATTTTACTTAAGAATTCTTTTATATATTACTTATAAAAATGCCTTTAAAAAATCCCTTAGCTTATATTAACCTTTTTAAAAGATTCTTTTAATAATATAT :  973
cen7-CR1 : ATTTTACTTAAGAATTTTTTTATATATTACTTATAAAAACACCTTTAAAAAATACTTTAGCTTATATTAACCTTCTTAAAGGACTTTTTTAATAATATAT :  731
cen7-CR2 : ATTTTATTTAAAAATTCTCTTATATATTACTTATAAAAACACCTTTAAAAAATGCCTTAACTTATACTAACCTTTTAAAAAAACTTTTTTAATAATATAT :  772
cen7-CR3 : ATTTTACTTAAAAATTCTCTTATATATTACTTATAAAAATACTTATAAAAAATGCTTTAACTTATACTAACTTTTTTAAAAGACTTTTTTAATAATATAT :  743
```

```
                     *      1020         *      1040         *      1060         *      1080         *      1100
cen1-CR1 : TAAATAATAAAAAA-GTAAAGAAAAAAGTAATTATATTAAAAAATAATATAGCCTTAAACCTTTTTAAAACCTTATTATAATATACTTATTATATTTTTA :  896
cen1-CR2 : TAAATAATAAAGAA-GTAAAGGAGGAAGTAGTTATATTAAGAGATAGTATAACCTTAAACCTTTTTAAAACCTTATTATAATATGCCTATTATATCTTTA :  999
cen1-CR3 : TAAATAATAAGAAA-GTAAAAGAGAAAGTAATTATACTAAAAGATAGTATAACCTTAAACCTTTTTAAAACCTTATTATAATATACTTATTATATCTTTA : 1045
cen1-CR4 : TAAATAATAAGGAA-GTGAAGGAGGAAGTAGTTATACTAGGAGATAGTATAGCCTTAAACCTTTTTAAAACCTTATTATAGTATGCCTATTATATCCTTA :  713
cen1-CR5 : TAAATAATAAGAAA-GTAAAGAAGGAAGTGGTTATACTAAAAGATAGTATAGCCTTAAACCTTTTTAAAACCTTATTATAATATACTTATTATATTTTTA :  804
cen2-CR1 : ---------------------------------------------------------------------------------------------------- :    -
cen2-CR2 : TAAATAATAAAAAA-GTAAAAGAAGAAGTAGTTATATTAAAAAATAGTATAGCCTTAAACCTTTTTAAAACCTTATTATAGTATACTTATTATATTTTTA : 1053
cen2-CR3 : TAAATAATAAGGAA-ATAAAGGAAAAAGTGGTTATACTAAGAGATAGTATAGCCTTAAATCTCCTTAAAACCTTATTATAGTATGCCTATTATATCCTTA :  884
cen2-CR4 : ---------------------------------------------------------------------------------------------------- :    -
cen2-CR5 : ---------------------------------------------------------------------------------------------------- :    -
cen3-CR1 : ---------------------------------------------------------------------------------------------------- :    -
cen3-CR2 : TAAATAATAAAAAA-GTAAAGAAAAAAGTAATTATAGTAAAAAAGAGTATAGCCTTAAACCTTTTTAAAATCTTATTATAGTATATTTATTATATTTTTA :  879
cen3-CR3 : TAAATAATAAAAAA-GTAAAA-AAAAAGTAATTATATTAAAAAATAATATAGCCTTAAACCTTTTTAAAACCTTATTATAATATACTTATTATATTTTTA :  892
cen3-CR4 : TAAATAATAAAAAA-ATAAAAAAAAAAGTAATTATATTAAAAAATAATATAGCCTTAAACCTTTTTAAAACCTTATTATAATATACTTACTATATTTTTA :  829
cen4-CR1 : ---------------------------------------------------------------------------------------------------- :    -
cen4-CR2 : TAAATAATAAAAAAAGTAAAAAAAAAAGTAATTATATTAAAAAATAATATAGCCTTAAACCTTTTTAAAATCTTATTATAATATATTTATTATATTTTTA :  970
cen4-CR3 : ---------------------------------------------------------------------------------------------------- :    -
cen4-CR4 : TAAATAATAAAAAA-GTAAAAGAGGAAGTAATTATATTAAAAAATAGTATAGCCTTAAACCTTTTTAAAACCTTATTATAATATACCTATTATATCTTTA : 1055
cen4-CR5 : ---------------------------------------------------------------------------------------------------- :    -
cen5-CR1 : TAAATAATAAAAAA-GTAAAGGAAGAAGTAGTTATATTAAAAGATAATATAGCCTTAAACCTTTTTAAAACCTTATTATAGTATACTTATTATATCTTTA :  863
cen6-CR1 : TAAATAATAAAAAA-GTAAAAAAAGAAGTAGTTATATTAAAAGATAATATAGCCTTAAACCTTTTTAAAACCTTATTATAATATATTTATTATATTTTTA : 1072
cen7-CR1 : TAAATAATAAAAAA-GTAAAGAAAAAAGTAATTATATTAAAAAATAATATAGTTTTAAACCTTTTTAAAATCTTATTATAATATACTTATTATATTCTTA :  830
cen7-CR2 : TAAATAATAAAAAA-ATAAAAGAAAAAGTAATTATATTAAAAAATAGTATAGCCTTAAACCTTTTTAAAACCTTATTATAATATACTTATTATATCCTTA :  871
cen7-CR3 : TAAAAAATAAAAAA-GTAAAAGAAAAAGTAATTATATTAAAAAATAGTATAGCTTTAAACCTTTTTAAAACCTTATTATAATATACTTATTATATTTTTA :  842

                     *      1120         *      1140         *      1160         *      1180         *      1200
cen1-CR1 : TATTTATTATAAAATTATATTAATATTATAATAAATTAATATTATATAAGTATTACTTTTAAAGTTTAATATATTAAAAAGTAAGTAAGCCTTTTATCTT :  996
cen1-CR2 : TATTTATTATAAAGTTATATTAATATTATAATAGACTAGTACTATATAAGTATTACTTTTAAGGTTTAGTATATTAAAAAGTAAGTAAGCCTTTTATCCT : 1099
cen1-CR3 : TATTTATTATAAAGTTATATTAATATTATAGTAGACTAGTGTTATATAAGTATTACTTTTAGGGTTTAGTATATTAAAAAGTAGGTAAGCTTTTTATCCC : 1145
cen1-CR4 : TATTTATTATAAAGTTATATTAATATTATAGCAGACTAGTGCTATATAAGTATTACTTTTAAGGTTTAGTATATTAAAAAGTAAGTAAGCCTTTTATCTT :  813
cen1-CR5 : TATTTATTATAAAGTTATACTAATATTATAATAAATTAATGCTATATAAGCATTACTTTTAAGGTTTAGTATATTAAAAAGTAGGTAAGCCTTTTATTTT :  904
cen2-CR1 : ---------------------------------------------------------------------------------------------------- :    -
cen2-CR2 : TATTTATTATAAAGTTATATTAATATTATAATAAACTAATGCTATATAAGTATTACTTTTAAGGTTTAGTATATTAAAAAGTAAGTAAGCTTTTTATTTT : 1153
cen2-CR3 : TATTTATTATAAAGTTATATTAATATTATAGCAGACTAATGCTATATAAGTATTACTTTTAAGGTTTAGTATATTAAAAAGTAAGTAAGCCTCTTATCCT :  984
cen2-CR4 : ---------------------------------------------------------------------------------------------------- :    -
cen2-CR5 : ---------------------------------------------------------------------------------------------------- :    -
cen3-CR1 : ---------------------------------------------------------------------------------------------------- :    -
cen3-CR2 : TATTTATTATAAAGTTATATTAATATTATAATAAATTAATATTATATAAGTATTATTTTTAAGGTTTAGTATATTAAAAAGTAAATAAGCCTTTTATTTT :  979
cen3-CR3 : TATTTATTATAAAGTTATATTAATATTATAATAAATTAATATTATATAAGTATTACTCTTAAGGTTTAATATATTAAAAAGTAAGTAAGCTTTTTATTTT :  992
cen3-CR4 : TATTTATTATAAAATTATATTAATATTATAATAAACTAGTGCTATATAAGTATTATTTTTAAAATTTAATATATTAAAAAATAAGTAAGCTTTTTATTTT :  929
cen4-CR1 : ---------------------------------------------------------------------------------------------------- :    -
cen4-CR2 : TATTTATTATAAAGTTATATTAATATTATAATAAATTAATATTATATAAGTATTACTTTTAAGGTTTAGTATATTAAAAAGTAAGTAAGCTTTTTATTTT : 1070
cen4-CR3 : ---------------------------------------------------------------------------------------------------- :    -
cen4-CR4 : TATTTATTATAAAGTTATATTAATATTATAGCAGACTAATACTATATAAGTATTACTTTTAAGGTTTAGTATATTAAAAAGTAAGTAAGCCTTTTATCTT : 1155
cen4-CR5 : ---------------------------------------------------------------------------------------------------- :    -
cen5-CR1 : TATTTATTATAAAGTTATATTAATATTATAGCAGACTAGTGTTATATAAGTATTACTTTTAGGGTTTAGTATATTAAAAAGTAAATAAGCCTCTTATCTT :  963
cen6-CR1 : TATTTATTATAAAGTTATATTAATATTATAGTAAACTAGTATTATATAAGTATTACTTTTAAGGTTTAATATATTAAAAAGTAAGTAAGCCTTTTATTTT : 1172
cen7-CR1 : TATTTATTATAAAGTTATATTAATATTATAGCAGACTAATACTATATAAGTATTACTTTTAAGGTTTAGTATATTAAAAAGTAAGTAAGCTTTTTATTTT :  930
cen7-CR2 : TATTTATTATAAAGTTATATTAATATTATAATAAAATAATATTATATAAGTATTATTTTTAAGGTTTAGTATATTAAAAAGTAAGTAAGCCTTTTATTTT :  971
cen7-CR3 : TATTTATGATAAAATTATATTAATATTATAATAAATTAATATTATATAAGTATTACTTTTAAGGTTTAGTATATTAAAAAGTAAGTAAGCTTTTTATTTT :  942
```

```
                       *        1220         *        1240         *        1260         *        1280         *        1300
cen1-CR1 : TTATTAA-AGGCTTTTTTTACTTTTTTAAAATATTTTTATTATACTTAAAATTAAGGTTAAAGTAGTTATATTTTATTATTTAAATATATATATATAAGGCT : 1095
cen1-CR2 : TTATTAAGAGGCTTTTACTGCTTTTTTAAAATATTTCTATTATACTTAAAATTAAGGTTAAGGTAGTTATATTTTATTACTTAAGTATATATATATAAGGCT : 1199
cen1-CR3 : TTATTAAAAGGCTTTTACTGCTTTTTTAAAATATTTCTATTATACCTAAAATTAAAGTTAAGGTAGTTATATCTTATTACTTAAGTATATATATATAAGGCT : 1245
cen1-CR4 : TTATTAAAAGGCTTTTACTGCTTTCTTAAAATATTTTTATTATGCCTAAAATTAAGGTTAAGGTAGTTATATCTTATTACTTAAGTATATATATATAAGGCT :  913
cen1-CR5 : TTATTAAAAGGCTTTTATTACTTTTTTAAAATATTTTTATTATACTTAAAATTAAAGTTAAGGTAGTTATATCTTATTACTTAAGTATATATATATAAGGCT : 1004
cen2-CR1 : ---------------------------------------------------------------------------------------------------- :    -
cen2-CR2 : TTATTAAAAGGCTTTTTCTACTTTTTTAAAATACTTTTATTATGCTTAAAATTAAGGTTAAGGTAGTTATATTTTATTACCTAAATATATATATATAAGGCT : 1253
cen2-CR3 : TTATTAAAAGGCTCCTTCTACTTCCTTAAAATGCTTCTATTATACTTAAAATTAAGGTTAAGGTAGTTATATCTTATTACTTAAATATATATATATAAGGCT : 1084
cen2-CR4 : ---------------------------------------------------------------------------------------------------- :    -
cen2-CR5 : ---------------------------------------------------------------------------------------------------- :    -
cen3-CR1 : ---------------------------------------------------------------------------------------------------- :    -
cen3-CR2 : TTATTAAAAAGCTTTTATTACTTTTTTAAAATATTTTTATTATATTTAAAATTAAAGTTAAAGTAATTATATTTTATTACTTAAATATATATATATAAGGCT : 1079
cen3-CR3 : ATATTAAAAGGCTTTTATTACTTTTTTAAAATATTTTTATTATATTTAAAATTAAAGTTAAAGTAATTATATTTTATTACTTAAATATATATATATAAGGCT : 1092
cen3-CR4 : TTATTAAAAGGCTTTTATTACTTTTTTAAAATATTTTTATTATATTTAAAATTAAGGTTAAAATAGTTATATTTTATTACTTAAATATATATATATAAGGCT : 1029
cen4-CR1 : ---------------------------------------------------------------------------------------------------- :    -
cen4-CR2 : TTATTAAAAGGCTTTTACTACTTTTTTAAAATATTTTTATTATACTTAAAATTAAAATTAAAGTAGTTATATTTTATTACTTAAGTATATATATATAAGGCT : 1170
cen4-CR3 : ---------------------------------------------------------------------------------------------------- :    -
cen4-CR4 : TTATTAAAAGGCTTTTATTACTTTTTTAAAATATTTTTATTATATTTAAAATTAAAGTTAAGGTAATTATATTTTATTACTTAAGTATATATATATAAGGCT : 1255
cen4-CR5 : ---------------------------------------------------------------------------------------------------- :    -
cen5-CR1 : TTATTAAGAGGCTTTTTCTGCTTTTTTAAAATATTTTTATTATACTTAAAATTAAGGTTAAGGTAGTTATATCTTATTACCTAAATATATATATATAAAGCT : 1063
cen6-CR1 : TTATTAAAAGGCTTTTTTACTTTTTTAAAATATTTTTATTATACTTAAAATTAAGGTTAAGGTAGTTATATCTTATTATTTAAATATATATATATAAGGCT : 1272
cen7-CR1 : TTATTAAAAAGCTTTTTTTACTTTTTTAAAATATTTTTATTATACTTAAAATTAAAGTTAAAGTAGTTATATCTTATTATTTAAATATATATATATAAGGCT : 1030
cen7-CR2 : TTATTAAAAGGCTTTTACTGCTTTTTTAAAATATTTTTATTATATTTAAAATTAAAGTTAAAGTAATTATATTTTATTATTTAAATATATATATATAAGACT : 1071
cen7-CR3 : TTATTAAAAGGCTTTTATTATTTTTTTAAAATATTTTTATTATACTTAAAATTAAGGTTAAGGTAGTTATATTTTATTACTTAAATATATATATATAAGGCT : 1042

                       *        1320         *        1340         *        1360         *        1380         *        1400
cen1-CR1 : ATATATATATTATAATCTGCCTTATTAATTAATTTTTAAAATATAGTAAAACTTA--TTTTCTTTTTTTACTTTTAGCTTAAATATTAAACTTATATTTC : 1193
cen1-CR2 : ATATATATATTATAATCTGCTTTATTAGCTAATTTATAAAATATAGTATAACTTA--TTTTTTTCTTTTACTTTTAATTTAAATACTAAACTTATATTTC : 1297
cen1-CR3 : ATATATATATTATAGTTTACCTTATTAGCTAATTTATAAAATATAATATAACTTA--TCTTCTTCTTTTACTTTTAGCTTAAATATTAAACTTATATTTT : 1343
cen1-CR4 : ATATGCATATTATAGTCTGCCTTATTAGCTAATTTATAAAATATAGTATAACTTA--TTTTCTTTTTTTACTCTTAGCCTAAATACTAAACTTATATTTC : 1011
cen1-CR5 : ATATATATATTATAGTCTGCCTTATTAGCTAATTTATAAAATATAATATAACTTA--TCTTCTTTTTTTACTCTTAGCTTAAATATTAAACTTATATTTC : 1102
cen2-CR1 : -----TATATTATAAACTGCTTTATTAGCTAATTTATAAAATATAATAAAACTTA---TTTTTTTTTTTTACTTTTAGCTAAAATACTAAATTTATATCTT :   92
cen2-CR2 : ATATATATATTATAATTTATTATATTAGCTAATCTTTAAAATATAATAAGACTTA--TTTTTTTTTTTTACTTTTAGCTTAAATATTAAACTTATATTTT : 1351
cen2-CR3 : GTATATATATTATAATCTACTATATTAGCTAATCCTTAAAATATAGTAAGACTTA--TTTTTTTCTTTTACTCTTAGCCTAAATATTAAACTTATATTTT : 1182
cen2-CR4 : ---------------------------------------------------------------------------------------------------- :    -
cen2-CR5 : ---------------------------------------------------------------------------------------------------- :    -
cen3-CR1 : ---------------------------------------------------------------------------------------------------- :    -
cen3-CR2 : ATATATATATTATAGTCTACTTTATTAATTAATTTTTAAAATATAATAAAACCTA----TTTTTTTTTTTACTTTTAATTTAAATACTAAACTTATATTTT : 1175
cen3-CR3 : ATATATATATTATAATCTGCTTTATTAATTAATTTATAAAATATAATAAAACTTA---TTTTTTTTTTTTACTTTTAGCTTAAATATTAAACTTATATTTA : 1189
cen3-CR4 : ATATATATATTATAGTTTACTTTATTAATTAATTTTTAAAATATAATAAAACTTATTTTTTTTTTTTTTACTTTTAGCTTAAATATTAAACTTATATTTT : 1129
cen4-CR1 : ---------------------------------------------------------------------------------------------------- :    -
cen4-CR2 : ATATATATATTATAGTCTGCTTTATTAATTAATTTATAAAATATAATATAACTTA--TTTTTTTCTTTTACTTTTAGCTTAAATACTAAACTTATATTTT : 1268
cen4-CR3 : ---------------------------------------------------------------------------------------------------- :    -
cen4-CR4 : ATATATATATTATAATTTATTTTATTAGCTAATTTATAAAATATAATATAACTTA--TTTTCTTTTTTTACTTTTAGCTTAAATATTAAACTTATATTTT : 1353
cen4-CR5 : ---------------------------------------------------------------------------------------------------- :    -
cen5-CR1 : ATATATATATTATAGTCTGCTTTATTAGCTAATCCTTAAAATATAATAAGACTTA--TCTTTTTCTTTTACTCTTAGCTTAAATATTAAACTTGCATTTT : 1161
cen6-CR1 : ATATATATATTATAATTTACTTTATTAGCTAATTTTTAAAATATAGTAAGATTTA-----TTTTTTTTTTACTTTTAGCCTAAATACTAAACTTATATTTT : 1367
cen7-CR1 : ATATATATATTATAGTTTGCTTTATTAACTAATTTTTAAAATATAATAAGACTTA---TTTTTTTTTTTTACTTTTAATTTAAATACTAAACTTATATTTT : 1127
cen7-CR2 : ATATATATATTATAATTTACTTTATTAATTAATTTTTAAAATATAATAAAACTTA----TTTTTTTTTTTACTTTTAATTTAAATATTAAACTTATATTTT : 1167
cen7-CR3 : ATATATATATTATAATTTACTTTATTAACTAATTTATAAAATATAATAAA--------TTTTTTTTTTTTACTTTTAGCTTAAATATTAAACTTATATTTT : 1134
```

```
                       *      1420         *      1440         *      1460         *      1480         *      1500
cen1-CR1 : TAAATATATATTAAAATATTATAAATATTTTATTAAAAATATATATAGTTTTTTTATAAAAACTTTTTATATTATTTTTAAAATAATTTTAACTTTTTTA : 1293
cen1-CR2 : TAAGTATATATTAAAGTATTATAAGTGCCTTATTAAAAATATATATAGTCTTTTTATAAAAACTTTTTATATTATTTTTAAAATAATTTTAATCCTTTTA : 1397
cen1-CR3 : TAAGTATATATTAAAATACTATAGGTGCTTTATTAAAAATATATATAGTCTTTTTATAAAAACTTTTTATATTATTTTTAAAATAATTTTAATCCTTTTA : 1443
cen1-CR4 : TAGGCATATATTAAAGTATTATAAGTACCTTATTAAAAATATATATAGTCTTTTTATAAGAACTCTTTATATTATTTTTAAGATAATTCTAATCCCTTTA : 1111
cen1-CR5 : TAAATATATATTAAAGTACTGTAAATGCCTTATTAAAAATATATATAGTCTTTTTATAAAAACTCCTTATATTATTTTTAAAATAATTCTAATCCTTTTA : 1202
cen2-CR1 : TAAGTATATATTAAAGTATTATAAATACTTTATTAAAAATATATATAGTCTTTTTATAAAAACTTTTTATATTATTTTTAAAATAACTTTAATTTTTTTA :  192
cen2-CR2 : TAGGTATATATTAAAGCACTGCAGGTGCTTTATTAAAAATATATATAGTCTTTTTATAAAAACTCTTTATATTATTTTTAAGATAATTCTAATCCTTTTA : 1451
cen2-CR3 : TAAGCATATATTAAAGCACTGCAAATGCCTTATTAAAAATATATATAGTCTTTTTATAAAAACTCTTTATATTATTTTTAAAATAATTTTAATCCCTTTA : 1282
cen2-CR4 : ---------------------------------------------------------------------------------------------------- :    -
cen2-CR5 : ---------------------------------------------------------------------------------------------------- :    -
cen3-CR1 : ---------------------------------------------------------------------------------------------------- :    -
cen3-CR2 : TAAATATATATTAAAGTACTATAAGTGCTTTATTAAAAATATATATAGTCTTTTTATAAAAACTTTTTTTACTATTTTTAAAATAATTTTAATTCTTTTA : 1275
cen3-CR3 : TAAATATATATTAAAGTATTATAAGTGCTTTATTAAAAATATATATAGTCTTTTTATAAAAATTCTTTATATTATTTTTAAAATAATTCTAATTCTTTTA : 1289
cen3-CR4 : TAAATATATATTAAAGTATTATAAATACTTTATTAAAAATATATATAGTTTTTTTATAAAAACTTTTTATATTATTTTTAAAATAATTTTAATTCTTTTA : 1229
cen4-CR1 : ---------------------------------------------------------------------------------------------------- :    -
cen4-CR2 : TAAGTATATATTAAAGTATTATAAATACCTTATTAAAAATATATATAGTCTTTTTATAAAAACTTTTTATATTATTTTTAAAATAATTCTAATCTTTTTA : 1368
cen4-CR3 : ---------------------------------------------------------------------------------------------------- :    -
cen4-CR4 : TAAGTATATATTAAAGTATTATAAGTGCTTTATTAAAAATATATATAATTTTTTTTATAAAAACTTTTTATATTATTTTTAAAATAATTTTAATTTTTTTA : 1453
cen4-CR5 : ---------------------------------------------------------------------------------------------------- :    -
cen5-CR1 : TAAATATATATTAAAGTATTATAAATGCCTTATTAAAAATATATATAGTTTTTTTATAAAAACTTTTTATATTATTTTTAAAATAATTTTAATTCTTTTA : 1261
cen6-CR1 : TAAGTATATATTAAAGTACTGCAGGTGCCTTATTAAAAATATATATAGTTTTTTTATAAAAACTTTTTATATTATTTTTAAAATAATTTTAATTTTTTTA : 1467
cen7-CR1 : TAAGTATATATTAAAGTATTATAAGTACTTAATTAAAAATATATATAGTTTTTTTATAAAAACTCTTTATATTATTTTTAAAATAATTTTAATCTTTTTA : 1227
cen7-CR2 : TAAGTATATATTAAAGTATTATAAATATTTTATTAAAAATATATATAGTCTTTTTATAAAAACTTTTTATATTATTTTTAAAATAATTTTAATCCTTTTA : 1267
cen7-CR3 : TAAATATATATTAAAGTATTATAAATGCCTTATTAAAAATATATATAGTCTTTTTATAAAAACTTTTTATATTATTTTTAAAATAATTTTAATTCTTTTA : 1234

                       *      1520         *      1540         *      1560         *      1580         *      1600
cen1-CR1 : TAAAAATAC------TTAAAAAAAAAGTAAAAAAATATTTTTATTTTTTTATTACTTTTAAAAAGCGCTTTTTTATATTTTTTTTATAATAAATATAATT : 1387
cen1-CR2 : TAAATATAT------TTAAGGAAGAGGTAAAAAAGCATTTTTATCCTTTTATTACTTTTAAAAGGCACTCCTTTATATTTTCTTTATAATAAATACAGTT : 1491
cen1-CR3 : TAAATATAT------TTAAAAAAGAGGTAAAAAAGTATTCTTATCCCTTTATTACTTTTAAAAGGCACTCCCTTATATTTCCTTTATAATAAATATAATT : 1537
cen1-CR4 : TAAATATAC------TTAAGGAAGAGGTAAAGAAGTATTCTTATCCCTTTATTACTTTTAAAAGGCGCTCCTTTATATTTTCTTTATAATAAATATAGTT : 1205
cen1-CR5 : TAAATATAC------TTAAGGAAGAGGTAAAGAAGTATTCTTATCCTTTTATTACTTTTAAAAGGCGCTTTTTTATATTTCCTTTATAGTAAATACAGTT : 1296
cen2-CR1 : TAAATATACTTATAATTAAAAAAAAAGTAAAAAAGTATTTTTATTTTTTTATTACTTTTAAAAAGTGCTTTTTTATATTTCCTTTATAATAAATATAATT :  292
cen2-CR2 : TAAAAATAC------TTAAAGAAAAGGTAAAAGAGTATTTTTATTTTTTTATTACTTTTAAAAAGCATTTTTTTATATTTTTTTTATAATAAATATAGTT : 1545
cen2-CR3 : TAAAAACAC------TTAAGAAAGAGGTAAAGGAGTATTTTTATCCCTTTATTACTTTTAAGGGGCACTTTTCTATATTCCCTTTATAATAAATATAATT : 1376
cen2-CR4 : ---------------------------------------------------------------------------------------------------- :    -
cen2-CR5 : ---------------------------------------------------------------------------------------------------- :    -
cen3-CR1 : ---------------------------------------------------------------------------------------------------- :    -
cen3-CR2 : TAAATATATT-----TAAAAAAAAAAGTAAAAAAGTATTTTTATTTTTTTATTACTTTTAAAAGGCGCTTTTTTATATTTCCTTTATAATAAATATAATT : 1370
cen3-CR3 : TAAATATAT------TTAAAAAAAAAGTAAAAAAGTATTTTTATTTTTTTATTACTTTTAAAAAGTATTTTTTTATATTTTTTTTTAGTAAATATAATT : 1383
cen3-CR4 : TAAATATAC-------TTAAAAAAAAGTAAAAAAGTATTTTTATTTTTTTATTACTTTTAAAAGGCATTTTTTTATATTTCCTTTATAATAAATATAATT : 1322
cen4-CR1 : ---------------------------------------------------------------------------------------------------- :    -
cen4-CR2 : TAAATATAC------TTAAAAAAAAGGTAAAAAAGTATTTTTATTTTTTTATTACTTTTAAAAGGCGCTTTTTTATATTTCTTTTATAATAAATATAATT : 1462
cen4-CR3 : ---------------------------------------------------------------------------------------------------- :    -
cen4-CR4 : TAAATATAT------TTAAGGAGGAGGTAAAGAAGTATTTTTATTTTTTTATTACTTTTAAAAAGCGCTTTTTTATATTTCCTTTATAATAAATATAGTT : 1547
cen4-CR5 : ---------------------------------------------------------------------------------------------------- :    -
cen5-CR1 : TAAAAACAC------TTAAGGAAAAGGTAAAGGAGTATTTTTATCTTTTTATTACTTTTAAAAGGCGCTTTTTTATATTTCTTTTATAATAAATATAATT : 1355
cen6-CR1 : TAAAAATAC------TTAAAAAAAAGATAAAAAAATATTTTTATCTTTTTATTACTTTTAAAAAGCGCTTTTTTATATTTTCTTTATAATAAATATAATT : 1561
cen7-CR1 : TAAAAATAC------TTAAAAAAAAAGTAAAAAAGCATTTTTATTTTTTTATTACTTTTAAAAGGCGCTTTTTTATATTTCCTTTATAATAAATATAGTT : 1321
cen7-CR2 : TAAATATAC------TTAAGGAAAAGATAAAAAAGTATTTTTATTTTTTTATTATTTTTAAAAAGCGCTTTTTTATATTTTCTTTATAATAAATATAATT : 1361
cen7-CR3 : TAAATATA-------TTTAAAAAAAAATAAAAAAGTATTTTTATTTTTTTATTACTTTTAAAAGGCACTTTTTTATATTTTTTTTATAATAAATATAATT : 1327
```

```
                        *       1620          *       1640          *       1660          *       1680          *       1700
cen1-CR1 : AAGGTTTAAGTTATTATAATTACTTAATAATATTTAGTAAAAGATTATTAATTTTTATAATAATTTCTTTTTAACTTTAAAATACTTTTATATTTTTTTA : 1487
cen1-CR2 : AGGGTTTAAGTTATTATAATTACTTAATAAAATTTAGCAGAAGGTTATTAATTTTTATAATAACTTCCTTTTAGCTTTAAAGTGCCTTTATATCTTTTTA : 1591
cen1-CR3 : AAGGTTTAAGTTACTATAATTACTTAATAAGATTTAGCAGGAGGTTATTAATCTTTATAATAACTTTCTTTTAGCTTTAAAGCGCCTTTATATCTCTTTA : 1637
cen1-CR4 : AAGGTTTAAGTTACTGTAATTATTTAATAAGATTTAGCAGGAGGTTATTAATTTTTATAATAACTTCCTTTTAGCTTTAAAGCGCCTTTATATCTCTTTA : 1305
cen1-CR5 : AGGGTTTAAGTTACTATAATTACTTAATAAAATTTAATAAAAGGTTATTAATTTTTATAATAACTTTCTTTTAGCTTTAAAGCGCCTTTATATCTCTTTA : 1396
cen2-CR1 : AAGGTTTAAATTACTATAATTACTTAATAATATTTAATAAAAGGTTATTAATTTTTATAATAACTTTTTTTTAATTTTAAAGTAATTTTATATTTCTTTA :  392
cen2-CR2 : AGGGTTTAAGTTATTATAATTACTTAATAATATTTAGTAAAAGGTTATTAATTTTTATAATAACTTCTTTTTAATTTTAAAGCGCTTTTATATTTCTTTA : 1645
cen2-CR3 : AAGGTTTAAGTTATTATAATTACTTAATAATATTTAGCAGGAGGTTATTAATCTTTATAATAACTTCTTTTTAACTTTAAAGCACCTTTATATTTCTTTA : 1476
cen2-CR4 : ---------------------------------------------------------------------------------------------------- :    -
cen2-CR5 : ---------------------------------------------------------------------------------------------------- :    -
cen3-CR1 : ---------------------------------------------------------------------------------------------------- :    -
cen3-CR2 : AAGGTTTAAGTTATTATAATTACTTAATAATATTTAGCAGGAGGTTATTAATTTTTATAATAACTTCTTTTTAATTTTAAAGTATTTTTATAACTTTTTA : 1470
cen3-CR3 : AAGGTTTAAGTTATTATAATTATTTAATAATATTTAATAAAAGGTTATTAATTTTTATAATAACTTTTTTTTAACTTTAAAGCGCTTTTATATCTTTTTA : 1483
cen3-CR4 : AAGGTTTAAGTTATTATAATTACTTAATAATATTTAGTAAAAGGTTATTAATTTTTATAATAACCTTTTTTTAATTTTAAAATGCTTTTATAT-TTTTTA : 1421
cen4-CR1 : ---------------------------------------------------------------------------------------------------- :    -
cen4-CR2 : AAAGTTTAAGTTATTATAATTACTTAATAATATTTAGCAGAAGGTTATTAATCTTTATAATAACTTCTTTTTAACTTTAAAGCGCTTTTATAT-TTTTTA : 1561
cen4-CR3 : ---------------------------------------------------------------------------------------------------- :    -
cen4-CR4 : AAGGTTTAAGTTACTATAATTATTTAATAATATTTAGCAGGAGGTTATTAATTTTTATAATAACTTCTTTTTAATTTTAAAGTGCTTTTATATCTTTTTA : 1647
cen4-CR5 : ---------------------------------------------------------------------------------------------------- :    -
cen5-CR1 : AAGGTTTAAATTACTATAATTACTTAATAATATTTAGTAAAAGGTTATTAATTTTTATAATAACTTCTTTTTAACTTTAAAGCGCCTTTATATTTCTTTA : 1455
cen6-CR1 : AAGGTTTAAGTTATTATAATTACTTAATAATATTTAGTAAAAGGTTATTAATTTTTATAATAACCTTTTTTTAATTTTAAAGTATTTTTATATTTTTTTA : 1661
cen7-CR1 : AAGGTTTAAGTTATTATAATTACTTAATAATATTTAGTAAAAGGTTATTAATTTTTATAATAACTTTCTTTTAATTTTAAAATACCTTTATATTTTTTTA : 1421
cen7-CR2 : AAGGTTTAAGTTATTATAATTACTTAATAATATTTAATAAAAGGTTATTAATTTTTATAATAACTTTTTTTTAATTTTAAAATACTTTTATATTTTTTTA : 1461
cen7-CR3 : AAGGTTTAAGTTATTATAATTACTTAATAATATTTAATAAAAGATTATTAATTTTTATAATAATTTCTTTTTAATTTTAAAATGCCTTTATATTTTTTTA : 1427

                        *       1720          *       1740          *       1760          *       1780          *       1800
cen1-CR1 : TATAAGTTTTATAAAATTATAAATATATATTTATAATAGCTTTAGTATTTAGTTTTCTTTTTTTAATTATTTTATATTTTATTTTTAATTTTAATTAATA : 1587
cen1-CR2 : TATAATTTTTATAAAGTTATAAATATATACTTATAATAGCTTTAGTATTTAATTTTCTTCTTTTAGCTATTTTATATTTTATTTCTAGTTTTAATTAATA : 1691
cen1-CR3 : TATAATTTTTATAAAGTTATAAATATATACTTATAATAGCTTTAGTATTTAGTTTCCTTTTTTTAATTATTTTATATTTTATTTTTAATTTTAATTAGTA : 1737
cen1-CR4 : TATAATTTCTGCAGGGTTATAAATATGCACTTATAATAGCTTTAGTATTTAGTTTCCTTTTTTTAGCTATTTTATATTTTATTTTTAATTTAGTTAATA : 1405
cen1-CR5 : TATAATTTCTATAAGGTTATAAATATATACTTATAATAGCTTTAGTATTTAATTTCCTCCTTTTAGCTATTTTATATTTTATTTCTAATTCTAGTTAGTA : 1496
cen2-CR1 : TATAATTTTTATAAAATTATAAATATATATTTATAATAGCTTTAGTATTTAGTTTTTTTCTTTTAGCTATTTTATATTTTATTTTTAATTTTAATTAATA :  492
cen2-CR2 : TATAATTTTTATAAGGTTATAAATATATATTTATAATAGCTTTAGTATTTAGTTTTTTTTTTTTAGCTATTTTATATTTTATTTTTAATTTTAGTTAGTA : 1745
cen2-CR3 : TATAATTTCTATAAGGTTATAAATATATATTTATAATAGCTTTAGTATTTAATTTCCTTTTTCTAGCTATTTTATATTTTATTTCTAGTTTTAATTAGTA : 1576
cen2-CR4 : ---------------------------------------------------------------------------------------------------- :    -
cen2-CR5 : ------------------ATAAACGCTTACTTATAGCAGTTATA-------------------------------------------------------- :   47
cen3-CR1 : ------------------------------------------------------------------------------TTATTATAAATTTTAATTAATA :   22
cen3-CR2 : TATAATTTTTATAAAGTTATAAATATATATTTATAATAGCTTTAATATTTAATTTCCTTTTTTTAGCTATTTTATATTTTATTTTTAATTTTAGTTAATA : 1570
cen3-CR3 : TATAATTTTTATAAAATTATAAATATATACTTATAATAGCTTTAATATTTAATTTCTTTTTTTTAACTATTTTATATTTTATTTTTAATTTTAATTAATA : 1583
cen3-CR4 : TATAATTTTTATAAGGTTATAAATATATATTTATAATAGCTTTAATATTTAGTTT---TTTTTTAGCTATTTTATATTTTATTTTTAATTTTAATTAATA : 1518
cen4-CR1 : ---------------------------------------------------------------------------------------------------- :    -
cen4-CR2 : TATAATTTTTATAAGATTATAAATATATACTTATAATAGCTTTAGTATTTAATTTCTTTTTTTTAGCTATTTTATATTTTATTTTTAGTTTTAATTAATA : 1661
cen4-CR3 : ---------------------------------------------------------------------------------------------------- :    -
cen4-CR4 : TATAATTTTTATAAAATTATAAATATATACTTATAATAACTTTAATATTTAATTTTCTTTTTTTAGCTATTTTATATTTTATTTTTAATTCTAATTAGTA : 1747
cen4-CR5 : ---------------------------------------------------------------------------------------------------- :    -
cen5-CR1 : TATAATTTTTATAAGGTTATAAATATATACTTATAATAGCTTTAGTATTTAATTTCCTTTTTTTAGCTATTTTATATTTTATTTCTAGTTTTAGTTAGTA : 1555
cen6-CR1 : TATAATTTTTATAAAATTATAAATATATACTTATAATAGCTTTAGTATTTAATTTCCTTTTTTTAGCTATTTTATATTTTATTTTTAATTCTAGTTAATA : 1761
cen7-CR1 : TATAATTTTTATAAAATTATAAATATATATTTATAATAACTTTAGTATTTAATTT-TTTTTTTTAACTATTTTATATTTTATTTTTAATTTTAATTAATA : 1520
cen7-CR2 : TATAATTTTTATAAAATTATAAATATATATTTATAATAGCTTTAGTATTTAATTT--TTTTTTTAGCTATTTTATATTTTATTTTTAATTTTAATTAGTA : 1559
cen7-CR3 : TATAATTTTTATAAAGTTATAAATATATATTTATAATAGCTTTAATATTTAGTTT---TTTTTTAACTATTTTATATTTTATTTTTAATTTTAGTTAGTA : 1524
                             ataaa      a ttata  a  t ta                                 ttatt    a tt ta tta ta
```

```
                     *       1820          *       1840          *       1860          *       1880          *       1900
cen1-CR1 : ATTTAATATATTATTTTAAGCTTTTT----TTTTATAAGTAATTACTTTTCTTTTATTATAATATTTTAATATATTTAATAAAATATTTATAAAAAAGTT : 1683
cen1-CR2 : GTTTAGTATATTATTTTAGGCTTTTTTTTTTTATAAGTAGTTACCTTTCTTTTATTATAGTATTTTAATATATTTAGTAAGATATTTATAATAAAGTT : 1791
cen1-CR3 : GTTTAGTATATTATTTTAGGCTTTTTTCCTTTTATAAGTAATTACCCTTTTTTTATTATAGTATTTTAATATACTTAGTAAGATATTTATAATAAAGTT : 1837
cen1-CR4 : ATTTAATATATTATTTTAGGCTTCTTTCCTTTTATAAGCAGTTGCCCTTTTTTTATTATAGTATTTTAATATACTTAATAAAATATTTATAGTAAAGTT : 1505
cen1-CR5 : ATTTAATATATTATTCTAGGCTTCTTTCTTTTTATAAGCAGTTACTCTTCCTTTATTATAATATCTTAATATACTTAGTAAGATATTTATAATAAAGTT : 1596
cen2-CR1 : ATTTAATATATTATTTTAAGCTTTTT----TTTTATAAGTAGTTATTTTTTTTTATTTATAATATTTTAATATATTTAATAAAATATTTATAAAAAAGTT :  588
cen2-CR2 : ATTTAATATATTATTTTAAGCTTCTTTTTTTTTTATAAGCAGTTGCCTTTTTTTTATTATAGTATTTTAATATATTTAGTAAGATATTTATAAAAAAGTT : 1845
cen2-CR3 : ATTTAGTATATTATTTTAAGCTTCCTTTCTTTTATAAGCAGTTGCCCTTCCTTTATTATAGCATTTTAATATATTTAGTAAGATATTTATAAAAAAGTT : 1676
cen2-CR4 : ---------------------------------------------------------------------------------------------------- :    -
cen2-CR5 : ---------------------------------------------------------------------------------------------------- :    -
cen3-CR1 : ATTTAATATATTATTTTAAGCTTTCTTTTTTTTTATAAGTAATTACTTTTTTTTTATTATAGTATTTTAATATATTTAGTAAAATATTTATAAAAAAGTT :  122
cen3-CR2 : ATTTAGTATATTATTTTAAGCTTTTTTT--TTTTATAAGTAATTACTTTTTT----TTATAATATTTTAATATACTTAGTAAGATATTTATAAAAAAATT : 1664
cen3-CR3 : GTTTAATATATTATTTTAGGCCTTTTT---TTTTATAAGCAGTTGCCTTTTTTTTATTATAGTATTTTAATATACTTAATAAAATATTTATAAAAAAGTT : 1680
cen3-CR4 : GTTTAATATATTATTTTAAGCTTTTTTTTCTTTTATAAGTAGTTACCTTTTT-TTATTATAATATTTTAATATACTTAATAAGATATTTATAAAAAAATT : 1617
cen4-CR1 : ---------------------------------------------------------------------------------------------------- :    -
cen4-CR2 : GTTTAATATATTATTTTAGGTTTTTTTTTTTTTTATAAGTAGTTACCTTTTTTTTATTATAGTATTTTAATATACTTAATAAAATATTTATAAAAAAGTT : 1761
cen4-CR3 : ---------------------------------------------------------------------------------------------------- :    -
cen4-CR4 : ATTTAGTATATTATTTTAAATTTCCTTTTTTTTTATAAGCAGTTACTTTTTT-TTATTATAGTATTTTAATATACTTAGTAAAATATTTATAAAAAAGTT : 1846
cen4-CR5 : ---------------------------------------------------------------------------------------------------- :    -
cen5-CR1 : ATTTAATATATTATTTTAAGCTTCCTTTCTTTTATAAGCAGTTGCCCTTTTTTTATTATAATATCTTAATGCATTTAGTAGGATATTTATAAAAAAGTT : 1655
cen6-CR1 : ATTTAATATATTATTTTAAGCTTTTTTTTCTTTTATAAGTAATTACCCTTTTTTTATTATAGTATTTTAATATACTTAATAAAATATTTATAAAAAAGTT : 1861
cen7-CR1 : ATTTAATATATTATTTTAAACTTTCTTTT-TTTTATAAGTAATTACCTTTTTTTTATTATAGTATTTTAATATACTTAATAAGATATTTATAAAAAAGTT : 1619
cen7-CR2 : ATTTAATATATTATTTTAAGCTTTTT----TTTTATAAGTAATTACCTTTTTTTTATTATAATATTTTAATATACTTAATAAAATATTTATAAAAAAGTT : 1655
cen7-CR3 : ATTTAATATATTATTTTAAGCTTTTTTT--TTTTATAAGTAATTACCTTTTTCTTATTATAGTATTTTAATATATTTAATAAAATATTTATAAAAAAATT : 1622
              ttta tatattatt ta    t  t    ttttataag a tt    tt       ttata  at ttaat  a tta ta  atatttata  aaa tt

                     *       1920          *       1940          *       1960          *       1980          *       2000
cen1-CR1 : AATATAATATAATATAATAAGTTATTTTTTTTTAATAAAGTAAAAGTATAGTAAGTAAATTATTATTATATTATAATACTAATATAATAAATTAATAAAT : 1783
cen1-CR2 : AGTATAATATAGTATAGTAAGTTATTTTTTTTAAATAAAGTAAAAGTATAATAAGTAAATTATTATTATATTATAATATTAATATAGTAAATTAGTAAAT : 1891
cen1-CR3 : AGTATAATATAGTATAATAGGTTATTCTTTTTAAATAAAGTAAAAGTATAATAAGTAAATTACTATTATATTATAATACTAATATAATAAATTAATAAAT : 1937
cen1-CR4 : AGTATAATATAATATAGTAAGTTATTCTTTTTAAATAAAGTAAAAGTATAATAAGTAAATTATTATTATATTATAGTGCTAATATAGTAAATTAGCAGGT : 1605
cen1-CR5 : AGTATAATATAATATAGTAAGTTATTCTTTTTAAATAAAGTAAAAGTATAGTAAGTAAATTACTATTATATTATAATATTAATATAGTAAATTAGTAAAT : 1696
cen2-CR1 : AATATAATATAATATAATAAGTTATTTTTTTTTAATAAAGTAAAAGTATAAAAAGTAAATTACTATTATATTATAATATTAATATAATAAATTAGTAAAT :  688
cen2-CR2 : AATATAATATAGTATAGTAAGTTATTTTTTTTTAATAAAGTAAAAGTATAGTAAGTAAATTACTATTATATTATAATATTAATATAATAAATTAGTAAAT : 1945
cen2-CR3 : AGTATAATATAGTATAGTAAGTTATTCTTTTTTAATAAAGTAAGAGTATAGTAAGTAAATTACTATTATATTATAATACTAATATAGTAAATTAGCAGGT : 1776
cen2-CR4 : ---------------------------------------------------------------------------------------------------- :    -
cen2-CR5 : ------------------AAGCTAGTTTTTTAATATAAAGTAAAAGTATAATAAGTAAATTATTATTATATTATAATATTAATATAATAAATTAATAAAT :  129
cen3-CR1 : AATATAATATAATATAATAAGTTATTTTTTTTTAATAAAGTAAAAGTATAATAAGTAAATTACTATTATATTATAATATTAATATAATAAATTAGCAGAT :  222
cen3-CR2 : AATATAATATAATATAATAAGTTATTTTTTTTTAATAAAGTAAAAGTATAATAAGTAAATTATTATTATATTATAATATTAATATAATAAATTAGCAGGT : 1764
cen3-CR3 : AATATAATATAATATAGCAGGTTATTCTTTCTTAATAAAGTAAAAGTATAGTAAATAAATTACTATTATATTATAATATTAATATAATAAATTAATAAAT : 1780
cen3-CR4 : AATATAATATAATATAATAAGTTATTTTTTTTTAATAAAGTAAAAGTATAATAAGTAAATTACTATTATATTATAATATTAATATAGTAAATTAGTAAAT : 1717
cen4-CR1 : ---------------------------------------------------------------------------------------------------- :    -
cen4-CR2 : AGTATAATATAATATAATAAGTTATTCTTTTTTAATAAAGTAAAAGTATAATAAGTAAATTATTATTATATTATAATATTAATATAGTAAATTAGTAAAT : 1861
cen4-CR3 : ---------------------------------------------------------------------------------------------------- :    -
cen4-CR4 : AGTATAATATAATATAATAAGTTATTCTTTTTTAATAAAGTAAAAGTATAATAAGTAAATTACTATTATATTATAATATTAATATAGTAAATTAGTAAAT : 1946
cen4-CR5 : ---------------------------------------------------------------------------------------------------- :    -
cen5-CR1 : AGTATAATATAGTATAGTAGGTTATTCTTTCTTAATAAAGTAAAAGTATAGTAAGTAAATTACTATTATGTTATAATATTAATATAGTAAATTAGTAAAT : 1755
cen6-CR1 : AATATAATATAATATAGCAGGTTATTCTTTTTTAATAAAGTAAAAGTATAATAAGTAAATTATTATTATATTATAATATTAATATAGTAAATTAGTAAAT : 1961
cen7-CR1 : AATATAATATAATATAATAAGTTATTTTTTCTTAATAAAGTAAAAGTATAATAAGTAAATTATTATTATATTATAATATTAATATAGTAAATTAGTAAGT : 1719
cen7-CR2 : AATATAATATAATATAGTAAGTTATTCTTTTTTAATAAAGTAAAAGTATAATAAGTAAATTACTATTATATTATAATATTAATATAGTAAATTAGTAAAT : 1755
cen7-CR3 : AATATAATATAATATAATAAGTTATTCTTTTTTAATAAAGTAAAAGTATAGTAAGTAAATTATTATTATATTATAATATTAATATAATAAATTAGTAAAT : 1722
            a tataatata tata  a gttatt ttt t aataaagtaaaagtata taagtaaatta tattatattataata taatata taaatta  a  t
```

```
                           *        2020         *        2040         *        2060         *        2080         *        2100
cen1-CR1 : ACTATTATATAATAATATATATATAAAAAGTAAGATTTTATATA-------------CCTTTTTTTTTTCTTTTTTTTTATACTATCTTTTTTAAAGTAT : 1870
cen1-CR2 : ACTGCTATATAATAGTATATATATAAAAGGTAAGATTTTATATA-CCTTTTTTCTTTTTTTTTTTTTCTTTTTTTTTTATACTATCTTTTTTAAGGTAT : 1990
cen1-CR3 : ACTGCTATATAATAATATATATATAAAAGGCAAAATCTTATATA--TTCTTTTTTCTCTTTTTTTTTTTTTTTTTTTTTATACTATCTTTTTTAAAGTAT : 2035
cen1-CR4 : ACTGCTATATAATAATATATATATAAAAGGCAAGATCTTATATA--CCTTTCTTCCTCTTCTTTTTTTTTTTCTTTTTTATACTCTCTCCTTTAAGGTAT : 1703
cen1-CR5 : ACTATTATATAATAGTATATATATAAAAGGCAAGATCTTATATATTTTTTTTTCTTTTTTTTTTTTTTTTTTTTTTTTTTATACCCTCTCCTTTAAAGTAT : 1796
cen2-CR1 : ACTATTATATAATAATATATATATAAAAAGTAAAATCTTATATA------------------------CTTTTTTTTTTATATTATCTTTTTTAAAATAT :  764
cen2-CR2 : ACTGCTATATAATAATATATATATAAAAGGTAAGATTTTATATA--------CTTTTTTTTTTTTTTCTTTTCTTTTTTATACTATCTTTTTTAAGGTAT : 2037
cen2-CR3 : ACTGCTATATAACAGTATATATATAAAAGGCAAGATCTTATATA-CCTTTTTTTCTCTTTTTTTTTTCTTTTCTTTTTTATACTATCTCCTTTAAGGTAT : 1875
cen2-CR4 : ---------------------------------------------------------------------------------------------------- :    -
cen2-CR5 : ATTATTATATAATAGTATATATATAAAAAATAAAATTTTATATA---------------TATATATATATATTTTTTTTATACTATTTTTTTTAAAATAT :  214
cen3-CR1 : ACTATTATATAATAGTATATATATAAAAGGTAAGATTTTATATA----------------------TATATTTTTTTTATACTATTTTTTTTAAAATAT :  300
cen3-CR2 : ACTGCTATATAATAGTATATATATAAAAGGCAAGATCTTATATA-CCTTTTTTTCTTTTCTTTTTTTCTTTTCTTTTTTATACTATCTCCTTTAAGGTAT : 1863
cen3-CR3 : ATTATTATATAATAATATATATATAAAAAGTAAAATCTTATATA----------------TTTTTTTTTCTTTTTTTTTTATATTATTTTCTTTAAAGTAT : 1864
cen3-CR4 : ACTATTATATAATAATATATATATAAAAAGTAAGATTTTATATA--------------------------CTTTTTTTTTATATTATCTTTTTTAAAATAT : 1791
cen4-CR1 : ---------------------------------------------------------------------------------------------------- :    -
cen4-CR2 : ATTATTATATAATAATATATATATAAAAGGTAAGATTTTATATA------------CCTTTTTTTTTTTTTTTTTTTTTTTATACTATTTTCTTTAAGGTAT : 1949
cen4-CR3 : ---------------------------------------------------------------------------------------------------- :    -
cen4-CR4 : ATTATTATATAATAATATATATATAAAAAGTAAGATTTTATATA---------------TTTTTTTTTCTTTTTTTTTTATACTATTTTCTTTAAGGTAT : 2031
cen4-CR5 : ---------------------------------------------------------------------------------------------------- :    -
cen5-CR1 : ACTGCTATATAATAGTATATATATAAAAGGCAAGATTTTATATA--CCTTTCTTCCTCTTTTTCTTTTTTTTTTTTTTTATACTATCTTTTTTAAAGTAT : 1853
cen6-CR1 : ACTGCTATATAATAATATATATATAAAAGGTAAGATTTTATATA-----------------CTTTTTTTTTTTTTTTTTTATATTATTTTTTTTAAGGTAT : 2044
cen7-CR1 : GCTGCTATATAATAATATATATATAAAAGGTAAGATTTTATATA-------------CTTTTTTTTTTCTTTTTTTTTTATACTATCTTTTTTAAGATAT : 1806
cen7-CR2 : ACTATTATATAATAGTATATATATAAAAGGTAAAATTTTATATA-------------CTTTTTTTTTTCTTTTTTTTTTATATTATTTTCTTTAAAGTAT : 1842
cen7-CR3 : ACTATTATATAATAGTATATATATAAAAAGTAAAATCTTATATA---------------------------TTTTTTTTTTATATTATTTTTTTTAAAATAT : 1795
             a t  tatataata tatatatataaaa g aa at ttatata                           t ttttttata t t t  tttaa  tat

                           *        2120         *        2140         *        2160         *        2180         *        2200
cen1-CR1 : ATACTATATATAAGTATACTACGCTATTTAATTATAAAGATATTATAATAAAGTATTAAGTTATTATATTATAAAGTATAGTATATTAATAAATTAGTAA : 1970
cen1-CR2 : ATGCTATATATAAGCATACTGCGCTATTTAATTATAAAGATATTATAATAAAGTGTTAAGTTATTATATTATAAGGTATAGTATATTAATAAGTAAGTAA : 2090
cen1-CR3 : ATACTATATATAAGCATGCTGCATTATTTAATTATAAAAATATTATAATAAAGTATTAAGTTATTATATTATAAAGTATAATATATTAATAAGTAAGTAA : 2135
cen1-CR4 : ATGCTATATATAAGCGCGCTATACTATTTAATTATAAAGATATTATAATAAAGTGTTAAGTTATTATATTATAAGGTATAGTATACTAATAGGTAAGTAA : 1803
cen1-CR5 : ATGCTATATATAAGTGCACTATATTATTTAATTATAAAGATATTATAATAAAGTATTAAGTTATTATATTATAAAGTATAGTATATTAATAAGTAGGTAA : 1896
cen2-CR1 : ATACTATATATAAATATATTATACTATTTAATTAAAAAAATATTATAATAAAGTATTAAGTTATTATATTATAAGGTATAGTATATTAATAAGTAAGTAA :  864
cen2-CR2 : ATATTATATATAAGCGCACTGCACTATTTAATTATAAAGATATTATAATAAAGTGTTAAGTTATTATATTATAAAGTAAAGTATATTAATAAATAGGTAA : 2137
cen2-CR3 : ATGCTATGCATAAGCGCACTATACTATTTAATTATAAAGATATTATAATAAAGTGTTAAGTTATTATATTATAAGGTAAAGTATGCTAATAAGTAGGTAA : 1975
cen2-CR4 : ---------------------------------------------------------------------------------------------------- :    -
cen2-CR5 : ATATTATATATAAGTATATTATATTATTTAATTATAAAAATATTATAATAAAGTATTAAGTTATTATATTATAAAGTATAGTATATTAATAAGTAAGTAA :  314
cen3-CR1 : ATATTATATATAAGTATATTATATTATTTAATTATAAAAATATTATAATAAAGTATTAAGTTATTATATTATAAAGTATAGTATATTAATAAGTAAGTAA :  400
cen3-CR2 : ATGCTATGCATAAGCGCGCTATACTATTTAATTATAAAAATATTATAATAAAGTATTAAGTTATTATATTATAAGGTATAGTATGCTAATAAATAAGTAA : 1963
cen3-CR3 : ATATTATATATAAGTATATTATACTATTTAATTATAAAAATATTATAATAAAGTATTAAATTATTATATTATAAAGTATAATATATTAATAAGTAAGTAA : 1964
cen3-CR4 : ATACTATATATAAATATACTATACTATTTAATTATAAAAATATTATAATAAAGTATTAAGTTATTAAATTATAAAGTATAGTATATTAATAAGTAAGTAA : 1891
cen4-CR1 : ---------------------------------------------------------------------------------------------------- :    -
cen4-CR2 : ATATTATATATAAGCATATTATACTACTTAATTATAAAAATATTATAATAAAGTATTAAGTTATTATATTATAAAGTATAGTATATTAATAAGTAAGTAA : 2049
cen4-CR3 : ---------------------------------------------------------------------------------------------------- :    -
cen4-CR4 : ATGCTATATATAAGTATACTATATTATTTAATTATAAAGATATTATAATAAAGTATTAAGTTATTATATTATAAGGTATAGTATATTAATAAGTAAGTAA : 2131
cen4-CR5 : ---------------------------------------------------------------------------------------------------- :    -
cen5-CR1 : ATACTATATATAAGCATACTATGCTATTTAATTATAAAGATATTATAATAAAGTGTTAAGTTATTATATTATAAGGTATAGTATACTAATAAGTAAGTAA : 1953
cen6-CR1 : ATATTATATATAAGTATACTATACTATTTAATTATAAAAATATTATAATAAAGTATTAAGTTATTATATTATAAAGTATAATATATTAATAAATAAGTAA : 2144
cen7-CR1 : ATACTATATATAAGTATACTATACTATTTAATTATAAAAATATTATAATAAAGTATTAAATTATTATATTATAAAGTATAATATATTAATAAGTAAGTAA : 1906
cen7-CR2 : ATACTATATATAAGTATATTATATTATTTAATTATAAAGATACTATAATAAAGTATTAAATTATTATATTATAAAGTATAGTATATTAATAAATAAATAA : 1942
cen7-CR3 : ATACTATATATAAGTATATTATACTATTTAATTATAAAAATATTATAATAAAGTATTAAGTTATTATATTATAAAGTATAGTATATTAATAAGTAAGTAA : 1895
             at  tat  ataa      t    tatttaattataaa atattataataaagt ttaa ttattatattataa gta a tat  taataa ta gtaa
```

```
                    *      2220         *      2240         *      2260         *      2280         *      2300
cen1-CR1 : ATACT------TAATAATAATAATAATAATAATAATAACTATTAAAAGGTAATATCCT-TTTTTTTATACTTATTTAT--AATTTACTATAAGTCTTTAA : 2061
cen1-CR2 : ATGCT------------TAATAATAATAATAATAATAACTACTAAGGGGTAATATCCCCCCCTTTTATATTTATTTAT--AATTTACTATAAGTCTTTAA : 2176
cen1-CR3 : ATGCT------------TAATAATAATAGTAATAATAACTATTAAAAGGTAATATCCCCCTCTTTTATATTTATTTAT--AATTTACTATAAGTCCTTAA : 2221
cen1-CR4 : ATGCT------------AAATAATAATAATAATAATAACTACTAGGGGGTAATATCCCCCCCTTTTATACCTATTTAT--AATTTACTATAAGTCCTTAA : 1889
cen1-CR5 : ATACT------------AAATAATAATAATAATAATAATTATTAAGGGGTAATATCCCCCCCTTTTATACCTATTTAT--AGTTTACTATAAGTTCTTAA : 1982
cen2-CR1 : ATACTAAATAATAATAATAATAATAATAATAATAATAATAATTAAAAAGTAATATCCTCTTTTTTTATATTTATTTAT--AATTTACTATAAGTCTTAAA :  962
cen2-CR2 : ATGCT---------AAATAATAATAATAATAATAATAACTATTAAAAGGTAATATCCCCCTTTTTTATACTTATTTAT--AATTTACTATAAGTCCTTAA : 2226
cen2-CR3 : ATACT---------AAATAATAATAATAATAATAATAACTGCTAAAGGGTAATATCCCCCCCTTTTATACCTATTTAT--AGTTTACTATAAGTCCTTAA : 2064
cen2-CR4 : ---------------------------------------------------------------------------------------------------- :    -
cen2-CR5 : ATACT------AAATAATAATAGTAATAATAATAATAACTATTAAAAAGTAATATCCTTTTTTTTTATATTTACTTAA--GGTTTACTATAAGTCCTTAA :  406
cen3-CR1 : ATACTTAATAATAATAATAATAATAATAATAATAATAATTATTAAAAAGTAATATCCTTTTTTTTTATACTTATTTAT--AATTTATTATAAGTTTTTAA :  498
cen3-CR2 : ATGCT---TAATAATAATAGTAATAATAATAATAATAACTATTAAAAGGTAATATCTTCCCTTTTTATACCTATTTAT--AATTTACTATAAGTCCTTAA : 2058
cen3-CR3 : ATACT------TAATAATAATAATAATAATAATAATAATTATTAAAAAATAATATCCTTTTTTTTTATACTTATTTATAAAATTTTTTATAAGTTTTTAA : 2058
cen3-CR4 : ATACTTAATAATAATAGTAATAATAATAATAATAAT------TAAAAAATAATATCCCCTTTTTTTATATTTATTTAT--AATTTATTATAAATCCTTAA : 1983
cen4-CR1 : ---------------------------------------------------------------------------------------------------- :    -
cen4-CR2 : ATATT------------AAATAATAATAATAATAATAACTATTAAGAGGTAATATCCCCCTTTTTTATATTTATTTAT--AATTTACTATAAGTCCTTAA : 2135
cen4-CR3 : ---------------------------------------------------------------------------------------------------- :    -
cen4-CR4 : ATACT------------AAATAATAATAATAATAATAACTGCTTAAGGGTAATATCCCCCTTTTTTATACTTATTTAT--AATTTACTATAAGTTTTTAA : 2217
cen4-CR5 : ----------------------------AT---------------------------------------------------------------------- :    2
cen5-CR1 : ATGCT------TAATAATAATAGTAATAATAATAATAACTGCTAAAAGGTAATATCCCCCCCTTTTATACTTATTTAT--AGTTTACTATAAGTTCTTAA : 2045
cen6-CR1 : ATACT------AAATAATAATAATAATAATAATAATAATTACTAAAAGGTAATATCCCCTTTTTTTACTTTTATTTAA--GGTTTATTATAAGTCTTTAA : 2236
cen7-CR1 : ATACT------AAATAATAATAATAATAATAATAATAACTATTAAAAGGTAATATCCCCCCTTTTTATATTTATTTAA--GGTTTACTATAAATCCTTAA : 1998
cen7-CR2 : ATACTTAATAATAATAATAATAATAATAATAATAATAATTACTAAAAGATAATATCCCCCTTTTTTATATTTATTTAT--AATTTACTATAAGTTTTTAA : 2040
cen7-CR3 : ATACT------TAATAATAATAGTAATAATAATAATAATTATTAAAAAGTAATATCCTTTTTTTTTATACTTATTTAT--AATTTACTATAAGTTTTTAA : 1987
           at ct             aata taataataataataa    taa    taatatcc     ttttata  tattta     ttta tataa t  ttaa

                    *      2320         *      2340         *      2360         *      2380         *      2400
cen1-CR1 : TAGCAGTATATTATAAAAAGATATTAATAATTATAATAATTATATTTCTTATATTAAATATTATAATTATATATTTATATTTAATTTAGTAAATAGTAAT : 2161
cen1-CR2 : TAGCAGTTTATAGTAAGGAAATATTAATAATTATAGTAATTATATTCCTTATATTAAGTATTATAACTATATATTTATATTTAATTTAGTAAATAATAAT : 2276
cen1-CR3 : TAGCAGTTTATAATAAGAAAATATTAATAATTATAATAATTATATTCCTTATATTAAGTATTATAACTATATATTTATATTTAATTTAGTAAATAGTAAT : 2321
cen1-CR4 : TAGTAGTTTATAATAAGGAAATACTAATAATTATAATAACTATATTCCTTATATTAAGTATTATAATTATATATTTATATTTAATTTAGTAAATAGTAAT : 1989
cen1-CR5 : TAGCAGTTTATAATAAGAAAATATTAATAATTATAATAACTATATTCTTTATATTAAGTGTTATAACTATATATTTATATTTAATTTAGTAAATAGTAAT : 2082
cen2-CR1 : TAATAGTTTATTATAAAAAGATATTAATAATTATAATAATTATATTTTTTATATTAAGTATTATAATTATATATTTATATTTAATTTAGTAAATAATAAT : 1062
cen2-CR2 : TAGCAGTTTATAATAAGGAAATATTAATAATTATAATAACTATATTCCTTATATTAAGTATTATAATTATATATTTATATTTAATTTAGTAAATAGTAAT : 2326
cen2-CR3 : TAGCAGTTTATAATAAGGAAATACTAATAATTATAATAACTATATTCCTTATATTAAGTATTATAACTATATATTTATATTTAATTTAGTAAATAGTAAT : 2164
cen2-CR4 : ---------------------------------------------------------------------------------------------------- :    -
cen2-CR5 : TAGCAGTTTATTATAAAAAAATATTAATAATTATAATAATTATATTTCTTATATTAAGTATTATAATTATATATTTATATTTAATTTAATAAATAATAAC :  506
cen3-CR1 : TAATAATATATTATAAAAAGATATTAATAATTATAATAATTATATTTTTTATATTAAGTACTATAATTATATATTTATATTTAATTTAGTAAATAGTAAT :  598
cen3-CR2 : TAGCAGTTTATTATAAAAAGATATTAATAATTATAGTAATTATATTCCCTGCATTAAGTATTATAATTATATATTTATATTTAATTTAGTAAATAGTAAT : 2158
cen3-CR3 : TAATAATATATTATAAAAAGATATTAATAATTATAATAATTATATTTCTTATATTAAGTATTATAACTATATATTTATATTTAATTTAGTAAATAGTAAT : 2158
cen3-CR4 : TAATAGTATATTATAAAAAGATATTAATAATTATAGTAATTATATTTTTTATATTAAATACTATAATTATATATTTATATTTAATTTAGTAAATAGTAAT : 2083
cen4-CR1 : ---------------------------------------------------------------------------------------------------- :    -
cen4-CR2 : TAGTAATTTATTATAAAGAAATATTAATAATTATAATAATTATATTTCTTATATTAAGTATTATAACTATATATTTATATTTAATTTAGTAAATAGTAAT : 2235
cen4-CR3 : ---------------------------------------------------------------------------------------------------- :    -
cen4-CR4 : TAGTAGTTTATTATAAGGAAATATTAATAATTATAATAATTATATTCCTTATATTAAGTATTATAACTATATATTTATATTTAATTTAATAAATAGTAAT : 2317
cen4-CR5 : ------------------------------------------TTTTTCTTATATAAAG------------------------GACTTAATAAA------- :   29
cen5-CR1 : TAGCAGTATATTATAAAAAGATATTAATAATTATAATAACTATATTCCTTATATTAAGTGCTATAACTATATATTTATATTTAATTTAATAAATAGTAAT : 2145
cen6-CR1 : TAGCAGTTTATTATAAGAAAATATTAATAATTATAATAACTATATTTCTTATATTAAGTATTATAACTATATATTTATATTTAACTTAGTAAATAATAAT : 2336
cen7-CR1 : TAATAGTTTATTATAAGAAAATATTAATAATTATAGTAATTATATTTTTTATATTAAGTATTATAATTATATATTTATATTTAATTTAGTAAATAATAAT : 2098
cen7-CR2 : TAATAGTATATTATAAAAAGATATTAATAATTATAATAATTATATTTTTTATATTAAGTGCTATAATTATATATTTATATTTAATTTAATAAATAGTAAT : 2140
cen7-CR3 : TAATAATATATTATAAAAAGATATTAATAATTATAATAATTATATTCTTTATATTAAGTATTATAATTATATATTTATATTTAATTTAGTAAATAGTAAT : 2087
           ta  a t tat ataa  a ata taataattata taa tatatt  ttatattaagt  tataa tatatatttatatttaattta taaata taat
```

```
                       *        2420         *        2440         *        2460         *        2480         *        2500
cen1-CR1 : ACTTTTAAAAGCTATTATTATACTAATATTAATAAAATTATACTATA-CTTATTATATTATAATAAAAATTAATATTAAGTAAGTTATATAGTTTATTAT : 2260
cen1-CR2 : ACTTTTAAAGGCTGCTATTATATTAATATTAGTAAGATTATAGTATA-CTTATTATATTATAATTAAAATTAATAATAAGTAAGTTATATAGTTTATTAT : 2375
cen1-CR3 : ACTTTTAAAGGCTGCTATTATATTAATACTAGTAAAATTATAATATA-CTTATTATATTATAACTAAAATTAATAATAAGTAAGTTATATAGTTTATTAT : 2420
cen1-CR4 : ACTTTTAAAGGCTGCTACTATACTAATACTAGTAAGATTATAATATA-CTTATTATATTATAATTAAAACTAATAATAAGTAGGTTATATAGTTTATTAT : 2088
cen1-CR5 : ACTTTTAAGGGCTACTATTATATTAATACTAGTAAAATTATAATATA-CTTATTATATTATAATTAAAATTAATAATAAGTAAGTTATATAATTTATTAT : 2181
cen2-CR1 : ACTTTTAAAAGCTATTATTATATTAATATTAATAAAATTATAATATA-CTTATTATATTATAATAAAAACTAATATTAAGTAAGTTATATAATTTATTAT : 1161
cen2-CR2 : ACTTTTAAAGGCTATTATTATACTAGTATTAGTAAGATTATAATATA-CTTATTATATTATAATAAAAATTAATAATAAATAAGTTATATAGTTTATTAT : 2425
cen2-CR3 : ACTCTTAAAGGCTACTATTATATTAGTATTAGTAAGATTATAATATA-CTTATTATATTATAATAAAAATTAATAATAAGTAAGTTATATAGTTTATTAT : 2263
cen2-CR4 : ---------------------------------------------------------------------------------------------------- :    -
cen2-CR5 : ACTTTTAAAAGCTATTACTATATTAATATTAATAAAATTATAATATA-CTTATTATATTATAATAAAAACTAATATTAAATAAGTTATATAATTTATTAT :  605
cen3-CR1 : ATTTTTAAAAGCTATTATTATATTAATATTAGTAAAATTATTATATATTTTATTATATTATAATAAAAACTAATATTAAATAAGTTATATAGTTTATTAT :  698
cen3-CR2 : ACTTTTAAAAGCTATTATTATACTAATACTAATAAGATTATAATATA-CTTATTATATTATAATAAAAACTAATACTAAGTAAGTTATATAATTTATTAT : 2257
cen3-CR3 : ACTTTTAAAAGCTATTATTATATTAATATTAATAAAATTATAATATA-TATATTATATTATAATAAAAACTAATATTAAGTAAGTTATATAATTTATTAT : 2257
cen3-CR4 : ACTTTTAAAGACTGCTATTATATTAATATTAATAAAATTATAATATA-CTTATTATATTATAATAAAAACTAATATTAAGTAAGTTATATAATTTATTAT : 2182
cen4-CR1 : ---------------------------------------------------------------------------------------------------- :    -
cen4-CR2 : ACTTTTAAAGGCTGCTATTATATTAATACTAGTAAGATTATAATATA-CTTATTATATTATAATAAAAATTAATAATAAGTAAGTTATATAGTTTATTAT : 2334
cen4-CR3 : ---------------------------------------------------------------------------------------------------- :    -
cen4-CR4 : ACTTTTAAAGGCTATTATTATATTAATATTAGTAAGATTATAATATA-CTTATTATATTATAATAAAAACTAATAATAAGTAAGTTATATAGTTTATTAT : 2416
cen4-CR5 : ------------------------GGTATTAATAA-----------------------------A-----TAGCTTTAATTAAGCTATTTAA-------- :   63
cen5-CR1 : ACTTTTAAAGGCTGCTATTATATTAATGCTAGTAAGATTATAATATA-CTTATTATATTATAATAAAAACTAATACTAAGTAAGTTATATAGTTTATTAT : 2244
cen6-CR1 : ACTTTTAAAGGCTATTATTATACTTATATTAGTAAAATTATAATATA-CTTATTATATTATAAT-AAAACTAATATTAAGTAAGTTATATAGTTTATTAT : 2434
cen7-CR1 : ACTTTTAAAAGCTATTATTATATTAATATTAGTAAAATTATAATATA-TTTATTATATTATAATAAAAACTAATATTAAATAAGTTATATAATTTATTAT : 2197
cen7-CR2 : ACTTTTAAAAGCTATTATTATATTAATATTAGTAAAATTATAATATA-CTTATTATATTATAATAAAAACTAATATTAAGTAAGTTATATAATTTATTAT : 2239
cen7-CR3 : ACTTTTAAAAGCTATTATTATATTAATATTTATAAGATTATAATATA-CTTATTATATTATAATAAAAACTAATACTAAGTAAGTTATATAGTTTATTAT : 2186
           acttttaaa gct  ta tata ta ta ta taa attata tata  ttattatattataat aaaa taata taa taagttatata tttattat

                       *        2520         *        2540         *        2560         *        2580         *        2600
cen1-CR1 : ATATATTATATTATTTATTATAAAATATATATACTTATTATAAATTCCTTAATTAATATTAGTATTTTTATAAATATAGCTATTATTATAAATAATATAA : 2360
cen1-CR2 : ATATACTATATTATTAATTATAAAATATATATACTTATTATAAATTCCCTAGTTAATATTAGCATTTTTATAAATATAGCTATTATTATAAATAATATAA : 2475
cen1-CR3 : ATATACTATATTATTAATTATAAAATATATATACTTATTATAAATTCTTTAATTAATACTAGTATTTTTATAAATATAGCTATTATTATAAATAATATAA : 2520
cen1-CR4 : ATATACTATATTATTAATTATAAAATATATATACTTATTATAAATTCCTTAATTAATACTAGCATTTTTATAAATATAGCTATTATTATAAATAATATAA : 2188
cen1-CR5 : ATATACTATATTATTAATTATAAAATATATATACTTATTATAAATTCCTTAATTAATATTAATATTTTTATAAATATAGTTATTATTATAAATAATATAG : 2281
cen2-CR1 : ATATATTTTATTATTTATTATAATATATATATACTTATTATAAATTTCTTAATTAATACTAGTATTTTTATAAATATAGCTATTATTATAAATAATATAA : 1261
cen2-CR2 : ATATATTATATTATTAATTATAAAATATATATATTTATTATAAATTCTTTAATTAATATTAGTATTTTTATAAATATAGCTATTATTATAAATAATATAA : 2525
cen2-CR3 : ATATACTGCATTATTAATTATAAAATATATATACTTATTATAAATTCTCTAGTTAATACTAGTATTTTTATAAATACAGCTATTATTATAAATAATATAG : 2363
cen2-CR4 : ---------------------------------------------------------------------------------------------------- :    -
cen2-CR5 : ATATATTATATTATTTATTATAAAATATATATACTTATTATAAATTCCTTAATTAATATTAGTATTTTTATAAATATAGCTATTATTATAAATAATATAA :  705
cen3-CR1 : ATATACTATATTATTTATTATAAAATATATATACTTATTATAAATTCTTTAATTAATATTAATATTTTTATAAATATAGCTATTATTATAAATAATATAA :  798
cen3-CR2 : ATATATTATATTATTTATTATAAAATATATATACTTATTATAAATTCTTTAGTTAATACTAGTATTTTTATAAATATAGTTATTATTATAAATAATATAA : 2357
cen3-CR3 : ATATATTATATTATTTATTATAAAATATATATATTTATTATAAATTCTTTAATTAATATTAGTATTTTTATTAATATAGCTATTATTATAAATAATATAG : 2357
cen3-CR4 : ATATATTATATTATTTATTATAAAATATATATATTTATTATAAATTCTTTAATTAATATTAGTATTTTTATAAATATAATTATTATTATAAATAATATAA : 2282
cen4-CR1 : ---------------------------------------------------------------------------------------------------- :    -
cen4-CR2 : ATATATTATATTATTAATTATAAAATATATATACTTATTATAAATTCCCTAGTTAATATTAATATTTTTATAAATATAGCTATTATTATAAATAATATAA : 2434
cen4-CR3 : ---------------------------------------------------------------------------------------------------- :    -
cen4-CR4 : ATATACTATATTATTAATTATAAAATATATATATTTATTATAAATTTTTTAATTAATATTAGCATTTTTATAAATATAGCTATTATTATAAATAATATAA : 2516
cen4-CR5 : ---------------------------------------------------------------------------------------------------- :    -
cen5-CR1 : ATATACTATATTATTTATTATAAAATATATATACTTATTATAAATTCCTTAATTAATACTAGTATTTTTATAAATATAGCTATTATTATAAATAATATAG : 2344
cen6-CR1 : ATATATTATATTATTTATTATAAAATATATATACTTATTATAAATTCCTTAATTAATACTAGTATTTTTATAAATATAGCTATTATTATAAATAATATAA : 2534
cen7-CR1 : ATATATTATATTATTTATTATAAAATAAATATACTTATTATAAATTCTTTAATTAATATTAGTATTTTTATAAATATAGCTATTATTATAAATAATATAA : 2297
cen7-CR2 : ATATATTATATTATTTATTATAAAATATATATACTTATTATAAATTCCTTAATTAATACTAGTATTTTTATAAATATAGCTATTATTATAAATAATATAA : 2339
cen7-CR3 : ATATATTATATTATTTATTATAAAATATATATATTTATTATAAATTCCTTAATTAATACTAGTATTTTTATAAATATAATTATTATTATAAATAATATAA : 2286
           atata t tattatt attataaaatatatata ttattataaatt   ta ttaata ta  atttttataaatata  tattattataaataatata
```

```
                    *      2620         *      2640         *      2660         *      2680         *      2700
cen1-CR1 : TAAGTATTTTTTATAATAACTATATAATATTTATATATTAATAATAAATAAAAATAATTAAAAGTAATATAAAATATATACTATAGTTTATTTATAAAAG : 2460
cen1-CR2 : TAAATACTTTTTATAATAACTTTATAATATTTATATATTAATAATAAATAAAAATAATTAAAAATAGCTTAAAATATATACTATAGCTTATTTATAAAAA : 2575
cen1-CR3 : TAAGTACTTTTTATAATAACTTTATAATATCTATATATTAATAATAAATAAAGATAGTTAAAAGTAGCTTAAAATATATACTATAGCCTATTTATAAAAA : 2620
cen1-CR4 : TAAGTGCTTTTTATAATAACTTTATAATATCTGCATATTAATAATAAATAAAGATAGTTAAAAGTAGCCTAAAATATATACTATAGCCTATTTATAAAAA : 2288
cen1-CR5 : TAAGTGCTTTTTATAATAACTTTATAGTATTTATATATTAATAATAAATAAAAATAATTAAAAGTAGTTTAAAATATATATTATAGCCTATTTATAAAAA : 2381
cen2-CR1 : TAAATACTTTTTATAATAACTATATAATATTTATATATTAATAATAAATAAAAATAATTAAAAATAGCTTAAAATATATACTATAGCCTATTTATAAAAA : 1361
cen2-CR2 : TAAATACTTTTTATAATAATTTTATAATATTTATATATTAATAATAAATAAAAATAGTTAAAAGTAGCCTTAAATATATACTATAGCCTATTTATAAAAG : 2625
cen2-CR3 : TAAGTGCTTTTTATAATAACTTTATAATATTTATATATTAATAATAAATAAAGACAGTTAAAAGTAGCCTAAAATATATACTATAGCCTATTTATAAAAG : 2463
cen2-CR4 : ---------------------------------------------------------------------------------------------------- :    -
cen2-CR5 : TAAATACTTTTTAT-ATAATTATATAATATTTATATATTAATAATAAATAAAAATAATTAAAAATAGCTTAAAATATATACTATAGCCTATTTATAAAAA :  804
cen3-CR1 : TAAGTATTTTTTATAATAATTATATAATATTTATATATTAATAATAAATAAAAATAATTAAAAATAGCTTAAAATATATACTATAGCTTATTTATAAAAA :  898
cen3-CR2 : TAAATACTGCTTAT-ATAACTATATAATATCTGCATATTAATAATAAATAAAGATAGTTTAAAATAGCTTAAAATATATACTATAGTTTATTTATAAAAA : 2456
cen3-CR3 : TAAGTACTTTTTATAATAATTATATAATATTTATATATTAATAATAAATAAAAATAATTAAAAATAGCTTAAAATATATATTATAGCTAGGCTATAAAAA : 2457
cen3-CR4 : TAAGTATTTTTTATAATAATTATATAGTATTTATATATTAATAATAAATAAAAATAATTAAAAATAGCTTAAAATATATATTATAATTAAATTATAAAAA : 2382
cen4-CR1 : ---------------------------------------------------------------------------------------------------- :    -
cen4-CR2 : TAAATACTTTTTATAATAATTATATAGTATTTATATATTAATAATAAATAAAAATAGTTAAAAGTAGCTTAAAATATATACTATAGCCTATTTATAAAAA : 2534
cen4-CR3 : ---------------------------------------------------------------------------------------------------- :    -
cen4-CR4 : TAAGTGCTTTTTATAATAATTATATAATATTTATATATTAATAATAAATAAAAATAATTAAAAATAGCTTAAAATATATATTATAGCTTATTTATAAAAA : 2616
cen4-CR5 : ---------------------------------------GGTAATAA----------------------------------------------------- :   71
cen5-CR1 : TAAGTGCTTTTTATAATAACTATATAATATTTATATATTAATAATAAATAAAGATAATTAAAAGTAATTTAAAATATATACTATAGCTTATTTATAAAAA : 2444
cen6-CR1 : TAAATGCTTTTTAT-ATAATTATATAATATTTATATATTAATAATAAATAAAAATAGTTAAAAGTAGCTTAAAATATATATTATAGCCTATTTATAAAGA : 2633
cen7-CR1 : TAAATACTTTTTAT-ATAATTATATAATATTTATATATTAATAATAAATAAAAATAATTAAAAGTAGCTTAAAATATATACTATAGCCTATTTATAAAAA : 2396
cen7-CR2 : TAAGTATTTTTTATAATAATTATATAATATTTATATATTAATAATAAATAAAAATAATTAAAAATAGCTTAAAATATATACTATAATTAAATTATAAAAA : 2439
cen7-CR3 : TAAATACTTTTTATAATAACTATATAGTATTTATATATTAATAATAAATAAAAATAATTAAAAATAGCTTAAAATATATACTATAGTTTATTTATAAAAA : 2386
           taa t  tttttat ataa t tata tat t  atattaataataaataaa ata ttaaaa ta   taaaatatata tata    a ttataaaa

                    *      2720         *      2740         *      2760         *      2780         *      2800
cen1-CR1 : AGTTTTAACTTTATTTATTTTTAATATAAATATATTATATATAATAAAAGCACTATAAGTATTATACTTTATTACTTTTAATAATATTATTTTAATTAAA : 2560
cen1-CR2 : AGTTTTAACTTTATTTACCTTTAATATAAATATATTATGCATAATAAAAATACTATAAGTATTATACCTTATTACCTTTAATAGTATTATTTTAATTAAA : 2675
cen1-CR3 : AGTCTTAACTTTATTTACCTTTAATATAAATATATTATATATAATAAAAGCGCTATAAGTATTATACTTTATTACCTTTAATAGTATTATTTTAATTAAA : 2720
cen1-CR4 : AGTCTTAATTTTATTTACCTTTAATATAAATGCATTATATATAATAAAAGCGCTATAGGCATTATACTTTATTACCCTTAGTAGTATTATCTTAATTAAA : 2388
cen1-CR5 : AGTCTTAACTTTATTTACCTTTAATATAAATATATTATATATAATAAGAGTGCTATAAGCATTATACCTTATTACCTTTAATAGCATTATCTTAATTAAA : 2481
cen2-CR1 : AGTCTTAACTTTATTTATTTTTAATATAAATATATTATATATAATAAAAGTGCTATAAGTATTATACTTTATTACTTTTAATAGTATTATTTTAATTAAA : 1461
cen2-CR2 : AGTTTTAACTTTATTTACCTTTAATATAAATATATTATATATAATAAAAGCGCTATAAGTATTATATCTTATTACCTTTAATAATATTATTTTAATTAAA : 2725
cen2-CR3 : AGTCTTAACTTTATTTACCTTTAATATAAATACATTATATATAATAAAAGCGCTATAAGTATTATACCTTATTACCCTTAGTAGCATTATCTTAATTAAA : 2563
cen2-CR4 : ---------------------------------------------------------------------------------------------------- :    -
cen2-CR5 : AGTCTTAACTTTATTTACTTTTAATATAAATATATTATATATAATAAAAGTGCTATAAGTATTATACTTTATTACTTTTAATAATATTATTTTAATTAAA :  904
cen3-CR1 : AGTTTTAACTTTATTTACTTTTAATATAAATATATTATATATAATAAAAGTATTATAAGTATTATACTTTATTACTTTTAATAGTATTATTTTAATTAAA :  998
cen3-CR2 : AGTCTTAACTTTATTTACCTTTAATATAAATATATTATATATAATAAAAGCGCTATAAGTATTATACCTTATTACCTTTAATAATATTATTTTAATTAAA : 2556
cen3-CR3 : AGTTTTAATTTTATTTACTTTTAATATAAATATATTATATATAATAAAAGTGCTATAAATATTATACCTTATTACTTTTAATAATATTATTTTAATTAAA : 2557
cen3-CR4 : AGTTTTAATTTTATTTACTTTTAATATAAATATATTATATATAATAAAAGTATTATAAGTATTATACTTTATTACTTTTAATAATATTATTTTAATTAAG : 2482
cen4-CR1 : ---------------------------------------------------------------------------------------------------- :    -
cen4-CR2 : AGTCTTAACTTTATTTATTTTTAATATAAATATATTATATATAATAAAAGCGCTATAAGTATTATATCTTATTACTTTTAATAATATTATCTTAATTAAA : 2634
cen4-CR3 : ---------------------------------------------------------------------------------------------------- :    -
cen4-CR4 : AGTCTTAACTTTATTTACTTTTAATATAAATATATTATATATAATAAAAGTGCTATAAATATTATACTTTATTACTTTTAATAATATTATTTTAATTAAA : 2716
cen4-CR5 : ----------------------AATATAAATATAT----------------------------------------------------------------- :   84
cen5-CR1 : AGTCTTAACTTTATTTACCTTTAATATAAATACATTATATATAATAAAAGCGCTATAAGTATTATACCTTATTACTTTTAATAGTATTATTTTAATTAAA : 2544
cen6-CR1 : AGTCTTAACTTTATTTACTTTTAATATAAATATATTATATATAATAAAAGTATTATAAATATTATATTTTATTACCTTTAATAGTATTATCTTAATTAAA : 2733
cen7-CR1 : AGTCTTAACTTTATTTACTTTTAATATAAATATATTATATATAATAAAAGTATTATAAGTATTATACTTTATTACCTTTAATAATATTATCTTAATTAAA : 2496
cen7-CR2 : AGTCTTAACTTTATTTACCTTTAATATAAATATATTATATATAATAAAAGTATTATAAATATTATACTTTATTACTTTTAATAATATTATCTTAATTAAA : 2539
cen7-CR3 : AGTCTTAATTTTATTTATTTTTAATATAAATATATTATATATAATAAAAGCATTATAAGTATTATATTTTATTACTTTTAATAATATTATTTTAATTAAA : 2486
           agt ttaa tttattta  tttaatataaata attatatataataaaag   tataa  attata  ttattac  tta ta  attat ttaattaaa
```

```
                        *       2820          *       2840          *       2860          *       2880          *       2900
cen1-CR1 : AAGTATAATATATAAATATAATATTATAATATAATAATATTATTAATTTATAATATAATTTTAAGTTTTATTTTTTATAAAAATAAATAAAAAGTAAAAA : 2660
cen1-CR2 : AAGCATAGTATATAAATATAATATTATAATATAATAGCATTACTAATTTATAATATAATTTTAAATCTTATTCCCTTTAAAAATAAATAAAAAATAAAAA : 2775
cen1-CR3 : AAGTATAATATATAAATATAGTATTATAATATAATAATATTATTAATTTATAATATAATCTTAAATCTTATTCTTTTTAAAAATAAATAAAAAATAAAAA : 2820
cen1-CR4 : AAGTATAGTATATAAATATAGTGTTATAGTATAATAGTATTACTAATTTATAATATAGTTTTAAGTCCTATTTCTTATAAAAAGAGATAAAAAGCAGAAA : 2488
cen1-CR5 : AAGCATAGTATATAAATATAGTATTATAGTATAATAGCATTACTAATTTATAATATAGTTTTAAGTCTTATTCCTTATAAAAAAAAATAAAAAGCAGAAA : 2581
cen2-CR1 : AAGTATAATATATAAATATAATATTATAATATAATAATATTATTAATTTATAATATAATTTTAAATCCTATTTTTTATAAAAATAAATAAAAAGTAAAAA : 1561
cen2-CR2 : AAGTATAATATATAAATATAGTATTATAATATAATAATATTACTAATTTATAATATAGTTTTAAATCCTATTCCTTTTAAAAATAAGTAAAAAGCAGAAA : 2825
cen2-CR3 : AAGCATAGTATATAAATATAATATTATAATATAATAGTATTACTAATTTATAATACAGTCTTAAGTCTTATTCCTTTTAAGAATAAGTAAAAAGCAGAAA : 2663
cen2-CR4 : ---------------------------------------------------------------------------------------------------- :    -
cen2-CR5 : AAGTATAATATATAAATATAATATTATAATATAATAATATTATTAATTTATAATATAATTTTAAGTTTTATTTTTTATAAAAATAAATAAAAAGTAAAAA : 1004
cen3-CR1 : AAGTATAATATATAAAAATAATATTATAATATAATAATATTACTAATTTATAATATAATTTTAAATCTTATTTTTTATAAAAATAAATAAAAAGTAAAAA : 1098
cen3-CR2 : AAGTATAGTATATAAATTTAATATTATAATATAATAATATTATTAATTTATAATACAGTTTTAAGTCCTATTCCTTATAAAAATAAATAAAAAGTAAAAA : 2656
cen3-CR3 : AAGTATAATATATAAATATAATATTATAATATAATAATATTATTAATTTATAATATAATTTTAAGTTTTATTTTTTATAAAAATAAATAAAAAGTAAAAA : 2657
cen3-CR4 : AAGTATAATATATAAATTTAATATTATAATATAATAATATTACTAATTTATAATATAATCTTAAGTTTTATTTTTTATAAAAATAAATAAAAAGTAAAAA : 2582
cen4-CR1 : ---------------------------------------------------------------------------------------------------- :    -
cen4-CR2 : AAGTATAGTATATAAATATAATATTATAATATAATAATATTATTAATTTATAATATAGTTTTAAGTCTTATTCCTTAAAGAAATAAATAAAAAGTAAAAA : 2734
cen4-CR3 : ---------------------------------------------------------------------------------------------------- :    -
cen4-CR4 : AAGTATAATATATAAATATAATATTATAATATAATAATATTATTAATTTATAATATAATTTTAAGTCTTATTTTTTAAAAAAATAAATAAAAAGCAGAAA : 2816
cen4-CR5 : ---------------------------------------------------------------------------------------------------- :    -
cen5-CR1 : AAGCATAGTATATAAATATAGTATTATAATATAATAATATTACTAATTTATAATATAATTTTAAATTCTATTTCTTATAAAAATAAATAAAAAGTAAAAA : 2644
cen6-CR1 : AAGTATAATATATAAATATAATATTATAATATAATAATATTACTAATTTATAATATAATTTTAAATTTTATTTTTTATAAAAGTAAATAAAAAGCAGAAG : 2833
cen7-CR1 : AAGTATAATATATGAATATAATATTATAATATAATAATATTACTAATTTATAATATAATTTTAAGTTTTATTTTTTATAAAAATAAATAAAAAGTAAAAG : 2596
cen7-CR2 : AAGTATAATATATAAATATAATATTATAATATAATAGTATTACTAATTTATAATATAATTTTAAGTTTTATTTTTTATAAAAATAAATAAAAAATAAAAG : 2639
cen7-CR3 : AAGTATAATATATAAATATAATATTATAATATAATAATATTATTAATTTATAATATAATTTTAAATTTTATTTTTTATAAAAATAAATAAAAAGTAAAAA : 2586
             aag ata tatataaat ta tattata tataata  atta taatttataata a t ttaa t  tatt  tt  aaaaa aa taaaaa  a aa

                        *       2920          *       2940          *       2960          *       2980          *       3000
cen1-CR1 : CTTATTATTATTAGCTTTAATAAAAAATTAAAAATAAGCTATATTATAATTTTTAATATTAAAGAGTTAAAGGTAAGTATAATAA--------AAAAAAA : 2752
cen1-CR2 : CCTATTATTATTAGCTTTAATAAAAAATTAAAGATAAGCTATATTATAATTTTTAATAATAAAAAGTTAAAGGTAGGTATAGTAA--------AGGAGGA : 2867
cen1-CR3 : CCTATTATTATTAGCTTTAATAAAGAATTAAAAATAAGCTATATTATAATTTTTAATAATAAGGAGTTAGAGGTAAGTATAGTAA--------AGGAGGA : 2912
cen1-CR4 : CCTATTATTATTAGCTTTAATAAAGAATTAAAAAGAAGCTATATTATAATTTCTAATAGTAAGAAGTTAGTAGTAAGTGTAATAA-----AGGAGGAGGA : 2583
cen1-CR5 : CTTATTATTATTAGCTTTAATAAAGAATTAAAAAGAAGCTATATTATAATTTTTAATAGTAAGGAGTTAATAATAAATATAGTAA--------AGGAGGA : 2673
cen2-CR1 : CTTATAGTTATTAGCTTTAATAAAAAATTAAAAATAAGCTATATTATAA-TTTTAATAATAAGAAGTTAAAAGTAAGTATAATAA---------AAAAAA : 1651
cen2-CR2 : CTTATTATTATTAGCTTTAATAAAAAATTAAAAATAAGCTATATTATAATTTTTAATAATAAGAAGTTAAGGGTAGGTATAGTAA--------AGGAGGA : 2917
cen2-CR3 : CCTATTATTATTAGCTTTAATAAAGAATTAAAGATAAGCTATATTATAATTTTTAATAATAAGAAGTTAGGGGTAGGTATAGTAA--------AGGAGGA : 2755
cen2-CR4 : ---------------------------------------------------------------------------------------------------- :    -
cen2-CR5 : GCTATTATTATTAGCTTTAATAAAAAATTAAAAAAAAGTTATATTATAATTTTTAATAATAAGAAGTTAAAAGTAAGTATAGTAAAAAAAAAAAAAAAAAA : 1104
cen3-CR1 : CCTATTATTATTAGCTTTAATAAAAAATTAAAAATAAGCTATATTATAATTTTTAATAATAAAAAGTTAAAGGTAGGTATAATAA--------AGAAGGA : 1190
cen3-CR2 : CTTATTATTATTAGCTTTAATAAAGAATTAAAAAAAAGCTATATTATAATTTTTAATAGTAAAGAGTTAAAAGTAGGTATAGTAA--------AGGAGGA : 2748
cen3-CR3 : CTTATTATTATTAGCTTTAATAAAAAAGTAAAAATAAGCTATATTATAATTTTTAATAATAAAAAGTTAAAGGTAAGTATAATAA--------AAAAGGA : 2749
cen3-CR4 : CTTATTATTATTAACTTTAATAAAAAATTAAAAATAAGCTATATTATAATTTTTAATAATAAGAAGTTAGAAGTAAATATAATAA--------------- : 2667
cen4-CR1 : ---------------------------------------------------------------------------------------------------- :    -
cen4-CR2 : CCTATTATTATTAGCTTTAATAAAGAATTAAAAAAAAGCTATATTATAATTTTTAATAATAAAAAGTTAAAGGTAAGTATAATAA--------AAAAAAA : 2826
cen4-CR3 : ---------------------------------------------------------------------------------------------------- :    -
cen4-CR4 : CTTATTATTATTAGCTTTAATAAAGAATTAAAAAAAAGCTATATTATAATTTTTAATAATAAGAAGTTAAGGGTAAGTATAATAA--------AAAAGGA : 2908
cen4-CR5 : ---------------------------------------------------------------------------------------------------- :    -
cen5-CR1 : CCTATTATTATTAGCTTTAATAAAGAATTAAAGATAAGCTATATTATAATTTTTAATAATAAAGAGTTAAAGGTAGATATAATAA--------AGGAGGA : 2736
cen6-CR1 : CCTATTATTATTAACTTTAATAAAAAATTAAAAAAAAGCTATATTATAATTTTTAATAGTAAGAAGTTAAAGGTAAGTATAGTAA--------AGGAGGA : 2925
cen7-CR1 : GCTATTATTATTAACTTTAATAAAAAATTAAAAAGAAGTTATATTATAATTTTTAATAATAAGAAGTTAAAGGTAGGTATAATAA--------AAAAGGA : 2688
cen7-CR2 : CTTATTATTATTAGCTTTAATAAAAAATTAAAAATAAGCTATATTATAATTTTTAATAATAAGAAGTTAAAGATAAGTATAGTAA--------AAAAGGA : 2731
cen7-CR3 : CCTATTATTATTAGCTTTAATAAAAAATTAAAAATAAGCTATATTATAATTTTTAATAATAAGAAGTTAAAAGTAAGTATAATAA---------AAAAAA : 2677
               tattattatta ctttaataaa aattaaa a aag tatattataattttaata taa  agtta    ta  tata taa          a  a
```

```
                    *      3020         *      3040         *      3060         *      3080         *      3100
cen1-CR1 : GAAGGAGAAAAAGAAAGAGATAAAAAAAAAGAAGT------------------TAAAAAAAAAATTAAAAAAGGAAAAGATAATAAAAAA---AAAGAAG : 2831
cen1-CR2 : GGAAAAAAAGAGGGAGGAGGAGGAGATAAAGGAGG------------------------AAGAGTTAAAAAAAAAGAAAGTAAAAAAAGGG--GAGAAAA : 2941
cen1-CR3 : GGAGGAGAAGGGGGAGGAGGAGGAAATAAAAAAGG------------------------AGGAGTTAAAAAAAAAGGAGGTAAAAAAAAAG--GAGGAAG : 2986
cen1-CR4 : GGAGGAGGAGGAGGAGGAGGAGGAGGAGGAGGAGGAGGAGGAGGAGGAGGAGGAGGAGGAGGAGTTAAAGGAGGAAGAATTAAAGAAG-----GAGGAGG : 2678
cen1-CR5 : GGAGGAGGAGGAGGAGGAGGAGAAGGAGAAGAAGG---------------------AGGAGGAGTTAAAGGAGGAAGAATTAAAAGAG-----GAGGAGG : 2747
cen2-CR1 : GGAGAAAATAAAGTAAGAGAAGTTAAAAAAGAAGT------------------------------TAAAAAAAAAGAAAGTAAAAGAATAATTAAAAAAA : 1721
cen2-CR2 : GGAGGAGGAGGAGGGGGAGAAGAAAGAAATAAAAA---------------------AAAAGAAGTTAAAAAAGAAAGAAGTAAAGGAGAGG--GAGAAAA : 2994
cen2-CR3 : GGAGGAGGAGGGGGAGGAGGAGAAGATAAAAGAGG------------------------AGGAGTTAAAGGAGGAGGAGGTAAAGAAAAAA--AAGGAGA : 2829
cen2-CR4 : ---------------------------------------------------------------------------------------------------- :    -
cen2-CR5 : GAAGGAGAAAAAAGAAAAGGAGGAGATAAAGAAAA------------------------AGAAGTTAAAAAAAAAGAAATTAAAAAAC-----AAAAAGG : 1175
cen3-CR1 : GAAGAAAATAAAAAAGAAAAAGTTAAAAAAAAAAG---------------AGGTAAAGGAATAATTAAAAAGAAAGAGAAAGTAAAA-----AAAAAAG : 1270
cen3-CR2 : GGTAAAGAAGGAGGAGGAGGAGTTAAAGAAGGAGG---------------------------AATTAAAAAAGGAGGAGGTAATA--------AAGGAGG : 2813
cen3-CR3 : GGAGGAAATAAAAAAAAAGTTAAAAAAGAAAAAGGTAAAAAAATAATTAAAAAAGAAGGAGGAGATAAAAAAAAAGTAAAAAAAAAAGTAAAGAAAAAGA : 2849
cen3-CR4 : -----------AAAAAAAGAAGGTAAAAAAAAAAA---------------------------ATTAAAAAAAAAAGAAGGTAAAA--------AAAAAAG : 2721
cen4-CR1 : -------------------------------------------------------------AAGTTAAAAAAAAAGGAATTAAAAA-------------- :   25
cen4-CR2 : GAAAGAGGAGAAAAAAAAGAAGAAAATAAAAAAAA--------------------------AGAGTTAAAAAAAAGGAATTAAAAAAA-----AAGAAAA : 2895
cen4-CR3 : ---------------------------------------------------------------------------------------------------- :    -
cen4-CR4 : GGAGGAGGAGGAGGAGGAGAAGGAGATAAAAAAGG------------------------AGAAGTTAAAAAAAAAGGAATTAAAAAA------AAAAAGG : 2978
cen4-CR5 : ---------------------------------------------------------------------------------------------------- :    -
cen5-CR1 : GGAGGAGATAAAAAAGGAGGAGGAGGAGTTAGAGA------------------------AAAAGTTAAAAAAAGAGGAGGTAAAGGAGGAGGT------- : 2805
cen6-CR1 : GGAGGAAAAGAAAGAGAAGGAGGAGGAGATAAAGA------------------------AAAAGTTAAAAAAAGAAAAATTAAAAAAA-----AAGAAGG : 2996
cen7-CR1 : GGATAAGGAAAAGGAGAAGGAGAAGAAAAAGAAAA----------------TAAAAAAAAAAAAGTTAAAAAAAAAGGAATTAAAAA-------AAAAAGG : 2765
cen7-CR2 : AGAGAAAATAAAAAAAAAGAAGTTAAAAAAGAAGT-----------------------------------TAAAAAAAAATAAAAAAAAAGGAAAAAAAA : 2796
cen7-CR3 : AAAGAAAATAAAAAAAAAGAAGTTAAAAAAAAAGT---------------------TAAAAAAAAAGATAAAAAAATAATTAAAAAAAAAG--AAGAAAG : 2754
                a        a  ag             a                                  aa  a  a  a  taa           a  a

                    *      3120         *      3140         *      3160         *      3180         *      3200
cen1-CR1 : TTAAAAAAAAAGTAAAAGTAATAAT-AAAAAAAAAAATAAAAGGTAGTAAAAATAATAATAATAAGAAGTTAAAGTTTAATAGTTTTTT-AAATTAAAG- : 2928
cen1-CR2 : AGAAAAAAAAAGTAAAAGCAGTAGT-AAAAAAAAAAGTAAAAAATAATAAA---AAGAATAGTAAAAAGTTAAAGTTTAATAGTTTTTT-AAACTAAAGT : 3036
cen1-CR3 : AGAAAAAAAAGGCAGAGGCAGTAGT-AAAAAAAAAAGTAAAAAGTAGTAAA---AAAAATAATAAAAAGTTAAAGTTTAATAGTTTTTT-AAATTAAAGT : 3081
cen1-CR4 : TAATAAAGGAGGTAAAGGAGGAGGT-AAAAAAAAAGGCAGAGGGTAGTAAA---AAGGATAATAAGAAGTTAAAGTTTAATAATTTTTT-AAATTAAAG- : 2772
cen1-CR5 : TAATAAAGAAAGTAAAGGAGGAGGT-AAAAAAGAAGGTAAAAGGTAATAAA---AAGAATAGTAAAAAGTTAAAGTTTAATAGTTTTTT-AAACTAAAG- : 2841
cen2-CR1 : AAGAAAAAAAAATAAAGGCAGTAAT-AAAAAAAAAAGTAAAAAGTAATAAAAA-AATAATAATAAAAAGTTAAAGTTTAATAATTTTTT-AAATTAAGG- : 1817
cen2-CR2 : AGGAGAAGGAGGTAAAGGCAGTAGT-AAAAAAAGAGGTAAAAGGTAGTAAA---AAAAATAATAAAAAGTTAAAGTTTAATAATTTTTT-AAATTAAAGT : 3089
cen2-CR3 : AGGAGAAGGAGGTAAAAGCAGTAGT-AAAAAAGGAGGTAAAAAGTAGTAAA---AAGGATAGTAAGGAGTTAAAGTTTAATAGTTTTTT-AAACTAAAGT : 2924
cen2-CR4 : ------------TAAAAAAAAAAGT--AAAAAAAAAATAATAAATAATAAAAATAATAATAATAAAAAGTTAAAGTTTAATAGTTTTTT-AAATTATAC- :   84
cen2-CR5 : TAATAAAAAAAGTAAAAAAAAAAGT--AAAAAAAAAGTAAAAAGTAGTAAA---AAGAATAATAAAAAGTTAAAGTTTAATAGTTTTTT-AAATTAAAG- : 1268
cen3-CR1 : GAAAAGTTAAAAAAAAAAAAAAAGT-AAAAAAAAA---AAGTAATAAAAAA---AATAATAATAAAAAGTTAAAGTTTAATAATTTTTT-AAATTAAAG- : 1361
cen3-CR2 : TAGAAAAGAAAGTAAAAAAAGAGGA-GGAGGAGAAGGTAAAAAGTAGTAAA---AAGAATAGTAAGGAGTTAAAGTTTAATAGTTTTTT-AAATTAAAG- : 2907
cen3-CR3 : TAAAAAAAAAAGTAAAAAAAAAAAAATAAATAAAAAGTAAAAAGTAATAAA---AAAAATAATAAAAAGTTAAAGTTTAATAATTTTTT-AAATTAAAG- : 2944
cen3-CR4 : TAAAAAAGAAAGTAAAAAAAAAGGT-AAAAAAAAAGGTAAATTATAATAAA---AAAAATAATAAAAAGTTAAAGTTTAATAGTTTTTT-AAATTAAAG- : 2815
cen4-CR1 : ------------------------------------------------------AATAATAATAAAAAGTTAAAGTTTAATAATTTTTT-AAACTAAAG- :   69
cen4-CR2 : TAATAAAAAAAGTAAAAAAAGAGGT-AAAAAAAAAGGCAGAAAGTAATAAA---AAAAATAATAAAAAGTTAAAGTTTAATAATTTTTT-AAATTAAAG- : 2989
cen4-CR3 : ---------------------------------------------------------------------------------------------------- :    -
cen4-CR4 : TAATAAAAAAAATAAAAAAAAAAGT-AAAAAAAAAAGTAAAAAATAATAAA---AAAGATAATAAAAAGTTAAAGTTTAATAATTTTTT-AAATTAAAG- : 3072
cen4-CR5 : ---------------------------------------------------------------------------------------------------- :    -
cen5-CR1 : --------------------------AGAGGAGGAGGTAAAAGGTAATAAA---AAGAATAATAAGGAGTTAGAGTTTAATAGTTTTTT-AAATTAAAG- : 2874
cen6-CR1 : TAATAAAAAAAGTAAAAAAAAAAATAAAAAAAAAAGGCAAAAAGTAATAAA---AAGAATAATAAAAAGTTAAAATTTAATAGTTTTTT-AAATTAAAGT : 3092
cen7-CR1 : TAATAAAAAAAGTAAAAAAAAAGGT--AAAAAAAAGGTAAAAAGTAATAAA---AAAAATAATAAAAAGTTAAAGTTTAATAATTTTTTTAAATTAAAG- : 2859
cen7-CR2 : AAATAAAAAAAAAATAAAAAAAAAT--AAAAAAAAAATAAAAAATAATAAAA--AAAAATAATAAAAAGTTAAAGTTTAATAGTTTTTT-AAATTAAAG- : 2890
cen7-CR3 : TAAAAATAAAAAATAAAAAAAAAAT-AATAAAAAAAGTAAAAAGTAATAAA---AAAAATAATAAAAAGTTAAAGTTTAATAGTTTTTT-AAATTAAAG- : 2848
                    a     a           aaa a  a   aaa   ta taaa   aa aata taa aagttaaagtttaata tttttt aaa taaag
```

```
                   *        3220          *        3240          *        3260          *        3280          *        3300
cen1-CR1 : TTTTAATATTTTTAAGTATATTTTT----TTTTTTTAATATATAATTAAATAATATATATTTTTATTATATTATTTTTATTATAATATTTAGTAATATTA : 3024
cen1-CR2 : TTTTAATATTTTTAAGTATATCTCC--T-TTTTTTTAATATATAATTAAATAATATATACTTTTATTATACTATTTTTATTATAATATTTAATAATATTA : 3133
cen1-CR3 : TTTTAATATTTTTAAATATATCTCC--T-TTTTTTTAATATATAATTAAATAATATATGCTTTTATTATACTATTTTTATTATAATATTTAGTAATATTA : 3178
cen1-CR4 : TTTTAGTATTTTTAAGTATATCTCC--TCTTTTTTTAATATATAATTAAATAATATATACTTTTATTATACTATTCTTATTATAATATTTAGTAATATTA : 2870
cen1-CR5 : TTTTAATATTTTTAAATATATCTTC--T-CTTTTTTAATATATAATTAAATAATATATACTTTTATTATACTATCTTTATTATAATATTTAATAATATTA : 2938
cen2-CR1 : TTTTAATATTTTTAAGTATATATT-----TTTTTTTAATATATAATTAAATAATATATGCTTTTATTATATTATTTTTATTATAATATTTAGTAATATTA : 1912
cen2-CR2 : TTTTAGTATTTTTAAATATATCTTC----TCTTTTTAATATATAATTAAATAATATATACTTTTATTATATTATTCTTATTATAATATTTAGTAATATTA : 3185
cen2-CR3 : TTTTAATATTTCTAGGTATATCTCC----TCTTTTTAATATATAATTAAATAATATATGCTTTTATTATGCTGTCTTTATTATAATATTTAGTAATATTA : 3020
cen2-CR4 : TTTTAGTATTTTTATATATATCT------TTTTTTTAATATATAATTAAATAATATATACTTTTATTATATTATTTTTATTATAATATTTAGTAATATTA :  178
cen2-CR5 : TTTTAGTATTTTTAAATATATTTTCTTTTTTTTTTTAATATATAATTAAATAATATATACTTTTATTATACTATTTTTATTATAATATTTAGTAATATTA : 1368
cen3-CR1 : TTTTAATATTTTTAAGTATA---------TTTTTTTAATATATAATTAAATAATATATACTTTTATTATATTATTTTTATTATAATATTTAATAATATTA : 1452
cen3-CR2 : TTTTAATATCTTTATATATATCTCC--T-CTTTTTTAATATATAATTAAATAATATATACTTTTATTATACTATTCTTATTATAATATTTAGTAATATTA : 3004
cen3-CR3 : TTTTAATATTTTTAAGTATATTTTC--T-TTTTTTTAATATATAATTAAATAATATATACTTTTATTATATTATTTTTATTATAATAATTAGTAATATTA : 3041
cen3-CR4 : TTTTAGTATTTTTAAGTATATT-------TTTTTTTAATATATAATAAAATAATATATACTTTTATTATATTATTTTTATTATAATATTTAGTAATATTA : 2908
cen4-CR1 : TTTTAGTATTTTTAAGTATATCTCC--T-TTTTTTTAATATATAATTAAATAATATATACTTTTATTATACTATCCTTATTATAATATTTAGTAATATTA :  166
cen4-CR2 : TTTTAGTATTTTTAAGTATATCTCC--T-TTTTTTTAATATATAATTAAATAATATATACTTTTATTATACTATTTTTATTATAATATTTAATAATATTA : 3086
cen4-CR3 : ---------------------------------------------------------------------------------------------------- :    -
cen4-CR4 : TTTTAATATTTTTAAGTATATCTCC--T-TTTTTTTAATATATAATTAAATAATATATATTTTTATTATGCTATTTTTATTATAATATTTAGTAATATTA : 3169
cen4-CR5 : ---------------------------------------------------------------------------------------------------- :    -
cen5-CR1 : TTTTAGTATTTTTAAGTATATCTCCTTT-TTTTTTTTAAATATAATTAAATAATATATACTTTTATTATACTATTCTTATTATAATATTTAGTAATATTA : 2973
cen6-CR1 : TTTTAATATTTTTAAATATACCTTC----TTTTTTTAATATATAATTAAATAATATATACTTTTATTATACTATTTTTATTATAATATTTAGTAATATTA : 3188
cen7-CR1 : TTTTAGTATTTTTAAATATATTTCT--T-TTTTTTTAATATATTATTAAATAATATATACTTTTATTATATTATTTTTATTATAATATTTAATAATATTA : 2956
cen7-CR2 : TTTTAATATTTTTAAGTATAT--------TTTTTTTAATATATAATTAAATAATATATACTTTTATTATATTATTTTTATTATAATATTTAATAATATTA : 2982
cen7-CR3 : TTTTAATATTTTTA----------C--T-TTTTTTTAATATATAATTAAATAATATATACTTTTACTATATTATTTTTATTATAATACTTAGTAATATTA : 2935
           tttta tattttta  tatat        tttttttaatatataattaaataatatatacttttattata tatt ttattataatattta taatatta

                   *        3320          *        3340          *        3360          *        3380          *        3400
cen1-CR1 : GCTTTATTTAATTATATTTTTATATTTTTTAATTATAGTATTATTTATAAAGTTTAAATATTTTAATTATTAATTATTTTTATTTAAGTAGTTTTTTTAT : 3124
cen1-CR2 : GCTTTATTTAATTATATCTTTATATTCCTTAATTATAATATTATTTATAAAGTCTAAATATTTTAACTATTAATTATTTTTATTTAAGTAGTCTTTTTAT : 3233
cen1-CR3 : GCTTTATTTAATTATATTTTTATATTCTTTAATTATAATATTATTTATAAAGTCTAAATATTTTAATTATTAATTATTTTTATTTAAGTAGTCTTTTTAT : 3278
cen1-CR4 : GCTTTATTTAATTATATCTTTATATTCCTTAATTATAGTGTTATTTATAAAGTCTAAATATTTTAACTATTAATTATCCTTATTTAAATAGTCTTTTTAT : 2970
cen1-CR5 : GCTTTATTTAATTATATCTTTATATTTCTTAATTATAATATTATTTATAAAGTCTAAATATTTTAATTATTAATTATTCTTATTTAAGTAGTCTTTTTAT : 3038
cen2-CR1 : GCTTTATTTAATTATATTTTTATATTTTTTAATTATAGTATTATTTATAAAGTCTAAATATTTTAATTATTAATTATTTTTATTTAAATAGTTTTTTTAT : 2012
cen2-CR2 : GCTTTATTTAATTATATTTTTATATTCCTTAATTATAGTATTATTTATAAAGTTTAAATATTTTAACTATTAATTATTTTTATTTAAGTAGTTTTTTTAT : 3285
cen2-CR3 : GCTTTATTTAATTATATCTTTATATTCCTTAATTATAGTATTATTTATAAAGTCTAAATATTTTAACTATTAATTATCCTTATTTAGGTAGTCTTTTTAT : 3120
cen2-CR4 : GCTTTATTTAATTATATTTTTATATTTTTTAATTATAATATTATTTATAAAGTTTAAATATTTTAATTATTAATTATTCTTATTTAAATAGTTTTTTTAT :  278
cen2-CR5 : GCTTTATTTAATTATATTTTTATATTTTTTAATTATAATATTATTTATAAAGTTTAAATATTTTAATTATTAATTATCCTTATTTAAGTAGTTTTTTTAT : 1468
cen3-CR1 : GCTTTATTTAATTATATTTTTATATTCTTTAATTATAATATTATTTATAAAGTTTAAATATTTTAATTATTAATTATTTTTATTTAAGTAGTCTTTTTAT : 1552
cen3-CR2 : GCTTTAGTTAATTATATTTTTATATTCCTTAATTATAATATTATTTATAAAGTCTAAGTATTTTAATTATTAATTATTTTTATTTAAGTAGTCTTTTTAT : 3104
cen3-CR3 : ACTTTATTTAATTATATTTTTATATTTTTTAATTATAATATTATTTATAATATTTAAATATTATAATTATTAATTATTTTTATTTAAATAGTCTTTTTAT : 3141
cen3-CR4 : ACTTTATTTAATTATATTTTTATATTTTTTAATTATAATATTATTTATAAAGTCTAAATATTTTAATTATTAATTATTTTTATTTAAGTAGTTTTTTTAT : 3008
cen4-CR1 : GCTTTATTTAATTATATCTTTATATTCCTTAATTATAGTATTATTTATAAAGTCTAAATATTTTAATTATTAATTATTTTTATTTAAGTAGTCTTTTTAT :  266
cen4-CR2 : GCTTTATTTAATTATATTTTTATATTCTTTAATTATAATATTATTTATAAAGTCTAAATATTTTAATTATTAATTATTTTTATTTAAGTAGTTTTTTTAT : 3186
cen4-CR3 : ---------------------------------------------------------------------------------------------------- :    -
cen4-CR4 : GCTTTATTTAATTATATTTTTATATTCCTTAATTATAATATTATTTATAAAGTTTAAATATTTTAACTATTAATTATTTTTATTTAAATAGTCTTTTTAT : 3269
cen4-CR5 : ---------------------------------------------------------------------------------------------------- :    -
cen5-CR1 : GCTTTATTTAATTATATTTTTATATTCTTTAATTATAGTATTATTTATAAAGTCTAAATATTTTAACTATTAATTATCCTTATTTAAGTAGTTATTTTAT : 3073
cen6-CR1 : GCTTTATTTAATTATATTTTTATATTTCTTAATTATAGTATTATTTATAAAGTTTAAATATTTTAACTATTAATTATCCTTATTTAAGTAGTCTTTTTAT : 3288
cen7-CR1 : GCTTTATTTAATTATATTTTTATATTTTTTAATTATAGTATTATTTATAAAGTCTAAATATTTTAATTATTAATTATTTTTATTTAAGTAGTCTTTTTAT : 3056
cen7-CR2 : GCTTTATTTAATTATATTTTTATATTTTTTAATTATAATATTATTTATAAAATTTAAATATTTTAACTATTAATTATTTTTATTTAAGTAGTTTTTTTAT : 3082
cen7-CR3 : ACTTTATTTAATTATATTTTTATATTTTTTAATTATAATATTATTTATAAAGTTTAAATATTTTAATTATTAATTATTTTTATTTAAGTAGTTTTTTTAT : 3035
           gctttatttaattatat tttatatt  ttaattata tattatttataaagt taaatattttaa tattaattat  ttatttaa tagt tttttat
```

```
                         *    3420          *     3440          *     3460          *     3480          *     3500
cen1-CR1 : TTAACTAAGTATAGCTATTATTTACCTTAATATTTATATATAAAAATATATTTAACTTATATATTTCTTTTAATATAAAAGGATAATTTATTAATTTAA : 3224
cen1-CR2 : TTAACTAAGTAAAGCTGCTATTTACTTTAATACTTATATATAAAAATATATTTAACTTATATATTTCCTTTAATATAAAGGGATAATTTATTAATTTAA : 3333
cen1-CR3 : TTAACTAAGTAAAGCTGCTATTTACCTTAATACTTATATATAAAAATATATTTAACTTATATATTTCCTTTAATATAAAAGGATAATTTATTAATTTAA : 3378
cen1-CR4 : TTAACTAAGTAAAGCTGCTATTTACCTTAATACTTATATATAAAAATGCATTTAACTTATATATTTCCTTTAATATAAAGGGATAATTTATTAGTTTAA : 3070
cen1-CR5 : TTAACTAAGTAAAGCTGCTATTTACCTTAATACTTATATATAAAAATATATTTAACTTATATATTTCCTTTAATATAAAGGGATAATCTATTAGTTTAA : 3138
cen2-CR1 : TTAATTAAGTAAAGCTACTATTTATTTTAATACTTATATATAAAAATATATTTAACTTATATATTTCCTTTAATATAAAAAAATAATTTATTAATTTTA : 2112
cen2-CR2 : TTAACTAAGTAAAGCTACTATTTACCTTAATACTTATATATAAGAATATATTTAACTTATATATTTCCTTTAATATAAAGGGATAATTTATTAATTTAA : 3385
cen2-CR3 : TTAACTAAGTAAAGCTGCTATTTACCTTAATACTTATATATAAGAATACATTTAACTTATATATTTCCTCTAGTATAAAAGGATAATCTATTAGTTTAA : 3220
cen2-CR4 : TTAATTAAGTAAAGCTATTATTTACTTTAATACTTATATATAAAAATATATTTAACTTATATATTTCTTTTAATATAAAAGAATAATTTATTAAGTTAA :  378
cen2-CR5 : TTAATTAAGTAAAGCTACTATTTATATTAATACTTATATATAAGAATATATTTAACTTATATATTTCCTTTAATATAAAAGGATAATTTATTAATTTAA : 1568
cen3-CR1 : TTAATTAAGTAAAGCTATTATTTACCTTAATACTTATATATAAAAATATATTTAACTTATATATTTCCTTTAATATAAAAGGATAATTTATTAATTTAA : 1652
cen3-CR2 : TTAACTAAGTAAAGCTGCTATTTACCTTAATACTTATATATAAGAATATATTTAACTTATATATTTCCTTTAATATAAAAGGATAATTTATTAATTTAA : 3204
cen3-CR3 : TTAACTAAGTAAAGCTGCTATTTACTTTAATACTTATATATAAGAATATATTTAATTTTATATATTTCTTTTAATATAAAAAAATAATTTATTAATTTAA : 3241
cen3-CR4 : TTAACTAAATAAAGGTATTATTTACTTTAATATTTATATATAAAAATATATTTAACTTATATATTTTTTTTAATATAAAGAGATAATTTATTAATTTAA : 3108
cen4-CR1 : TTAATTAAGTAAAGCTGCTATTTACCTTAATACTTATATATAAAAATGCATTTAACTTATATATTTCCTTTAATATAAAAGGATAATTTATTAATTTAA :  366
cen4-CR2 : TTAACTAAGTAAAGCTACTATTTACTTTAATACTTATATATAAAAATATATTTAACTTATATATTTCCTTTAATATAAAAGGATAATTTATTAATTTAA : 3286
cen4-CR3 : --------------------------------------------------------------------------------------------------- :    -
cen4-CR4 : TTAATTAAGTAAAGCTACTATTTACTTTAATACTTATATATAAGAATATATTTAACTTATATATTTCCTTTAGTATAAAAGGATAATTTATTAATTTAA : 3369
cen4-CR5 : --------------------------------------------------------------------------------------------------- :    -
cen5-CR1 : TTAACTAAATAAAGCTGCTATTTACTTTAATACTTATATATAAAAATGCATTTAACTTATATATTTCCTTTAATATAAAAGAATAATCTATTAATTTAA : 3173
cen6-CR1 : TTAATTAAGTAAAGCTGCTATTTACTTTTATATTTATATATAAAAATATATTTAACTTATATATTTCCTTTAATATAAAAGAATAATTTATTAATTTAA : 3388
cen7-CR1 : TTAATTAAGTAAAGCTGCTATTTACTTTAATACTTATATATAAAAATATATTTAACTTATATATTTCCTTTAATATAAAAGGATAATTTATTAATTTAA : 3156
cen7-CR2 : TTAATTAAGTAAAGCTATTATTTACTTTAATACTTATATATAAAAATATATTTAACTTATATATTTCCTTTAGTATAAAAAAATAATTTATTAATTTAA : 3182
cen7-CR3 : TTTATTAAATAAAAATGCTATTTACTTTAATACTTATATATAAAAATATATTTAACTTATATATTTCCTTTAGTATAAAAGAATAATTTATTAATTTAA : 3135
           ttaa taagtaaagct  tatttac ttaatacttatatataa aata atttaacttatatatttc ttta tataaa   ataatttattaatttaa

                         *    3520          *     3540          *     3560          *     3580          *     3600
cen1-CR1 : ATAAATATTTATTTTTATTATAGTATTTTTTAAAATTAATAATATTAAAAATATTATAAATATAAAGTAATAATAGTAAGTATAATTAATATACTAGTTT : 3324
cen1-CR2 : ATAAATATTTATCTTTACTATAGTATTTTTTAAGGTTAGTAATATTAAAAATATTATAAATATAAAGTAATAATAATAAGTGTAATTAATATACTAGTTT : 3433
cen1-CR3 : ATAAATATTTATTTTTATTATAGTATTTTTTAAGGTTAGTAATATTAAAAATATTATAAATATAAAGTAATAATAATAAGTATAATTAATATGCTAGTTT : 3478
cen1-CR4 : ATAAATATCTATTTTTACTATAGTATTCTTTAAGGTTAATAATATTAAAAATATTATAAATATAAAGTAATAATAATAAGTATAATTAATATACTAGTTT : 3170
cen1-CR5 : ATAAATATTTATTTTTACTATAGTATTTTTTAAGGTTAGTAATATTAAAAATATTATAAATATAAAGTAATAATAGTAAGTATAATTAATATATTAGTTT : 3238
cen2-CR1 : ATAAATACTTATTTTTACTATAGTATTTTTTAAAATTAGTAATATTAAAAATATTATAAATATAAAGTAATAATAATAAGTATAATTAATATATTAATTT : 2212
cen2-CR2 : ATTAATATTTATTTTTACTATAGTATTTTTTAAGGTTAATAATATTAAAAATATTATAAATATAAAGTAATAATAGTAAGTATAATTAATATACTAGTTT : 3485
cen2-CR3 : ATAAATATTTATTTTTACTATAGTATTTTTTAAGGTTAATAATATTAAAAATATTATAAATATAAAGTAATAATAGTAAGTATAATTAATATGCTAGTCT : 3320
cen2-CR4 : ATAAATATTTATTTTTATTATAATATTTTTTAAAATTAGTAATATTAAAAATATTATAAATATAAAGTAATAATAATAAATATAATTAATATACTAGTTT :  478
cen2-CR5 : ATAAATATTTATTTTTATTATAATATTTTTTAAAGTTAGTAATATTAAAAATATTATAAATATAAAGTAATAATAGTAAGTATAATTAATATATTAATTT : 1668
cen3-CR1 : ATAAATATTTATTTTTATTATAGTATTTTTTAAAGTTAGTAATATTAAAAATATTATAAATATAAAGTAATAATAATAAGTATAATTAATATATTAATTT : 1752
cen3-CR2 : ATAAATATTTATTTTTATTATAGTATTTTTTAAAGTTAGTAATATTAAAAATATTATAAATATAAAGTAATAATAATAAGTGTAATTAATATACTAGTCT : 3304
cen3-CR3 : ATAAATATTTATTTTTATTATAATATTTTTTAAAATTAGTAATATTAAAAATATTATAAATATAAAGTAATAATAATAAGTATAATTAATATACTAGTTT : 3341
cen3-CR4 : ATAAATATTTATTTTTATTATAATATTTTTTAAAATTAGTAATATTAAAAATATTATAAATATAAAGTAATAATAATAAATATAATTAATATATTAATTT : 3208
cen4-CR1 : ATAGCTATTTATTTTTATTATAGTATTTTTTAAAGTTAATAATATTAAAAATATTATAAATATAAAGTAATAATAATAAGTATAATTAATATACTAGTTT :  466
cen4-CR2 : ATAAATATTTATTTTTACTATAGTATTTTTTAAGGTTAATAATATTAAAAATATTATAAATATAAAGTAATAATAATAAGTATAATTAATATACTAGTTT : 3386
cen4-CR3 : --------------------------------------------------------------------------------------------------- :    -
cen4-CR4 : ATAAATATTTATCTTTATTATAGTATTTTTTAAGGTTAGTAATATTAAAAATATTATAAATATAAAGTAATAATAATAAGTATAATTAATATATTAATTT : 3469
cen4-CR5 : ----------------------------------------------------------------------------------------ATATACTAGTTT :   96
cen5-CR1 : ATAAATATTTATTTTTACTATAGTATTTTTTAAAATTAGTAATATTAAAAATATTATAAATATAAAGTAATAATAGTAAGTATAATTAATATACTAGTTT : 3273
cen6-CR1 : ATAAATGCCTATTTTTATTATAGTATTTTTTAAGGTTAGTAATATTAAAAATATTATAAATATAAAGTAATAATAATAAGTATAATTAATATATTAGTTT : 3488
cen7-CR1 : ATAAATATTTATTTTTATTATAATATTTTTTAAAGTTAGTAATATTAAAAATATTATAAATATAAAGTAATAATAATAAGTATAATTAATATATTAATTT : 3256
cen7-CR2 : ATAAATATTTATTTTTATTATAGTATTTTTTAAAGTTAGTAATATTAAAAATATTATAAATATAAAGTAATAATAATAAGTATAATTAATATACTAGTTT : 3282
cen7-CR3 : ATAAATATTTATTTTTATTATAGTATTTTTTAAAGTTAGTAATATTAAAAATATTATAAATATAAAGTAATAATAATAAGTATAATTAATATACTAGTTT : 3235
           ataaatatttattttta tata tattttttaa  tta taatattaaaaatattataaatataaagtaataata taagtataattaatata ta ttt
```

```
                          *       3620         *       3640         *       3660         *       3680         *       3700
cen1-CR1 : ATTAAT-ATTAATAA--TCTTTTTAATTTAATATAATTTTATATATTTTTTATATTAGCTTATAAATTAAATAGTATAAGTTTAAGTACTTAATAAAAAT : 3421
cen1-CR2 : ATTAAT-ACTAATAA--TCTTCTTAATTTAATATAGTTTTATATA-TTTTTATACTAGCTTATAAGTTAAGCAGTATAGGTTTAAGTACTTAATAGAAAT : 3529
cen1-CR3 : ATTAAT-ACTAATAA--TCTTTTTAATTTAATATAGTTTTATATA-TTTTTATACTAGCTTATAAGTTAGGCAGTATAGGTTTAAGTGCTTAGTAAAAAT : 3574
cen1-CR4 : ACTAGT-ACTAATAA--TCTTCTTAATTTAATATAGTTTTATATA-TTTTTATACTAGCTTATAAGTTAGGCAGTATAGGTTTAAGTGCTTAGTAGAAAT : 3266
cen1-CR5 : ACTAAT-ACTAATAA--TCTTTTTAATTTAATATAGTCTTATATA-TTTTTATACTAGCTTATAAATTAGGCAGTATAGGTTTAAGTGCTTAATAAAAAT : 3334
cen2-CR1 : ATTAAT-ATTAATAA--TCTTTTTAATTTAATATAGTTTTATATA-TTTTTATATTAGCTAATAAAGTAAGTAGTAAAAGTTTAAGTGCTTAATAAAAAT : 2308
cen2-CR2 : ATTAAT-ATTAATAA--TTTTCTTAATTTAATATAATTTTATATA-TTTTTATATTAGCTTATAAGTTAGGTAGTATAAATTTAAGTGCTTAATAAAAAT : 3581
cen2-CR3 : ACTAGT-GCTAATAA--TCTTCTTAATTTAATATAGTCTTATATA-TTTTTATACTAGTTTATAAGTTAAGTAGTGCAGATTTAAGTACTTAATAAAAAT : 3416
cen2-CR4 : ATTAAT-ATTAATAA--TTTTTTTAATTTAATATAATTTTATATA-TTTTTATACTAGCTTATAAGTTAAGTAATATAAATTTAAATACTTAATAAAAAT :  574
cen2-CR5 : ACTAAT-ATTAATAA--TTTTCTTAATTTAATATAGTTTTATATA-TTTTTATACTAGCTTATAAATTAAGTAGTATAAGTTTAAGTGCTTAATAAAAAT : 1764
cen3-CR1 : ATTAAT-ATTAGTAA--TCTTTTTAATTTAATATAGTTTTATATA-TTTTTATATTAGCTTATAAATTAAGCAGTATAAGTTTAAATACTTAATAAAAAT : 1848
cen3-CR2 : ACTAGT-ACTAATAA--TTTTCTTAATTTAATATAGTTTTATATA-TTTTTATACTAGCTTATAAGTTAAGTAATATAGGTTTAAGTGCTTAGTAAAAAT : 3400
cen3-CR3 : ATTAATAATTAATTA--TCTTTTTAATTTAATATAGTTTTATATA-TTTTTATACTAGTTTATAAATTAAATAGTAAAGGTTTAAGTATTTAATAAAAAT : 3438
cen3-CR4 : ACTAAT-ATTAATAA--TCTTTTTAATTTAATATAATTTTATATA-TTTTTATACTAGCTTATAAGTTAAGTAGTATAAGTTTAAGTGCTTAATAAAAAT : 3304
cen4-CR1 : ATTAAT-ACTAATAA--TCTTTTTAATTTAATATAGTTTTATATA-TTTTTATACTAGCTTATAAGTTAAATAGTATAGGTTTAAGTGCTTAATAAAAAT :  562
cen4-CR2 : ATTAAT-ATTAATAA--TCTTTTTAATTTAATATAGTTTTATATA-TTTTTATATTAGCTTATAAATTAGGCAGTATAGGTTTAAGTGCTTAATAAAAAT : 3482
cen4-CR3 : --------------------------------------------------------------------------------------TGCTTAATAAAAAT :   14
cen4-CR4 : ATTAAT-ATTAATAA--TCTTTTTAATTTAATATAGTTTTATATA-TTTTTATATTAGCTTATAAATTAAGTAGTATAAGTTTAAGTGCTTAGTAAAAAT : 3565
cen4-CR5 : ATTAAT-ATTAATAAATTTTTTTAATTTAATATAGTTTTATATA-TTTTTATATTAGTTTATAAATTAAGTAGTATAAGTTTAAGTACTTAATAAAAAT :  194
cen5-CR1 : ATTAAT-ACTAATAA--TTTTCTTAATTTAATATAATCTTATATA-TTTTTATACTAGCTTATAAATTAGGCAGTATAGGTTTAAGTACTTAGTAAAAAT : 3369
cen6-CR1 : ATTAAT-ATTAATAA--TCTTTTTAATTTAATATAATTTTATATA-TTTTTATATTAGCTTATAAGTTAAGTAGTATAGGTTTAAGTACTTAATAAAAAT : 3584
cen7-CR1 : ACTAAT-ATTAATAA--TTTTTTTAATTTAATATAATTTTATATA-TTTTTATACTAGCTTATAAATTAAATAATATAGGTTTAAGTACTTAGTAAAAAT : 3352
cen7-CR2 : ATTAAT-ATTAATAA--TTTTCTTAATTTAATATAATTATATATA-TTTTTATACTAGCTTATAAATTAAGTAATATAAATTTAAGTACTTAATAAAAAT : 3378
cen7-CR3 : ATTAAT-ATTAATAA--TTTTCTTAATTTAATATAATTATATATA-TTTTTATACTAGCTTATAAATTAAGTAATATAAATTTAAGTACTTAATAAAAAT : 3331
           a taat a taataa  t tt ttaatttaatata ttttatata tttttata tagcttataa tta g a tata  tttaagT cTTA TAaAAAT

                          *       3720         *       3740         *       3760         *       3780         *       3800
cen1-CR1 : AAGCATTTAATTATTAATATAAAAGGTTTTTTAAATTTATTTTATATTATATTATTTATTATATATAATAATATTATTTTATAATATTTTTATAGTATTT : 3521
cen1-CR2 : AAGTACTTAATTACTAATATAAAAGGTCTTTTAAGTTTATTTTATATTATACTATTTATTATATATAGTAATGCTATTTTATAATATTTTTATAGTATTT : 3629
cen1-CR3 : AAGCACTTAATTATTAATATAAAAGGTTTTTTAAGTTTATTTTATATTATATTATTTATTATATATAATAATACTATTTTATAATATCTTTATAGTATTT : 3674
cen1-CR4 : AAGCACCTAATTACTAATATAAAAGGTTTTTTAAGTTTATTTTATATTATACTATTTATTATATATAGTAATACTATTTTATAATATTTTTATAATATCC : 3366
cen1-CR5 : AAGTACTTAATTACTAATATAAAAGGTTTTTTAAGTTTATTTTATATTATATTATTTATTATATATAGTAATGCTATTTTATAATATTTTTATAGTATTT : 3434
cen2-CR1 : AAAAACCTAATTATTAATATAAAAGGTTTTTTAAATTTATTTTATATTATATTATTTATTATATATAGTAATATTATTTTATAATATTTTTATAATATTT : 2408
cen2-CR2 : AAGCATTTAATTATTAATATAAAAGGTTTTTTAAGTTTATTTTATATTATATTATTTATTATATATAGTAATACTATTTTATAATATTTTTATAGTATTT : 3681
cen2-CR3 : AAGCACTTAATTATTAATATAAAAGGTTTTTTAAGTTTATTTTATATTATACTATTTATTATATATAGTAATACTATTTTATAATATTTTTATAGTATTT : 3516
cen2-CR4 : AAGTATTTAATTATTAATATAAAAGGTTTTTTAAGTTTACTTTATATTATATTATTTATTATATATAGTAATATTAATTTATAATATTTTTATAGCTTTT :  674
cen2-CR5 : AAGTATTTAATTATTAATATAAAAGGTCTTTTAAGTTTATTTTATATTATATTATTTATTATATATAATAATACTATTTTATAATATTTTTATAATATTT : 1864
cen3-CR1 : AAGCACTTAATTATTAATATAAAAGGTTTTTTAAGTTTATTTTATATTATATTATTTATTATATATAATAATACTATTTTATAATATTTTTATAATATTT : 1948
cen3-CR2 : AAGCACTTAATTACTAATATAAAAGGTCTTTTAAGTTTATTTTATATTATATTATTTATTATATATAGTAATACTATTTTATAATATCTTTATAATATTT : 3500
cen3-CR3 : AAGAGTTTAATTATTAATATAAAAGGTTTTTTAAATTTATTTTATATTATATTATTTATTATATATAATAATATTATTTTATAATATTTTTATAGTATTT : 3538
cen3-CR4 : AAGCACTTAATTATTAATATAAAAGGTCTTTTAAGTTTATTTTATATTATATTATTTATTATATATAATAATACTATTTTATAATATTTTTATAATATTT : 3404
cen4-CR1 : AAGCATTTAATTATTAATATAAAAGGTTTTTTAAGTTTATTTTATATTATATTATTTATTATATATAATAATACTATTTTATAATATCTTTATAGTATTT :  662
cen4-CR2 : AAATACTTAATTACTAATATAAAAGGTTTTTTAAGTTTATTTTATATTATATTATTTATTATATATAATAATATTATTTTATAATATTTTTATAGTATTT : 3582
cen4-CR3 : AAGTATTTAATTATTAATATAAAAGGTTTTTTAAGTTTGCCTTATATTATATTATTTATTATATATAGTAATACTATTTTATAATATTTTTATAATATTT :  114
cen4-CR4 : AAGCACTTAATTATTAATATAAAAGGTTTTTTAAGTTTATTTTATATTATACTATTTATTATATATAATAATATTATTTTATAATATTTTTATAGTATTT : 3665
cen4-CR5 : AAGTATTTAATTATTAATATAAAAGGTTTTTTAAATTTACTTTATATTATATTATTTATTATATATAGTAATATTATTTTATAATATTTTTATAATATTT :  294
cen5-CR1 : AAGCACTTAATTATTAATAAAAAAGGTTTTTTAAGTTTATTTTATATTATACTATTTATTATATATAGTAATACTATTTTATAATATTTTTATAGCATTT : 3469
cen6-CR1 : AAGCACTTAATTACTAATATAAAAGGTTTTTTAAGTTTATTTTATATTATATTATTTATTATATATAGTAATACTATTTTATAATATTTTTATAATATTT : 3684
cen7-CR1 : AAGTATTTAATTATTAATATAAAAGGTCTTTTAAGTTTATTTTATATTATATTATTTATTATATATAGTAATATTATTTTATAATATTTTTATAATATTT : 3452
cen7-CR2 : AAGTATTTAATTATTAATATAAAAGGTTTTTTAAGTTTATTTTATATTATACTATTTATTATATATAATAATATTATTTTATAAT-TTTTTATAATATTT : 3477
cen7-CR3 : AAGTATTTAATTACTAATATAAAAGGTTTTTTAAGTTTATTTTATATTATACTATTTATTATATATAATATTATTATTTTATAAT-TTTTTATAATATTT : 3430
           AAg a tTAATTA TAATAtAAAAGGTtTTTTAAgTTTattTTATATTATA TATTTATTATATATA TAaTa TAtTTTATAATaTtTTTATA taTtt
```

```
                                  *         3820           *         3840           *         3860           *         3880           *         3900
cen1-CR1 : TTATAAATATATTATATTTACTAAACTTATTTAATTATAAAAGCGCTTATATATTTAACTTAGCTTTAAAAAAGTCTAAAAGCTTTTAAATTATATTTAA : 3621
cen1-CR2 : TTATAAGTATACTATATTTACTAGACTTATTTAATTACAGAAGCGCTTATATATTTAACTTAACTTTAAAGAAGTCTAAAAGCTTTTAAATTATATTTAA : 3729
cen1-CR3 : TTATAAGTATATTATATTTACTAAACTTATTTAATTATAAAAGCGCTTATATATTTAACTTAACTTTAAAAAAGTTTAAAAGCTTTTAAATTATATTTAA : 3774
cen1-CR4 : TTATAAGTATACTGCATTTACTAGACTTATTTAATTATAAAAGCATTTATATATTTAACTTAGCTTTAAAGAAGTCTAGGAGCTTTTAAATTATATTTAA : 3466
cen1-CR5 : TTATAAGTATACTGCATTTACTAAACTTATTTAATTATAAAAGCACTTATATATTTAACTTAGCTTTAAAAAAGTCTAAAAGCTTTTAAATTATATTTAA : 3534
cen2-CR1 : TTATAAATTATTTATATTTACTAAACTTATTTAATTATAAAAGCGCTTATATATTTAACTTAGCTTTAAAAAAGTTTAAAAGCTTTTAAGTTATATTTAA : 2508
cen2-CR2 : TTATAAGTATATTATATTTACTAGACTTATTTAATTATAAAAGCGCTTATATATTTAACTTAACTTTAAAAAAGTTTAAAAGCTTTTAAATTATATTTAA : 3781
cen2-CR3 : TTATAAGTATACTGCATTTACTAAACTTATTTAATTATAAAAGTGCTTATATATTTAACTTAACTTTAAAGAAGTCTAGGAGCTTTTAAATTATATTTAA : 3616
cen2-CR4 : TTATAAATATATTATATTTATTAAACTTATTTAATTATAAAAGTATTTATATATTTAACTTAGCTTTAAAATAGTTTAAAAGCTTTAAAATTATATTTAA :  774
cen2-CR5 : TTATAAATATATTATATTTACTAAACTTATTTAATTATAAAAGTGCTTATATATTTAACTTAGCTTTAAAAAAGTTTAAAAGCTTTTAAATTATATTTAA : 1964
cen3-CR1 : TTATAAGTATATTATATTTACTAAACTTATTTAATTATAAAAGCACTTATATATTTAACTTAGCTTTAAAAAAGTTTAAAAGCTTTTAAGTTATATTTAA : 2048
cen3-CR2 : TTATAAGTATATTATATTTACTAAACTTATTTAATTATAAAAGCGCTTATATATTTAACTTAGCTTTAAAGAAGTTTAAAAGCTTTTAGATTATATTTAA : 3600
cen3-CR3 : TTATAAATTATTTATATTTATTAAACTTATTTAATTATAAAAGTAATTATATATTTAACTTAGCTTTAAAAAAGTTTAAAAGCTTTTAGGTTATAGTTAA : 3638
cen3-CR4 : TTATAAATATATTATATTTACTAGATTTATTTAATTATAAAAGTGCTTATATATTTAACTTAGCTTTAAAAAAGTTTAAAAGCTTTTAAATTATATTTAA : 3504
cen4-CR1 : TTATAAGTATATTATATTTACTAAACTTATTTAATTATAAAAGTGCTTATATATTTAACTTAGCTTTAAAAAAGTCTAAAAGCTTTTAAATTATATTTAA :  762
cen4-CR2 : TTATAAGTATATTATATTTACTAGACTTATTTAATTATAAAAGCGCTTATATATTTAACTTAGCTTTAAAAAAGTCTAGAAGCTTTTAAATTATATTTAA : 3682
cen4-CR3 : TTATAAATATATTATATTTATTAAACTTATTAAATTATAAAAGTATTTATATATTTAACTTAGCCTTAAAGTAATTTAAGGGCTTTTAAATTATATTTAA :  214
cen4-CR4 : TTATAAGTATATTATATTTACTAAACTTATTTAATTATAAAAGTGCTTATATATTTAACTTAGCTTTAAAAAAGTCTAAAAGCTTTTAAATTATATTTAA : 3765
cen4-CR5 : TTATAAATATA-------------ACTTATTTAATTATAAAAGCACTTATATATTTAACCTAGCTTTAAAATAATTTAAAAGCTTTTAAATTATATTTAA :  381
cen5-CR1 : TTATAAGTATACTATATTTACTAGACTTATTTAATTATAAAAGCGCTTATATATTTAACTTAGCTTTAAAGAAATTTAAGAGCTTTTAGATTATATTTAA : 3569
cen6-CR1 : TTATAAATATACTATATTTATTAAACTTATTTAATTATAAAAGTATTTATATATTTAACTTAGCTTTAAAAAAGTCTTAAAGCTTTTAAATTATATTTAA : 3784
cen7-CR1 : TTATAAATATACTATATTTATTAAACTTATTTAATTATAAAAGCGCTTATATATTTAACTTAGCTTTAAAAAAATTTAAAAGCTTTTAAATTATATTTAA : 3552
cen7-CR2 : TTATAAATATATTATATTTATTAAACTTATTTAATTATAAAAGCGCTTATATATTTAACTTAGCTTTAAAAAAGTTTAAAAGCTTTTAAATTATATTTAA : 3577
cen7-CR3 : TTATAAATATACTATATTTATTAAACTTATTTAATTATAAAAGTGCTTATATATTTAACTTAGCTTTAAAAAAGTTTAAAAGCTTTTAAATTATATTTAA : 3530
           TTATAA TaTA tatattta ta AcTTATTtAATTAtAaAAG  cTTATATATTTAACtTAgCtTTAAA aAgT TaaaaGCTTTtAaaTTATAtTTAA

                                  *         3920           *         3940           *         3960           *         3980           *         4000
cen1-CR1 : ATAAAAGCTAATTTAAAAAGTATTTTATTAATAAATATTATATAAATATTATAAGTAAACTTAATTAAAGGTAATTTAATTACTTAATTAAGTTAGTTTTT : 3721
cen1-CR2 : GCAGGAGCTAACTTAAGGAGTATCCTATTAATAAATATTATATAGTATTATAAGTAAACTTAGCTAAAGGCAATTTATTTACTTAATTAAATTAATCCTT : 3829
cen1-CR3 : GCAGGAGCTAACTTAAGGAGTGTCTTATTAATAAATATTATATAGTATTATAAATAAACTTAATTAAAGGCAATTTATTTACCTAATTAAGTTAGTTCTT : 3874
cen1-CR4 : GTAAAAGCTAACTTAAGGAGTGTCCTGTTAATAAATATTATGCAGTATTATAAGTAAACTTAGCTAAAGGCAATTTATTTACCTAATTAAGTTAGTTCTT : 3566
cen1-CR5 : GCAAAAGCTAACTTAAAAAGTGTCTTATTAATAAATATTATACAGTATTATAAGTAAACTTAGCTAAAGGTAATTTATTTACCTAATTAAGTTAGTCCTT : 3634
cen2-CR1 : GTAAAAGCTAGCTTAAAAAGTATTTTATTAATAAATATTATATAATATTATAAGTAAACTTAGCTAAAGGTAATTTAATTACTTAATTAAGTTAGTTTTT : 2608
cen2-CR2 : GTAAGAGCTAACTTAAGGAGTGTCTTATTAATAAATATTATATAGTATTATAAGTAAACTTAGCTAAAGGTAATTTATTTACTTAATTAAGTTAGTCTTT : 3881
cen2-CR3 : GCAAGAGCTAACTTAAGGAGTGTCCTGTTAATAAATATTATGCAGTGTTATAGGTAAACTTAGCTAAAGGCAATTTATTTACTTAATTAAGTTAGTCCTT : 3716
cen2-CR4 : ATAATAACTAGCTTAAAAAGTATTTTATTAATAAATATTATACTATATTATAAATAAACTTAATTAAAAAGAGTTTAATTACTTAATTAAGTTAATTTTT :  874
cen2-CR5 : GTAAAAGCTAATTTAAAAAGTATTTTATTAATAAATATTATATAGTATTATAAATAAACTTAACTAAAAGTAATTTATTTACTTAATTAAGTTAGTTCTT : 2064
cen3-CR1 : GTAAGAGCTAATTTAAAAAGTGTTTTATTAATAAATATTATATAGTATTATAAGTAAATTTAGCTAAAAGTAATTTAATTACTTAATTAAGTTAATTCTT : 2148
cen3-CR2 : GCAAGAGCTAATTTAAAAAGTGTCTTATTAATAAATATTATATAATATTATAAATAAACTTAGCTAAAGGTAATTTATTTACTTAATTAAATTAATCTTT : 3700
cen3-CR3 : ATAAAAGCTAGCTTAAAAAGTATTTTATTAATAAATATTATATAATATTATAAGTAAATTTAGCTAAAGGTAATTTAATTACTTAATTAAGTTAGTTTTT : 3738
cen3-CR4 : GTAAAAGCTAACTTAAAAAGTATTTTATTAATAAATATTATATAGTATTATAAATAAACTTAGCTAAAAGTAATTTAATTACTTAATTAAGTTAGTTTTT : 3604
cen4-CR1 : GTAAGAGCTAACTTAAAAAGTATTTTATTAATAAATATTATATAATATTATAAGTAAACTTAGCTATTAGTAATTTAATTACTTAATTAAGTTAGTTCTT :  862
cen4-CR2 : GTAAAAGCTAATTTAAGAAGTGTTTTATTAATAAATATTATATAGTATTATAAATAAACTTAGCTAATAGTAATTTAATTACTTAATTAAATTAGTCCTT : 3782
cen4-CR3 : GTAATAACTAGCTTAAAAAGTATTTTATTAATAAATATTATATTATATTATAAGTAAATTTAATTAAAAAAAGTTTAATTACTTAATTAAGTTAGTTTTT :  314
cen4-CR4 : GTAAGAGCTAATTTAAAAAGTGTTTTATTAATAAATATTATATAGTATTATAAGTAAACTTAGTTAATAGTAATTTAATTACTTAATTAAGTTAGTTTTT : 3865
cen4-CR5 : GTAATAACTAGCTTAAAAAGTATTTTATTAATAAATATTATATTATATTATAAATAAACTTAATTAAAAGAAGTTTAATTACTTAATTAAGTTAGTATTT :  481
cen5-CR1 : GTAAGAGCTAACTTAAAAAGTGTTTTATTAATAAATATTATGCAGTATTATAAGTAAACTTAGCTAAAGGTAATTTATTTACTTAATTAAGTTAGTTCTT : 3669
cen6-CR1 : GTAAAAGCTAATTTAAAAAGTATTTTATTAATAAATATTATATAATATTATAAGTAAACTTAGCTAAAGGTAATTTATTTACTTAATTAAGTTAGTTTTT : 3884
cen7-CR1 : GTAAAAGCTAACTTAAAAAGTGTTTTATTAATAAATATTATATAGTATTATAAGTAAACTTAGCTAAAAGTAATTTATTTACTTAATTAAGTTAGTTTTT : 3652
cen7-CR2 : GTAAAAGCTAATTTAAAAAGTATTTTATTAATAAATATTATATAATATTATAAGTAAACTTAGCTAAAGGTAATTTAATTACTTAATTAAATTAGTTTTT : 3677
cen7-CR3 : GTAAAAGCTAATTTAAAAAGTATTTTATTAATAAATATTATATAGTATTATAAGTAAACTTAGCTAAAAGTAATTTAATTACTTAATTAAGTTAGTCTTT : 3630
           gtAa AgCTAa TTAA aAGT T tTaTTAATAAATATTATata TaTTATAa TAAAcTTA  TAaa g AaTTTA TTACtTAATTAAgTTAgT  TT
```

```
                    *      4020         *      4040         *      4060         *      4080         *      4100
cen1-CR1 : ATTATAAAAGGCTTATAAGTAATATTTAAGATTTTAATTTTATTTTTTTATTTAGCTATTTATTTATAAATAAAATATAATACTTAATTATTACTTAGTT : 3821
cen1-CR2 : ATTATAAAAGGCTTATAAGTAATATTTAAGATTTTAGTTTTATTTTTTTATCTAGCTATTTATTTATAAATAAAATATAATACTTAATTATTACTTAGCT : 3929
cen1-CR3 : ATTATAAAAGGCTTATAAGTAATATTTAAGATTTTAATTCTATTTTTTTATCTAGCTATTTATTTATAAATAAAATATAGTACTTAATTATTACTTAGCT : 3974
cen1-CR4 : ATTATAAAAGGCTTATAAGTAATATTTAAGATTTTAGTTTTATTTCTTTATTTAGCTATTTATTTATAAATAAAATATAATATTTAATTACTACTTAGCT : 3666
cen1-CR5 : ATTATAAAAGGCTTATAAATAATATTTAAGATTTTAGTTTTATCTCTTTATCTAGCTATTTATTTATAAATAAAATACAGTGCTTAATTACTACTTAGCT : 3734
cen2-CR1 : ATTATAAAAGGCTTATAAATAATATTTAAGATTTTAATTATATTTTTTTATTTAGCTATTTATTTATAAATAAAATATAATACTTAATTATTACTTAATT : 2708
cen2-CR2 : ATTATAAAAGGCTTATAAGTAATATTTAAGATTTTAATTTTATTTTTTTATTTAATTATTTATTTATAAATAAAATATAGTACTTAATTATTACTTAGCT : 3981
cen2-CR3 : ATTATAAAAGGCTTATAAGTAATATTTAAGATTTTAGTTTTATCTTTTTATTTAGCTATCTATCTGCAAATAAAATATAATGCTTAATTATTACTTAGCT : 3816
cen2-CR4 : ACTATAAAAAGCTTATAAATAGTATTTAAGATTTTAATTTTATTACTTTATTTAGCTATTTATTTATAAATAAAATATAATATTTAATTATTATTTAATT :  974
cen2-CR5 : ATTATAAAAGGCTTATAAGTAATATTTAGAATTTTAATTTTATTTTTTTATTTAGTTATTTATTTATAAATAAAATATAATACTTAATTACTACTTAGCT : 2164
cen3-CR1 : ATTATAAAAGGCTTATAAGTAATATTTAAAA-TTTAATTTTATTTTTTTATTTAGCTATTTATTTATAAATAAAATATAATACTTAATTACTACTTAATT : 2247
cen3-CR2 : ATTATAAAAGGCTTATAAGTAATATTTAATATTTTAGTTTTATTTTTTTATTTAGCTATTTATTTATAAATAAAATGCAGTACTTAATTACTACTTAGCT : 3800
cen3-CR3 : ATTATAAAAGGCTTATAAGTAATATTTAAAATTTTAATTTTATTTTTTTATTTAGCTATTTATTTATAAATAAAAGATAATATTTAATTATTACTTAGTT : 3838
cen3-CR4 : ATTATAAAAGGCTTATAAATAATATTTAAAATTTTAATTTTATTTTTTTATTTAGCTATTTATTTATAAATAAAATATAATACTTAATTACTACTTAGCT : 3704
cen4-CR1 : ATTATAAAAAGCTTATAAGTAATATTTAAGATTTTAATTTTATTTTTTTATTTAGCTATTTATTTATAAATAAAATATAATACTTAATTATTACTTAGCT :  962
cen4-CR2 : ATTATAAAAAGCTTATAAATAATATTTAAGATTTTAATTTTATTTTTTTATTTAGCTATTTATTTATAAATAAAATATAATACTTAATTATTACTTAGCT : 3882
cen4-CR3 : ATTATAAAAAGCTTATAAGTAATATTTAAGGTTTTAATTTTATTACTTTATTTAGCTATTTATTTATAAATAAAATATAATACTTAATTATTACTTAGCT :  414
cen4-CR4 : ATTATAAAAGGCTTATAAGTAATATTTAAAATTTTAGTTTTATTTTTTTATTTAGCTACTTATTTATAAATAAAATATAGTACTTAATTATTACTTAGCT : 3965
cen4-CR5 : ATTATAAAAAGCTTATAAATAGTATTTAAGGTTTTAGTTTTATTATTTTATTTAGCTATTTATTTATAAATAAAATATAGTATTTAGTTATTACTTAATT :  581
cen5-CR1 : ATTATAAAAGGCTTATAAGTAATATTTAAGATTTTAATTTTATCTTTTTATTTAGCTATTTATTTATAAATAAAATATAATACTTAATTACTACTTAGTT : 3769
cen6-CR1 : ATTATAAAAGGCTTATAAGTAATATTTAAGATTTTAATTTTATTTTTTTATTTAGCTATTTATTTATAAATAAAATATAGTACTTAATTACTACTTAGCT : 3984
cen7-CR1 : ATTATAAAAGGCTTATAAGTAATATTTAAAATTTTAATTTTATTTTTTTATTTAGCTATTTATTTATAAATAAAATATAGTACTTAATTACTACTTAGTT : 3752
cen7-CR2 : ATTATAAAAGGCTTATAAATAATATTTAAGATTTTAATTTTATTTTTTTATTTAGCTACTTATTTATAAATAAAATATAATACTTAATTATTATTTAGCT : 3777
cen7-CR3 : ATTATAAAAAGCTTATAAATAATATTTAAGATTTTAATTTTATTTTTTTATTTAGCTATTTATTTATAAATAAAATATAATACTTAATTATTATTTAGCT : 3730
            AtTATAAAA GCTTATAA TAaTATTTAa atTTTA TTtTATttttTTATtTAgcTAttTATtTatAAATAAAAtatA TacTTAaTTA TAcTTAg T

                    *      4120         *      4140         *      4160         *      4180         *      4200
cen1-CR1 : ATAAAAAGGTAGTAAAAACTTTATTAAAACTAGCTAGTAAAAACTAAACCTTAATTAAACTTAATATTATTTAAAATATTATAAAGTTAAAAAATATAAT : 3921
cen1-CR2 : ATAAAAAGGTAATAAAAACTTTACTAAAACTAGCTAATAAAAACTAAACCTTAGTTAGAAATAATACTATTTAAAATACTATAAGGTTAAAAAATATAAT : 4029
cen1-CR3 : ATAAAAAGGTAGTAAAAACTTTACTAAAACTAGCTAGTAAAAACTAAACCTTAGTTAAAAATAATACTATTTAAAACACTATAAGGTTAAAAGATATAAT : 4074
cen1-CR4 : ATAAAAAGGTAGTAAAAACTTTATTAAAACTAGCTAGTAAAAACTAAACCTTAGTTAGAAATAATACTATTTAAAACACTATAAAGTTAAAAGATATAAT : 3766
cen1-CR5 : ATAAAAAGGTAATAAAAACTTTACTAAAACTAGCTAGTAAAAACTAAACTTTAATTAAAAATAATACTATTTAAAATATTATAAGGTTAAAAGATATAAT : 3834
cen2-CR1 : ATAAAAAAGTAGTAAAAACTTTATTAAAACTAGCTAGTAAAAACTAAACCTTAATTAAAAATAATACTATTTAAAATACTATAAAATTAAAAAATATAAT : 2808
cen2-CR2 : ATAAAAAGGTAGTAAAAACTTTATTAAAACTAGCTAGTAAAGACTAAACCTTAATTAAAAATAATATTATTTAAAATACTATAAGGTTAAAAAATATAAT : 4081
cen2-CR3 : ATAAAAAGGTAGTAGAAACTTTACTAAAACTAGCTAGTAAAGACTAAACCTTAGTTAAAAATAATACTATTTAAAATATTATAAGGTTAGAAAATATAAT : 3916
cen2-CR4 : ATAAAAAAGTAATAAAAGCTTTATTAAAATTAATTAATAAAAACTAAAGTTTAATTAAAAATTATATTATTAAAAATATTATAAAGTTAAAA-ATATAAT : 1073
cen2-CR5 : ATAAAAAGGTAGTAAAAACTTTATTAAAACTAGCTAGTAAAGACTAAACCTTAATTAAAAATAATACTATTTAAAATATTATAAAGTTAAAAAATATAAT : 2264
cen3-CR1 : ATAAAAAAGTAATAAAAACTTTACTAAAACTAGCTAATAAAAACTAAACCTTAATTAAAAATAATACTATTTAAAATACTATAAAATTAAAAAATATAAT : 2347
cen3-CR2 : ATAAAAAGGTAGTAAAAACTTTACTAAAACTAGGTAATAAAAACTAAACCTTAGTTAAAAATAATACTATTTAAAATACTATAAAATTAAAAGATATAAT : 3900
cen3-CR3 : ATAAAAAAGTAATAAAAACTTTACTAAAACTAGCTAGTAAAAACTAAACCTTAATTAAAAATAATACTATTTTAAATACTATAAGATTAAAAAATATAAT : 3938
cen3-CR4 : ATAAAAAGGTAGTAAAAACTTTATTAAAACTAGTTAATAAAAACTAAACCTTAATTAAAAATAATACTATTTAAAATATTATAAAATTAAAAAATATAAT : 3804
cen4-CR1 : ATAAAAAAGTAATTAAAACTTTATTAAAACTAGCTAGTAAAAACTAAACTTTAGTTAAAAATAATACTATTTAAAATACTATAAGGTTAAAAAATATAAA : 1062
cen4-CR2 : ATAAAAAGGTAATAAAAACTTTACTAAAACTAGCTAATAAAAACTAAACTTTAGTTAAAAATAATACTATTTAAAATACTATAAGATTAAAAAATATAAT : 3982
cen4-CR3 : ATAAAAAGATAATAAAAGCTTTATAAAAACTAATTAATAAAAACTAAACCTTAATTAAAAATAATACTATTAAAAATATTA-AAAGTAAAAAAATATAAT :  513
cen4-CR4 : ATAAGAAAGTAATAAAAACTTTACTAAAACTAGTTAATAAAGACTAAACCTTAATTAAAAATAATACTATTTAAAATACTATAAGGTTAAAAGATATAAT : 4065
cen4-CR5 : ATAAAAAAGTAATAAAAGCTTTATTAAAATTAATTAGTAAAAACTAAACCTTAATTAAGAATAATAGTATTAAAAATACTATAAGATTAAAAAATATAAT :  681
cen5-CR1 : ATAAAAAGGTAGTAAAAACTTTACTAAAACTAGCTAGTAAAAACTAAACTTTAATTAAAAATAATACTATTTAAAATATTATAAGGTTAAAAAATATAAT : 3869
cen6-CR1 : ATAAAAAGATAGTAAAAACTTTATTAAAACTAGCTAGTAAAAACTAAACTTTAATTAAAAATAATACTATTTAAAATACTATAAGGTTAAAAAATATAAT : 4084
cen7-CR1 : ATAAAAAGGTAATAAAAGCTTTACTAAAACTAGCTAATAAAAATTAAACTTTAGTTAAAAATAATATTATTTAAAATACTATAAGGTTAAAAAATATAAT : 3852
cen7-CR2 : ATAAAAAAGTAGTAAAAACTTTATTAAAATTAGCTAATAAAAATTAAACTTTAATTAAAAATAATACTATTTAAAATACTATAAGGTTAAAAAATATAAT : 3877
cen7-CR3 : ATAAAAAAGTAATAAAAACTTTACTAAAATTAGCTAATAAAAATTAAACTTTAATTAAAAATAATATTATTTAAAATACTATAAAGTTAAAAAATATAAT : 3830
            ATAAaAA gTA TaaAAaCTTTA tAAAAcTAg TA TAAAaAcTAAAc TTA TTAaaaaTaATA TATTtaAAAtA TAtAA  TtAaAA ATATAAt
```

```
                *       4220        *       4240        *       4260        *       4280        *       4300
cen1-CR1 : AGTAAAATATTTTAATTAGTTTAATATTTATAATATTTTTATAATATAAAATATATAATATATACTTTATAAAGTAATTAATAATAATTAGAATTAAATT : 4021
cen1-CR2 : AGTAAAATATTTTAGCTAATTTAGTATTTATAATATTCTTATAATATAAAATATATAATATATACTTTATAAAGTAATTAATAATAATTAAAATTAAATT : 4129
cen1-CR3 : AGTAAAATATTTTAGCTAGTCTAGTACTTATAATATTTTTATAATATAAAATATATAATATATACTTTATAAAGTAATTAATAATAACTAGAATTAAATT : 4174
cen1-CR4 : AGTAGAATATTTTAGCTAGTCTAGCACTTATAATATTTTTATAGTATAGAATATATAATATATACTTTATAAAGTAATTAATAATAACTAAAATTAAATT : 3866
cen1-CR5 : AGTAAAATATTTTAGCTAGTCTAGTACTTATAATATTCTTATAGTATAAAATATATAATATATATTTTATAAAGTAATTAATAATAACTAAAATTAAATT : 3934
cen2-CR1 : AATAAAATATTTTAATTAGTTTAGTATTTATAATATTCTTATAATATAAAATATATAATATATACTTTATAAAATAATTAATAATAATTAAAATTAAATT : 2908
cen2-CR2 : AATAAAATATTTTAGCTAGTCTAGTACTTATAATATTTTTATAATATAAAATATATAATATATACTTTATAAAGTAATTAATAATAACTAGAATTAAATT : 4181
cen2-CR3 : AATAGAATGTTTTAGCTAGTCTAGTACTTATAATATTTTTATAATATAAAATATATAATATATACTTTATAAAGTAATTAATAATAACTAGGATTAAATT : 4016
cen2-CR4 : AATAAAATATTTTAATTAAAGTAGTATTTATAATATTTTTATAATATAAAATATAAAATATATATTTTATAAAATAATTAATAATAATAAAAATTAAATT : 1173
cen2-CR5 : AATAAAATATTTTAATTAATTTAATATTTATAATATTTTTATAATATAAAATATATAATATATATTTTATAAAGTAATTAATAATAACTAGAATTAAATT : 2364
cen3-CR1 : AGTAAAATATTTTAGCTAATTTAATATTTATAATATTTTTATAATATAAAATATATAATATATATTTTATAAAGTAATTAATAATAATTAAAATTAAATT : 2447
cen3-CR2 : AATAAAATATTTTAGCTAGTCTAGTATTTATAATATTTTTATAATATAAAATATATAATATATACTTTATAAAGTAATTAATAATAACTAGAATTAAATT : 4000
cen3-CR3 : AATAAAATATTTTAGCTAGTTTAATATTTATAATATTTTAATAATATAAAATATATAATATATACTTTATAAAGTAATTAATAATAATTAAAATTAAATT : 4038
cen3-CR4 : AATAAAATATTTTAGCTAATTTAATATTTATAATATTTTTATAATATAAAATATATAATATATATTTTATAAAGTAATTAATAATAATTAAAATTAAATT : 3904
cen4-CR1 : AATAAAATATTTTAGCTAGTATAATACTTATAATATTTTTATAATATAAAATATATAATATATACTTTATAAAGTAATTAATAATAATTAAAATTAAATT : 1162
cen4-CR2 : AATAAAATATTTTAATTAATATAATACTTATAATATTTTTATAATATAAAATATATAATATATACTTTATAAAGTAATTAATAATAACTAAAATTAAATT : 4082
cen4-CR3 : AATAAAATATTTTAATTAAGGCTATACTTATAATATTTTTATAATATAAAATATAAAATATATATTTTATAAAATAATTAATAATAATAAAAATTAAATT :  613
cen4-CR4 : AATAAAATATTTTAGCTAATATAGTATTTATAATATTTTTATAATATAAAATATATAATATATACTTTATAAAGTAATTAATAATAACTAGAATTAAATT : 4165
cen4-CR5 : AATAAAATATATTAATTAAGGCTATATTTATAATATTTTTATAATATAAAATATAAAGTATATATTTTATAAAATAATTTATAATAATAAAAATTAAATT :  781
cen5-CR1 : AGTAAAATATTTTAGCTAGTTTAGTATTTATAATATTCTTATAATATAAAATATATAATATATACTTTATAAAGTAATTAATAATAACTAGAATTAAATT : 3969
cen6-CR1 : AATAAAATATTTTAGCTAGTTTAATATTTATAATATTTTTATAATATAAAATATATAATATATACTTTATAAAGTAATTAATAATAACTAAAATTAAATT : 4184
cen7-CR1 : AATAAAATATTTTAACTAGTTTAGTATTTATAATATTTTTATAATATAAAATATATAATATATATTTTATAAAGTAATTAATAATAACTAAAATTAAATT : 3952
cen7-CR2 : AATAAAATATTTTAGCTAGTTTAATACTTATAATATTTTTATAATATAAAATATATAATATATACTTTATAAAGTAATTAATAATAATTAAAATTAAATT : 3977
cen7-CR3 : AGTAAAATATTTTAGCTAGTTTAATACTTATAATATTTTTATAATATAAAATATATAATATATACTTTATAAAGTAATTAATAATAATTAAAATTAAATT : 3930
           A TAaAATaTtTTA   TA t ta tA TTATAATATTtTtATAaTATAaAATATAtAaTATATA TTTATAAAgTAATTaATAATAA tA aATTAAATT

                *       4320        *       4340        *       4360        *       4380        *       4400
cen1-CR1 : ATATATATAGTTATTATAAATTACTTTTAATAATTTTATAATAAAGTTTATAAATATTTTTTATTATAGTTATATTAAGGTAAGTAGTAGTATAAGTTTA : 4121
cen1-CR2 : ATATATATAGTTATTATAAATTACTTTTAGTAATTTTATAATAAAGTTTATAAATATCTTTTACTATAGTTATATTAAGGTAAGTAGTAAAATAAGTTTA : 4229
cen1-CR3 : ATATATATAATTATTATAAATTACTTTTAATAATTTTATAATAAAGTTTATAAATATCTTTTATTATAGTTATATTAAGGTAAGTAGTAGGATAAGTTTA : 4274
cen1-CR4 : ATATATGCAGTTATTATAAATTACTTTTAGTAATTTTATAATAAAGTTTATAAATATCTCTTACTATAGTTATATTAAGGTAAGTAGCAGGATAAGTTTA : 3966
cen1-CR5 : ATATATATAGTTATTATAAATTACTTTTAATAATTTTATAATAAAGTTTATAAATATCTCTTACTATAGTTATATTAAGGTAAGTAGTAAGATAAGTTTA : 4034
cen2-CR1 : ATATATATAATTATTATAAATTACTTTTAATAATTTTATAATAAAGTTTATAAATATCTTTTACTATAGTTATATTAAAGTAAATAATAATATAAGTTTA : 3008
cen2-CR2 : ATATATATAGTTATTATAAATTACTTTTAGTAATTTTATAATAAATTTTATAAATATCTTTTACTATAATTATATTAAGGTAGGTAATAGGATAAGTTTA : 4281
cen2-CR3 : ATATATATAATTATTATAAATTACTTTTAGTAATTTTATAATAAAGTTTATAAATATCTCTTACTATAGTTATATTAAAGTAAGTAATAGAATAAGTTTA : 4116
cen2-CR4 : ATATATATACTTATTATAAACTGCCTTTAATAAGCTAGTAATAAAATTTATAAATAGTTCTTATTATAAGTATATTAAAGTAAGTAATATAATAAGTTTA : 1273
cen2-CR5 : ATATATATAATTATTATAAATTACTTTTAATAATTTTATAATAAAGTTTATAAATATTTTTTACTATAATTATATTAAAGTAAGTAATAAAATAAGTTTA : 2464
cen3-CR1 : ATATATATAATTATTATAAATTACTTTTAGTAATTTTATAATAAAGTTTATAAATATTTTTTATTATAATTATATTAAAGTAAATAATAATATAAGTTTA : 2547
cen3-CR2 : ATATATATAGTTATTATAAATTACTTTTAGTAATTTTATAATAAAGTTTATAAATATCTCTTACTATAGTTATATTAAGGTAAGTAGCAGAATAAGTTTA : 4100
cen3-CR3 : ATATATATAATTATTATAAATTACTTTTAATAATTTTATAATAAAGTTTATAAATATCTTTTATTATAATTATATTAAGATAAGTAATAGTATAAGTTTA : 4138
cen3-CR4 : ATATATATAATTATTATAAATTACTTTTAATAATTTTATAATAAAGTTTATAAAAATCTTTTATTATAATTATATTAAAGTAAGTAATAAAATAAGTTTA : 4004
cen4-CR1 : ATATATATAATTATTATAAATTACTTTTAATAATTTTATAATAAAGTTTATAAATATCTTTTACTATAATTATATTAATATAAGCAGTAATATAAGTTTA : 1262
cen4-CR2 : ATATATATAGTTATTATAAATTACTTTTAGTAATTTTATAATAAAGTTTATAAATATCTTTTATTATAATTATATTAATATAAGTAGTAATATAAGTTTA : 4182
cen4-CR3 : ATATATATACTTATTATAAACTGCTTTTAATAAGTTAGTAATAAAATTTATAAATAGTTTTTATTATAAGTATATTAAAATAAGTAATATAATAAGTTTA :  713
cen4-CR4 : ATATATATAGTTATTATAAATTACTTTTAGTAATTTTATAATAAAGTTTATAAATATCTCTTATTATAGTTATATTAATATAAGCAGTAGTATAAGTTTA : 4265
cen4-CR5 : ATATATATATTTATTTTAAACTGCTTTTAATAAGCTAGTAATAAAATTTATAAATAATTCTTATTATAAATATATTAAGGTAAGTAATATAATAAGTTTA :  881
cen5-CR1 : ATATATATAGTTATTATAAATTACTTTTAGTAATTTTATAATAAAGTTTATAAATATCTCTTACTATAATTATATTAAGGTAAGTAGTAGGATAAGTTTA : 4069
cen6-CR1 : ATATATATAATTATTATAAATTACTTTTAGTAATTTTATAATAAAGTTTATAAATATTTTTTATTATAGTTATATTAAGGTAAGTAGTAAAATAAGTTTA : 4284
cen7-CR1 : ATATATATAGTTATTATAAATTACTTTTAGTAATTTTATAATAAAGTTTATAAATATCTTTTACTATAATTATATTAAGGTAAATAATAAAATAAGTTTA : 4052
cen7-CR2 : ATATATATAGTTATTATAAATTACTTTTAATAATTTTATAATAAAGTTTATAAATATTTTTTATTATAGTTATATTAAAGTAAGTAATAAAATAAGTTTA : 4077
cen7-CR3 : ATATATATAGTTATTATAAATTACTTTTAATAATTTTATAATAAAGTTTATAAATATTTTTTATTATAGTTATATTAAAGTAAGTAATAAAATAAGTTTA : 4030
           ATATATatA TTATTaTAAAtTaCtTTTA TAAttTtaTAATAAAgTTTATAAAtAt T TTA TATA tTATATTAA gTAagtA tA  ATAAGTTTA
```

```
                  *        4420         *        4440         *        4460         *        4480         *        4500
cen1-CR1 : TTATAAAGTTAATAATATTTTAATTTAGTTTTTTAATAAAT----TATATATATATTAATATATATTTTAATATTTTACTTTATACTTTATTAATAATAA : 4217
cen1-CR2 : CTATAAGGTTAATAATATCTTAGCTTAATTTCTTAATAAAC----TATATATATATTAATATATATTTTAATATTTTACCTTATACCTTATTAATAGTAA : 4325
cen1-CR3 : TTATAAGGTTAATAGTGTTTTAGCTTAGTTTTTTAATAAAC----TATATATATATTAATATATATCTTAATATCTTGCCTTATACCTTACTAATAGTAA : 4370
cen1-CR4 : CTATAAGGTTAATAGTGTTTTAGCTTAGTTTTTTAATAAAC----TATATATATATTAATATATATCTTAATATCTTACTTTATACCTTACTAATAGTAA : 4062
cen1-CR5 : CTATAAGGTTAATAGTATCTTAGCTTAGTTTTTTAATAAAC----TATATATATATTAATATATATCTTAATATCTTGCCTTATACCTTACTAATAGTAA : 4130
cen2-CR1 : TTATAAAGTTAATAATATTTTAACTTAATTTTTTAATAAACTATATATATATATATTAATATATATTTTAATATTTTATCTTATATTTTATTAATAGTAA : 3108
cen2-CR2 : CTATAAGGTTAATAGTGTTTTAGCTTAGTTTTTTAATAAAC----TGTATATATATTAATATATATCTTAATATTTTATCTTATACCTTATTAATAGTAA : 4377
cen2-CR3 : CTATAAAGTTAATAATATTTTAGCTTAGTTTTTTAACAGAC----TGTATATATATTAATATATATCTTAATATCTTGCCTTATACTTTACTAATAGTAA : 4212
cen2-CR4 : TTATAAGGTTAATAGTATTTTAATTTAGTTTTTTAATAAAT----TATATATATATTAATATATATTTTAATATTTTACTTTATACTTTATTAATAATAA : 1369
cen2-CR5 : TTATAAGGTTAATAATATTTTAGTATAGTTTTTTAATAAAC----TATATATATATTAATATATATTTTAATATTTTACTTTATACTTTATTAATAATAA : 2560
cen3-CR1 : TTATAAAGTTAATAATATTTTAGCTTAATTTCTTAATAAAC----TATATATATATTAATATATATTTTAATATCTTGCCTTATACTTTATTAATAATAC : 2643
cen3-CR2 : CTATAAGGTTAATAATATCTTAGCTTAGTTTCTTAATAGAC----TATATATATATTAATATATATTTTAATATCTTATCTTATACCTTATTAATAGTAA : 4196
cen3-CR3 : TTATAAAGTTAATAATATTTTAACTTAATTTTTTAATAAAT----TATATATATATTAATATATATTTTAATATTTTACTTTATACTTTATTAATAGTAA : 4234
cen3-CR4 : TTATAAGGTTAATAGTATTTTAGCTTAGTTTTTTAATAAAC----TATATATATATTAATATATATCTTAATATCTTACTTTATATTTTATTAATAATAA : 4100
cen4-CR1 : TTATAAGGTTAATAGTATTTTAACTTAATTTCTTAATAAAC----TATATATATATTAATATATATTTTAATATCTTGCCTTATACTTTACTAATAGTAA : 1358
cen4-CR2 : TTATAAGGTTAATAATATTTTAGCTTAGTTTTTTAATAAAC----TATATATATATTAATATATATTTTAATATTTTACCTTATACTTTATTAATAATAA : 4278
cen4-CR3 : TTATAAAGTTAATAATATTTTAATTTAATTTCTTAATAAAT----TATATATATATTAATATATATTTTAATATTTTATTTTATACTTTATTAATAATAA :  809
cen4-CR4 : CTATAAGATTAATAATATTTTAACTTAGTTTTTTAATAAAC----TGTATATATATTAATATATATTTTAATATTTTACCTTATACTTTATTAATAGTAA : 4361
cen4-CR5 : TTATAAAGTTAATAATATTTTAATTTAGTTTTTTAATAAAT----TATATATATATTAATATATATTTTAATATTTTACTTTATATTTTACTAATAGTAA :  977
cen5-CR1 : CTATAAGGTTAATAGTGTTTTAGCTTAGTTTCTTAATAAAC----TATATATATATTAATATATATCTTAATATCTTGCCTTATACTTTACTAATAGTAA : 4165
cen6-CR1 : TTATAAAGTTAATAATATTTTAGCTTAGTTTTTTAATAAAC----TATATATATATTAATATATATTTTAATATCTTGCCTTATACTTTATTAATAATAA : 4380
cen7-CR1 : TTATAAAGTTAATAATATCTTAATTTAATTTTTTAATAAAC----TATATATATATTAATATATATTTTAATATCTTGCCTTATACTTTATTAATAATAA : 4148
cen7-CR2 : TTATAAAGTTAATAATATTTTAGCTTAATATTTTAATAAAT----TATATATATATTAATATATATTTTAATATTTTATTTTATACTTTACTAATAGTAA : 4173
cen7-CR3 : TTATAAAGTTAATAATATTTTAGCTTAATTTTTTAATAAAT----TATATATATATTAATATATATTTTAATATCTTATTTTATACTTTACTAATAGTAA : 4126
            TATAA gTTAATA TaTtTTA  tTA TtT TTAAtAaA      TaTATATATATTAATATATAT TTAATAT TT   TTATAc TTA TAATA TAa

                  *        4520         *        4540         *        4560         *        4580         *        4600
cen1-CR1 : TATAATATAATCCTAGCTATTATTTAATAAAGTTTTTAATATTTTATTATAAAATTATTATAAAATAATATAAGGATTTTAAAATATATTATATATAAAA : 4317
cen1-CR2 : TATAATATAATCCTAGCTACTATTTTATAAAGTCCTTAATATCTTACTATAAAATTATTATAAAATAGTATAAGGATTTTAAAGTATATTATATATAAAA : 4425
cen1-CR3 : TATAATATAATCCTAACTACTATTTTATAAAGTCTTTAATATCTTACTATAAGATTATTATAAAATAGTATAAGGATTTTAAAGTATATTATATATAAAA : 4470
cen1-CR4 : TATAATATAATCCTAGCTACTATTTTATAAAGTCCTTAATATCCTACTATAGGATTATTATAAAATAGCATAAGGATTTTAAAGTATATTATATATAAAA : 4162
cen1-CR5 : TATAATATAATCCTAGCTACTGTTTTATAAAGTCTTTAATATCTTACTATAAGATTATTATAAAATAGTATAAGGATTTTAAAGTATATTATATATAAAA : 4230
cen2-CR1 : TATAATATAATTTTAATTATTATTTTATAAAGTTTTTAATATTTTATTATAAAATTATTATAAAATAATATAAGGATTTTAAAATATATTATATATAAAA : 3208
cen2-CR2 : TATAATATAATCCTAGCTATTATTTTATAAAGTCCTTAATATCCTACTATAGGATTATTATAAAATAATATAAGGATTTTAAAATATATTATATATAAAA : 4477
cen2-CR3 : TATAATATAATCCTAGCTATTATTTTATAAAGTTCTTAATATCCTGCTATAAGATTATTATAAAATAGTATAAGGATTTTAAAGCATATTATATATAAAA : 4312
cen2-CR4 : TATAATATAATTTTAATTATTATTTAATAAAGTTTTTAATATTTTATTATAAAATTATTATAAAATAATATAAGAATTTTAAAATATATTATATATAAAA : 1469
cen2-CR5 : TATAATATAATTCTAGTTATTATTTTATAAAGTCTTTAATATTTTATTATAAAATTATTATAAAATAATATAAAGATTTTAAATTATATTATATATAAAA : 2660
cen3-CR1 : TATAATATAATCCTAGCTATTATTTAATAAAGTCTTTAATATTTTATTATAAAATTATTATAAAATAATATAAGGATTTTAAAGTATATTATATATAAAA : 2743
cen3-CR2 : TATAGTATAATTTTAACTACTATTTTATAAAGTCTTTAATATCCTGCTATAAGATTATTATAAAATAGTATAAGGATTTTAAAGCATATTATATATAAAA : 4296
cen3-CR3 : TATAATATAATTTTAATTATTATTTTATAAAGTTTTTAATATTTTATTATAAAATTATTATAAAATAATATAAAGATTTTAAAATATATTATATATAAAA : 4334
cen3-CR4 : TATAATATAATTTTAGCTATTATTTTATAAAATCCTTAATATTTTATTATAAAATTATTATAAAATAATATAAGAATTTTAAAGTATATTATATATAAAA : 4200
cen4-CR1 : TATAATATAATTCTAATTACTATTTTATAAAGTCTTTAATATTTTACTATAAAATTATTATAAAATAATATAAGAATTTTAAAGTATATTATATATAAAA : 1458
cen4-CR2 : TATAATATAATTCTAGCTATTATTTTATAAAGTTTTTAATATTTTATTATAAAATTATTATAAAATAATATAAGAATTTTAAAATATATTATATATAAAA : 4378
cen4-CR3 : TATAATATAATTCTAGCTATTATTTAATAAAGTTTTTAATATTTTATTATAAGATTATTATAAAATAATATAAGGATTTTAAAATATATTATATATAAAA :  909
cen4-CR4 : TATAATATAATCCTAGCTATTATTTTATAAAGTCTTTAATATTTTATTATAAAATTATTATAAAATAATATAAAGATTTTAAAATATATTATATATAAAA : 4461
cen4-CR5 : TATAATATAATCTTAGCTATTATTTAATAAAGTCTTTAATATTTTATTATAAAATTATTATAAAATAATATAAGGATTTTAAAATATATTATATATAAAA : 1077
cen5-CR1 : TATAATATAATCTTAGCTATTATTTTATAAAGTCTTTAATATTTTACTATAAGATTATTATAAAATAATATAAGGATTTTAAAGTATATTATATATATAA : 4265
cen6-CR1 : TATAATATAATTTTAATTACTATTTTATAAAGTCTTTAATATTTTATTATAAAATTATTATAAAATAATATAAGGATTTTAAAGTATATTATATATAAAA : 4480
cen7-CR1 : TATAATATAATTCTAGCTATTATTTTATAAAGTTCTTAATATTTTATTATAAAATTATTATAAAATAATATAAGGATTTTAAAGTATATTATATATAAAA : 4248
cen7-CR2 : TATAATATAATTCTAGCTATTATTTTATAAAGTTTTTAATATTTTATTATAAAATTATTATAAAATAATATAAGGATTTTAAAGTATATTATATATAAAA : 4273
cen7-CR3 : TATAATATAATCCTAGCTATTATTTTATAAAGTTTTTAATATTTTATTATAAAATTATTATAAAATAATATAAGGATTTTAAAATATATTATATATAAAA : 4226
           TATAaTATAAT   TA   TA TaTTTtATAAAgT  TTAATAT tTa TATAa ATTATTATAAAATA tATAAggATTTTAAA tATATTATATATAaAA
```

```
                       *        4620         *        4640         *        4660         *        4680         *        4700
cen1-CR1 : TAAAAACTTATTTATAATATTATAATAACTAGTTTATATTATAAGTTTATTATTTTTAATATATATAT--TATTAATAAAAAAA-CTATTAATTTTAAA : 4414
cen1-CR2 : TAAAAACTTATTTATAATATTATAATAACTAGTCTATACTATAAGTCTATTACTATTTAATATATATAC--TATTAATAAAAGAA-TTATTAATTTTAAA : 4522
cen1-CR3 : TAAAGACTTATTTATAATATTATAATAACTAGTTTATATTATAAGTCTATTACTATTTAATATATATAC--TATTAATAAAAGAA-TTATTAATTTTAAA : 4567
cen1-CR4 : TAAAGACTTATTTATAATATTATAATAACTAGTCTATACTATAAGTCTATTACTTTTTAATATACATGC--TATTAATAAAAGAA-TTATTAACTTTAAG : 4259
cen1-CR5 : TAAAGACTTATTTATAGTATTATAATAACTAGTTTATACTATAAGTTTATTACTTTTTAATATATATAC--TATTAATAAAAGAA-CTATTAACTTTAAG : 4327
cen2-CR1 : TAAAGACTTATTTATAATATTATAATAACTAGTTTATATTATAAGTTTATTACTTTTTAATATATATAT--TATTAATAAAAAAA-TTATTAATTTTAAA : 3305
cen2-CR2 : TAAAAACTTATTTATAATATTATAATAACTAGTTTATATTATAAATTTATTACTTTTTAATATATATAC--TATTAGTAAAAGAA-CTATTAACTTTAAA : 4574
cen2-CR3 : TAAAGACTTATTTATAGTATTATAATAACTAGTTTATACTATAAGTCTATTACTTTATAATACGCATGC--TATTAATAAAAGAA-CTATTAATTTTAAA : 4409
cen2-CR4 : TAAAGACTTATTTATAATATTATAATAACTAGTTTATATTAAAAGTTTATTATTTTTAATATATATAT--TATTAATAAAAAAA-CTATTAATTTTAAA : 1566
cen2-CR5 : TAAAGACTTATTTATAATATTATAATAATTAGTTTATATTATAAATTTATTACTTTTTAATATATATAT--TATTAATAAAAAAA-CTATTAACTTTAAA : 2757
cen3-CR1 : TAAAAACTTATTTATAATATTATAATAACTAGTTTATATTATAAATTTATTACTTTTTAATATATATAT--TATTAATAAAAAAA-CTATTAATTTTAAA : 2840
cen3-CR2 : TAAAAACTTATTTATAGTATTATAATAACTAGTTTATACTATAAGTTTATTACTTTTTAATATATATAC--TATTAGTAAAAGAA-TTATTAACTTTAAG : 4393
cen3-CR3 : TAAAAACTTATTTATAATATTATAATAACTAGTTTATATTATAAGTTTATTACTTTTTAATATATATAT--TATTAATAAAAGAA-TTATTAATTTAAAA : 4431
cen3-CR4 : TAAAAACTTATTTATAATATTATAATAATTAATTTATATTATAAATTTATTACTTTTTAATATATATATAATATTAATAAAAAAATCTTTTATTTTTAAA : 4300
cen4-CR1 : TAAAAACTTATTTATAATATTATAATAACTAGTTTATATTATAAGTTTATTACTTTTTAATATATATAC--TATTAATAAAAAAA-CTATTAATTTTAAA : 1555
cen4-CR2 : TAAAGACTTATTTATAATATTATAATAACTAGTTTATATTATAAATTTATTACTTTTTAATATATATAT--TATTAATAAAAAAA-CTATTAACTTTAAA : 4475
cen4-CR3 : TAAAAACTTATTTATAATATTATAATAACTAGTTTATATTAAAAGTTTATTATTTTTAATATATATAT--TAATAGTAAAAAAAACTATTAGTTTTAAA : 1007
cen4-CR4 : TAAAGACTTATTTATAATATTATAATAACTAGTTTATATTATAAGTTTATTACTTTTTAATATATATAC--TATTAATAAAAGAA-TTATTAACTTTAAA : 4558
cen4-CR5 : TAAAGATTTATTTATAATATTATAATAACTAGTTTATATTAAGAGTTTATTATTTTTAATATATATAT--TATTAATAAAAGAA-TTATTAATTTTAAA : 1174
cen5-CR1 : TAAAGACTTATTTATAGTATTATAATAATTAGTTTATATTATAAATTTATTACTTTTTAATATATATAC--TATTAGTAAAAGAA-CTATTAGTTTTAAA : 4362
cen6-CR1 : TAAAGACTTATTTATAATATTATAATAACTAGTTTATATTATAAGTTTATTACTTTTTAATATATATAC--TATTAATAAAAAAA-CTATTAACTTTAAG : 4577
cen7-CR1 : TAAAGACTTATTTATAATATTATAATAACTAGTTTATATTATTAATTTATTACTTTTTAATATATATAC--TATTAATAAAAAAA-CTATTAATTTTAAA : 4345
cen7-CR2 : TAAAAACTTATTTATAATATTATAATAATTAGTTTATATTATAAGTTTATTATATTTTAATATATATAT--TAATAGTAAAAAAA-CTATTAGTTTTAAA : 4370
cen7-CR3 : TAAAAACTTATTTATAATATTATAATAATTAATTTATATTATAAGTTTATTATATTTTAATATATATAT--TAATAGTAAAAAAA-CTATTAGTTTTAAA : 4323
           TAAA AcTTATTTATAaTATTATAATAAcTAgTtTATAtTAtaA TtTATTA ttTtTAATAtatATa   TAtTA TAAAA AA  TaTTAa TTtAAa

                       *        4720         *        4740         *        4760         *        4780         *        4800
cen1-CR1 : AATTTATATAAATATTTAAATATAAGTGTTTTTATATTAAGCTTTTTATAATACTTTTTTAAGTATTTTTTAATTTTTTTATAATATTATAATAATGCTT : 4514
cen1-CR2 : AGTTTATATAAGTATTTATATATAAGTGTTTTTATATTAAGCTTTTTATAATGCCTTTTTAAGTATCCTTTAATCTTTTTATAATATTATAGCAGCACTT : 4622
cen1-CR3 : AGTTTATATAAGTATTTATATATAAGCATTTTTATATTAAGCTTTTTATAATGCCTTTTTAAGTGTTTTTTAGTCTTTTTATAATATTATAGCAGCGCTT : 4667
cen1-CR4 : AGTCTGCATAAGCATTTATATATAGGCATCCTTATATTAAGCTTTTTATAATGCCTTTTTAAGTATTTTTTAGTCTTTTTATAATATTATAGCAGCACTT : 4359
cen1-CR5 : AGTTTATATAAGTATCTGCATATAGGCGTCTTTATATTAGGCTTTTTATAATGCCTTTTTAAGTGTTTTTTAGTCTTTTTATAATATTATAGCAGTGCTT : 4427
cen2-CR1 : AGTTTATATAAGTATTTAAATATAAGTGTTTTTATATTAAGCTTTTTATAATACTTTTTTAAATATTTTTTAATCTTTTTATAATATTATAATAATACTT : 3405
cen2-CR2 : AGTTTATATAAATATTTATATATAAGTATTCTTATATTAAGCTTTTTATAATATCTTTTTAAGTGTTTTTTAGTCTTTTTATAATATTATAATAGCGCTT : 4674
cen2-CR3 : AGTCTGCATAAGCATTTATATATAGGCGTCCTTATATTAAGCTTTTTATAATGCCTTTTTAAGTATTTTCTAGTCTTTTTATAATATTATAGCAGCGCTT : 4509
cen2-CR4 : AGTTTATATAAGTATTTAAATATAAGTATTTTTATATTAAGCTTTTTATAATA-TTTTTTAAGTATTTTTTAGTTTTTTTATAATATTATAATAGCGCTT : 1665
cen2-CR5 : ATTTTATATAAGTATTTATATATAGGTATTTTTATATTAAGCTTTTTATAATACTTTTTTAAGTATTTTTTAATCTTTTTATAATATTATAATAGCGCTT : 2857
cen3-CR1 : AGTTTATATAAGTATTTAAATATAAGTATTTTTATATTAAGCTTTTTATAATACTTTTTTAAGTATTTTCTAGTCCTTTTATAATATTATAGTAATACTT : 2940
cen3-CR2 : AGTTTATATAAGTATTTATATATAGGCATCCTTATATTAGGCTTTTTATAATACCTTTTTAAATATCTTCTAGTCTTTTTATAATATTATAGCAGCATTT : 4493
cen3-CR3 : AGTTTATATAAATATTTAAATATAAGTGTTTTTATATTAGGCTTTTTATAATACCTTTTTAAGTATTTTTTAATTTTTTTATAATATTATAATAATACTT : 4531
cen3-CR4 : AGTTTATATAAGTATTTAAATATAAGTGTTTTTATATTAGGCTTTTTATAATACCTTTTTAAATATTTTTTAATTTTTTTATAATATTATAATAATATTT : 4400
cen4-CR1 : AGTTTATATAAGTATTTATATATAAGTATTTTTATATTAGGCTTTTTATAATATTTTTTTAAGTATTTTCTAGTCTTTTTATAATATTATAGCAGCGCTT : 1655
cen4-CR2 : AGTTTATATAAGTATTTATATATAGGCATCCTTATATTAAGCTTTTTATAATACCTTTTTAAGTATTTTTTAATTTTTTTATAATATTATAATAGCGCTT : 4575
cen4-CR3 : AGTTTATATAAATATTTAAATATAAGTATTTTTATATTAAGCTTTTTATAATACTTTTTTAAATATTTTTTAGTTTTTTTATAATATTATAATAATACTT : 1107
cen4-CR4 : AGTTTATATAAGTATTTATATATAGGTATTTTTATATTAGGCTTTTTATAATGCCTTTTTAAGTGTCTTTTAGTCTTTTTATAATATTATAATAATACTT : 4658
cen4-CR5 : AGTTTATATAAGTATTTAAATATAAATATTCTTATATTAGGCTTTTTATAATACCTTTTTAAATATTTTTTAATTTTTTTATAATATTATAATAATATTT : 1274
cen5-CR1 : AGTTTATATAAGTATTTAAATATAGGCATTTTTATATTAAGCTTTTTATAATACCTTTTTAAGTATTTTTTAGTTTTTTTATAATATTATAGCAGCGCTT : 4462
cen6-CR1 : AGTTTATATAAGTATTTATATATAAGTATTTTTATATTAAGCTTTTTATAATGCCTTTTTAAATATTTTCTAGTCTTTTTATAATATTATAGCAGTGCTT : 4677
cen7-CR1 : AGTTTATATAAGTATTTATATATAAGTATTTTTATATTAGGCTTTTTATAATGCCTTTTTAAGTATTTTTTAGTTTTTTTATAATATTATAATAGCACTT : 4445
cen7-CR2 : AGTTTATATAAGTATTTAAATATAAGTATTTTTATATTAAGCTTTTTATAATACTTTTTTAAGTATTTTTTAATCTTTTTATAATATTATAATAATACTT : 4470
cen7-CR3 : AGTTTATATAAGTATTTAAATATAGGTATTTTTATATTAAGCTTTTTATAATACTTTTTTAAGTATTTTTTAGTTTTTTTATAATATTATAATAATACTT : 4423
           AgTtTatATAAgtATtTa ATATA g  Tt TTATATTA GCTTTTTATAAT c TTTTTAA TaTttTtTA T tTTTTATAATATTATA  A   cTT
```

```
                    *      4820         *      4840         *      4860         *      4880         *      4900
cen1-CR1 : TATTATATCTTTAAAGTTAACTTAAATATATATTAAAAGCAGGCTACTTATATACTTATAATTATAGCCCTTTTAGC-TTAAAGAACTGCCTTTAAGTAT : 4613
cen1-CR2 : TATTATGCCTTTAAGGTTAACTTAAATACATACTAAAAGCAGGCTACTTATATACTTATAATTATAGCCCTTTTAGC-TTAAGGAACTGCCTTTAAATAT : 4721
cen1-CR3 : TATTATGCCTTTAAGGTTAACCTAAATATATATTAAAGGCAGGCTACTTATATATTTATAATTATAGCCCTTTTAGC-TTAAAAAACTGCCTTTAAATAT : 4766
cen1-CR4 : TATTATGCCTTTAAGGTTAACCTAAATATATACTAAAAGCAGGCTACTTATATACTTATAATTATAGCCCTCTTAGC-TTAAGGAACCGCCCCTAGGTAT : 4458
cen1-CR5 : TATTATACCTTTAAAGTTAACTTAAATATATATTAAAGGCAGGCTACTTATATATTTATAATTACAGCCCTCCTAGC-TTAAGGAACTGCCTTTAAGTAT : 4526
cen2-CR1 : TATTATACCTTTAAAATTAATTTAAATTTATATTAAAGGCAGGTTACTTATATACTTATAATAATAGCCCTTCTAGC-TTAAAGAACTACTTTTAAATAT : 3504
cen2-CR2 : TATTATACCTTTAAGATTAACCTAGATATATATTAAAGGCAGGCTACTTATATACTTATAATTATAGCCCTTTTAGC-TTAAGGAACTGCCTTTAAGTAT : 4773
cen2-CR3 : TATTATGCCTTTAAGGTTAACCTAAATGTATACTAAAAGCAGGCTACTTATATACTTATAATTATAGCCCTTCTAGC-TTAAAGAACTGCCCTTAAATAT : 4608
cen2-CR4 : TATTATACTTTTAAAGTTAATTTAAATATATATTAAAAGTAGGCTACTTATATATTTATAATTATAACCCTTTTAGC-TTAAAAAACTACCTTTAAGTAT : 1764
cen2-CR5 : TATTATACTTTTAAAATTAACTTAAATATATATTAAAAGTAAGCTACTTATATACTTATAATTATAGCCTTTTTAAC-TTAAAAAACTGCCTTTAAGTAT : 2956
cen3-CR1 : TATTATACTTTTAAGGTTAACTTAAATATATATTAAAAGCAGGCTACTTATATATTTATAATTATAGCCCTTTTAGC-TTAAAAAACTACTTTTAAATAT : 3039
cen3-CR2 : TATTATGCCTTTAAGGTTAACCTAAATATATACTAAAGGCAGGCTACTTATATACTTATAATTATAGCCCTTCTAGT-TTAAAAAACTGCTCTTAGGTAT : 4592
cen3-CR3 : TATTATACCTTTAAGGTTAATTTAAATATATATTAAAAGCAGGCTATTTATATACTTATAATTATAGCCCTTTTAGC-TTAAAAAACTGCCTTTAAATAT : 4630
cen3-CR4 : TATTATACTTTTAAAGTTAACTTAAATATATATTAAAGGCAGGCTATTTATATACTTATAATTATAGCCTTTTTAGC-TTAAGAAACTGCCTTTAAGTAT : 4499
cen4-CR1 : TATTATACTTTTAAAATTAACTTAAATATATATTAAAGGCAGGCTATTTATATACTTATAATTATAGCCCTTTTAGC-TTAAAAAACCGCTTTTAAATAT : 1754
cen4-CR2 : TATTATGCCTTTAAGGTTAACCTAAATATATATTAAAGGCAGGCTACTTATATACTTATAATTATAGCCCTTCTAGC-TTAAAAAACTGCCCTTAAGTAT : 4674
cen4-CR3 : TATTATACTTTTAAAGTTAACCTAAATATATATTAAAAGCAGGCTATTTATATATTTATAATTATAGCCTATTTAGCTTTAAAAAACTACCTTTAAGTAT : 1207
cen4-CR4 : TATTATATCTTTAAGGTTAACTTAAATATATATTAAAGGCAGGCTACTTATATACTTATAATTATAGCCCTTTTAGC-TTAAAGAACTGCCTTTAAGTAT : 4757
cen4-CR5 : TATTATATTTTTAAAGTTAACTTAAATATATATTAAAAGCAGGCTATTTATATACTTATAATTATAGCCTTTTTAAC-TTAAAAAACTGCCTTTAAGTAT : 1373
cen5-CR1 : TATTATACCTTTAAGGTTAACTTAAATATATATTAAAAGCAGGCTACTTATATACTTATAATTATAGCCCTTATAGC-TTAAAGAACCGCCCTTAAATAT : 4561
cen6-CR1 : TATTATACCTTTAAGGTTAACTTAAATATATATTAAAAGTAGGCTACTTATATACTTATAATTATAGCCCTTCTAGC-TTAAAGAACTGCCCTTAAATAT : 4776
cen7-CR1 : TATTATGCCTTTAAGGTTAATTTAAATATATATTAAAAGCAGGCTACTTATATACTTATAATTATAGCCTTTTTAAC-TTAAAAAACTGCCTTTAAATAT : 4544
cen7-CR2 : TATTATATTTTTAAAGTTAACCTAAATATATATTAAAAGCAGGCTATTTATATACTTATAATTATAGCCCTTTTAAT-TTAAGAAACTGCCTTTAAGTAT : 4569
cen7-CR3 : TATTATATTTTTAAAATTAACCTAAATATATATTAAAAGCAGGCTATTTATATACTTATAATTATAGCCCTTTTAAT-TTAAGGAACTGCCTTTAAGTAT : 4522
           TATTAT c TTTAA gTTAAc TAaATatATAtTAAA GcAgGcTA TTATATAcTTATAATtAtAgCCctt TAgc TTAA  AACtgCc tTAa TAT

                    *      4920         *      4940         *      4960         *      4980         *      5000
cen1-CR1 : AATTTAAAAGTTAAGTAATTAATTATTAATATAATATTAAAGTTTTAAATTTTTTTTTTTACTTTATAATTAAATTATAATTATTATAAAATTATATAAAA : 4713
cen1-CR2 : AACCTAGGAGTTAAGTAACTAATTATTAATATAATATTAAAGTTTTAAA-TTCCCTTTTACTTTATAGCTAAATTATAATTATTATAAAATTATATAAGG : 4820
cen1-CR3 : AACCTAAAAGTTAAATAACTAATTATTAATATAATATTAAAGTTTTAGG-TTCCCTTTTACTTTATAGCTAAACTATAATTATTATAAAATTATATAAGG : 4865
cen1-CR4 : AACCTAGGGGTTAAGTAACTAATTATTAATAAGATATTAAAGTTCTAGG-TTCCCTCTTACTTTATAGCTAAACTATAATTATTATAAAATTATATAAAA : 4557
cen1-CR5 : AACTTAAGGGTTAAGTAACTAATTATTAATAAGATATTAGAGTTTTAGG-TTCCCTTTTACTTTATAGCTAAACTATAATTATTATAAAATTATATAAAG : 4625
cen2-CR1 : AACCTAAAAGTTAAATAACTAATTAATAATATAATATTAAAGTTTTAAG-TTTTTTTTTACTTTATAGCTAAACTATAATTATTATAAAATTATATAAAA : 3603
cen2-CR2 : AACTTAAAAGTTAAGTAACTAATTATTAATATAATATTAAAGTTTTAAG-TTCCTTTTTACTTTATAGCTAAACTATAATTATTATAAAATTATATAAGG : 4872
cen2-CR3 : AACCTAGGAGTTAAGTAACTAATTATTAGTATAATATTAAAGTTCTAGA-TTCCCTTTTACTTTATAGCTAAACTGTAATTATTATAAAATTATATAAGG : 4707
cen2-CR4 : AACTTAAAAATTAAGTAATTAATTATTAATATAATATTAAAGTTTTAAG-TTTTTTTTTACTTTATAATTAAATTATAATTATTATAAAATTATATAAAA : 1863
cen2-CR5 : AACTTAAAAGTTAAGTAATTAATTATTAATATAATATTAAAGTTTTAAG-TTTTTTTTTATTTTATAACTAAACTATAATTATTATAAAATTATATAAAA : 3055
cen3-CR1 : AACCTAAAAGTTAAGTAATTAATTATTAATATAATATTAAAGTTTTAAG-TTCTTTTTTATTTTATAATTAAATTATAATTATTATAAAATTATATAAAA : 3138
cen3-CR2 : AACCTAGGAGTTAAGCAGCTAATTATTAATATAATATTAAAGTTCTAGG-TTCCCTTTTACTTTATAGTTAAATTATAATTATTATAAAATTATATAAAA : 4691
cen3-CR3 : AACTTAAAAGTTAAATAATTAATTATTAATATAATATTAAAGTTTTAAATTTTTTTTTTTACTTTATAATTAAACTATAATTATTATAAAATTATATAAAA : 4730
cen3-CR4 : AATTTAAAAGTTAAGTAATTAATTATTAATATAATATTAAAGTTTTAAA-TTCTTTTTTACTTTATAGCTAAATTATAATTATTATAAAATTATATAAAA : 4598
cen4-CR1 : AACCTAAAAGTTAAGTAATTAATTATTAATATAATATTAAAGTTTTAAG-TTCCTTTTTATTTTATAGCTAAACTATAATTATTATAAAATTATATAAAA : 1853
cen4-CR2 : AACTTAAAAGTTAAGTAATTAATTATTAATATAATATTAAAGTTTTAGG-TTTTTTTTTACTTTATAGCTAAATTATAATTATTATAAAATTATATAAAA : 4773
cen4-CR3 : AACTTAAAAGTTAAGTAATTAATTATTAATATAATATTAAAGTTTTAAG-TTTTTATTTATATTATAGTTAAATTATAATTATTATAAAATTATATAAAA : 1306
cen4-CR4 : AATTTAGAAGTTAAATAACTAATTATTAATATAATATTAAAGTTTTAGG-TTTTTTTTTACTTTATAGCTAAATTATAATTATTATAAAATTATATAAAA : 4856
cen4-CR5 : AATCTAAGAGTTAAATAACTAATTATTAATATAATATTAAAGTTTTAAG-TTTTTTTTTACTTTATAGCTAAATTATAATTATTATAAAATTATATAAAA : 1472
cen5-CR1 : AATTTAAGAGTTAAGTAACTAATTATTAATATAATATTAAAGTTTTAGG-TTTTTTTTTACTTTATAGCTAAACTATAATTATTATAAAATTATATAAAG : 4660
cen6-CR1 : AACTTAAAAGTTAAGTAATTAATTATTAATATAATATTAAAGTTTTAAA-TTCTTTTTTACTTTATAGCTAAATTATAATTATTATAAAATTATATAAAA : 4875
cen7-CR1 : AATTTAAAAGTTAAGTAATTAATTATTAATATAATATTAAAGTTTTAAG-TTCTTTTTTACTTTATAGCTAAACTATAATTATTATAAAATTATATAAGA : 4643
cen7-CR2 : AATTTAAGAGTTAAGTAATTAATTATTAATATAATATTAAAGTTTTAAATTTTTTTTTTTACTTTATAATTAAATTATAATTATTATAAAATTATATAAAA : 4669
cen7-CR3 : AATTTAAAAGTTAAGTAATTAATTATTAATATAATATTAAAGTTTTAAA-TTTTTTTTTACTTTATAATTAAATTATAATTATTATAAAATTATATAAAA : 4621
           AA  TAa agTTAAgtAa TAATTAtTAaTAtaATATTAaAGTTtTA   TT   ttTTActTTATA  TAAA TaTAATTATTATAAAATTATATAAa
```

```
                    *      5020         *      5040         *      5060         *      5080         *      5100
cen1-CR1 : AATAATTTAAAATAGTTATAAATTATTTTATTTTATTTTTTATTATTAT-AAATATTATTATATTTTATAAGTATATTAGCTAAGTTAGCT--------- : 4803
cen1-CR2 : AATAGTTTAAAGTAGCTATAAGCTATTTTATCTTATCCTTTATTATTAT-AAATATTATTATACCTTATAAATATATTAGCTAAGTTAGCT--------- : 4910
cen1-CR3 : AATAGTTTAAGGTAGCTATAAGCTATTTTATCTTATCTTTTACTATTAT-AAATATTATTATATTTTATAAATACATTAGCTAAGTTAGCT--------- : 4955
cen1-CR4 : AACAGTTTAAAGTAGCTATAAGCTATTTTATCTTATCTTTTACTACTAT-AAATATTATTATGCTTTATAAATATATTAGCTAGGTTAGCT--------- : 4647
cen1-CR5 : AATAGTTTAAGGTAGCTATAAGCTATTTTATTTTATCCTTTATTATTAT-AAATATTATTATACCTTATAAGTGTATTAGCTAGGTTAGCT--------- : 4715
cen2-CR1 : AATAATTTAAAGTAATTATAAGCTATTTTATTTTAGCTTTTATTATTATAAAATATTATTATATTTTATAAGTATATTAGCTAAATTAGCT--------- : 3694
cen2-CR2 : AATAGTTTAAGGTAGTTATAAGTTATTTTATTTTATTCTTTACTATTAT-AAATATTATTATACTTTATAAATATATTAGCTAGGTTAGCT--------- : 4962
cen2-CR3 : AATAGTTTAAGGTAGCTATAAGCTATTTTATCTTATCCTTTATTACTGT-AAATATTATTATACCTTATAAATATATTAGCTAGGTTAGCT--------- : 4797
cen2-CR4 : AATAGTTTAAAATAGCTTTAAGTTATTTTATTTTATTTTTTATTATTAT-AAATATTATTATATTTTATAAATATATTAATTAAATTAGCT--------- : 1953
cen2-CR5 : AATAGTTTAAAATAGCTATAAGCTATTTTATTTTATTCTTTATTATTAT-AAATATTATTATACTTTATAAATATATTAGCTAAGTTAGCT--------- : 3145
cen3-CR1 : AATAAATTAAAATAGCTATAAGTTATTTTATTTTATTTTTTATTATTAT-AAATATTATTATATTTTATAAATATATTAACTAAATTAGCT--------- : 3228
cen3-CR2 : AATAGTTTAAAATAGCTATAAGCTATTTTATTTTATCCTTTATTATTAT-AAATATTATTATATTTTATAAGTATATTAGCTAGGTTAGCT--------- : 4781
cen3-CR3 : AATAGTTTAAAATAGCTATAAGCTATTTTATTTTATTTTTTATTATTAT-AAATATTATTATATTTTATAAATTTATTAACTAAATTAGCT--------- : 4820
cen3-CR4 : AATAATTTAAAATAGCTATAAATTATTTTATTTTATTTTTTATTATTAT-AAATATTATTATATTTTATAAATATATTAACTAAATTAGCT--------- : 4688
cen4-CR1 : AATAGTTTAAAATAGCTATAAGCTATTTTATTTTATCTTTTACTATTAT-AAATATTATTATATTTTATAAATATATTAGCTAGGTTAGCT--------- : 1943
cen4-CR2 : AATAATTTAAAGTAGTTATAAGCTATTTTATTTTATTTTTTATTATTAT-AAATATTATTATATTTTATAAATATATTAGCTAGGTTAGCT--------- : 4863
cen4-CR3 : AATAATTTAAAATAACTTTAAGCTATTTTATTTTATTTTTTATTATTAT-AAATATTATTATATTTTATAAATATATTAACTAAATTAGCT--------- : 1396
cen4-CR4 : AATAATTTAAAATAGCTATAAGCTATTTTATTTTATTCTTTACTATTAT-AAATATTATTATATTTTATAAATATATTAGCTAAATTAGCT--------- : 4946
cen4-CR5 : AATAATTTAAAATAGCTTTAAGCTATTTTATTTTATTTTTTATTATTAT-AAATATTATTATATTTTATAAATATATTAATTAAATTAGCT--------- : 1562
cen5-CR1 : AATAATTTAAAGTAGCTATAAGCTATTTTATCTTATTTTTTATTATTAT-AAATATTATTATATTTTATAAATATATTAGCTAAATTAGCT--------- : 4750
cen6-CR1 : AATAGTTTAAAATAGCTATAAGCTATTTTATTTTATTTTTTATTATTAT-AAATATTATTATATTTTATAAATATATTAGCTAGGTTAGCT--------- : 4965
cen7-CR1 : AATAATTTAAGGTAGTTATAAGCTATTTTATTTTAT-TTTTATTATTAT-AAATATTATTATACTTTATAAGTATATTAGCTAAATTAGCT--------- : 4732
cen7-CR2 : AATAGTTTAAAATAGCTATAAGCTATTTTATTTTATTTTTTATTATTAT-AAATATTATTATATTTTATAAATATATTAGCTAAGTTAGCT--------- : 4759
cen7-CR3 : AATAGTTTAAAATAGCTATAAGCTATTTTATTTTATTTTTTATTATTAT-AAATATTATTATATTTTATAAGTATATTAGCTAAGTTAGCTTTTTTTAAT : 4720
           AAtA tTTAAa TAgcTaTAAgcTATTTTATtTTAt  TTTAtTAtTaT AAATATTATTATa tTTATAA TatATTA cTA  TTAGCT

                    *      5120         *      5140         *      5160         *      5180         *      5200
cen1-CR1 : -----TTTTTTAATTAAAATTTAATTATAAAATTATAAAGTATTAATTTTTATAATTATAATACTTACTTAATTAATAGC-TTTTCCTTTATAATAAGGT : 4897
cen1-CR2 : -----TTTCTTAATTAGAATTTAATTATAAAATTATAAAATGCTAATTTTTATAATTATAATACTTACTTAGTTAATAGC-TTTTTTTTTATAATAAGGT : 5004
cen1-CR3 : -----TTTTTTAATTAGAATTTAATTATAAAATTATAAAGTGCTAATTCTTATAATTATAACACTTACTTAATTAATAGC-TTTTTTTTTATAGTAAAAT : 5049
cen1-CR4 : -----TTTCCTAGTTAGAATTTAATTATAAAATTATAAAGTGCTAATTCCTATAATTATAACACTTACTTAGTTAATAGC-TTTTTCTTTATAGTAAGGT : 4741
cen1-CR5 : -----TTTCTTAATTAAAATTTAATTATAAAATTATAAAATGCTAATTTTTATAATTATAACACTTACTTAGTTAATAGC-TTTTTTTTTATAATAAGGT : 4809
cen2-CR1 : -----TTTTTTAATTAAAATTTAATTATAAAATTATAAAATACTAATTTTTATAATTATAATACTTACTTAATTAATAGC-TTTTTCTTTATTATAAAGT : 3788
cen2-CR2 : -----TTTTTTAATTAGAATTTAATTATAAAATTATAAAGTGCTAATTCTTATAATTATAATACTTACTTAATTAATAGC-TTTTTTTTTATAGTAAAAT : 5056
cen2-CR3 : -----TTTCTTAATTAGAATTTAATTATAAAATTATAAAATACTAATTCTTATAATTATAACACTTACTTAGTTAATAGC-TTTTTCTTTATAGTAAGGT : 4891
cen2-CR4 : -----TTTCTTAATTAAAATTTAATTATAAAGTTATAAAGTATTAGTTTTTATAATTATAATACTTATTTAATTAATAGC--TTTTTTTTATAATAAAGT : 2046
cen2-CR5 : -----TTTCTTAATTAAAATTTAATTATAAAATTATAAAATATTAATTTTTATAATTATAATACTTACTTAATTAATAGC-TTTTTTTTTATAATAAAGT : 3239
cen3-CR1 : -----TTTTTTAATTAAAATTTAATTATAAAATTATAAAGTATTAATTCTTATAATTATAATACTTACTTAATTAATAGC---TTTTTTTATAATAAAGT : 3320
cen3-CR2 : -----TTTTTTAATTAAAATTTAATTATAAAATTATAAAGTGCTAATTCTTATAATTATAATACTTACTTAATTAATAGC-TTTTTCTTTATAGTAAGAT : 4875
cen3-CR3 : -----TTTTTTAATTAAAATTTAATTATAAAATTATAAAATACTAGTTTTTATAATTATAATACTTACTTAATTAATAGCTTTTTTTTTTATAATAAAGT : 4915
cen3-CR4 : -----TTTTTTAATTAAAATTTAATTATAAAATTATAAAGTATTAATTCTTATAATTATAATACTTACTTAATTAATAGC-TTTTTTTTTATAATAAGGT : 4782
cen4-CR1 : -----TTTTTTAATTAAAATTTAATTATAAAATTATAAAATGCTAATTTTTATAATTATAATACTTACTTAATTAATAGC-TTTTTCTTTATAATAAGGT : 2037
cen4-CR2 : -----TTTTTTAATTAGAATTTAATTATAAAATTATAAAATACTAATTTTTATAATTATAATACTTACTTAATTAATAGC-TTTTTTTTTATAGTAAAGT : 4957
cen4-CR3 : -----TTTTTTAATTAAAATTTAATTATAAAATTATAAAATATTAGTTTTTATAATTATAATACTTATTTAATTAATAGC--TTTTTTTTATTATAAAGT : 1489
cen4-CR4 : -----TTTTTTAATTAAAATTTAATTATAAAATTATAAAGTATTAATTCTTATAATTATAATACTTACTTAATTAATAGC-TTTTTTTTTATAATAAGGT : 5040
cen4-CR5 : -----TTTTTTAATTAAGGTTTAATTATAAAATTATAAAATACTAGTTTTTATAATTATAATACTTATTTAATTAATAGC-CTTTTTTTTATTATAAAGT : 1656
cen5-CR1 : -----TTTCTTAATTAAAATTTAATTATAAAATTATAAAGTGCTAATTCTTATAATTATAACACTTACTTAGTTAATAGC-TTTTTCTTTATAATAAGGT : 4844
cen6-CR1 : -----TTTTTTAATTAGAATTTAATTATAAAATTATAAAATGCTAATTTTTATAATTATAATACTTACTTAGTTAATAGC-TTTTTTTTTATAATAAGAT : 5059
cen7-CR1 : -----TTTTTTAATTAAAATTTAATTATAAAATTATAAAATGCTAATTTTTATAATTATAATACTTACTTAATTAATAGC-TTTTTCTTTATAATAAAAT : 4826
cen7-CR2 : -----TTTTTTAATTAGAATTTAATTATAAAATTATAAAATATTAATTCTTATAATTATAATACTTACTTAATTAATAGC-TTTTTTTTTATAATAAAGT : 4853
cen7-CR3 : TAAGATTTTTTAATTAAAATTTAATTATAAAATTATAAAATACTAATTCTTATAATTATAATACTTACTTAATTAATAGC-TTTTTTTTTATAATAAAGT : 4819
                TTT tTAaTTA aaTTTAATTATAAAaTTATAAA T  TAaTT tTATAATTATAAtACTTAcTTA TTAATAGC ttTTt TTTATa TAA gT
```

```
                        *        5220         *        5240         *        5260         *        5280         *        5300
cen1-CR1 : AAATATAGTTTAAGTAATTAGTTAATACTTAAATTAGGTATATAGTATATATAATATAATATTATTAAACTTAAAATATTTTAATAATAATAAGTATTTT : 4997
cen1-CR2 : AGGTATAGTTTAAGTAATTAGTTAATACTTAAACTAGGTATATAGTATATATAATATAATATTACTAAACTTAAAATACTTTAGTAATAATAAGTATTTT : 5104
cen1-CR3 : AGGTATAGTTTAAGTAATTAATTAATACTTAAACTAGATATATAGTATATATAATATAGTATTACTAAACTTAAAATATTTTAATAATAGTAAGTATTTT : 5149
cen1-CR4 : AGGTATAGTTTAAGTAATTAGTTAATACTTAAACTAGGTATGCAGTATATATAATATAATATTACTAAACTTAAAATACCTTAGTAATAGTAAGCATTTT : 4841
cen1-CR5 : AGGTATAGTTTAAGTAATTAGTTAATACTTAAACTAGGTATATAATATATATAATATAGTATTATTAAACTTAAAATACTTTAATAATAATAAGTATTTT : 4909
cen2-CR1 : AAGTATAGTTTAAGTAATTAATTAATATTTAAACTAAATATATAATATATATAATATAATATTATTAAATTTAAAATACCTTAATAATAATAAGTATTTT : 3888
cen2-CR2 : AGATATAGTTTAAGTAATTAGTTAATACTTAAATTAAGTATATAGTATATATAATATAATATTATTAAACTTAGAATGCCTTAGTAATAGTAAGTATTTT : 5156
cen2-CR3 : AGGTATAGTTTAAGTAATTAGTTAATACTTAAACTAAGTGTATAGTATATATAATATAGTATTACTAAACTTAAAATGCCTTAATAATAGTAAGCATTTT : 4991
cen2-CR4 : AAGTATAATTTAAGTAATT-----------AAACTAGATATATAATATATATAATATAATATTATTAAACTTAAAATGCCTTAATAATAATAAGTA-TTT : 2134
cen2-CR5 : AAGTATAATTTAAGTAATTAGTTAATACTTAAACTAGGTATATAATATATATAATATAATATTATTAAACTTAAAATGCTTTAGTAATAATAAGTATTTT : 3339
cen3-CR1 : AAGTATAGTTTAAATAATTAGTTAATATTTAAATTAAGTATATAGTATATATAATATAATATTACTAAACTTAAAATACTTTAATAATAGTAAGTATTTT : 3420
cen3-CR2 : AGGTATAGTTTAAGTAATTAATTAATACTTAAACTAGGTATATAGTATATATAATATAATATTATTAAACTTAAAATACCTTAGTAATAGTAAGTATTTT : 4975
cen3-CR3 : AAGTATAATTTAAGTAATTAGTTAATACTTAAACTAGATATATAATATATATAATATAATATTATTAAACTTAAAATACTTTAATAATAATAAGTATTTT : 5015
cen3-CR4 : AAATATAGTTTAAGTAATTAGTTAATATTTAAATTAAGTATATAATATATATAATATAATATTATTAAACTTAAAATACTTTAATAATAATAAGCATTTT : 4882
cen4-CR1 : AGGTATAGTTTAAGTAATTAGTTAATACTTAAACTAGGTATATAATATATATAATATAATATTACTAAACTTAAAATACCTTTATAATAATAAGCATTTT : 2137
cen4-CR2 : AAGTATAGTTTAAGTAATTAGTTAATACTTAAACTAGGTATATAATATATATAATATAATATTATTAAACTTAAAATGCTTTTATAATAATAAGTATTTT : 5057
cen4-CR3 : AAGTATAGTTTAAGTAATTAATTAATACTTAAACTAAATATATAATATATATAATATAATATTATTAAACTTAAAATACCTTAATAATAATAAGTATTTT : 1589
cen4-CR4 : AAGTATAGTTTAAGTAATTAGTTAATACTTAAACTAGGTATATAGTATATATAATATAGTATTACTAAACTTAAAATACTTTTATAATAATAAGTATTTT : 5140
cen4-CR5 : AAGTATAGTTTAAGTAATTAGTTAATACTTAAATTAAGTATATAATATATATAATATAATATTACTAAATTTAAAATAATTTAATAATAATAAGTATTTT : 1756
cen5-CR1 : AGGTATAGTTTAAGTAATTAGTTAATACTTAAACTAGGTGTATAGTATATATAATATAATATTA------------------------------------ : 4908
cen6-CR1 : AAGTATAGTTTAAGTAATTAGTTAATACTTAAACTAGATATATAGTATATATAATATAATATTATTAAACTTAAAATGCCTTAATAATAGTAAGTATTTT : 5159
cen7-CR1 : AAGTATAGTTTAAGTAATTAGTTAATACTTAAATTAAATATATAATATATATAATATAATATTACTAAACTTAAAATACTTTAATAATAATAAGTATTTT : 4926
cen7-CR2 : AAATATAATTTAAGTAATTAGTTAATACTTAAATTAAATATATAGTATATATAATATAATATTACTAAATTTAAAATACTTTAGTAATAGTAAGTATTTT : 4953
cen7-CR3 : AAGTATAATTTAAATAATTAGTTAATACTAAAATTAAGTATATAATATATATAATATAATATTACTAAACTTAAAATACCTTAATAATAATAAATATTTT : 4919
           A gTATAgTTTAAgTAATTagttaatacttAAA TA  TaTatA TATATATAATATAaTATTA taaacttaaaat c tta taata taagtatttt

                        *        5320         *        5340         *        5360         *        5380         *        5400
cen1-CR1 : TATATTTAATATAATATAATACTATTTAAATAAGTTAAGTTTTATAATAAGTATATTATAAAATATTATTACTTATTTATAAAAATTAATAATATTATA : 5097
cen1-CR2 : TATATTTAGTATAGTATAATACTATTTAAATAAGTTAAGTTTTATAATAAGTATATTATAAAATGCTATTACCTATTTATAAAAATTAATAGTATTATA : 5204
cen1-CR3 : TATATTTAATATAGTATAATATTATTTAAATAGGTTAAGTTTTATAATAAGTATATTATAAAATACTATTACTTATTTATAAGGATTAATAGTATTATA : 5249
cen1-CR4 : TATATCTAGTATAGTATAATATTATTTAAATAGGTTAAGTTTTATAATAAGTATGCTATAGGATGCTATTACCTATTTATAAGGATTAATAGTATTATA : 4941
cen1-CR5 : TATATTTAGTATAGTATAATACTATTTAAATAGGTTAAGTTTTATAATAAGTATACTATAAAATGCTATTACCTATTTATAAGGATTAATAGTATTATA : 5009
cen2-CR1 : TATATTTAATATAATATAATACTATTTAAATAAGTTAAGTTTTATAATAAGTATATTATAAAATACTATTATTTATTAATAAAGATTAATAGTATTATA : 3988
cen2-CR2 : TATATTTAATATAGTATAATACTATTTAAATAAGTTAAGTTTTATAATAAGTATACTATAAAATACTATTACTTATTTATAAAAATTAATAATATTATA : 5256
cen2-CR3 : TATATTTAGTATAGCATAATGCTATTTAAATAGGTTAAGTTTTATAATAAGTATACTATAGGATGCTATTGCCTATTTATAAGGATTAATAGTATTATA : 5091
cen2-CR4 : TATATTTAATATAATATAATACTATTTAAATAAGTTAAGTTTTATAATAAGTATATTATAAATTACTATTATTTATTAATAAATATTAATAATATTATA : 2234
cen2-CR5 : TATATTTAGTATAATATAATACTATTTAAATAAGTTAAGTTTTATAATAAGTATATTATAAAATATTATTATTTATTTATAAAAATTAATAGTATTATA : 3439
cen3-CR1 : TATATTTAGTATAATATAATATTATTTAAATAAGTTAAGTTTTATAATAAGTATATTATAAAATACTATTACTTATTTATAAGAATTAATAGTATTATA : 3520
cen3-CR2 : TATATTTAATATAGCATAATACTATTTAAATAAATTAAGTTTTATAATAAATATACTATAAGATATTATTACTTATTTATAAAGATTAATAATATTATA : 5075
cen3-CR3 : TATATTTAATATAATATAATACTATTTAAATAAGTTAAGTTTTATAATAAATATACTATAAAATATTATTATTTATTTATAAAAATTAATAGTATTATA : 5115
cen3-CR4 : TATATTTAATATAATATAATATTATTTAAATAAGTTAAGTTTTATAATAAGTATATTATAAAATACTATTACTTATTTATAAAAATTAATAATATTATA : 4982
cen4-CR1 : TATATTTAATATAATATAATATTATTTAAATAAGTTAAGTTTTATAATAAGTATATTATAAAATACTATTACTTATTTATAAAAATTAATAATATTATA : 2237
cen4-CR2 : TATATTTAATATAGTATAATACTATTTAAATAAGTTAAGTTTTATAATAAGTATACTATAAGATATTATTATTTATTTATAAAAGTTAATAGTATTATA : 5157
cen4-CR3 : TATATTTAATATAATATAATACTATTTAAATAAATTAAGTTTTATAATAAGTATATTATAAAATACTATTACTTATTTATAAAAATAAATAGTATTATA : 1689
cen4-CR4 : TATATTTAATATAATATAATATTATTTAAATAAGTTAAGTTTTATAATAAGTATATTATAAAATGCTATTATTTATTTATAAGAATTAATAATATTATA : 5240
cen4-CR5 : TATATTTAATATAATATAATATTATTTTAATAAATTAAGTTTTATTATAAATATATTATAAAATACTATTACTTATTTATAAAAATTAATAATATTATA : 1856
cen5-CR1 : --------------------------------------------------------------------------------------------------- : -
cen6-CR1 : TATATTTAATATAATATAATACTATTTAAATAAGTTAAGTTTTATAATAAGTATATTATAAAATACTATTACTTATTTATAAGGATTAATAATATTATA : 5259
cen7-CR1 : TATATTTAGTATAGTATAATATTATTTAAATAGGTTAAGTTTTATAATAAGTATATTATAAAATACTATTACTTATTTATAAGAATTAATAGTATTATA : 5026
cen7-CR2 : TATATTTAATATAATATAATACTATTTAAATAAGTTAAGTTTTATAATAAGTATATTATAAAATACTATTACTTATTTATAAAAATTAATAATATTATA : 5053
cen7-CR3 : TATATTTAATATAATATAATATTATTTAAATAAGTTAAGTTTTATAATAAATATATTATAAAATACTATTATTTATTTATAAAAATTAATAGTATTATA : 5019
           tatattta tata tataata tatttaaata gttaagttttataataagtata tataaaat  tatta ttatttataa  attaata tattata
```

```
                    *       5420         *       5440         *       5460         *       5480         *       5500
cen1-CR1 : TTAATAGTATATTTTAATATATTAGGTTTAATATAGATTTAAGCTATAAAGTTAAAATATATAAAAATAATTTATTTATAAAATTTCTTTTTT-AATATT : 5196
cen1-CR2 : TTAATAATATACTTTAATGCATTAGGTTTAATATAAATTTAGGCTATAGGGTTAAAATATATAAGAATAGGTTATTTATAAAATTTCTTTTTT-AATATC : 5303
cen1-CR3 : CTAATAGTATACTTTAATATATTAGATTTAATATAAATTTAGGCTATAAGGTTAAAATATATAAAAATAGGTTATTTATAAAATTTCTTTTTT-AATATC : 5348
cen1-CR4 : TTAATAGCATACTTTAATATATTAGGTTTAATATAAATTTAGGCTGCAGGGTTAAAATGCATAAGAATAGGTTATTTATAAAATTTCTTTTTT-AATATT : 5040
cen1-CR5 : TTAATAAATACTTTAATATATTAGGTTTAATATAAATTTAGGCTGCAGGGTTAAAATGCATAAGAATAAGTTATTTATAAAATTTCTTTTTT-AATATC : 5108
cen2-CR1 : TTAATAAATACTTTAATATATTAAGTTTAATATAAATTTAAGTTATTAAATTAAAATATATAAAAATAAGTTATTTATAAAATTTCTTTTTT-AATATT : 4087
cen2-CR2 : TTAATAATATATTTTAATATATTAGGTTTAATATAAATTTAGGCTATAAAGTTAAAATATATAAGAATAGGTTATTTATAAAATTTCTTTTTT-AATATT : 5355
cen2-CR3 : TTAATAGCATATTTTATTATATTAGGTTTAATATAAATTTAGGCTGCAGGGTTAAAATATATAAGAATAGGTTATTTATAAAATTTCTTTTTT-AATATC : 5190
cen2-CR4 : TTAATAATATACTTTAATATATTAAATTTAATATAAATTTAAGTTATAAAGTTAAAATATATAAAAATAAATTATTTATAAAATTTCTTTTTT-AATATT : 2333
cen2-CR5 : TTAATAATATACTTTAATATATTAAGTTTAATATAAATTTAAGCTATAAGGTTAAAATATATAAAAATAAATTATTTATAAAATTTCTTTTTT-AATATT : 3538
cen3-CR1 : TTAATAATATACTTTAATATATTAAGTTTAATATAAATTTAGGCTATAAGGTTAAAATATATAAAAATAAGTTATTTATAAAATTTCTTTTTT-AATATT : 3619
cen3-CR2 : TTAATAGTATACTTTAATATATTAGGTTTAATATAAATTTAGGCTATAAGGTTAAAATATATAAAAATAGGTTATTTATAAAAATTTTTTTTT-AATATT : 5174
cen3-CR3 : CTAATAATATATTTTAATATATTAAGTTTAATATAAATTTAAGTTATAAGGTTAAAATATATAAAAATAAGTTATTTATAAAATTTCTTTTTT-AATATT : 5214
cen3-CR4 : TTAATAGTATATTTTAATATATTAAGTTTAATATAAATTTAGGCTATAAGGTTAAAATATATAAAAATAAATTATTTATAAAATTTCTTTTTT-AATATT : 5081
cen4-CR1 : TTAATAATATACTTTAATATATTAAGTTTAATATAAATTTAAGCTATAAGGTTAAAATATATAAAAATAAGTTATTTATAAAATTTCTTTTTT-AATATT : 2336
cen4-CR2 : TTAATAATATATTTTAATATATTAAGTTTAATATAAATTTAAGTTATAAGGTTAAAATATATAAAAATAAGTTATTTATAAAATTTCTTTTTT-AATATT : 5256
cen4-CR3 : TTAATAATATACTTTAATATATTAAGTTTAATATAAATTTAGGTTATAAAGTTAAAATATATAAAAATATATTATTTATAAAATTTCTTTTTTAAATATT : 1789
cen4-CR4 : CTAATAGTATATTTTAATATATTAGGTTTAATATAAATTTAAGCTATAAAGTTAAAATATATAAAAATAGGTTATTTATAAAATTTCTTTTTT-AATATT : 5339
cen4-CR5 : TTAATAATATACTTTAATATATTAAGTTTAATATAAATTTATACTATAAGGTTAAAATATATAAAAATAAGTTATTTATAAAATTTCTTTTTT-TATATT : 1955
cen5-CR1 : ---------------------------------------------------------------------------------------------------- :    -
cen6-CR1 : TTAATAATATACTTTAATATATTAAATTTAATATAAATTTAAGCTATAAGGTTAAAATATATAAAAATAAGTTATTTATAAAATTTCTTTTTT-AATATT : 5358
cen7-CR1 : TTAATAATATACTTTAATATATTAAGTTTAATATAAATTTAAGCTATAAGATTAAAATATATAAAAATAAGTTATTTATAAAATTTCTTTTTT-AATATT : 5125
cen7-CR2 : TTAATAATATACTTTAATATATTAAGTTTAATATAAATTTAAGCTATAAGGTTAAAATATATAAAAATAAGTTATTTATAAAATTTCTTTTTT-AATATT : 5152
cen7-CR3 : TTAATAATATATTTTAATATATTAAGTTTAATATAAATTTAAGCTATAAGATTAAAATATATAAAAATAAGTTATTTATAAAATTTCTTTTTT-AATATT : 5118
           ttaata tata tttaatatatta gtttaatataaattta g tataa gttaaaatatataa aata  ttatttataaaatttctttttt aatatt

                    *       5520         *       5540         *       5560         *       5580         *       5600
cen1-CR1 : TTAAATACTTTATATTTAATTAATATAAATTAAAACTTTTAATTCTTTTTATTATTAATTAAGTTAATAAAAGGTATTATAATTTTATTATAATTATAAA : 5296
cen1-CR2 : TTAAATACTTTATATTTAACTAATATAAATTAAAATTTCTAATCCTTTTTACTACTAGTTAAGTTAATAAAAGGGACTATAATTTTACTATAATTATAAA : 5403
cen1-CR3 : TTAAATACTTTATATTTAATTAATATAAATTAAAATTTCTAATTCTTTTTACTATTAGTTAAATTAGTAAAAGGGACTGTAATCTTATTATAATTATAAA : 5448
cen1-CR4 : TTAAATGCTTTATATTTAACTAATATAAATTAAAATTTCTAATCCTTCTTACTACTAATTAAATTAGTAAAAGGCACTATAATTTTACTATAATTATAAA : 5140
cen1-CR5 : TTAAATACTTTATATTTAACTAATATAAATTAAAATTTTTAATCTTTTTTATTACTAGTTAAGTTAATAAAAGGCATTATAATCTTACTATAATTATAAA : 5208
cen2-CR1 : TTAAATACTTTATATTTAATTAATATAAATTAAAACTTTTAATTCTTTTTATTATTAGTTAAATTAATAAAAAGTATTATAATTTTATTATAATTATAAA : 4187
cen2-CR2 : TTAAATGCTTTATATTTAACTAATATAAATTAGAATTTCTAATCCTTTTTATTATTAATTAGGTTAATAAAAGGCACTATAATTTTACTATAATTATAAA : 5455
cen2-CR3 : TTAAATGCTTTATATTTAACTAATATAAATTAAAATTTCTAATCCTTCTTATTACTAATTAGGTTAGTAAAAGGCATTATAATCTTACTATAATTATAAA : 5290
cen2-CR4 : TTAAATACTTTATATTTAATTAATATATATTAAAACTTTTAATTTTTCTTATTATTAATTTAATTAATAAAAAGTATTATAATTTTATTATAATTATAAA : 2433
cen2-CR5 : TTAAATACTTTATATTTAATTAATATAAATTAAAACTTTTAATCTTTTTTATTATTAATTAGGTTAATAAAAGGTATTATAATCTTATTATAATTATAAA : 3638
cen3-CR1 : TTAAATACTTTATATTTAATTAATATAAATTAAAACTTTTAATCTTTTTTATTATTAATTAAGTTAGTAAAAGGTATTATAATTTTATTATAATTATAAA : 3719
cen3-CR2 : TTAAATACTTTATATTTAATTAATATAAATTAAAACTTTTAATTCTTCTTACTATTAGTTAGGTTAGTAAAGGGCATTATAATCTTATTATAATTATAAA : 5274
cen3-CR3 : TTAAATACTTTATATTTAATTAATATAAATTAAAACTTTTAATTCTTTTTATTATTAATTAAATTAATAAAAGGTATTATAATTTTACTATAATTATAAA : 5314
cen3-CR4 : TTAAATACTTTATATTTAATTAATATAAATTAAAACTTTTAATTCTTTTTACTACTAGTTAAATTAATAAAAGGTATTATAATCTTATTATAATTATAAA : 5181
cen4-CR1 : TTAAATATTTTATATTTAACTAATATAAATTAAAATTTTTAATTTTTTTTACTTTTAATTAAGTTAGTAAAAGGTATTATAATTTTACTATAATTATAAA : 2436
cen4-CR2 : TTAAATACTTTATATTTAATTAATATAAATTAAAACTTTTAATTTTTCTTACTACTAATTAAATTAGTAAAAGGCATTATAATCTTACTATAATTATAAA : 5356
cen4-CR3 : TTAAAAACTTTATATTTAATTAATATAAATTAAAATTTTTAATTCTTTTTATTATTAATTAAGTTAATAAAAGGTATTATAATTTTATTATAATTATAAA : 1889
cen4-CR4 : TTAAATGCTTTATATTTAATTAATATAAATTAGAACTTTTAATCCTTTTTACTATTAGTTAAATTAATAAGGGGTATTATAATTTTATTATAATTATAAA : 5439
cen4-CR5 : TTAAATACTTTATATTTAATTAATATAAATTAAAACTTTTAATTTTTTTTATTATTAATTAAATTAGTAAAAGATATTATAATTTTATTATAATTATAAA : 2055
cen5-CR1 : ---------------------------------------------------------------------------------------------------- :    -
cen6-CR1 : TTAAATACTTTATATTTAATTAATATAAATTAAAATTTCTAATTCTTTTTATTACTAATTAGGTTAATAAAAGGCATTATAATCTTATTATAATTATAAA : 5458
cen7-CR1 : TTAAATACTTTATATTTAATTAATATAAATTAAAACTTTTAATTCTTTTTACTATTAATTAAGTTAATAAAAAGTATTATAATTTTATTATAATTATAAA : 5225
cen7-CR2 : TTAAATGCTTTATATTTAATTAATATAAATTAGAATTTTTAATCTTTTTTATTATTAATTAAGTTAGTAAAAGGTATTATAATCTTACTATAATTATAAA : 5252
cen7-CR3 : TTAAATGCTTTATATTTAATTAATATAAATTAAAACTTTTAATTTTTTTTATTATTAATTAAGTTAGTAAAAGGTATTATAATTTTATTATAACTATAAA : 5218
           ttaaat ctttatatttaa taatataaattaaaa tt taat  tt tta ta ta tta  tta taaaagg attataat tta tataattataaa
```

```
                        *      5620          *      5640          *      5660          *      5680          *      5700
cen1-CR1 : TAAAATATTAATAAAAATTATAAAATTTAAGAAATCTTAATATAGCTTTAATAAAGGTTAATTTTAGCTAGTTTATAATAACTTTAATATAATCTTTTTT : 5396
cen1-CR2 : TAAAATACTAATAAAAATTATAAAATTTAAAAAATCCTAATATAGCTTTAATAAAGGTTAGTTTTAGCTAATTTATAATAACTTTAATATAATTTTTTTT : 5503
cen1-CR3 : TAAAATATTAATAAAAATTATAAAATTTAAGAAATCCTAATATAGCTTTAATAAAGGTTAGTTCTAGCTAATTTATAATAACTTTAATATAATCTCTTTT : 5548
cen1-CR4 : TAAAACGCTAATAAAAATTATAAAATCTAAAAAATCTTAATATAGCTTTAATAGAGGTTAATTCTAGCTAGTTTATAATAACTTTAATATAATCTTTTTT : 5240
cen1-CR5 : TAAAATGCTAATAAAAATTATAAAATTTAAAAAATCTTAATATAGCTTTAATAAAGATTAGTTTTAGCTAGTTTATAATAACTTTAATATAATCTTTTTC : 5308
cen2-CR1 : TAAAATACTAATAAAAATTATAAAATTTAAAAAATCTTAATATAGCTTTAATAAAAGTTAATTTTAGCTAGTTTATAATAGCTTTAATATAATTATTTTT : 4287
cen2-CR2 : TAAAATATTAATAAAAATTATAAAATTTAAAAAATCTTAATATAGCTTTAATAAAGGTTAGTTTTAGCTAATTTATAATAATTTTAATATAATCTCTTTT : 5555
cen2-CR3 : TAAAATACTAATAAAAATTATAAAATTTAAGAAATCTTAATATAGCTTTAATAAAGGTTAGTTTTAGCTAGTTTATAATAACTTTAATATAATCTCTTTC : 5390
cen2-CR4 : TAAAATATTAATAAAAATTATAAAATTTAAAAAATCTTAATATAGCTTTAATAAAGGTTAATTTTAGCTAGTTTATAATAACTTTAATATAATCTTTTTT : 2533
cen2-CR5 : TAAAATACTAATAAAAATTATAAAATTTAAAAAATCTTAATATTACTTTAATAAAAATTAGTTTTAGCTAATTTATAATAACTTTAATATAATTTTTTTT : 3738
cen3-CR1 : TAAAATATTAATAAAAATTATAAAATTTAAAAAATCTTAATATAGCTTTAATAAAAGTTAATTTTAGCTAGTTTATAATAACTTTAATATAATCTTTTTT : 3819
cen3-CR2 : TAAAACACTAATAAAAATTATAAAATTTAAAAAATCTTAATATAGCTTTAATAAAGGTTAGTTTTAGCTAGTTTATAATAACTTTAATATATTCTCTTTT : 5374
cen3-CR3 : TTAAATATTAATAAA--------------------------------------------------------TTTATAATAACTTTAATATAATCTTGCTC : 5358
cen3-CR4 : TAAAATATTAATAAAAATTATAAAATTTAAAAAATCCTAATATAGCTTTAATAAAAGTTAGTTTTAGTTAATTTATAATAACTTTAATATAATCTTTTTT : 5281
cen4-CR1 : TAAAATACTAATAAAAATTATAAAATTTAAGAAATCTTAATATAGCTTTAATAAAGGTTAGTTTTAGCTAGTTTATAATAACTTTAATATAATCTTTTTT : 2536
cen4-CR2 : TAAAATATTAATAAAAATTATAAAATTTAA-AAATCTTAATATAGCTTTAATAAAGGTTAATTTTAGCTAGTTTATAATAACTTTAATATAATCTCTTTT : 5455
cen4-CR3 : TAAAATATTAATAAAATTTATAAAATTTAAAAAATCCTAATATAGCTTTAATAAAAGTTAATTTTAACTAGTTTATAATAACTTTAATATAATCTTTTTT : 1989
cen4-CR4 : TAAAATATTAATTAAAATTATAAAATTTAAAAAATCCTAATATAGCTTTAATAAAGGTTAGTTTTAACTAGTTTATAATAACTTTAATATAATTTCTTTT : 5539
cen4-CR5 : TAAAATATTAATAAAAATTATAAAATTTAAAAAATCTTAATATAGCTTTAATAAAGGTTAATTTTAATTAGTTTATAATAACTTTAATATAATCTCTTTT : 2155
cen5-CR1 : ---------------------------------------------------------------------------------------------------- :    -
cen6-CR1 : TAAAATACTAATAAAAATTATAAAATTTAAAAAATCCTAATATAGCTTTAATAAAAGTTAATTTTAGCTAGTTTATAATAACTTTAATATAATCTCTTTT : 5558
cen7-CR1 : TAAAATACTAATAAAAATTATAAAATTTAAGAAATCTTAATACTACTTTAATAAAGGTTAATTCTAGCTAGTTTATAATAACTTTAATATAATCTCTTTT : 5325
cen7-CR2 : TAAAATATTAATAAAAATTATAAAATTTAAAAAATCTTAATATAGCTTTAATAAAGGTTAGTTTTAACTAGTTTATAATAACTTTAATATAATTTTTTTT : 5352
cen7-CR3 : TAAAATATTAATAAAAATTATAAAATTTAAAAAATCTTAATATAGCTTTAATAAAGGTTAATTTTAGCTAGTTTATAATAACTTTAATATAAT-TTTTTT : 5317
           taaaata taataaaaattataaaatttaa aaatc taatatagctttaataaa gtta tttta cta tttataataactttaatataat t tttt

                        *      5720          *      5740          *      5760          *      5780          *      5800
cen1-CR1 : TATTTATACTTTAATAAGGCTAATAATAAATTTTAAATATAAAATAATATTAATATAAAATTTATATTTCTTTAAGTTAATATAAAGACTATATTATTAA : 5496
cen1-CR2 : TATTTACACTTTAGTAAAGCTAATAATAAATCCTAAATATAAGATAGTATTAATATAAAATTTACATTTCTTTAAATTAATATAAAGACTATATTATTAA : 5603
cen1-CR3 : TATTTATACCTTAGCAAGGCTAATAATAAATCCTAAATATAAAACAGTATTAATATAAAATTTATATTTTTTTAAATTAATATAAAGACTATGCTATTAA : 5648
cen1-CR4 : TATTTATACCTTAGCAGAGCTAATAATAAATCCTAAATATAAAATAGTATTAATATAAAATTTACATTTCTTTAAATTAATATAAAGACTATGCTGTTAA : 5340
cen1-CR5 : TATCTGCATTTTAGTAAGGCTAATAATAAATCCTAAATATAAAATAGTATTAATATAAAATTTATATTTCTTTAAATTAATATAAAGACTATACTATTAA : 5408
cen2-CR1 : TATTTATATTTTAATAAGGCTAATAATAAATCCTAAATATAAAATAGTATTAATATAAAATTTATATTTTTTTAAGTTAATATAAAGACTATATTATTAA : 4387
cen2-CR2 : TATTTATACTTTAGCAGGGCTAATAATAAATCCTAAATATAAAATAATATTAATATAAAATTTATATTTCTTTAAGTTAATATAAAGACTATACTATTAA : 5655
cen2-CR3 : TATCTGCACTTTAGCAGGGCTAATAATAAATCCTAAATATAAGACAGTATTAATATAAAATTTGCATTTTTTTAGGTTAATATAAAGACTATACTATTAA : 5490
cen2-CR4 : TATTTATATTTTATTAAGGTTAATAATAAATCCTAAATATAAAATAATATTAATATAAAATTTATATTTTTTTAAATTAATATATAAATTATATTACTAA : 2633
cen2-CR5 : TATTTATACTTTAATAAGGCTAATAATAAATTCTAAATATAAAATAGTATTAATATAAAATTTATATTTTTTTAAGTTAATATAAAGACTATATTATTAA : 3838
cen3-CR1 : TATTTATACTTTAATAAGGCTAATAATAAATTTTAAATATAAAATAATATTAATATAAAATTTATATTTTTTTAAGTTAATATAAAGACTATATTATTAA : 3919
cen3-CR2 : TATTTATACTTTAGTAAGGCTAATAATAAATTCTAAATATAAAATAGTATTAATATAAAATTTATATTTCTTTAAGTTAATATAAAGACTATGCTATTAA : 5474
cen3-CR3 : GACTGG----------AGGCTGATATAAGGCTCC---------------------------------------------------AGA------------ : 5385
cen3-CR4 : TATTTATACTTTAATAAGGCTAATAATAAATCTTAAATATAAAATAGTATTAATATAAAATTTATATTTTTTTAAGTTAATATAAAGACTATATTATTAA : 5381
cen4-CR1 : TATTTATATTTTAATAAAGCTAATAATAAATTCTAAATATAAAATAGTATTAATATAAAATTTATATTTTTTTAAGTTAATATAAAGACTATATTATTAA : 2636
cen4-CR2 : TATTTATACTTTAATAAGGCTAATAATAAATCCTAAATATAAAATAATATTAATATAAAATTTATATTTTTTTAAGTTAATATAAAGACTATATTATTAA : 5555
cen4-CR3 : TATTTATATTTTATTAAAGTTAATAATAAATTTTAAATATAAAATAGTATTAATATAAAATTTATATTTTTTTAAATTAATATATAAACTATATTATTAA : 2089
cen4-CR4 : TATTTATACTTTAGCAGGGTTAATAATAAATCCTAAATATAAAATAGTATTAATATAAAATTTATATTTTTTTAAGTTAATATAAAAACTATATTATTAA : 5639
cen4-CR5 : TATTTATACTTTATTAAAGCTAATAATAAATCCTAAATATAAAATAATATTAATATAAAATTTAGATTTTTTTTAATTAATATATAAATTATATTACTAA : 2255
cen5-CR1 : ---------------------------------------------------------------------------------------------------- :    -
cen6-CR1 : TATTTATACTTTAATAAGGCTAATAATAAATTCTAAATATAAAATAGTATTAATATAAAATTTATATTTCTTTAAGTTAATATAAAGACTATATTATTAA : 5658
cen7-CR1 : TATTTATACTTTAATAAGGCTAATAATAAATCCTAAATATAAAATAATATTAATATAAAATTTATATTTTTTTAAGTTAATATAAAGACTATACTATTAA : 5425
cen7-CR2 : TATTTATATTTTAATAAGGCTAATAATAAATTCTAAATATAAAATAATATTAATATAAAATTTATATTTTTTTAAGTTAATATAAAAACTATACTATTAA : 5452
cen7-CR3 : TATTTATACTTTATTAAGGCTAATAATAAATCCTAAATATAAAATAATATTAATATAAAATTTATATTTTTTTAAATTAATATATAAATTATATTATTAA : 5417
           tatttata ttta  aa gctaataataaat ctaaatataaaata tattaatataaaattta attt tttaa ttaatata a actata tattaa
```

```
                          *       5820          *       5840          *       5860          *       5880          *       5900
cen1-CR1 : AGTTATTTAAGAATTATATAAATATATCTTTTATACTTTTACTTATTTTAATTATAAATTAAAATATTATTTAAATAAATAATATATATAATATTAATAA : 5596
cen1-CR2 : AGTTATTTAAGGACTATATAAATATATCCTTTATACTCTTGCTTATTTTAATTATAAATTAAAATATTATTTAAATAGATAATATATATAATATTAATAA : 5703
cen1-CR3 : AGTTATTTAAGGACTATATAAATATATCCTTTATACTCTTGCTTATTTTAATTATAAATTAAAATATTATTTAAATAAATAATATATATAATATTAATAA : 5748
cen1-CR4 : AGTTATTTAAGGATTATATAAATATATCCTTTATACTCTTACTTATTCTAACTGTAAATTAAAATATTATCTAAATAAATAATATATATAATATTAATAA : 5440
cen1-CR5 : AGTTATTTAAAGATTATATAAATATATCCTTTATACTTTTACTTATTCTAACTATAAATTAAAATATTATTTAAATAAATAATATATATAATATTAATAA : 5508
cen2-CR1 : AGTTATTTAAGGATTATATAAATATATCCTTTATACTTTTACTTATTCTAGCTATAAATTAAAATATTATTTAAATAAATAATATATATAATATTAATAA : 4487
cen2-CR2 : AGTTATTTAAGGATTATTTAAATATATCCTTTATACTTTTACTTATTTTAACTATAGATTAAAATATTATTTAAATAAATAATATATATAATATTAATAA : 5755
cen2-CR3 : AGTTATTTAAGGACTATCTAAATATATTCTTTATGCTCTTGCTTATTCTAACTGTAAATTAAAATGTTATTTAAATAAATAATATATATAATATTAATAA : 5590
cen2-CR4 : AGTTATTTAAGAACTATATAAATATAATTTTTATACTTTTACTTATTTTAGCTATAAATTAAAATATTATTTAAATAAATAATATATATTATATTAATAA : 2733
cen2-CR5 : AGTTATTTAAGGACTATTTAAATATATTTTTTATGCTTTTATTTATTTTAACTATAAATTAAAATATTATTTAAATAAATAATATATATAATATTAATAA : 3938
cen3-CR1 : AGTTATTTAAAAACTATATAAATATATTTTTTATACTTTTACTTATTTTAATTATAAATTAAAATATTATTTAAATAAATAATATATATAATATTAATAA : 4019
cen3-CR2 : AGTTATTTAAGGATTATATAAATATATCCTTTATGCTTTTACTTATTTTAACTATAAATTAAAATATTATTTAAATAAATAATATATATAATATTAATAA : 5574
cen3-CR3 : ---------------------------------------------------------------------------------------------------- :    -
cen3-CR4 : AGTTATTTAAGGACTATATAAATATATTTTTTATACTTTTACTTTTTTTAATTATAAATTAAAATATTATTTAAATAAATAATATATATAATATTAATAA : 5481
cen4-CR1 : AGTTATTTAAGGACTATATAAATATATCCTTTATACTCTTACTTATTTTAATTATAAATTAAAATATTATTTAAATAAATAATATATATAATATTAATAA : 2736
cen4-CR2 : AGTTATTTAAGGATTATATAAATATATTTTTTATACTTTTACTTATTTTAATTATAAATTAAAATATTATTTAAATAAATAATATATATAATATTAATAA : 5655
cen4-CR3 : AGTTATTTAAGGATTATATAAATATAATTTTTATACTTTTACTTATTTTAATTATAATTTAAAATATTATTTAAATAAATAATATATATAATATTAATAA : 2189
cen4-CR4 : AGTTATTTAAGAACTATATAAATATATTCTTTATGCTTTTGCTTATTTTAATTATAAATTAAAATATTATTTAAATAAATAATATATATAATATTAATAA : 5739
cen4-CR5 : AGTTATTTAAAAATTATATAAATATAATTTTTATACTTTTACTTATTTTAGCTATAAATTAAAATATTATTTAAATAAATAATATATATAATATTAATAA : 2355
cen5-CR1 : ---------------------------------------------------------------------------------------------------- :    -
cen6-CR1 : AGTTATTTAAGGACTATTTAAATATATTCTTTATACTTTTACTTATTTTAATTATAAATTAAAATATTATTTAAATAAATAATATATATAATATTAATAA : 5758
cen7-CR1 : AGTTATTTAAAGACTATTTAAATATATTTTTTATACTTTTACTTATTTTAACTATAAATTAAAATATTATTTAAATAAATAATATATATAATATTAATAA : 5525
cen7-CR2 : AGTTATTTAAGGACTATATAAATATATCCTTTATACTTTTACTTATTTTAATTATAAATTAAAATATTATTTAAATAAATAATATATATAATATTAATAA : 5552
cen7-CR3 : AGTTATTTAAAAACTATATAAATATATTTTTTATACTTTTACTTATTTTAGCTATAAATTAAAATATTATTTAAATAAATAATATATATAATATTAATAA : 5517
            agttatttaa  a tat taaatatat  tttat ct tt cttatt ta  tataaattaaaatattatttaaataaataatatatataatattaataa

                          *       5920          *       5940          *       5960          *       5980          *       6000
cen1-CR1 : GTTTTATAAAAATAAAGTTAATATATATTTACTAAATAACTAAGGTATTTATAAAGCTAAATAATAAAACCTAGTATTAAAAAAGACTATAATAATATTA : 5696
cen1-CR2 : GTTTTATAAAAATAAAGTTAATATATATTTATTAAGTAGCTAGGGTATTTATAAGGCTAAATAGTAAAACCTAGTATTAAAAAAGACTATAATAATATTA : 5803
cen1-CR3 : GTCTTATAAGAATAAAGTTAATATATATCTATTAGGTAGCTAAGGCATTTATAAGGCTAAATAGTAAAACCTAGTATTAAAAGAACTATAATAATATTA : 5848
cen1-CR4 : GTCTTATAAGAATAAAGTTAATATATATCTGCTAGGTAGCTAAGGTATTTATAAGGCTAAATAGTAAAACCTAGTACTAAAAAAGACTATAATAATATTA : 5540
cen1-CR5 : GTCTTATAAAAATAAAGTTAATATATATCTGCTAAGTAGCTAAGGTATTTATAAGGCTAAATAGTAAAATCTAGTATTAAAAGAGACTATAATAATATTA : 5608
cen2-CR1 : GTTTTATAAAAATAAAGTTAATATATATTTATTAAGTAACTAGGGTATTTATAAAGCTAAGTAATAAAACTTAATACTAAAAGAGATTATAATAATATTA : 4587
cen2-CR2 : GTTTTATAAGGATAAAGTTAATATATATTTACTAAGTAGCTAGGGTATTTATAAGGCTAAATAGTAAAACCTAGTACTAAAAGAGATTATAATAATATTA : 5855
cen2-CR3 : GTTTTATAAGAATAGAGTTAATATATATCTGCTAGGTGGCTAGGGCATTTATAAGGCTAAATAGTAAAACCTAGTATTAAAAAAGACTATAATAATATTA : 5690
cen2-CR4 : GTTTTATAAAAATAAAGTTAATATATATTTATTAAGTTATTAAGGTATTTATAAAGCTAAATAATAAAACCTAGTATTAAAAAAAATTATAAAATATTA : 2833
cen2-CR5 : GTTTTATAAAAATAAAGTTAATATATATTTACTAAATAGCTAGGGTATTTATAAGGCTAAATAGTAAAACCTAGTATTAAAAAAAACTATAATAATATTA : 4038
cen3-CR1 : GTTTTATAAAAATAAAGTTAATATATATTTATTAAGTAGCTAAGGTATTTATAAAGCTAAATAATAAAACCTAATATTAAAAAAAACTATAATAATATTA : 4119
cen3-CR2 : GTCTTATAAGAATAAAGTTAATATAAATTTACTAAATAGCTAAGGCATTTATAAGGCTAAATAGTAAAACCTAATACTAAAAGAGATTATAATAATATTA : 5674
cen3-CR3 : -------------------------ATGCGGACTCGGCGCCTGAGG------------------------------------------------ACATTA : 5412
cen3-CR4 : GTCTTATAAAAATAAAGTTAATATATATTTATTAAATAGCTAGGGTATTTATAAGGCTAAATAATAAAACTTAATATTAAAAGAGATTATAATAATATTA : 5581
cen4-CR1 : GTTTTATAAAAATAAAGTTAATATATATTTATTAAATAGCTAAGGTATTTATAAGGCTAAATAGTAAAACCTAGTATTAAAAAAAATTATAATAATATTA : 2836
cen4-CR2 : GTTTTATAAAAATAAAGTTAATATATATTTACTAAGTAGCTAGGGTATTTATAAAGCTAAATAGTAAAATTTAATATTAAAAAAAACTATAATAATATTA : 5755
cen4-CR3 : GTTTTATAAAAATAAAGTTAATATATATTTACTAAATTATTAAAGTATTTATAAAGCTAAATAATAAAACCTAATATTAAAAAAGACTATAATAATATTA : 2289
cen4-CR4 : GTCTTATAAGAATAAAGTTAATATATATTTACTAAGTGGCTAGAGTATTTATAAGGCTAAATAGTAAAACCTAGTATTAAAAAAGATTATAATAATATTA : 5839
cen4-CR5 : GTTTTATAAAAATAAAGTTAATATATATTTATTAAGTTATTAAGGTATTTATAAAGCTAAATAATAAAACCTAATATTAAAAAAGATTATAATAATATTA : 2455
cen5-CR1 : ---------------------------------------------------------------------------------------------------- :    -
cen6-CR1 : GTTTTATAAAAATAAAGTTAATATATATTTATTAAGTAGCTAGAGTATTTATAAAGCTAAATAATAAAACCTAGTATTAAAAAAAACTATAATAATATTA : 5858
cen7-CR1 : GTTTTATAAAAATAAAGTTAATATATATTTACTAAATAGCTAAGGTATTTATAAAGCTAAATAGTAAAACTTAGTATTAAAAAAAACTATAATAATATTA : 5625
cen7-CR2 : GTTTTATAAAAATAAAGTTAATATATATTTATTAAGTAGCTAAGGTATTTATAAAACTAAATAATAAAATTTAATATTAAAAAAGACTATAATAATATTA : 5652
cen7-CR3 : GTCTTATAAAAATAAAGTTAATATATATTTATTAAATAGCTAAGGTATTTATAAAGCTAAATAGTAAAACTTAATATTAAAAAAAACTATAATAATATTA : 5617
            gt ttataa aataaagttaatatatat ta taa t  cta ggtatttataa gctaaata taaaac ta ta taaaa a a tataataatatta
```

```
                    *      6020         *      6040         *      6060         *      6080         *      6100
cen1-CR1 : CTATATTATTTTTTTTTTATTT-----CCTTTTTATATATAAATATA-ATAATAAGTATTTTTTAAGTTAAGTTTTATAAAGATTTTTACTTTTTATAAT : 5790
cen1-CR2 : CTATACTATCTTTTCTTTATCT-----CCTTTTTATATATAAATATA-ATAATAAGCATTTTTTAAGTTAAGTTTTATAAAGATTTTTACTTTTTATAAT : 5897
cen1-CR3 : CTATACTGTCTTTTCCTTATCT-----CCTTTTTATATATAAATATA-ATAGTAGGCATTTTTTAGGTTAAGTTTTATAAAGATTTTTACTTTTTATAAT : 5942
cen1-CR4 : CTATATTATCTTTTTCTTATCT-----CCTTCTTATATATAAATATA-ATAGTAGGTATCTTTTAAGTTAAGTTTTATAAAGATTTTTACTCCTTATAAT : 5634
cen1-CR5 : CTATACTATTTTTTTTTTATCT-----CCTTTTTATATATAAATATA-ATAGTAAGTATTTTTTAAGTTAAGTTTTATAAAAATTTTTACTTTTTATAAT : 5702
cen2-CR1 : TTATATTATATTTTTTTTATTT-----TCTTTTTATATATAAATATA-ATAATAAGTATTTTTTAAGTTAAGTTTTATAAAGATTTTTACTTTTTATAAT : 4681
cen2-CR2 : CTATACTATCTTTTCTTTATCT-----CCTTTTTATATATAAATATA-ATAATAGGCATTTTTTAAGTTAAGTTTTATAAAGATTTTTACTTTTTATAAT : 5949
cen2-CR3 : CTATACTATCTTTTCCTTATCT-----CCTTCTTATATATAAATATA-ATAGTAAGTATTTTTTAAGTTAAGTTTTATAAAGATTTTTACTTTTTATAAT : 5784
cen2-CR4 : CTATATTATTTTTTTCTTATTTTAATATATATATATATATATATATATATAATAAGTATTTTTTAAATTAAGTTTTATAAAAATCTTTACTTTTTATAAT : 2933
cen2-CR5 : TTATATTATCTTTTTTTTATTT-----TTTTTTTATATATAAATATA-ATAATAGGCATTTTTTAAATTAAGTTTTATAAAAATTTTTACTTTTTATAAT : 4132
cen3-CR1 : TTATACTATCTTTTTTTTATCT-----TTTTTTTATATATAAATATA-ATAATAAGCATTTTTTAAGTTAAGTTTTATAAAGATTTTTACTTTTTATAAT : 4213
cen3-CR2 : CTATACTATCTTTTCCTTATCT-----CCTTTTTATATATAAATATA-ATAATAGGCATCTTTTAGGTTAAGTTTTATAAAGATTTTTACTTTTTATAAT : 5768
cen3-CR3 : TT-------------------------------------------------------------------------------------------------- : 5414
cen3-CR4 : TTATATTATCTTTTTTTTATTT-----TCTTTTTATATATAAATATA-ATAATAAGTAT-TTTTAAGTTAAGTTTTATAAAGATTTTTACTTTTTATAAT : 5674
cen4-CR1 : TTATATTATTTTTTTTTTATTT-----TCTTTTTATATATAAATATA-ATAATAAGCATTTTTTAAATTAAGTTTTATAAAAAT-TTTACTTTTTATAAT : 2929
cen4-CR2 : TTATAT----TTTTTTTTATCT-----CTTTTTTATATATAAATATA-ATAATAAGTATTTTTTAAGTTAAGTTTTATAAAGATTTTTACTTTTTATAAT : 5845
cen4-CR3 : TTATATTATTTTTTTTATTTTC-----TTTTTATATATATATATATA-ATAGTAAGTATTTTTTAAGTTAAGTTTTATAAAAATCTTTACTTTTTATAAT : 2383
cen4-CR4 : TTATACTATCTTTTCTTTATTT-----CTTTTTTATATATAAATATA-ATAATAGGTATTTTTTAAGTTAAGTTTTATAAAGATTTTTACTTTTTATAAT : 5933
cen4-CR5 : TTATATTAATTTTTTTTTATTT-----TTTTCTTATATATATATATA-ATAATAAGTATTTTTTAAGTTATATTTTATAAAAATTTTTACTTTTTATAAT : 2549
cen5-CR1 : ---------------------------------------------------------------------------------------------------- :    -
cen6-CR1 : TTATATTATTTTTTTTTTATCT-----TTTTTTTATATATAAATATA-ATAATAAGTATTTTTTAAGTTAAGTTTTATAAAGATTTTTACTTTTTATAAT : 5952
cen7-CR1 : TTATACTAT-TTTTTTTTATTT-----TCTTTTTATATATAAATATA-ATAGTAAATATTTTTTAAGTTAAGTTTTATAAAGATTTTTACTTTTTATAAT : 5718
cen7-CR2 : TTATATTATTTTTTTT--ATT-----TTTTTTTATATATAAATATA-ATAATAAATATTTTTTAAATTAAGTTTTATAAAGATTTTTACTTTTTATAAT : 5744
cen7-CR3 : TTATATTA--TTTTTTTTATCT-----CTTTTTTATATATATATATA-ATAATAAGTATTTTTTAAGTTAAGTTTTATAAAAATCTTTACTTTTTATAAT : 5709
            tata tat tttt  ttat t       tttttatatata atata ata ta g attttttaa ttaagttttataaa at tttactttttataat

                    *      6120         *      6140         *      6160         *      6180         *      6200
cen1-CR1 : TAATTTATAATTTTATAAATAAAAAGTAAAAGTATAATATTTTTTT--TATAATTTTATTAAGTTTATAGTAGTTTATATAAAGTTATAATTTT-CCTT- : 5886
cen1-CR2 : TAGTTTATAATTTTATAAATAAGAGGTAAAGGTATAATATTCTTTTT-TATAATTTTATTAAGTTTATAGTAATTTATATAAAGTTATAATTTTCCTCT- : 5995
cen1-CR3 : TAGTTTATAATTTTATAAATAAAGAGTAAAAGTATAATATTCTTTTT-TATAATTTTATTAAGTTTATAGTAATTTATATAAAGTTATAATTTTCCCCC- : 6040
cen1-CR4 : TAGTTTATAATTTTATAAATAAGGGGTAAAGGTATAATATTCTTTTT-TATAATTTTATTAAGTTTATAATAGTTTATATAAAGTTATAATTTTCCCCC- : 5732
cen1-CR5 : TAGTTTATAATTTTATAAATAAAAGGTAAAGGTATAATATTCTTTTT-TATAATTTTATTAAGTTTATAATAGTTTATATAAAGTTATAATTTTCCCCC- : 5800
cen2-CR1 : TAGTTTATAATTTTATAAATAAAAAATAAAAATATAATATTTTTTTT-TATAATTTTATTAAGTTTATAATAATTTATATAAAGTTATAATTTTCCCCC- : 4779
cen2-CR2 : TAGTTTATAATTTTATAAATAAAAGGTAAAGGTATAATATTCTTTTT-TATAATTTTATTAAGTTTATAGTAATTTATATAAAGTTATAATTTTCCCCC- : 6047
cen2-CR3 : TAGTTTATAATTTTATAAATAAGGGGTAGAGGTATAATATTCTTTTT-TATAATTTTATTAAGTTTATAGTAATTTATATAAAGTTATAATTTTCCCCT- : 5882
cen2-CR4 : TAGTTTATAATTCTATAAATAAGTAATAAAAATATAATATTCTTTT--TATAATTTTATTTAATTTATAATAATTTATATAAAGTTATAACTTT-TTAC- : 3029
cen2-CR5 : TAGTTTATAATTTTATAAATAAAAGGTAAAGGTATAATATTCTTTTT-TATAATTTTATTAAGTTTATAGTAGTTTATATAAAATTATAATTTTCCCTT- : 4230
cen3-CR1 : TAGTTTATAATTTTATAAATAGAAAGTAAAAGTATAATATTTTTT---TATAATTTTATTAAGTTTATAATAATTTATATAAAGTTATAATTTTCCCCCT : 4310
cen3-CR2 : TAGTTTATAATTTTATAAATAAAGGGTAGAGGTATAATATTCTTTTT-TATAATTTTATTAAGTTTATAGTAGTTTATATAAAGTTATAATTTTCCCCC- : 5866
cen3-CR3 : ---------------------------------------------------------------------------------------------------- :    -
cen3-CR4 : TAATTTATAATTTTATAAATTAAAGGTAAAGGTATAATATTTTTTT--TATAATTTTATTAAGTTTATAATAGTTTATATAAAGTTATAATTTTCTTCC- : 5771
cen4-CR1 : TAGTTTATAATTTTATAAATAAAAGGTAAAGATATAATATTCTTTTT-TATAATTTTATTAAGTTTATAGTAATTTATATAAAGTTATAATTTTCCCCC- : 3027
cen4-CR2 : TAGTTTATAATTTTATAAATAAAAGGTAAAGATATAATATTTTTTTT-TATAATTTTATTAAATTTATAATAGTTTATATAAAGTTATAATTTTCCCTT- : 5943
cen4-CR3 : TTATTTATAATTTTATAAATAAATAGTAAAAGTATAATATTTTTTTT-TATAATTTTATTTAATTTATAATAATTTATATAAAGTTATAACTTTTTACC- : 2481
cen4-CR4 : TAGTTTATAATTTTATAAATAAAAGGTAAAGGTATAATATTTTTTTTATATAATTTTATTAAGTTTATAGTAGTTTATATAAAGTTATAATTTTCCCCC- : 6032
cen4-CR5 : TAGTTTATAATTTTATAAATAAATAGTAAAAGTATAATATCTTTTTTTTATAATTTTATTTAAGTTATAATAGTTTATATAAAGTTATAACTTT-TTAC- : 2647
cen5-CR1 : ---------------------------------------------------------------------------------------------------- :    -
cen6-CR1 : TAGTTTATAATTTTATAAATAAAAAGTAAAAGTATAATATTCTTTTT-TATAATTTTATTAAGTTTATAGTAATTTATATAAAGTTATAATTTTCCCTT- : 6050
cen7-CR1 : TAGTTTATAATTATATAAATAAAAAGTAAAGGTATAATATTCTTTTT-TATAATTTTATTAAGTTTATAATAGTTTATATAAAGTTATAATTTTCCCTT- : 5816
cen7-CR2 : TAATTTATAATTTTATAAATAAAAGGTAAAAATATAATATTTTTTTT-TATAATTTTATTAAGTTTATAGTAATTTATATAAAGTTATAATTTT--CCT- : 5840
cen7-CR3 : TAATTTATAATTTTATAAATAAGTAATAAAGGTATAATATTTTTTTTTTATAATTTTATTTAATTTAAAGTAGT-------------------------- : 5783
           ta tttataattttataaataa   gtaaa  tataatatt tttt  tataattttatt a tttata ta tttatataaagttataa ttt
```

```
                     *      6220        *      6240        *      6260        *      6280        *      6300
cen1-CR1 : TTTTTTTAATATAAATAATATAAAAGCTTTATATAAACTAGATAATTTTATAATTTACTTATTAAATAATATATAGTTAATATACTTATAAAGTATTACT : 5986
cen1-CR2 : TTTCTTTAATATAAATAATATAAAAGCTTTATATAAACTAGATAATTTTATAATCTACTTATTAAATAATATATAGTTAATATACTTATAAAGTACTGCT : 6095
cen1-CR3 : CTTTTTTAATATAAATAATATAAAAGCTTTATATAAACTAAATAATTTTATAATTTACTTATTAAATAATATATAGTTAATATACTTATAAAGTACTACT : 6140
cen1-CR4 : CTTTTTTAATATAAATAACATAGGAGCTTTATATAAACTAAATAATTTTATAATCTACCTATTAAATAATGCATAGTTAATATACTTATAAAGTACTACT : 5832
cen1-CR5 : CTTTTTTAATATAAATAATACAGAAGCTTTATATAAACTAGATAATTTTATAATTTACTTATTAAATAATATATAGTTAATATACTTATAAAGTACTACT : 5900
cen2-CR1 : TTTTTTTAATATAAATAATATAAAAGCTTTATATAAACTAGATAATTTTATAATTTACTTATTAAATAATATATAGTTAATATACTTATAAAATATTACT : 4879
cen2-CR2 : CTTTTTTAATATAAATAATACAGAAGCTTTATATAAACTAAATAATTTTATAATCCACTTATTAAATAATATATAGTTAATATACTTATAAAGTACTATT : 6147
cen2-CR3 : CTTTTTTAATATAAATAATACAGGAGCTTTATATAAACTAGATAATTTTATAATCCACCTATTAAATAATATATAGTTAATATACTTATAAAGTACTGCT : 5982
cen2-CR4 : CTTTTTTACTATAAATAATATAAAAGTATTATATAAACTAAATAACTTTATTATTTACTTATTAAATAATATATAATTAATATACTTATAAAATACTACT : 3129
cen2-CR5 : CTTTTTTAATATAAATAATATAAAAGCTTTATATAAACTAAATAATTTTATAATTTACTTATTATATAATATATAGTTAATATACTTATAAAATACTATT : 4330
cen3-CR1 : TTTTTTTAATATAAATAATATAAAAGCTTTATATAAACTAAATAATTTTATAATTTACTTATTAAATAATATATAATTAATATACTTATAAAGTACTGCT : 4410
cen3-CR2 : CTTTTTTAATATAAATAATATAAGAGCTTTATATAAACTAGATAATTTTATAATCTACTTATTAAATAATATATAGTTAATATATTTATAAAGTACTGCT : 5966
cen3-CR3 : ---------------------------------------------------------------------------------------------------- :    -
cen3-CR4 : TTTTTTTAATATAAATAATATAAAAGCTTTATATAAATTAAATAATTTTATAATTTACTTATTAAATAATATATAGTTAATATACTTATAAAGTATTATT : 5871
cen4-CR1 : TTTCTTTAATATAAATAATATAAAAGCTTTATATAAACTAAATAATTTTATAATTTACTTATTAAATAATATATAATTAATATACTTATAAAGTATTACT : 3127
cen4-CR2 : TTTTTTTAATATAAATAATATAAAAGCTTTATATAAATTAAATAATTTTATAATTTATTTATTAAATAATATATAGTTAATATACTTATAAAGTACTGCT : 6043
cen4-CR3 : TTTTCTTAATATAAATAATATAAAAGTATTATATAAATTAAATAATTTTATAATTTACTTATTAAATAATATATAATTAATATATTTATAAAATATTACT : 2581
cen4-CR4 : TTTTTTTAATATAAATAATATAAAAGCTTTATATAAATTAAATAATTTTATAATTTACTTATTAAATAATATATAGTTAATATATTTATAAAATACTATT : 6132
cen4-CR5 : CTTTTTTAATATAAATAATATAAAAATACTATATAAATTAAATAATTTTATAATTTACTTATTAAATAATATATAGTTAATATACTTATAAAATACTACT : 2747
cen5-CR1 : ---------------------------------------------------------------------------------------------------- :    -
cen6-CR1 : TTTTTTTAATATAAATAATATAAAAGCTTTATATAAACTAGATAATTTTATAATTTACTTATTAAATAATATATAATTAATATATTTATAAAGTACTGCT : 6150
cen7-CR1 : TTTTTTTAATATAAATAATATAAAAGCTTTATATAAATTAAATAATTTTATAATTTACTTATTAAATAATATATAATTAATATACTTATAAAGTATTATT : 5916
cen7-CR2 : TTTTTTTAATATAAATAATATAAAAGCTTTATATAAATTAAATAATTTTATAATTTACTTATTAAATAATATATAGTTAATATATTTATAAAGTATTACT : 5940
cen7-CR3 : ---------------------------------------------------------------------------------------------------- :    -
            ttttttaatataaataata a  ag  ttatataaa ta ataattttataat tacttattaaataatatata ttaatata ttataaa ta t  t

                     *      6320        *      6340        *      6360        *      6380        *      6400
cen1-CR1 : AATTCTTTTTTTATTAATAAATAAATTAGTTTTTATAAAGGTTTATATTTAGGTTTAAGGTTAATTATATATATTAAGCTATTTAATAATACTAATTATA : 6086
cen1-CR2 : AATTCTTTTTTTATTAATAAATAAATTAGTCTTTATAAGGGTTTATATTTAGGTTTAAGGTTAATTATATATATTAAGCTATCTAATAGTATTAATTATA : 6195
cen1-CR3 : AATTCTTTTTCTACTAGTAAATAAATTAGTCTTTATAAAGGTTTATATTTAGGTTTAAGGTTAATTATATATATTAGGCTATTTAATAGCGCTAATTATA : 6240
cen1-CR4 : AATTCTTTTTCTACTAGTAAATAAATTAGTCTTTATAAGGGTTTATATCTAGGTTTAAGGTTAATTATATATATTAAACTATTTAATAGCATTAATTATA : 5932
cen1-CR5 : AATTCTTTTTTTACTAGTAAATAAATTAATTTTTATAAAGGTTTATATTTAGGTTTAAGGTTAATTATATATATTAAACTATTTAATAGCGCTAATTATA : 6000
cen2-CR1 : AATTCTTTTTTTATTAATAAATAAATTAATTTTTATAAAAGTTTATATTTAAGTTTAAGGTTAATTATATATATTAGGCTATTTAATAATATTAATTATA : 4979
cen2-CR2 : AATTCTTTTTTTACTAATAAATAAATTAGTTTTTATAAAGGTTTATATTTAGATTTAAGGTTAATTATATATATTAAGCTATTTAATAGTATTAATTATA : 6247
cen2-CR3 : AATTCTTTTTTTACTAGTAAATAAATTAGTCTTTATAAGGGTTTATATTTAGGTTTAAGGTTAATTATATATATTAGGCTATTTAATAGTGCTAATTATA : 6082
cen2-CR4 : AGTT-TTTTTTTATAAGTAAATAAATTAATTTTTAAAAGGTTTATATTTTAATTTAAAATTAATTATATATATTAAGCTATTTAATAATATTAATTATC : 3228
cen2-CR5 : AATTCTTTTTTTATTAATAAATAAATTAGTTTTTATAAAGGTTTATATTTAGGTTTAAGGTTAATTATATATATTAAGCTATTTAATAATACTAATTATA : 4430
cen3-CR1 : AATTCTTTTTTTATTAATAAATAAATTAGTTTTTATAAAGGTTTATATTTAGGTTTAAGGTTAATTATATATATTAAGCTATTTAATAGTATTAATTATA : 4510
cen3-CR2 : AATTCTTTTTTTATTAGTAAATAAATTAGTCCTTATAAGGGTTTATATTTAGATTTAAGGTTAATTATATATATTAAGCTATTTAATAATGCTAATTATA : 6066
cen3-CR3 : ---------------------------------------------------------------------------------------------------- :    -
cen3-CR4 : AATTCTTTTTTTATTAATAAATAAATTAGTTTTTATAAAGGTTTATATTTAAATTTAAGGTTAATTATATATATTAGGCTATTTAATAATACTAATTATA : 5971
cen4-CR1 : AATTCTTTTTTTATTAGTAAATAAATTAATCTTTATAAAGGTTTATATTTAAGTTTAAAATTAATTATATATATTAAGCTATTTAATAATACTAATTATA : 3227
cen4-CR2 : AATTCTTTTTTTATTAGTAAATAAATTAGTCTTTATAAAAGTTTATATTTAGGTTTAAGGTTAATTATATATATTAAGCTATTTAATAGCGCTAATTATA : 6143
cen4-CR3 : AGT--TTTTTTTATAAGTAAATAAATTAATTTTTAAAAGGTTTATATTTTAGCTTAAAATTAATTATATATATTAAGCTATTTAATAATACTAATTATA : 2679
cen4-CR4 : AATTCTTTTTCTATTAGTAAATAAATTAGTTTTTATAAAGGTTTATATTTAGGTTTAAGGTTAATTATATATATTAGGCTATTTAATAATATTAATTATA : 6232
cen4-CR5 : AGTTTTTTTTTTATAAATAAATAAATTAATTTTTAAAAGGTTTATATTTTAACTTAAAATTAATTATATATATTAAGCTATTTAAGAATATTAATTATA : 2847
cen5-CR1 : ---------------------------------------------------------------------------------------------------- :    -
cen6-CR1 : AATTCTTTTTCTATTAGTAAATAAATTAATCTTTATAAAAGTTTATATTTAGATTTAAGGTTAATTATATATATTAAGCTATTTAATAGTATTAATTATA : 6250
cen7-CR1 : AATTTTTTTTCTATTAGTAAATAAATTAGTTTTTATAAAGGTTTATATTTAAGTTTAAGGTTAATTATATATATTAAGCTATTTAATAATACTAATTATA : 6016
cen7-CR2 : AATT-TTTTTTTATTAATAAATAAATTAGTTTTTATAAGGGTTTATATTTAGGTTTAAAGTTAATTATATATATTATGCTATTTAATAATACTAATTATA : 6039
cen7-CR3 : ---------------------------------------------------------------------------------------------------- :    -
           a tt ttttt ta  a taaataaatta t ttta aa  gtttatattt   tttaa  ttaattatatatatta gctatttaata    taattata
```

```
                           *       6420        *       6440        *       6460        *       6480        *       6500
cen1-CR1 : TTATAATATTTTTATTTATAATATTTATATAATTTTAATATTATAAAAGTATATT--TTTTTTTATTACTTTTAGTTAAATAGTAAAATACTTATATTAA : 6184
cen1-CR2 : CTATAGTATTTTTATTTATAATATTTATATAATCCTAATATTATAAAAGCATATTCCCTTTCTTATTACTTTTAGTTAAATAGTAAGATGCTTATATTAA : 6295
cen1-CR3 : CTATAGTATTTTTATTTATAATATTTATATAATTCTAATATTATAAAGGTATATTCCCTTTCTTATTACTTTTAGTTAAATAATAAGATGCTTATATTAA : 6340
cen1-CR4 : CTGCAGCATTTTTATTTATAATATCTATATAATCCTAATATTATAAAGGCATATTCCCTTTCTTATTACTTTTAGTTAAATAGTAAGATACTTATACTAA : 6032
cen1-CR5 : TTATAGTATTTTTATTTATAATATTTATATAATTTTAATATTATAAAAGTATATTCCCTTTCTTATTACTTTTAGTTAAATAGTAAGATGCTTATATTAA : 6100
cen2-CR1 : TTATAGTATTTTTATTTATAATATTTATATAATTTTAATATTATAAAAGTATATT--TTTTTTTATTACTTTTAGTTAAATAGTAAAATACTTATACTAA : 5077
cen2-CR2 : CTATAGTGTTTTTATTTATAATATTTATATAATCTTAATATTATAAAGGTATATTCCTTTTTTTATTACTTTTAATTAAATAGTAAGATACTTATACTAA : 6347
cen2-CR3 : TTATAGTATCTTTATTTATAATATTTATATAATTTTAATATTATAAAGGCATATTCCCTTTTTTATTACTTTTAGTTAAATAGTAAGATGCTTATATTAA : 6182
cen2-CR4 : TTATAGTATTTTTATTTATAATATATATATAATTTTAATATTATAAAAGTATATTTCTTTTTTTTTTACTTTTTATTAAGTAATAAAATGCTTATATTAA : 3328
cen2-CR5 : TTATAATATTTTTATTTATAATATTTATATAATCTTAATATTATAAAAGTATATTTCTTTTTTTATTACTTTTAGTTAAATAGTAAAATACTTATATTAA : 4530
cen3-CR1 : TTATAGTATTTTTATTTATAATATTTATATAATCTTAATATTATAAAAATATATTCTTTTTCTTATTACTTTTAATTAAATAGTAAGATACTTATATTAA : 4610
cen3-CR2 : TTATAGTATTTTTATTTATAATATTTATATAATCTTAATATTATAAAAGTATATTCCCTTTTTTATTACTTTTAGTTAAATAGTAAAATACTTATACTAA : 6166
cen3-CR3 : ---------------------------------------------------------------------------------------------------- :    -
cen3-CR4 : TTATAGTATTTTTATTTATAATATTTATATAATTTTAATATTATAAAAGTATATT---TTTTTTATTACTTTTAGTTAAATAATAAAATACTTATATTAA : 6068
cen4-CR1 : TTATAGTATTTTTATTTATAATATTTATATAATTTTAATATTATAAAAGTATATTTCTTTTTTTATTACTTTTAATTAAATAGTAAAATACTTATACTAA : 3327
cen4-CR2 : TTATAGTGTTTTTATTTATAATATTTATATAATCTTAATATTATAAAAGTATATTTTTTTTTTTATTACTTTTAATTAAATAGTAAAATACTTATATTAA : 6243
cen4-CR3 : TTATAGTATTTTTATTTATAATATTTATATACTTTTAATATTATAAAAGTATATTTTCTTTTTTTTTAATTTTTATTAAATAATAAAATACTTATATTAA : 2779
cen4-CR4 : CTATAGTATTTTTATTTATAATATTTATATAATTTTAATTTTATAAAGGTATATTCCTTTTTTTATTACTTTTAGTTAAATAGTAAGATACTTATATTAA : 6332
cen4-CR5 : TTATAATATTTTTATTTATAATATTTATATAATTTTAATATTATAAAAGTATATTTTTTT-----TTACTTTTAATTAAGTAATAAAATGCTTATATTAA : 2942
cen5-CR1 : ---------------------------------------------------------------------------------------------------- :    -
cen6-CR1 : CTATAGTATTTTTATTTATAATATTTATATAATCTTAATATTATAAAAGTATATTCTTTTTTTTATTACTTTTAGTTAAATAGTAAAATACTTATACTAA : 6350
cen7-CR1 : CTATAGTATTTTTATTTATAATATTTATATAATTTTAATATTATAAAGGTATATTCCTTTTTTTATTACTTTTAGTTAAATAATAAAATACTTATATTAA : 6116
cen7-CR2 : TTATAATATTTTTATTTATAATATTTATATAATCTTAATATTATAAAAGTATATTTCTTTTTTTATTACTTTTAATTAAATAGTAAGATATTTATATTAA : 6139
cen7-CR3 : ---------------------------------------------------------------------------------------------------- :    -
            tata tatttttatttataatatttatataat  taatattataaa g atatt   ttt tt ttactttta ttaaata taa at cttata taa

                           *       6520        *       6540        *       6560        *       6580        *       6600
cen1-CR1 : ATAAAGTTTTAAGGTTTATAAGTTAATAAAGTAAATCTTTAATATTATTTTAAAAAGATTATAAGTATATTATAAATCCTTTAATATAATAATTTTAATA : 6284
cen1-CR2 : ATAAAGTCCTAGGGTTTATAAGTTAATAAAGTGAATTTTTAGTGCTATTTTAAAAAGATTATAAATATATTATAAGTCTTTTAATATAATAATTTTAATA : 6395
cen1-CR3 : ATAAAGTTTTAAGGTTTGCAGGTTAATAAAGTGAATTTTTAATACTATTTTAAAAAGATTATAAGTATATTATAGGTCCTTTAGTATAATAATTTTAATA : 6440
cen1-CR4 : ATAAAGTCCTAGGGTCTGCAGGTTAATAAAGTAAATCTCTAGTACTATTTTAAAAAGATTATAGATATATTATAAGTCCTTTAGTATAATAATTTTAATA : 6132
cen1-CR5 : ATAAAGTCTTAAGGTTTATAAGTTAATAAAGTAAATCTCTAGTGCTATTTTAAAAAGATTATAAGTATATTATAAGTCCTTTAATATAATAATTTTAATA : 6200
cen2-CR1 : ATAAAGTCTTAAAGTTTATAAATTAATAAAATAAATTTTTAATACTATTTTAAAAAGGTTATATATATATTATAAGTCTTTTAATATTATAATTTTAATA : 5177
cen2-CR2 : ATAAAGTTTTAAGGTCTATAAGTTAATAAAGTAAATTTCTAGTACTATTTTAAAAAGATTATAAGTATATTATAAGTCTTTTAGTATAATAATTTTAATA : 6447
cen2-CR3 : ATAAAGTCCTAGGGTCTGCAGGTTAATAAAGCAAATTTCTAATACTATTTTAAAGAGATTGCAGGTATATTATAGGTCTTTTAGTATAATAATTTTAATA : 6282
cen2-CR4 : GTAAAGTTTTAAGGTTTATAAATTAATAAAATAAATTTTTACTACTATTTTAAAAAAATTATATATATATTATAAGTCTTTTAATATTATAATCTTAATA : 3428
cen2-CR5 : ATAAAGTTTTAAAGTTTATAAGTTAATAAAGTAAATTTCTAATACTATTTTAAAAAAATTATAAGTATATTATAAGTCTTTTAATATAATAATTTTAATA : 4630
cen3-CR1 : ATAAAGTTTTAAAATCTGCAGGTTAATAAAGTAAATTTTTAATACTATTTTAAAAAGATTATAAGTATATTATAAATCCTTTAATATAATAATTTTAATA : 4710
cen3-CR2 : ATAAAGTTCTAAGGTTTATAAGTTAATAAAGCAAATCTTTAATACTATTTTAAAAAGATTATAAATATATTATAAGTCCTTTAATATAATAATTTTAATA : 6266
cen3-CR3 : ---------------------------------------------------------------------------------------------------- :    -
cen3-CR4 : ATAAAGTCTTAAGGTTTATAAGTTAATAAAGTAAATCTTTAATACTATTTTAAAAA-ATTATAAGTATATTATAAATCTTTTAATATAATAATTTTAATA : 6167
cen4-CR1 : GTAAAGTCTTAAAGTTTATAAATTAATAAAGTAAATCTTTAATACTATTTTAAAAAGATTATAAGTATATTATAAGTCCTTTAATATAATAATTTTAATA : 3427
cen4-CR2 : GTAAAGTCTTAAAGTTTATAAGTTAATAAAGTAAATCTCTAGTACTATTTTAAAAAGATTATAAATATATTATAAGTCCTTTAATATAATAATTTTAATA : 6343
cen4-CR3 : GTAAAGTTTTAAAGTTTATAAGTTAATAAAATAAATTTTTAATACTATTTTAAAAAAATTAAATATATATTATAAGTCCTTTAATATTATAATATAAATA : 2879
cen4-CR4 : GTAAAGTCTTAAAGTTTATAAATTAATAAAGTAAAGTTTTAATATTATTTTAAAAAGATTATAAGTATATTATAAGTCTTTTAATATAATAATTTTAATA : 6432
cen4-CR5 : GTAAAGTCTTAAGGTTAATAAGTTAATAAAATAAATTTCTAATATTATTTTAAAAAGATAATATATATATTATAAGTTTTTTAATATTATAATTTTAATA : 3042
cen5-CR1 : ---------------------------------------------------------------------------------------------------- :    -
cen6-CR1 : ATAAAGTCTTAAGGTTTATAAGTTAATAAAATAAATTTCTAATACTATTTTAAAAAGATTATAAGTATATTATAAGT-TTATAGTATAATAATTTTAATA : 6449
cen7-CR1 : ATAAAGTCTTAAAGTCTGCAGGTTAATAAAGTAAATTTTTAATACTATTTTAAAAAGATTATAAGTATATTATAAATTTTTTAATATAATAATTTTAATA : 6216
cen7-CR2 : ATAAAGTTTTAAAGTCTGCAGGTTAATAAAGTAAATCTTTATTACTATTTTAAAAAGATTATAAGTATATTATAAGTCTTTTAATATAATAATTTTAATA : 6239
cen7-CR3 : ---------------------------------------------------------------------------------------------------- :    -
            taaagt  ta  gt t  a  ttaataaa taaat t ta ta tattttaaaaa attata  tatattataa t  ttta tat ataattttaata
```

```
                      *        6620          *        6640          *        6660          *        6680          *        6700
cen1-CR1 : TTTTATAGCTTAAGGTTAAATTATATAATATTTTATATTATAAAAGTAAGAATATTTTATTACTTTTTTTATAATAATTTTAAAATAATATTTTCTTATA : 6384
cen1-CR2 : TCTTATAGCTTAAGGTTAAATTATATAGTATTTTATATTATAAAGGTAAAAATATTTTATTACTTTTCTTATAGTAATTTTAAAATAATATCTTCTTATA : 6495
cen1-CR3 : TTTTATAGCTTAAGGTTAAATTATATAGTGTTTTATATTATAAAAGTAAAAATATTTTATTACTTTTCTTATAGTAATTTTAAAATAATATCTTCTTATA : 6540
cen1-CR4 : TCTTATAGCTTAAGGTTAAATTATATAGTGTTTTATATTATAAAGGTAAAAATATTTTATTACTTTTCCTATAGTAATCTTAAAATAATATCTTCTTATA : 6232
cen1-CR5 : TCTTATAGCTTAAGGTTAAATTATATAATATTTTATATTATAAAAATAGAAATATTTTATTACTTTTCTTATAATAATCTTAAAATAATATCTTTTTATA : 6300
cen2-CR1 : TTTTATAGTTTAAGGTTAAATTATATAATATTTTATATTATAAAAGTAAAAATATTTTATTACTTTTTTTATAATAATTTTAAAATAATATCTTTTTATA : 5277
cen2-CR2 : TTTTATAGCTTAAGGTTAAATTATATAGTATTTTATATTATAAAGGTAAAAATATTTTATTACTTTTCTTATAATAATTTTAAAATAATATTTTCTTATA : 6547
cen2-CR3 : TTTTATAGCTTAAGGTTAAATTATATAGTGTTTTATACTATAAAAGTAAAAATATTTTATTACTTTTCCTATAGTAATCTTAAAATAATATCTTCTTATA : 6382
cen2-CR4 : TTTTATAGCTTAAAATTAAATTATATAGTATTTTATATTATAAAAGTAAAAATATTTTATTACTTTTTTTATAATAATTTTAAAATAATATTCTTTTATA : 3528
cen2-CR5 : TTTTATAACTTAAGGTTAAATTATATAGTATTTTATATTATAAAAGTAAAAATATTTTATTATTTCTTTTATAATAATTTTAAAATAATATCTTTTTATA : 4730
cen3-CR1 : TTTTATAACTTAAGGTTAAATTATATAATATTTTATATTATAAAAGTAAAAATATTTTATTACTTTTCTTATAATAATTTTAAAATAATATTTTTTTATA : 4810
cen3-CR2 : TCTTATAGCTTAAGGTTAAATTATATAGTATTTTATATTATAAAAGTAGAAATATTTTATTACTTTTTTTATAATAATCTTAAAATAATATCTTTTTATA : 6366
cen3-CR3 : ---------------------------------------------------------------------------------------------------- : -
cen3-CR4 : TTTTATAGCTTAAGGTTAAATTATATAATATTTTATATTATAAAAGTAAAAATATTTTATTACTTTTTTTATAATAATTTTAAAATAATATCTTTTTATA : 6267
cen4-CR1 : TTTTATAGCTTAAAGTTAAATTATATAATATTTTATATTATAAAAGTAAAAATATTTTATTACTTTTTTTATAATAATTTTAAAATAATATCTTTTTATA : 3527
cen4-CR2 : TCTTATAGCTTAAGGTTAAATTATATAATATTTTATATTATAAAGGTAAAAATATTTTATTACTTTTTTTATAATAATTTTAAAATAATATTTTTTTATA : 6443
cen4-CR3 : TTTTATAACTTAAAGTTAAATTATATAATATTTTATATTATAAAAGTAAAAATATTTTATTACTTTTTTTATAATAATTTTAAAATAATATTTTCTTATA : 2979
cen4-CR4 : TTTTATAGCTTAAGGTTAAATTATATAGTATTTTATATTATAAAGGTAAAAATATTTTATTACTTTTTTTATAATAATTTTAAAATAATATTTTTTTATA : 6532
cen4-CR5 : TTTTATAGCTTAAGGTTAAATTATATAGTATTTTATATTATAAAAATAAAAATATTTTATTACTTTTTTTATAATAATTTTAAAATAATATTTTCTTATA : 3142
cen5-CR1 : ---------------------------------------------------------------------------------------------------- : -
cen6-CR1 : TCTTATAGCTTAAGGTTAAATTATATAATATTTTATATTATAAAAGTAAAAATATTTTATTACTTTTCTTATAGTAATTTTAAAATAATATTTTCTTATA : 6549
cen7-CR1 : TCTTATAGCTTAAAATTAAATTATATAATATTTTATATTATAAAAGTAAAAATATTTTATTACTTTTTTTATAATAATTTTAAAATAATATCTTTTTATA : 6316
cen7-CR2 : TCTTATAGCTTAAGGTTAAATTATATAATATTTTATATTATAAAAGTAAAAATATTTTATTACTTTTTTTATAATAATTTTAAAATAATATCTTTTTATA : 6339
cen7-CR3 : ---------------------------------------------------------------------------------------------------- : -
           t ttata cttaa gttaaattatata t ttttatattataaa gtaaaaatattttattactttt ttata taat ttaaaataatat tt ttata

                      *        6720          *        6740          *        6760          *        6780          *        6800
cen1-CR1 : TATTTATTATAATAAAATTAATAAAAAGC-TTCTTTTTTATATTAAAGTTATTATAAAGTTATAGTTTTTATTTATAATAGCTATAGATTTTTTATAATA : 6483
cen1-CR2 : TATCTGCTATAATAAAGCTAGTAAAAAGC-TTTTTTTTTATACTAAAGTTATTATAAAATTATAATTTTTATTTATAATAACTATAAATTTCTTATAGTA : 6594
cen1-CR3 : TATTTATTATAATAAAGCTAGTAAAAAGC-TTTTTTTTTATATTAAAGTTATTATAAAGTTATAATTTTTATTTATAATAGCTATAAATTTCTTATAGTA : 6639
cen1-CR4 : TATTTACTATAATAAAACTAGTAAGGAGC-TCCTTTTTTATACTAAAGTTATTATAAAGTTATAATTTTTATTTATAGTAGCTATAAATTTCTTATAGTA : 6331
cen1-CR5 : TATTTACTATAATAAAACTAATAAAAAGC-TTCTTTTTTATATTAAAGTTATTATAAAGTTATAATTTTTATTTATAATAGCTATAGATTTTTTATAATA : 6399
cen2-CR1 : TATTTATTATAATAAAACTAGTAAAAAGC-TTTTTTTTTATATTAAAGTTATTATAAAGTTATAATTTTTATTTATAATAGCTATAGATTTTTTATAATA : 5376
cen2-CR2 : TATTTATTATAATAAAGCTAGTAAAAAGC-TTTTTTTTTATATTAAAGTTATTATAAAGTTATAATTTTTATTTATAATAATTATAAATTTCTTATAATA : 6646
cen2-CR3 : TATTTACTATAATAAAGCTAGTAAGGAGC-TTTTTTTTTATATTAAAGTTATTATAAAGTTATAATTTTTATTTATAATAGCTATAGATTTTTTATAGTG : 6481
cen2-CR4 : TATTTATTATAATAAAATTAATAAAAAGC-T-TTTTTTTAAATTAAAGTTATTATAAAGTTATAGTTTTTATTTATAATAATTATAAATTTCTTATAATA : 3626
cen2-CR5 : TATTTATTATAATAAAACTAATAAAAAGC-TCTTTTTTTATATTAAAGTTATTATAAAATTATAATTTTTATTTATAATAGCTATAGATTTTTTATAATA : 4829
cen3-CR1 : TATTTATTATAATAAAATTAATAAAAAGCTTTTTTTTTTATATTAAAGTTATTATAAAATTATAGTTTTTATTTATAATAGCTATAAATTTTTTATAATA : 4910
cen3-CR2 : TATTTACTATAATAAAACTAGTAAAAAGC-TTTTTTTTTATATTAAAGTTATTATAAAGTTATAATTTTTATTTATAATAGCTATAAATTTCTTATAGTA : 6465
cen3-CR3 : --------------------------------------------------------------------------------------------------- : -
cen3-CR4 : TATTTACTATAATAAAACTAGTAAAAAGC-TTTTTTTTTATATTAAAGTTATTATAAAGTTATAGTTTTTATTTATAATAGCTATAAATTTTTTATAATA : 6366
cen4-CR1 : TATTTATTATAATAAAATTAGTAAAAAGC-TTTTTTTTTATATTAAAGTTATTATAAAGTTATAATTTTTATTTATAATAATTATAAATTTCTTATAATA : 3626
cen4-CR2 : TATTTACTATAATAAAACTAGTAAAAAGC-TCTTTTTTTATATTAAAGTTATTATAAAGTTATAATTTTTATTTATAATAATTATAAATTTCTTATAATA : 6542
cen4-CR3 : TATTTATTATAATAAAATTAATAAAAAGC-T-TTTTTTTATATTAAAGTTATTATAAAGTTATAATTTTTATTTATAATAATTATAAATTTTTTATAATA : 3077
cen4-CR4 : TATTTATTATAATAAAACTAGTAAAAAGC-TTTTTTTTTATATTAAAGTTATTATAAAGTTATAATTTTTATTTATAATAGTTATAGATTTTTTATAATA : 6631
cen4-CR5 : TATTTATTATAATAAAACTAATAAAAAGC-T-TTTTTTTATATTAAAGTTATTATAAAATTATAGTTTTTATTTATAATAATTATAAATCTTTTATAATA : 3240
cen5-CR1 : --------------------------------------------------------------------------------------------------- : -
cen6-CR1 : TATTTACTATAATAAAATTAATAAAAAGC-TTTTTTTTTATATTAAAGTTATTATAAAGTTATAATTTTTATTTATAATAGCTATAGATTTCTTATAATA : 6648
cen7-CR1 : TATTTACTATAATAAAACTAGTAAAAAGC-TTTTTTTTTATACTAAAGTTATTATAAAGTTATAATTTTTATTTATAATAGCTATAGATTTCTTATAATA : 6415
cen7-CR2 : TATTTATTATAATAAAATTAATAAAAAGC-TTTTTTTTTATATTAAAATTATTATAAAGTTATAATTTTTATTTATAATAGCTATAGATTTTTTATAATA : 6438
cen7-CR3 : --------------------------------------------------------------------------------------------------- : -
           tattta tataataaa  ta taaaaagc t  ttttttata taaagttattataaa ttata tttttatttataata  tata attt ttata ta
```

```
                   *        6820         *        6840         *        6860         *        6880         *        6900
cen1-CR1 : TAATAATAAAAGTATTTATTATTATAAAGTTAATAAGTAATAATAGTATATTTTTTATAATCCTTTTATTAATAATATTAACCTAACTTTTATTATTTAA : 6583
cen1-CR2 : TAGTAATAAAAACACTTATTATTATAAAGTTAATAAGTAATAATAATATATTCTTTATAATTCTTTTATTAATAATATTAACCTAACTTTTATTATTTAA : 6694
cen1-CR3 : TAGTAATAAAAGCACTTATTATTATAAAGTTAATAAGTAATAATAATATATTCCTTATAATCCTCTTATTAATAATATTAACCTAACTTTTATTATTTAA : 6739
cen1-CR4 : TAGTAATAAAAGCACTTATTATTATAAAGTTAATAGGTAATAATAGCATATTACCTATAATCCTTTTACTAATAATATTAACCTAACTTTTATTATCTAA : 6431
cen1-CR5 : TAGTAATAAAAGCACTTATTATTATAAAGTTAATAAGTAATAATAGCATATTACTTATAATTCTCTTACTAATAATATTAATTTAACTTCTATTATTTAA : 6499
cen2-CR1 : TAATAATAAAAGTATTTATTATTATAAAGTTAATAAGTAATAATAATATATTTTTTATAATTTTTTAATTAATAATATTAACCTAGCTTTTATTATTTAA : 5476
cen2-CR2 : TAGTAATAAAAGTACTTATTATTATAAAGTTAATAAGTAATAATAGTATATTTCTTATAATCCTTTTACTAATAATATTAACCTAACTTTTATTATTTAA : 6746
cen2-CR3 : TAGTAATAAAAGCACTTATTATTATAAAGTTAATAAGTAATAATAGCATATTCCTTATAATCCTCTTACTAATAATATTAACCTAACTTCTATTATTTAA : 6581
cen2-CR4 : TAATAATAAAAGTATTTATTATTATAAAGTTAATAAGTAATAATAATATATTTTTTATAATTCTTTTATTAATAATATTAACCTAGCTTTTATTATTTAA : 3726
cen2-CR5 : TAATAATAAAAGCACTTATTATTATAAAGTTAATAAATAATAATAATATATTTTTTATAATCCTTTTATTAATAATATTAATTTAACTTTTATTATTTAA : 4929
cen3-CR1 : TAATAATAAAAGTATTTATTATTATAAAATTAATAAGTAATAATAATATATTTTTTATAATTCTTTTATTAATAATATTAACCTAACTTTTATTATTTAA : 5010
cen3-CR2 : TAATAATAAAAGCACTTATTATTATAAAGTTAATAAGTAATAATAGTATATTTTTAGTAATTCTTTTACTAATAATATTAACCCTGCTTTTATTATTTAA : 6565
cen3-CR3 : ---------------------------------------------------------------------------------------------------- : -
cen3-CR4 : TAATAATAAAAGCATTTATTATTATAAAGTTAATAAGTAATAATAATATAATTTTTATAATTCTTTTATTAATAATATTAACTTAACTTTTATTATTTAA : 6466
cen4-CR1 : TAATAATAAAAGTATTTATTATTATAAAGTTAATAAGTAATAATAATATATTTTTAATAATTCTTTTATTAATAATATTAACCCTGCTTTTATTATTTAA : 3726
cen4-CR2 : TAATAATAAAAGCACTTATTATTATAAAGTTAATAAGTAATAATAGTATATTTTTAATAATTCTTTTATTAATAATATTAACCCTGCTTTTATTATTTAA : 6642
cen4-CR3 : TAATAATAAAAGTATTTATTATTATAAAATTAATAAATAATAATAATATATTTTTTATAATTCTTTTATTAATAATATTAACCTAGCTTTTATTATTTAA : 3177
cen4-CR4 : TAGTAATAAAAGCATTTATTATTATAAAGTTAATAAGTAATAATAATATATTTTTAATAATTTTTTTATTAATAATATTAATTTTACTTTTATTATTTAA : 6731
cen4-CR5 : TAATAATAAATATATTTATTATTATAAAGTTAATAAGTAATAATAATATATTTTTTATAATTCTTTTATTAATAATATTAACCTAGCTTTTATTATTTAA : 3340
cen5-CR1 : ---------------------------------------------------------------------------------------------------- : -
cen6-CR1 : TAATAATAAAAGCACTTATTATTATAAAATTAATAAGTAATAATAATATATTTCTTATAATCCTTTTATTAATAATATTAACCTAACTTTTATTATTTAA : 6748
cen7-CR1 : TAATAATAAAAGCACTTATTATTATAAAGTTAATAAGTA------------------------------------------------------------- : 6454
cen7-CR2 : TAATAATTAAAGTATTTATTATTATAAAATTAATAAGTAATAATAATATATTTTT-ATAATCCTTTTATTAATAATATTAACTTAACTTTTATTATTTAA : 6537
cen7-CR3 : ---------------------------------------------------------------------------------------------------- : -
             ta taataaaag a ttattattataaa ttaataagtaataata  atatt  t ataat  t tta taataatattaa     ctt tattatttaa

                   *        6920         *        6940         *        6960         *        6980         *        7000
cen1-CR1 : AAGTGCTATTATATTTTATTATTCTTTTTATATTTTAATTTAAATAAGGTATTAAAGTAATAAACTTATATTAAATTTAAAGCTAAGTAAATAAGAATTA : 6683
cen1-CR2 : AAGTGCTATTATATTTTACTATCCTCCTTACACTTTAATTTAAATAAGGCATTAAAGTAATAGACCTATATTAAGCTTAAAGCTAAGTAAATAAAAATTA : 6794
cen1-CR3 : AAGTGCTATTATATTTTACTATCCTCCTTATACTTTAATTTAAATAAGGTATTAAAGTAATAGACTTATATTAAGCCTAAAGCTAAGTAAATAGGAATTA : 6839
cen1-CR4 : AAGTGCTATTATATTTTACTATCCTTTTTACACTTTAATCTAAATAAGGTATTAAAGTAATAAACCTATATTAGGCCTAAAGCTAAGTAAATAAAAACTA : 6531
cen1-CR5 : GAGTGCTATTATATTTTACTATCCTTCTTATACTTTAATTTAAATAAGGTATTAAAGTAATAGACTTATATTAGGCCTAAAGCTAAGTAAATAAAAACTA : 6599
cen2-CR1 : AAGTATTATTATATTTTACTATCCTTCTTATACTTTAATTTAAATAAGGTATTAAAGTAATAAACTTATATTAAGCTTAAAGCTAAGTAAATAAAAATTA : 5576
cen2-CR2 : AAGTGCTATTATATTTTACTATCCTCCTTACACTTTAATTTAAATAAGGTATTAAAGTAATAAACTTATATTAAGCCTAAAGCTAAGTAAATAAGAACTA : 6846
cen2-CR3 : AAGTGCTATTATATTCTACTATCCTTCTTATACTTTAATTTAAATAAGGCATTAAAGTAATAAACCTATATTAGGCCTAAAGCTAAGTAAATAAGAATTA : 6681
cen2-CR4 : AAATACTATTATATTATACTATTTTTTTTATACTTTAATTTAAATAAAATATTAAAGTAATAAACTTATATTAAGCTTAAAGTTAAGTAAATAAAAATTA : 3826
cen2-CR5 : AAATACTATTATATTTTACTATTCTTTTTATACTTTAATTTAAATAAGATATTAAAGTAATAAACTTATATTAAGCCTAAAGCTAAGTAAATAAAAATTA : 5029
cen3-CR1 : AAGTATTATTATATTTTACTATTCTTTTTATATTTTAATTTAAATAAAGTATTAAAGTAATAGACTTATATTAAGCCTAAAGCTAAGTAAATAAAAACTA : 5110
cen3-CR2 : AAATGCTATTATATTTTACTATCCTCCTTATACTTTAATTTAAATAAGGTGTTAAAGTAATAAACTTATATTAGGCCTAAAGCTAAATAAATAAAAACTA : 6665
cen3-CR3 : ---------------------------------------------------------------------------------------------------- : -
cen3-CR4 : AAGTGCTATTATATTTTACTATTCTTTTTATATTTTAATTTAAATAAAGTATTAAAGTAATAAACTTATATTAAGCTTAAAGCTAAGTAAATAAAAATAA : 6566
cen4-CR1 : AAATGCTATTATATTTTATTATTCTTTTTATACTTTAATTTAAATAAGGCATTAAAGTAATAAACTTATATTAAGCTTAAAGCTAAGTAAATAAAAACTA : 3826
cen4-CR2 : AAATACTATTATATTTTACTATCCTTTTTATACTTTAATTTAAATAAGGTATTAAAGTAATAAACCTATATTAAGCTTAAAGCTAAGTAAATAAAAACTA : 6742
cen4-CR3 : AAGTGCTATTATATTTTATTATTTTTCTTATACTTTAATTTAATAAAAATATTAAAGTAATAAATTTATATTAAGCTTAAAGCTAAGTAAATAAAAATTA : 3277
cen4-CR4 : GAGTATTATTATATTTTACTATCCTCTTTATACTTTAATTTAAATAAGGCATTAAAGTAATAAACTTATATTAAGCTTAAAGCTAAGTAAATAAAAATTA : 6831
cen4-CR5 : AAGTGCTATTATATTTTATTATTTTTTTTATACTTTAATTTAAATAAGGTATTAAAGTAATAAACTTATATTAAGCTTAAAGCTAAATAAATAAGAATTA : 3440
cen5-CR1 : ---------------------------------------------------------------------------------------------------- : -
cen6-CR1 : AAGTGCTATTATATTTTACTATCTTTCTTATACTTTAATTTAAATAAGGTATTAAAGTAATAAACTTATATTAAGCTTAAAGCTAAGTAAATAAAAATTA : 6848
cen7-CR1 : ---------------------------------------------------------------------------------------------------- : -
cen7-CR2 : AAGTGCTATTATATTTTACTATTCTTCTTATACTTTAATTTAAATAAGGCATTAAAGTAATAAACTTATATTAAGCTTAAAGCTAAGTAAATAAAAATTA : 6637
cen7-CR3 : ---------------------------------------------------------------------------------------------------- : -
              a t  tattatatt ta tat  t  tta a tttaatttaaataa   attaaagtaata ac tatatta gc taaagctaa taaataa aa ta
```

```
                   *       7020         *       7040         *       7060         *       7080         *       7100
cen1-CR1 : ATATTTTATTATTAAATTCTTATTAACTTAATATTTAATATAATAGCTAG-TTTTTTTTATTAATTTATTAAATTATTTATAGTATTTTATAATATAACTA : 6782
cen1-CR2 : ATATTTTATTATTAAGTTCTTACTAGCTTAATATTTAGTATAATAGCTAG-TTTTTTTTACTAGTTTATTAAATTACTATAGCATTTTATAATATAGCTA : 6893
cen1-CR3 : ATATTCTATTATTAAGTTCTTACTAGCTTAGTATTTAATATAGTAGCTAG-TTTCTTTTACTAGTTTATTAAATTATTATAGTATTTTATAATATAGCTA : 6938
cen1-CR4 : ATATCCTATTATTAAGTTCCTACTAGCTTAATATTTAATGCAGTAGCTGG-TTTCTTTTACTAGTTTACTAAATTACTATAGTATTTTATAATATAGCTA : 6630
cen1-CR5 : ATATCTTATTACTAAGTTTTTACTAGCTTAATATTTAATATAGTAGCTGG-TTTTTTTTACTAGTTTATTAAATTACTATAGTATTTTATAATATAGCTA : 6698
cen2-CR1 : ATATTTTATTATTAAGTTCTTACTAGCTTAATATTTAATATAATAGCTAG-TTTTTTTTATTAATTTATTAAATTATTATAATATTTTATAATATAGCTA : 5675
cen2-CR2 : ATATTTTATTATTAAGTTCTTACTAGCTTAGTATTTAATATAGTAGCTAG-TTTTTTTTACTAATTTATTAAATTACTATAGTATTTTATAATATAGCTA : 6945
cen2-CR3 : ATATTCTATTACTAAGTTCTTACTAGCTTAGTGTTTAATATAGTAGCTGG-TTTTTTTTACTAGTTTACTAAATTACTATAGTATTTTATAATATAGCTA : 6780
cen2-CR4 : ATATTTTATTATTAAGTGTTTACTAACTTAATATAATAATATAATAACTAG-TTTTTTTTATTAATTTATTAAATTATTATAATATTTTATAATATAATTA : 3925
cen2-CR5 : ATATTTTATTACTAAGTTCTTACTAGTTTAATATTTAATATAATAGCTAG-TTTTTTTTACTAGTTTATTAAATTATTATAGTATTTTATAATATAGCTA : 5128
cen3-CR1 : ATATTTTATTATTAAATTCTTATTAGCTTAGTATTTAATATAGTAATTAG-TTTTTTTTATTAATTTATTAAATTATTATAGTATTTTATAATATAGCTA : 5209
cen3-CR2 : ATATTTTATTACTAAGTTTTTATTAGCTTAGTATTTAATATAGTAGCTAG-TTTTTTTTACTAATTTACTAAATTACTATAGTGTTTTATAGTATAGCTA : 6764
cen3-CR3 : ---------------------------------------------------------------------------------------------------- : -
cen3-CR4 : ATATTTTATTATTAAGTTTTTATTAACTTAATATTTAATATAATAGCTAGTTTTTTTTTATTAATTTATTAAATTATTATAGTATTTTATAATATAGCTA : 6666
cen4-CR1 : ATATCTTATTATTAAGTTCTTATTAGCTTAATATTTAATATAATAACTAG-TTTTTTTTATTAATTTATTAAATTATTATAGTATTTTATAATATAATTA : 3925
cen4-CR2 : ATATTTTATTATTAAATTCTTACTAGCTTAATATTTAATATAATAACTAG-TTTTTTTTATTAATTTACTAAATTATTATAATATTTTATAATATAGTTA : 6841
cen4-CR3 : ATATTTTATTATTAAGTTTTTACTAACTTAATATTTAATATAATAACTAG-TTCTTTTTACTAGTTTATTAAATTATTATAATATTTTATAATATAGCTA : 3376
cen4-CR4 : ATATTTTATTATTAAGTTCTTACTAGCTTAGTATTTAATATAATAGTTAG-TTTTTTTTACTAATTTATTAAATTACTATAGTATTTTATAATATAGTTA : 6930
cen4-CR5 : ATATTTTATTATTAAGTTTTTATTAGCTTAATATTTAATATAATAGCTAG--TTTTTTTATTAATTTATTAAATTATTATAGTATTTTATAATATAGCTA : 3538
cen5-CR1 : ---------------------------------------------------------------------------------------------------- : -
cen6-CR1 : ATATTTTATTACTATATTCCTATTAACTTAATATTTAATATAATAGCTGG-TTTTTTTTATTAATTTACTAAATTATTATAGTATTTTATAATATAGCTA : 6947
cen7-CR1 : ---------------------------------------------------------------------------------------------------- : -
cen7-CR2 : ATATCTTATTATTAAGTTTTTACTAGCTTAATATTTAATATAATAGC----TTTTTTTTATTAATTTATTAAATTATTATAATATTTTATAATATAGCTA : 6733
cen7-CR3 : ---------------------------------------------------------------------------------------------------- : -
           atat  tatta taa tt  ta ta ctta tatttaatata ta  t g ttt tttta ta ttta taaatta tata tattttataatata  ta

                   *       7120         *       7140         *       7160         *       7180         *       7200
cen1-CR1 : TAATTATTATATATTATATAGTTTTTAATATAATATATTTTTTATAAGTAATATTTTTTATATTTTTAATATAATAAAGTAATTTTTAAAACTTTTAAAA : 6882
cen1-CR2 : TAATTACTATATATTATATAGTCTTTAATATAATATATTCTTTATAAGTAATATCCCCTATACTTTTAATATAATAAGGTAATTTTTAAAACCTTTAAAG : 6993
cen1-CR3 : TAATTACTATATATTATATAGTCTTTAATATAATATATTTTTTATAAGTAATATCCCCTGCACTTTTAATATAATAAGGTAGTTTTTAAAACCTTTAAAG : 7038
cen1-CR4 : TAATTACTGTATATTATATAGTCTTTAGTATAATATATCCTTTATAAGTAATATCCTTTATACTTTTAATATAATAAGGTAGTTTTTAAAACCTTTAGAG : 6730
cen1-CR5 : TAATTACTATATATTATATAATTTTTAATATAATATATCTTTTATAAGTAATATCCCTTACACTTTTAATATAATAAGGTAGTTTTTAAAACCTTTAAAG : 6798
cen2-CR1 : TAATTATTATATATTATATAGTTTTTAATATAATATATTTTTTATAAGTAATATTCTTTATATTTTTAATAAAATAAAGTAGTTTTTAAAACTTTTAAAG : 5775
cen2-CR2 : TAATTACTATATATTATATAGTCTTTAATATAATATATCCTTTATAAGTAATATCCCCTGCACTTTTAATATAATAAGGTAGTTTTTAAAACCTTTAAAG : 7045
cen2-CR3 : TAATTACTATATATTATATAGTCTTTAATATAATATATCCTTTATAAGTAATATCCCTTACACTTTTAATATAATAAGGTAGTTTTTAAAACCTTTAGAG : 6880
cen2-CR4 : TAATTATTATATATTATATAATTTTTAATATAATATATTTTTTATAAGTAATATTCTTTATATTTTTAATATAATAAAGTAGTTTTTTAAACTTTTAAAG : 4025
cen2-CR5 : TAATTACTATATATTATATAGTTTTTAATATAATATATTTTTTATAAGTAATATTCCTTATACTTTTAATATAATAAAGTAGTTTTTAAAACTTTTAAAG : 5228
cen3-CR1 : TAATTATTATATATTATATAGTTTTTAATATAATATATTTTTTATAAGTAATATCTTTTATACTTTTAATATAATAAGGTAGTTTTTAAAACCTTTAAAG : 5309
cen3-CR2 : TAATTACTATATATTATATAGTCTTTAGTATAATATATCCTTTATAAGTAATATCCCCTACACTTCTAATAGGATAAGGTAGTCTTTAAAGCCTCTAGAG : 6864
cen3-CR3 : ---------------------------------------------------------------------------------------------------- : -
cen3-CR4 : TAATTATTATATATTATATAGTTTTTAATATAATATATTTTTTATAAGTAATATCTCTTATACTTTTAATATAATAAAGTAGTTTTTAAAACTTTTAAAG : 6766
cen4-CR1 : TAATTACTATATATTATATAGTTTTTAATATAATATATTTTTTATAAGTAATATCTTTTATACTTTTAATAAAATAAAGTAGTTTTTAAAACCTTTAAAG : 4025
cen4-CR2 : TAATTATTATATATTATATAGTTTTTAATATAATATATCTTTTATAAGTAATATCTCTTATACTTTTAATAAAATAAAGTAGTTTTTAAAACCTTTAAAG : 6941
cen4-CR3 : TAATTATTATATATTATATAATTTTTAATATAATATAT-TTTTATAAGTAATATTTTTTATACTTTTAATATAATAAAGTAGTTTTTAAAACTTTTAAAG : 3475
cen4-CR4 : TAATTATTATATATTATATAGTCTTTAATATAATATATTTTTTATAAGTAATATCCTCTACACTTTTAATAAAATAAAGTAATTTTTAAAACCTTTAAAG : 7030
cen4-CR5 : TAATTACTATATATTATATAGTTTTTAATATAATATATTTTTTATAAATAATATCTTTTATATTTTTAATATAATAAAATAGTTTTTAAAACCTTTAAAG : 3638
cen5-CR1 : ---------------------------------------------------------------------------------------------------- : -
cen6-CR1 : TAATTACTATATATTATATAGTCTTTAATATAATATATTTTTTATAAGTAATATCTCTTATACTTTTAATAAAATAAAGTAATTTTTAAAACCTTTAAAG : 7047
cen7-CR1 : ---------------------------------------------------------------------------------------------------- : -
cen7-CR2 : TAATTATTATATATTATATAGTTTTTAATATAATATATTTTTTATAAGTAATATCTTTTATACTTTTAATATAATAAAGTAGTTTTTAAAACTTTTAAAG : 6833
cen7-CR3 : ---------------------------------------------------------------------------------------------------- : -
           taatta tatatattatata t ttta tataatatat  tttataagtaatat    t  a ttttaata aataa gta tttttaaaac ttta ag
```

```
                    *      7220         *      7240         *      7260         *      7280         *      7300
cen1-CR1 : TATTTAAGTTTATATTATTTAATATAATAGTATTATTATAATATTATTTTAATATTACTAAGGTATTTTTCTACTTAATATATAGTTTTTTATAAAAGTT : 6982
cen1-CR2 : TATTTAAGTTTATATTATTTAGTATAATAATGCTATTATAATATTATTCTAATACTACTAAGGTATCCTCCTACTTAATATATAATTTTTTATAAAAATT : 7093
cen1-CR3 : TGTTTAAGTTTATATTATTTAGTATAGCAGTGCTATTATAATATTATTTTAGTACTGCTAAGGTATCCTCTTACTTAATATATAGTTTTTTATAAAAGTT : 7138
cen1-CR4 : TGTTTAAGTTTATATTATTTAGTATAGCAGCGCTATTATAATATTATTCTAATACTACTAAAGTATCCTCCTGCTTAATATATAGTTTTTTATAAAAGTT : 6830
cen1-CR5 : TATTTAAGTTTATATTATTTAGTATAATAGCGCTATTATAATATTATTTTAATACTACTAAGGTATTCTCTTACTTAATATATAGTTTTTTATAAAAGTT : 6898
cen2-CR1 : TATTTAAGTTTATATTATTTAATATAATAGTGCTATTATAATATTATTTTAATACTATTAAAATATTCTTTTACTTAATATATAGTTTTTTATAAAAGTT : 5875
cen2-CR2 : TGTTTAAATTTATATTATTTAATATAGCAGTACTATTATAATATTATTCTAGTGCTATTAAAGTATCCTCTTACTTAATATATAGTTTTTTATAAGAGTT : 7145
cen2-CR3 : TATTTAAGTTTATATTATTTAGTATAGCAGCGCTATTATAATATTATTCTAGTATTACTAAAGTATCCTCCTGCTTAATATATAGTTTTTTATAAGAGTT : 6980
cen2-CR4 : TATTTAAGTTTATATTATTTAATATAGTAATACTATTATAATATTATTTTAATATTATTAAACTGTTTTTTTACTTAATATATAGTTTTTTATAAAAGTT : 4125
cen2-CR5 : TATTTAAGTTTATATTATTTAGTATAGTAGTATTATTATAATATTATTTTAATATTATTAAAGTATCCTCTTACTTAATATATAGTTTTTTATAAAAGTT : 5328
cen3-CR1 : TATTTAAGTTTATATTATTTAGTATAGTAATACTATTATAATATTATTTTAATATTATTAAAATATCCTTTTACTTAATATATAATTTTTTATAAAAGTT : 5409
cen3-CR2 : TGTTTAAGTTTATATTATTTAATATACTAG---------------------------------------------------------------------- : 6894
cen3-CR3 : ---------------------------------------------------------------------------------------------------- :    -
cen3-CR4 : TATTTAAGTTTATATTATTTAATATAATAATACTATTATAATATTATTTTAATATTATTAAAGTATTCTCTTATTTAATATATAGTTTTTTATAAAAGTT : 6866
cen4-CR1 : TATTTAAGTTTATATTATTTAATATAATAATACTATTATAATATTATTCTAGTATTATTAAAGTATCCTTCTACTTAATATATAGTTTTTTATAAAAGTT : 4125
cen4-CR2 : TATTTAAGTTTATATTATTTAGTATAATAATACTATTATAATATTATTTTAATATTATTAAGGTATCCTCTTACTTAATATATAGTTTTTTATAAAAGTT : 7041
cen4-CR3 : TATTTAAGTTTATATTATTTAATATAATAATATTATTATAATATTATTTTAATATTATTAAAGTATTTTCTTATTTAATATATAGTTTTTTATAAAAATT : 3575
cen4-CR4 : TATTTAAGTTTATATTATTTAGTATAATAGCACTATTATAATATTATTTTAATATTATTAAGGTATCCTCCTGCTTAATATATAGTTTTTTATAAAAGTT : 7130
cen4-CR5 : TATTTAAGTTTATATTATTTAATATAATAGTATTATTATAATATTATTTTAGTATTATTAAAGTATTCTCTTACTTAATATATAGTTTTTTATAAAAGTT : 3738
cen5-CR1 : ---------------------------------------------------------------------------------------------------- :    -
cen6-CR1 : TGTTTAAGTTTATATTATTTAATATAGTAATACTATTATAATATTATTTTAATATTATTAAGGTATTCTTTTACTTAATATATAGTTTTTTATAAAAGTT : 7147
cen7-CR1 : ---------------------------------------------------------------------------------------------------- :    -
cen7-CR2 : TATTTAAGTTTATATTATTTAATATAATAATACTATTATAATATTATTTTAATATTATTAAAGTATCCTCTTATTTAATATATAGTTTTTTATAAAAGTT : 6933
cen7-CR3 : ---------------------------------------------------------------------------------------------------- :    -
           t tttaagtttatattattta tata  a    tattataatattatt ta t  t  taa  t t  t  t  ttaatatata ttttttataa a tt

                    *      7320         *      7340         *      7360         *      7380         *      7400
cen1-CR1 : ATTTCTAGTTTTTTAATAATTAAGTTATTAATTTCTTTATTATTTTTTTTATAATATTATATTTTATTCCTTTTATACTATATATTTTACTTAAATACTT : 7082
cen1-CR2 : ATTACTAATTCCTTAATAGTTAGGCTATTAATTCCCTTATTATTATTTTTATAATATTATATCTTATTCCTTTTATACTAAGTATTTTACTTAAATACTT : 7193
cen1-CR3 : ATTACTAGTTCCTTAATAGTTAGGCTATTAATTCTTTATTATTATTCTTATAATATTATATCTTATTCCTTTTATATTAAGTATTTTACTTAAATGCCT : 7238
cen1-CR4 : ATTACTAGTTCTTTAATAGTTAGGCTATTAGTTCCCTTATTATTATTTTTATAATATTATATCTTATTCCTTTTATATTAAGTATTCTGCCTAAATGCCT : 6930
cen1-CR5 : ATTACTAGTTCTTTAATAATTAGGCTATTAGTTCTTTATTATTATTTTTATAATATTATATCTTATTCCTTTTATATTAAGTATTCTGCCTAAATGCTT : 6998
cen2-CR1 : ATTATAATTTTTTTAATAATTAAGTTATTAATTCTTTATTATTATTTTTATAATATTATATTTTATTTTTTTTATATTATATATTTTATTTAAATACCT : 5975
cen2-CR2 : ATTACTAATTCCTTAATAATTAGGCTATTAGTTTTTTTATTATTATTTTTATAATATTATATTTTATTTTTTTTATATTAAGTATTCTGCCTAAATACTT : 7245
cen2-CR3 : ATTACTAGTTCTTTAATAGTTAGGCTATTAGTTCTTTATTATTATTTTTATAATATTATATCTTATTCCTTTTATATTAAGTATTCTGCCTAAATGCCT : 7080
cen2-CR4 : ATTATTAGTTTTTTAATAATTAAGTTATTAATTTCTTTATTATTATTTTTATAATATTATATTTTATTT-TTTTATATTATATATTTTACTTAAATACTT : 4224
cen2-CR5 : ATTACTAGTTTTTTAATAATTAAGTTATTAATTTCTTTATTATTATTTTTATAATATTATATTTTATTTCTTTTATATTATATATTTTACTTAAATACTT : 5428
cen3-CR1 : ATTTTTAATTTTTTAATAATTAAGTTATTAATTTTTTTATTATTATTTTTATAATATTATATTTTATTTCTTTTATATTATATATTTTACTTAAATACCT : 5509
cen3-CR2 : ---------------------------------------------------------------------------------------------------- :    -
cen3-CR3 : ---------------------------------------------------------------------------------------------------- :    -
cen3-CR4 : ATTTTTAATTTCTTAATAATTAAGTTATTAATTTTTTTATTATTATTTTTATAATATTATATTTTATTTCTTTTATATTATATATTTTACTTAAATACCT : 6966
cen4-CR1 : ATTATTATTTTTTTAATAATTAAGTTATTAATTCTTTATTATTATTTTTATAATATTATATTTTATTTCTTTTATACTATATATTTTACTTAAATACTT : 4225
cen4-CR2 : ATTACTATTTTCTTAATAATTAGGTTATTAATTTTTTTATTATTATTTTTATAATATTATATTTTATTCTTTTTATACTATATATTTTACTTAAATACTT : 7141
cen4-CR3 : ATTACTAATTCCTTAATAATTAAGTTATTAATTCCTTTATTATTATTTTTATAATATTATATTTTATTTCTTTTATATTATATATTTTACTTAAATACTT : 3675
cen4-CR4 : ATTATTAGTTTTTTAATAATTAAGTTATTAATTTTTATATTATTATTTTTATAATATTATATTTTATTTTTTTTATATTATATATTTTACTTAAATACCT : 7230
cen4-CR5 : ATTATTAGTTTTTTAATAATTAAGTTATTAATTTTTATATTATTATTTTTATAATATTATATTTTATTTTTTTTATATTATATATTTTACTTAAATACTT : 3838
cen5-CR1 : ---------------------------------------------------------------------------------------------------- :    -
cen6-CR1 : ATTACTAATTCCTTAATAATTAGGTTATTAATTCCTTTATTATTATTTTTATAATATTATATTTTATTTCTTTTATACTATATATTTTACTTAAATATTT : 7247
cen7-CR1 : ---------------------------------------------------------------------------------------------------- :    -
cen7-CR2 : ATTACTAGTTTTTTAATAATTAAATTATTAATTTTTTTATTATTATTTTTATAATATTATATTTTATTTCTTTTATATTATATATTTTACTAAAATACTT : 7033
cen7-CR3 : ---------------------------------------------------------------------------------------------------- :    -
           att   a tt  ttaata tta   tatta tt    tattatt tt ttataatattatat ttatt  ttttata ta  tatt t    aaat   t
```

```
                    *      7420         *      7440         *      7460         *      7480         *      7500
cen1-CR1 : ATATTATAAATATTAGCTTATAATTTAAAAATGCCTTAATTAAAAATACTTATTTAAATCTTTAATAGGCATTTTTTAAATAAAAAAAGGTATTTAAAAA : 7182
cen1-CR2 : ATATTATAAATATTAGCCTATAACTTAAGAATGCCTTAATTAAAGATACTTATTTAGATTTTTAATAAGCATTTCTT-AAAGAAAAAAGGTATTTAAAAA : 7292
cen1-CR3 : ATATTATAAATATTAGCCTATAACTTAAGAATGCCTTAATTAAAAATACTTATTTAGATTTTTAGTAAGCATTTTTT-AAAGAAAAAAGGTATTTAAAAA : 7337
cen1-CR4 : ATATTATAAATATTAGCCTATAACTTAAAGATGCCTTAATTAAAAATACTTATTTAGATTTTTAGTAAGCATTTCTT-AAAGAAAAAAGGTATTTAAAAA : 7029
cen1-CR5 : ATATTATAAATATTAGCCTATAACTTAAGGATGCCTTAATTAAAAATACTTATTTAGATTTTTAATAAATATTTCTT-AAAAAAAAAAGGTATTTAAAAA : 7097
cen2-CR1 : ATATTATAAATATTAGCCTATAACTTAAAAATACTTTAATTAAAAATACTTATTTAGATTTTTAATAAGTATTTTTT-AATAAAAAAAAGTATTTAAAAA : 6074
cen2-CR2 : ATATTATAAATATTAGCTTATAACTTAAGGATGCCTTAATTAAAAATACTTATCTAGATTTTTAATAAGCATTTTTT-AAAAAAAAAAGGTATTTAAAAA : 7344
cen2-CR3 : GCATTATAAATATTAGCCTATAACTTAAAAATGCCTTAATTAAAGATATTTATCTAGATTTTTAATAAGTATTTCTT-AAGGAAAAAAGGTATTTAAAAT : 7179
cen2-CR4 : ATATTATAAATATTAGCTTATAACTTAAAAATACCTTAATTAAAAATACTTATTTAAATTTTTAATAAGTATTTTTT-TTAAAAAAAAGGTATTTAAAAA : 4323
cen2-CR5 : ATATTATAAATATTAACTTATAATTTAAAAATCCTTTAATTAAAGATACTTATTTAAATCTTTAATAAGTATTTTTT-AATAAAAAAAGATATTTAAAAA : 5527
cen3-CR1 : ATATTATAAATATTAGCTTATAACTTAAAAATACCTTAATTAAAAATATTTATTTAAATTTTTATTAAGTATTTTTT-AATAAAAAAAAGTATTTAAAAA : 5608
cen3-CR2 : ---------------------------------------------------------------------------------------------------- :    -
cen3-CR3 : ---------------------------------------------------------------------------------------------------- :    -
cen3-CR4 : ATATTATAAATATTAGCTTATAACTTAAAAATACCTTAATTAAAAATACTTATTTAAATTTTTAATAAGTATTTTTT-AATAAAGAAAGGTATTTAAAAA : 7065
cen4-CR1 : ATATTATAAATATTAGCTTATAATTTAAAAATACCTTAATTAAAAATACTTATTTAGATTTTTAATAGGCATTTCTT-AATAAAGAAAGGTATTTAAAAA : 4324
cen4-CR2 : ATATTATAAATATTAGCCTATAACTTAAAAATACCTTAATTAAAAATACTTATTTAAATCTTTAGTAAGTATTTTTT-AATAAAAAAAGGTATTTAAAAA : 7240
cen4-CR3 : ATATTATAAATATTAACTTATAATTTAAAAATACTTTAATTAAAAATACTTATTTAAATTTTTAATAAGTATTTTTAAAAAATAAAAAGCTATTTAAAAA : 3775
cen4-CR4 : ATATTATAAATATTAGCTTATAACTTAAAAATGCCTTAGTTAAAAATACTTATTTAAATCTTTAATAGGCATTTTTT-AATAAAAAAAGGTATTTAAAAA : 7329
cen4-CR5 : ATATTATAAATATTAGCTTATAACTTAAAAATACCTTAATTAAAAATACTTATTTAAATCTTTAATAAGCATTTTTT-AATAAAAAAAAGTATTTAAAAA : 3937
cen5-CR1 : ---------------------------------------------------------------------------------------------------- :    -
cen6-CR1 : ATATTATAAATATTAGCCTATAACTTAAAAATACTTTAGTTAAAAATACTTATTTAAATCTTTAGTAGGCATTTTTT-AATAAAAAAAGGTATTTAAAAA : 7346
cen7-CR1 : ---------------------------------------------------------------------------------------------------- :    -
cen7-CR2 : ATATTATAAATATTAACTTATAA-TTAAAAATACCTTAATTAAAAATACTTATTTAAATTTTTAATAAGTATTTTT--AATAAAAAAAGATATTTAAAAA : 7130
cen7-CR3 : ------------------------------------------------CCTATTTAAA------------------------------------------ : 5793
               attataaatatta c tataa ttaa  at c tta ttaaa ata ttat ta at ttta ta   attt t       a aaa  tatttaaaa

                    *      7520         *      7540         *      7560         *      7580         *      7600
cen1-CR1 : AAATTTAGTATAAAAGTAGCTACTTTTATTATAATAATTTTATAATAATAAAAGAATATTATAAACTTTTTATTATTATACTATTATAAATTAATAAGTT : 7282
cen1-CR2 : AGATTTAGTATAAGAGTAGCCACTTTTACTATAATAACTTTATAGTAATAAAGAAATATTATAAACTTTTTATTATTATATTATTATAAGTTAGTAAGTT : 7392
cen1-CR3 : AGGTTTAGTATAAGGGTAGTTACTTTTACTATAATAACTTTATAATAATAAAAAAATATTATAAACTTTTTATTATTATATTATTATAAGTTAGTAAGTT : 7437
cen1-CR4 : AGGTCTAGTATAAGGGTAGCTACTTCTGCTATAATAACTTTATAGTAATAAAGAAATATTATAAACTTTTTATTATTATACTATTATAAGTTAGTAAGTT : 7129
cen1-CR5 : AGGTCTAGTATAAAAGTAGCTACTTTTACTATAATAACTTTATAATAATAAAGAAATATTATAAACTTTTTATTATTATACTATTATAAGTTAGTAAGTT : 7197
cen2-CR1 : AAATTTAATATAAAAGTAATTACTTTTATTATAATAACCTTATAATAATAAAGAAATATTATAAACTTTTTATTATTATATTATTATAAATTAGTAAGTT : 6174
cen2-CR2 : AGGTCTAGTATAAAAGTAGCTACTTTTACTATAATAACTTTATAATAATAAAGAAATATTATAAACTTTTTATTATTATACTATTATAAGTTAGTAAGTT : 7444
cen2-CR3 : AGGTCTAGTATAAGGGTAGCTACTTTTATTATAATAACTTTATAATAATAAAGAAATATTATAAACTTTTTATTATTATATTATTATAAATTAGTAAGTT : 7279
cen2-CR4 : ATATTTAATATAAAAGTAGCTACTTTTATTATAATAACCTTATAATAATAAAAAAATATTATAAACTTTTTATTATTATATTATTATAAATTAATAAGTT : 4423
cen2-CR5 : AGATTTAGTATAAAAGTAGCTACTTTTATTATAATAACCTTATAATAATAAAAGAATATTATAAACTTTTTATTATTATATTATTATAAGTTAGTAAGTT : 5627
cen3-CR1 : AGATTTAATATAAAAGTAGCTACTTTTATTATAATAACCTTATAATAATAAAGAAATATTATAAACTTTTTATTATTATATTATTATAAGTTAATAAGTT : 5708
cen3-CR2 : ---------------------------------------------------------------------------------------------------- :    -
cen3-CR3 : ---------------------------------------------------------------------------------------------------- :    -
cen3-CR4 : AGATTTAATATAAAAGTAACTACTTTTATTATAATAACCTTATAATAATAAAAGAATATTATAAACTTTTTATTATTATACTATTATAAATTAATAAATT : 7165
cen4-CR1 : AGATTTAGTATAAAAGTAGCTACTTTTATTATAATAACCTTATAATAATAAAAAAATATTATAAACTTTTTATTATTATATTATTATAAATTAGTAAGTT : 4424
cen4-CR2 : AGATTTAATATAAAAGTAGCTATTTTTATTATAATAACCTTATAGTAATAAAAAAATATTATAAACTTTTTATTATTATATTATTATAAGTTAGTAAGTT : 7340
cen4-CR3 : AGATTTAATATAAAAGTAGCTACTTTTATTATAATCTTTTTATAATAATAAAA-AATATTATAAACCTTTTATTATTATATTATTATAAATTAATAAATT : 3874
cen4-CR4 : AGATTTAGTATAAAAGTAGCTACTTTTATTATAATAACCTTATAATAATAAAAAAATATTATAAACTTTTTATTATTATATTATTATAAGTTAGTAAGTT : 7429
cen4-CR5 : AGATTTAATATAAAAGTAACTACTTTTATTATAATAACTTTATAATAATAAAAAAATATTATAAACTTTTTATTATTATATTATTATAAGTTAGTAAGTT : 4037
cen5-CR1 : ---------------------------------------------------------------------------------------------------- :    -
cen6-CR1 : AGATTTAATATAAAAGTAGCTACTTTTATTATAATAACCTTATAATAATAAAAGAATATTATAAACTTTTTATTATTATATTATTATAAGTTAATAAGTT : 7446
cen7-CR1 : ---------------------------------------------------------------------------------------------------- :    -
cen7-CR2 : AGATTTAATATAAAAATAGCTACTTTTATTATAATAATTTTATAATAATAAAGAAATATTATAAACTTTTTATTATTATATTATTATAAGTTAATAAGTT : 7230
cen7-CR3 : ---------------------------------------------------------------------------------------------------- :    -
           a  t ta tataa   ta   a tt t  tataat    ttata taataaa  aatattataaac ttttattattata tattataa tta taa tt
```

```
                       *       7620         *       7640         *       7660         *       7680         *       7700
cen1-CR1 : TAAAAGCTGCTTTTATTACTTAGATTTTCTTATTTAATAAATAACTTATATAATATTTAAAATATCTTTTTTAAGGAATTTTATTATAATTATTATATTT : 7382
cen1-CR2 : TAAAAGCTACTTTTATTACTTATATCTTTTTATTTAGCAGATAGCTTATATAATATTTAAAATATCTTTCTTAAAGAATTTTATTATAATTATTATATTT : 7492
cen1-CR3 : TAAAGGCTGCTTTTATTACTTATATTTTCTTATTTAGTAAATAGCTTATATAATATTTAAGATATCTTTTTTAAGGAATTTTATTATAATTATTATATTT : 7537
cen1-CR4 : TAAAGGCTGCTTTTATTACTTATATCTTCTTATCTAGCAGATAGCTTATATAATATTTAAAATATCTTTCTTAAGGAATTTTATTATAATTATTATATTT : 7229
cen1-CR5 : TAAAAGCTGCTTTTATTACTTATATCTTTTTATTTAGCAGATAGCTTATATAATATTTAAGATATCTTTCTTAAGAAATTTTATTATAATTATTATATTT : 7297
cen2-CR1 : TAAAAGCTGCTTTTATTACTTAAGTTTTTTTATTTAGTAAATAACTTATATAATATTTAAAATATCTTTTTTAAAGAATTTTATTATAATTATTATATTT : 6274
cen2-CR2 : TAAAGGCTGCTTTTATTACTTATATCTTCTTATTTAATAAATAGCTTATATAATATTTAAAATATCTTTCTTAAGGAATTTTATTATAATTATTATATTT : 7544
cen2-CR3 : TAAAGGCTACTTTTATTACTTATATCTTTTTATTTAGTAGATAGCTTATATAATATTTAAAATATCTTTTTTAAGGAATTTTATTATAATTATTATATCT : 7379
cen2-CR4 : TAAAAGCTATTTTTATTACTTAGGTTTTTTTATTTAATAAGTAACTTATATAATATTTAAGGTA--TTTTTTAAGGGATTTTATTATAGTTTTTATATTT : 4521
cen2-CR5 : TAAAAGCTGCTTTTATTACTTAGGTTTTTTTATTTAATAAATAACTTATATAATATTTAAAATATCTTTTTTAAAGAATTTTATTATAATTATTATATTT : 5727
cen3-CR1 : TAAGAGCTGCTTTTATTACTTAGGTTTT-TTATTTAGTAAATAACTTATATAATATTTAAAATATCTTTTTTAAAGAATTTTATTATAATTATTATACTT : 5807
cen3-CR2 : ---------------------------------------------------------------------------------------------------- :    -
cen3-CR3 : ---------------------------------------------------------------------------------------------------- :    -
cen3-CR4 : TAAAAGCTGCTTTTATTACTTAGGTTTTTTTATTAAGTAAATAACTTATATAATATTTAAAATATTTTCTTTAAGGAATTTTATTATAATTATTATATTT : 7265
cen4-CR1 : TAAAAGCTACTTTTATTATTTAAGTTTTCTTATTTAGTAAATAACTTATATAATATTTAAAATAT-TTTTTTAAGGAATTTTATTATAATTATTATATTT : 4523
cen4-CR2 : TAAAAGCTACTTTTATTACTTAAGTTTTTTTATTTAATAAATAATTTATATAGTATTTAAAATATCTTTCTTAAGGAATTTTATTATAATTATTATATTT : 7440
cen4-CR3 : TAAAAGCTATTTTTATTATTTATATTTTTTTATTTTATAAGTAACTTATATAATATTTAAAATATCTATTTTAAAGAATTTTATTATAATTTTTATATTT : 3974
cen4-CR4 : TAAAAGCTGCTTTTATTACTTAAGTTTTTTTATTTAGTAAATAACTTATATAATATTTAAGATATCTTTTTTAAAGAATTTTATTATAATTATTATATTT : 7529
cen4-CR5 : TAAAAGCTGCTTTTATTACTTAGGTTTTTTTATTTAATAAATAATTTATATAGTATTTAAGGTATTTTTCTTAAAGAATTTTATTATAATTATTATATTT : 4137
cen5-CR1 : ---------------------------------------------------------------------------------------------------- :    -
cen6-CR1 : TAAAAGCTGCTTTTATTACTTAGGTTTTCTTATTTAATAAATAATTTATATAATATTTAAAATATCTTTTTTAAAGAATTTTATTATAATTATTATATTT : 7546
cen7-CR1 : ---------------------------------------------------------------------------------------------------- :    -
cen7-CR2 : TAAAAGCTGCTTTTAATACTTAGGTTTTTTTATTTAATAAATAACTTATATAATATTTAAAATATCTTTTTTAAAAAATTTTATTATAATTATTATATTT : 7330
cen7-CR3 : ---------------------------------------------------------------------------------------------------- :    -
           taa  gct  tttta ta tta  t tt ttat     a  ta  ttatata tatttaa  ta  t   ttaa   attttattata tt ttata  t

                       *       7720         *       7740         *       7760         *       7780         *       7800
cen1-CR1 : AAGTATATAAAAATTTATTTATTTTATTACTAGCTTATTATAAAGGTATAATACTATAAGTTATATTTTATTCTTTTTTAATATTAATAGTATTATATTA : 7482
cen1-CR2 : AGGTATATAAAAATCTATCTATCTTATTACTAGCTTATTATAAAGGTATAATACTATAAGTTATATCTTATTCTCTTTTAATATTAATAGCATTATATTA : 7592
cen1-CR3 : AAGTATATAAAAATTTATTTATTTTATTACTAGCTTATTATAAAAATATAATACTATAAGTTATATCTTATTCTTTTTTAATATTAATAGTATTATACTA : 7637
cen1-CR4 : AGATATATAAAAATCTATTTATTCTATTACTAGCTTATTATAAAGGTATAATACTATAAGTTATATTTTATTCTCTTTTAATATTAATAGCATTATATTA : 7329
cen1-CR5 : AAGTATATAAAAATCTATTTATTTTATTATTAGCTTATTATAAAGGTATAATACTATAAGTTATATCTTATTCTCTTTTAATATTAATAGTATTATATTA : 7397
cen2-CR1 : AAGTATATAAAAATTTATTTATTTTATTACTAGCTTATTATAAAAATATAATATTATAAATTATATTTTATTTTTTTTTAATATTAATAATATTATATTA : 6374
cen2-CR2 : AAGTATATAAAAATCTATTTATTTTATTATTAGCTTATTATAAAGGTATAATATTATAAGTTATATTTTATTCTCTTTTAATATTAATAGTATTATATTA : 7644
cen2-CR3 : AAGTATATAAAAATCTATTTATCTTATTACTAGCTTATTATAAAGGTATAATACTATAAGTTATATTTTATTCTCTTTTAATATTAATAGTATTATACTA : 7479
cen2-CR4 : AAATATATAAAAATTTACTTATTTTATTATTAGCTTATTATAAAAATATAATATTATAAATTATATTTTATT-TTTTTTAATATTAATAGTATTATATTA : 4620
cen2-CR5 : AAGTATATAAAAATTTATTTATTTTATTACTAGCTTATTATAAAAATATAATACTATAAGTTATATTTTATTTTTTTTTAATATTAATAGTATTATATTA : 5827
cen3-CR1 : AAGTATATAAAAATTTATTTATTTTATTATTAATTTATTATAAAAGTATAATATTATAAGTTATATTTTATTTCTTTTTAATATTAATAGTATTATATTA : 5907
cen3-CR2 : ---------------------------------------------------------------------------------------------------- :    -
cen3-CR3 : ---------------------------------------------------------------------------------------------------- :    -
cen3-CR4 : AAATATATAAAAATTTATTTATTTTATTACTAGCTTATTATAAAAGTATAATACTATAAGTTATATTTTATTTTTTTTTAATATTAATAATATTATATT- : 7364
cen4-CR1 : AAGTATATAAAAATCTATTTATTTTATTATTAGCTTATTATAAAGGTATAATATTATAAGTTATATTTTATTCTTTTTTAATATTAATAGTATTATATTA : 4623
cen4-CR2 : AAATATATAAAAATTTATTTATTTTATTATTAGCTTATTATAAAAGTATAATACTATAAGTTATATTTTATTCTCTTTTAATATTAATAATATTATATTA : 7540
cen4-CR3 : AAATATATAAAAATTTATTTATTTTATTACTAGCTTATTATAAAAGTATAATATTATAAGTTATATTTTATTCTTTTTTAATATTAATAGTATTATATTA : 4074
cen4-CR4 : AAGTATATAAAAATTTATTTATTTTATTACTAGCTTATTATAAAAGTATAATACTATAAGTTATATTTTATTCTTTTTTAATATTAATAGTATTATATTA : 7629
cen4-CR5 : AAGTATATAAAAATCTATTTATTTTATTATTAGCTTATTATAAAAGTATAATATTATAAGTTATATTTT----TTTTTTAATATTAATAATATTATATTA : 4233
cen5-CR1 : ---------------------------------------------------------------------------------------------------- :    -
cen6-CR1 : AAGTATATAAAAATTTATTTATTTTATTACTAGTTTATTATAAAAATATAATATTATAAGTTATATTTTATTTTTTTTTAATATTAATAATATTATATTA : 7646
cen7-CR1 : ---------------------------------------------------------------------------------------------------- :    -
cen7-CR2 : AAGTATATAAAAATCTATTTATTTTATTATTAGCTTATTATAAAAGTATAATATTATAAGTTATATCTTATTCTTTCTTAATATTAATAGTATTATATTA : 7430
cen7-CR3 : ---------------------------------------------------------------------------------------------------- :    -
           a  tatataaaaat ta  tat  tatta ta  ttattataaa  tataata tataa ttatat tt      t ttaatattaata  attata t
```

```
                     *       7820          *       7840          *       7860          *       7880          *       7900
cen1-CR1 : AACTAATTATTTAAGGCTTAAAGGTATATTTAGTATTTAACTTAGCTTTTATTTAAAAAGATAAGTTATATTAAGGTAATTTTTATAAAAGTAATAATAT : 7582
cen1-CR2 : AACTAATTATTTAAGGCTTAAAAGTATATTTAGTATTTAACCTAGCTTTTACTTAAAAAGGTAGGTTATATTAAGGTAATTTTTATAAAGGCAGTAATAT : 7692
cen1-CR3 : AACTAATTATTTAAGGCCTAGAGGTATATTTAGTATTTAACCTAGCTTTTACTTAAAAAGGTAGGTTATATTAAGGTAATTTTTATAAAGGCAGTAGTAT : 7737
cen1-CR4 : AACTAATTATCTAGGGCCTAGAGGTATATTTAGTATTTAACCTAGCTTTTACTTAAGAAAGTAGGTTATATTAAGGTAATTTTTATAAAAGCAGTAGTGT : 7429
cen1-CR5 : AATTAATTATCTAAGGCCTAAAGGTATATTTAATATTTAACCTAGCTTTTACTTAAAAAGGTAGGTTATACTAAGGTAATTTTTATAAAGGCAGTAGTGT : 7497
cen2-CR1 : AACTAATTATTTAAGGCTTAAAAGTATATTTAGTATTTAATCTAGCTTTTATTTAAGAAGGTAAGTTATATTAAGGTAATTTTTATAAAAGTAATAGTAT : 6474
cen2-CR2 : AACTAATTATTTAAGGCCTAGAGGTATATTTAGTATTTAACCTAGCTTTTATTTAAGAAGGTAAGTTATATTAAGGTAATTTTTATAAAAGCAGTAGTAT : 7744
cen2-CR3 : AACTAATTATCTAAGGCTTAAAGGTATATTTAGTATTTAACCTAGCTTTTGCTTAAGAAAGTGGGTTATATTAAGGCAATTTTTATAAAAGCAGTAGTGT : 7579
cen2-CR4 : AATTAATTATTTAAAGCTTAAAAGTATATTTAGTATTTAACTTAGCTTTTATTTAAAAAAGTAAATTATATTAAAGTAACTTTTATAAAAGCAGTAGTAT : 4720
cen2-CR5 : AACTAATTATTTAAGGCCTAGAGGTATATTAAATATTTAATTTAGCTTTTATTTAAAAAAGTAGGTTATATTAAGGTAATTTTTATAAAAGTAGTAGTAT : 5927
cen3-CR1 : AACTAATTATTTAAGGCTTAAAAGTATATTTAGTATTTAACCTAGCTTTTATTTAAAAAAGTAGGTTATATTAAAGTAACTTTTATAAAGGCAGTAGTAT : 6007
cen3-CR2 : ---------------------------------------------------------------------------------------------------- : -
cen3-CR3 : ---------------------------------------------------------------------------------------------------- : -
cen3-CR4 : AACTAATTATTTAAGGCTTAAAAGTATATTTAGTATTTAATTTAGCTTTTATTTAAAAAGGTAAGTTATATTAAGGTAATTTTTATAAAGGTAGTAGTAT : 7464
cen4-CR1 : AATTAATTATTTAAGGCTTAAAGGTATATTTAATATTTAACCTAGCTTTTATTTAAAAAAGTAGGTTATATTAAGGTAATTTTTATAAAAGTAGTAGTAT : 4723
cen4-CR2 : AACTAATTATTTAAGGCTTAAAGGTATATTTAGTATTTAACTTAGCTTTTACTTAAAAAGATAGGTTATACTAAGGTAATTTTTATAAAGGCAGTAGTAT : 7640
cen4-CR3 : AACTAATTATTTAAGGCTTAAAAATATATTTAATATCTAACTTAGCTTTTATTTAAAAAAGTAAGTTATATTAAAGTAACTTTTATAAAAGCTATAGTAT : 4174
cen4-CR4 : AATTAATTATTTAAGGCTTAGAGGTATATTTAGTATTTAACCTAGCTTTTATTTAAGAAGGTAGGTTATATTAAGGTAATTTTTATAAAGGCAGTAGTAT : 7729
cen4-CR5 : AATTAATTATTTAAGGCTTAGAGGTATATTTAATATTTAACTTAGCTTTTATTTAAAAAAGTAAGTTATATTAAGGTAATTTTTATAAAAGTAGTAGTAT : 4333
cen5-CR1 : ---------------------------------------------------------------------------------------------------- : -
cen6-CR1 : AACTAATTATTTAAGGCCTAGAGGTATATTTAGTATTTAATCTAGCTTTTATTTAAAAAGGTAGGTTATATTAAGGTAATTTTTATAAAGGCAGTAGTAT : 7746
cen7-CR1 : ---------------------------------------------------------------------------------------------------- : -
cen7-CR2 : AACTAATTATTTAAGGCTTAAAAGTATATTTAATATTTAACCTAGCTTTTATTTAAAAAAGTAAGTTATATTAAGGTAATTTTTATAAAAGTAGTAGTAT : 7530
cen7-CR3 : ---------------------------------------------------------------------------------------------------- : -
             aa taattat ta  gc ta a  tatatt a tat taa  tagctttt  ttaa aa  t   ttata taa g aa ttttataaa g    ta t t

                     *       7920          *       7940          *       7960          *       7980          *       8000
cen1-CR1 : TTACTTACTTTTTTATAAATTA-TTTTTTATTAT-TTTTTTTAATATAAATATATATAATTTTAAGTCTTTTTTTTATATTAATTTCTTTATAAATTTAA : 7680
cen1-CR2 : TTACTTACTTTTTTATAAGTTA-TTTTTTATTATTTTCTTTTAGTATAAATATATTTAATTTTAAATCTTCTTTTTATATTAATTTCTTTATAAATTTAA : 7791
cen1-CR3 : TTACTTACTTTTTTATAAATTA-TTCTTTATTATCTTTTTTTAGTATAAATATATTTAATTCTAGATCTTCTCCTTATATTAATTTCTTTATAAATTTAA : 7836
cen1-CR4 : CTGCTTACTTTTTTATAGGTTA-TTCTTTATTATCTTCTTTTAATATAAATATATTTAATTTTAAGTCTTCTTTTTATATTAATTTCTTTATAAATTTAA : 7528
cen1-CR5 : TTACTTACTTTTTTATAAGTTA-TTTTTTATTATCTTTTTTTAATATAAATATATTTAATTCTAAGTCTTCTCCTTATATTAATTTCTTTATAAATTTAA : 7596
cen2-CR1 : TTACTTACTTTTTTATAAATTA-TTTTTTATTATCTTTTTTTAATATAAATATATTTAATTTTAAGTCTTTTTTTTATATTAATTTCTTTATAAATTTAA : 6573
cen2-CR2 : TTACTTACTTTTTTATAAGTTA-TTTTTTATTATCTTTTCTTAATATAAATATATTTAATTTTAAATCTTCTCTTTATATTAATTTTTTTATAAATTTAA : 7843
cen2-CR3 : TTACTTACTTTTTTATAGGTTA-TTCTTTATTATCTTCTTTTAGTATAAATATATCTAATTTTAAATCTTCTTCTTATATTAATTTCTTTATAAATTTAA : 7678
cen2-CR4 : TTATTTATTTTTTTATAAGTTATTTTTTTATTATTTTCTTTTAATATAAATATATTTAATTTTAAGT-CTTTTTTTATATTAATTTCTTTATAAATTTAA : 4819
cen2-CR5 : TTACTTACTTTTTTATAAGTTA-TTTTTTATTATCTTTTTTTAATATAAATATATTTAATTTTAGATTTTCTTTTTATATTAATTCTTTTATAAATTTAA : 6026
cen3-CR1 : TTACTTACTTTTTTATAAGTTA-TTTTTTATTATTTTCTTTTAATATAAATATATTTAATTTTAAGTCTTTTTTTTATATTAATTTCTTTATAAATTTAA : 6106
cen3-CR2 : ---------------------------------------------------------------------------------------------------- : -
cen3-CR3 : ---------------------------------------------------------------------------------------------------- : -
cen3-CR4 : TTACTTACTTTTTTATAAATTA-TTTTTTATTATTTTCTTTTAATATAAATATATTTAATTTTAAGTTTTTTTTTTATATTAATTTTTTTATAAATTTAA : 7563
cen4-CR1 : TTACTTACTTTTTTATAAGTTA-TTTTTTATTATCTTCTTTTAGTATAAATATATTTAATTTTAAGTCTTTTTTTTATATTAATTTTTTTATAAATTTAA : 4822
cen4-CR2 : TTACTTACTTTTTTATAAGTTA-TTCTTTATTATTTTCTTTTAATATAAATATATTTAATTTTAAATCTTTTTTTTATATTAATTTTTTTATAAATTTAA : 7739
cen4-CR3 : TTATTTATTTTTTTATAAGTTA-TTTTTTATTATCTTTTTTTAATATAAATATATTTAATTTTAAG--TTTTTTTTATATTAATTTCTTTATAAATTTAA : 4271
cen4-CR4 : TTACTTACTTTTTTATAAGTTA-TTTTTTATTATTTTTTTTTAATATAAATATATTTAATTTTAAGTTTTCTTTTTATATTAATTTTTTTATAAATTTAA : 7828
cen4-CR5 : TTATTTACTTTTTTATAAATTA-TTTTTTATTAT-TTTTTTTAATATAAATATATTTAATTTTAG---TTTTTTTTATAATAATTTCTTTATAAATTTAA : 4428
cen5-CR1 : ---------------------------------------------------------------------------------------------------- : -
cen6-CR1 : TTACTTACTTTTTTATAAGTTA-TTTTTTATTATCTTTTTTTAATATAAATATATTTAATTTTAAATCTTCTCTTTATATTAATTTTTTTATAAATTTAA : 7845
cen7-CR1 : ---------------------------------------------------------------------------------------------------- : -
cen7-CR2 : TTATTTACTTTTTTATAAGTTA-TTTTTTATTATTTTCCTTTAATATAAATATATTTAATTTTAGGTTTTTTTTTATATTAATTTCTTTATAAATTTAA : 7629
cen7-CR3 : ---------------------------------------------------------------------------------------------------- : -
              t  tta ttttttata  tta tt tttattat tt   tta tataaatatat taatt ta     t t  ttata taatt  tttataaatttaa
```

```
                    *      8020         *      8040         *      8060         *      8080         *      8100
cen1-CR1 : TAAGTTATTACTTTT-TTTATAC--TTTTTTTTATTTTTTTACTTAGCTTTATAACTTTTTTATAAATATTCTTAAAAGTATAAGGTTTAATAAAACTAT : 7777
cen1-CR2 : TAAGTTATTACTTTT-TTTATAC--TTTTTTTAATTCTTTTACCTAGCTTTATAACTTTTTTATAAATATCTTTAAAAATGCAGGGTTTAATAAAACTAT : 7888
cen1-CR3 : TAAGTTATTACTTTT-TTTATAC--TTTTTTTAATTCTTTTACCTAGCTTTATAACTTTTTTATAAATATCTTTAAAAGTGCAGGGTTTAATAAAACTAT : 7933
cen1-CR4 : TAAGTTATTACTTTT-TTTATAC--TTTTTTTAATTCTTTTACCTAGCTTTATAACTTTTTTATAAATATCTTTAAGAGTATAAGGTTTAATAAAACTAT : 7625
cen1-CR5 : TAAGTTATTACTTTT-TTTATAC--TTTTTTTAATTCTTTTACCTAGCTTTATAACTCTTTTATAAATATTTTTAAAAGTGCAGGGTTTAATAAAACTAT : 7693
cen2-CR1 : TAAGTTATTACTTTT-TTTATAC--TTTTTTTAATTTTTTTACTTAGCTTTATAATTCTTTTATAAATATTTTTAAAAATATAAGATTTAATAAAATTAT : 6670
cen2-CR2 : TAAGTTATTACTTTT-TTTATAC--TTTTTTTAATTCTTTTACTTAGCTTTATAACTCTTTTATAAATATTTTTAAAAATGCAGGGTTTAATAAAATTAT : 7940
cen2-CR3 : TAAGTTATTACTTTT-TTTATAC--TTCTCTTAATTCTTTTACCTAGCTTTATAACTCTTTTATAAATATCCTTAAAAGTATAAGGTTTAATAAAACTAT : 7775
cen2-CR4 : TAAGTTATTACTTTT-TTTATAC--TTTTTTTAATTTTTTTATTTAGCTTTATAACTTTTTTATAAATATTTTTAAAAATATAAAATTTAATAAAATTAT : 4916
cen2-CR5 : TAAGTTATTACTTTT-TTTATAC--TTTTTTTAATTTTTTTACTTAGCTTTATAACTTTTTTATAAATATTTTTAAAAGTATAAGGTTTAATAAAACTAT : 6123
cen3-CR1 : TAAATTATTACTTTT-TTTATAC--TTTTTTTAATTTTTTTACTTAGCTTTATAACTTTTTTATAAATATTTTTAAAAATATAAGATTTAATAAAACTAT : 6203
cen3-CR2 : ---------------------------------------------------------------------------------------------------- :    -
cen3-CR3 : ---------------------------------------------------------------------------------------------------- :    -
cen3-CR4 : TAAGTTATTACTTTT-TTTATAC--TTTTTTTAATTTTTTTACTTAGCTTTATAACTTTTTTATAAATATTCTTAAAAGTATAAAGTTTAATAAAACTAT : 7660
cen4-CR1 : TAAATTATTACTTTT-TTTATAC--TTTTTTTAATTCTTTTACTTAGCTTTATAACTTTTTTATAAATATTTTTAAAAATATAAGGTTTAATAAAACTAT : 4919
cen4-CR2 : TAAGTTATTACTTTT-TTTATAC--TTTTTTTAATTCTTTTACCTAGCTTTATAACTCTTTTATAAATATCTTTAAAAGTATAAGGTTTAATAAAACTAT : 7836
cen4-CR3 : TAAGTTATTACTTTT-TTTATACTTTTTTTTTAATTTTTTTACTTAATTTTATAACCTTTTTATAAATATTTTTAAAAATATAAAATTTAATAAAGCTAT : 4370
cen4-CR4 : TAAGTTATTACTTTTTTTTATAC--TTTTTTTAATTCTTTTACTTAACTTTATAACTTTTTTATAAATATCCTTAAAAATGCAAAGTTTAATAAAACTAT : 7926
cen4-CR5 : TAAGCTATTACTTTT-TTTATAC-TTTTTTTTAATTCTTTTACTTAGCTTTATAACTTTTTTATAAATATTTTTAAAAGTATAAGGTTTAATAAAACTAT : 4526
cen5-CR1 : ---------------------------------------------------------------------------------------------------- :    -
cen6-CR1 : TAAGTTATTACTTTT-TTTATAC-TTTTTTTTAATTTTTTTACTTAGCTTTATAACTTTTTTATAAATATCCTTAAAAGTATAAGGTTTAATAAAACTAT : 7943
cen7-CR1 : ---------------------------------------------------------------------------------------------------- :    -
cen7-CR2 : TAAGTTATTATTTTT-TTTATAC--TTTTTTTAATTTTTTTATTTAGCTTTATAACTTTTTTATAAATATTTTTAAAAGTATAAGATTTAATAAAATTAT : 7726
cen7-CR3 : ---------------------------------------------------------------------------------------------------- :    -
           taa  tatta tttt tttatac  tt t tt att tttta  ta  tttataa   ttttataaatat  ttaa a t  a   tttaataaa  tat

                    *      8120         *      8140         *      8160         *      8180         *      8200
cen1-CR1 : ATATATTTTTTAAGTTATAGTATTTATAAGTAAACTTTATATTATTTTTAATAAAGCTTAATTTTATATTACTGCTTTTATTATTAAATTTTACTTACTT : 7877
cen1-CR2 : ATATATTCCTTAAGTTATAGTATTTATAAGTAAACTTTATGCTATTTTTAATAAAGCTTAATTTTATATTACTGCCCTTATTATTAAGTTTTACTTACTT : 7988
cen1-CR3 : ATATATTCCTTAAGTTATAGTATTTATAAGTAAACTTTATGCTGTTTTTAATAAAGCTTAATTTTATATTACTGCCTTTATTATTAAGTTTTACCTACTT : 8033
cen1-CR4 : ATACATTCCCTAAGTTATAGTATTTATAAGTAAACTCTATGCTATTTCTAGTAAAGCTTAATTTTATATTACTGCCCTTATTATTAAGTTTTACCTACTT : 7725
cen1-CR5 : ATATATTCCTTAAGTTATAGTATTTATAAGTAAACTCTATACTGTTTTTAATAAAGCTTAATTTTATATTACTGCCCTTATTATTAAGTTTTACTTACTT : 7793
cen2-CR1 : ATATATTTTTTAAATTATAATATTTATAAATAAACTTTATACTATTTTTAATAAAGCTTAATTTTATATTATTATTTTTATTATTAAGTTTTACTTACTT : 6770
cen2-CR2 : ATATATTCCTTAAGTTATAGTATTTATAAATAAACTTTATACTATTTTTAATAAAGCTTAATTCTGCATTACTGCCTTTATTATTAAGTTTTACTTACTT : 8040
cen2-CR3 : ATATATTCCTTAAGTTATAGTATTTATAAGTAAACTTTATATTATTTTTAATAAAGCTTAATTCTGCATTACTGCCTTTATTATTAAGTTTTACTTACTT : 7875
cen2-CR4 : ATATATTTTTTAAGTTATAGTATTTATAAATAAAGTTTATGCTATTTTTAATAAAGCTTAGTTTTATATTATTATTTTTATTATTAAGTTTTATTTACTT : 5016
cen2-CR5 : ATATATTTTTTAAGTTATAGTATTTATAAGTAAACTTTATACTATTTTTAATAAAGCTTAATTTTATATTACTGCTTTTATTATTAAGTTTTACTTACTT : 6223
cen3-CR1 : ATATATTCTTTAAGTTATAGTATTTATAAGTAAATTTTATATTATTTTTAATAAAGCTTAATTTTATATTACTGCCTTTATTATTAAGTTTTACTTATTT : 6303
cen3-CR2 : ---------------------------------------------------------------------------------------------------- :    -
cen3-CR3 : ---------------------------------------------------------------------------------------------------- :    -
cen3-CR4 : ATATATTTTTTAAGTTATAGTATTTATAAATAAACTTTATATTATTTTTAATAAAGCTTAATTTTATATTACTACTTTTATTATTAAGTTTTATTTACTT : 7760
cen4-CR1 : ATATATTTTTTAAGTTATAGTATTTATAAGTAAACTTTATACTATTTTTAATAAAGCTTAATTTTATATTATTACTTTTATTATTAAGTTTTACTTACTT : 5019
cen4-CR2 : ATATATTTTTTAAATTATAGTATTTATAAATAAACTTTATACTATTTTTAATAAAGCTTAATTTTATATTACTGCTTTTATTATTAAGTTTTACTTACTT : 7936
cen4-CR3 : ATATATTTTTTAAGTTATAATATTTATAAATAAACTTTATATTATTTTTAATAAAGCTTAATTTTATATTATTATTTTTATTATTAAGTTTTACTTACTT : 4470
cen4-CR4 : ATATATTCTTTAAGTTATAATATTTATAAGTAAACTTTATACTATTTTTAATAAAGCTTAATTTTATATTACTGCCTTTATTATTAAGTTTTATTTACTT : 8026
cen4-CR5 : ATATATTTTTTAAGTTATAGTATTTATAAGTAAACTTTATATTATTTTTAATAAAGCTTAATTTTATATTACTGCTTTTATTATTAAGTTTTACTTACTT : 4626
cen5-CR1 : ---------------------------------------------------------------------------------------------------- :    -
cen6-CR1 : ATATATTTCTTAAGTTATAGTATTTATAAGTAAACTTTATACTATTTTTAATAAAGCTTAATTTTATATTACTGCTTTTATTATTAAGTTTTACTTACTT : 8043
cen7-CR1 : ---------------------------------------------------------------------------------------------------- :    -
cen7-CR2 : ATATATTTTTTAAGTTATAATATTTATAAATAAACTTTATATTATTTTTAATAAAGCTTAATTTTATATTATTACTTTTATTATTAAGTTTTACTTATTT : 7826
cen7-CR3 : ---------------------------------------------------------------------------------------------------- :    -
           ata att   taa ttata tatttataa taaa t tat  t ttt ta taaagctta tt t  atta t    ttattattaa tttta  ta tt
```

```
                    *      8220         *      8240         *      8260         *      8280         *      8300
cen1-CR1 : TATTAAAAGCTTATCCTTTTTTTATTTATTATTAAAGGATTATTATTATAAGATTATAAAGTTTATAACTTAATATAATTATTATTTTATATATTTACTA : 7977
cen1-CR2 : TATTAAAAGCTTATCCTTTCCTTATTTACTATTAAAAGGTTATTACTATAAGCTTATAAAGTTTATAACTTATTATAACTATTATTTTATATATTTACTA : 8088
cen1-CR3 : TATTAAAAGCTTATCCCTTCCTTATTTATTATTAAAGGGTTATTATTATAAGCTTATAAAGTTTATAACTTATTATAACTATTATTTTATATATTTACTA : 8133
cen1-CR4 : TATTAAAAGCTTATCCCTTTTTTATTTACTATTAAAGGGTTATTACTATAAGATTATAAAGTCTATAACTTATTATAACTATTATTTTATATATTTACTA : 7825
cen1-CR5 : TATTAAAAGCTTATCCCTTTCTTATTTATTATTAAAGGGTTATTATTATAAGATTATAAAGTCTATAACTTATTATAACTATTATTTTATATATTTACTA : 7893
cen2-CR1 : TATTAAAAGCTTATCCCTTTTTTATTTATTATTAAGAGGTTATTATTATAAAATTATAAAATTTATAACTTATTATAATTATTATTTTATATATTTACTA : 6870
cen2-CR2 : TATTAAAAGCTTATCCCTTTTTTATTTATTATTAAAGGGTTATTATTATAAGATTATAAAGTCTATAATTTATTATAACTATTATTTTATATATTTATTA : 8140
cen2-CR3 : TATTAAAAGCTTATCCCTTCCTTATTTATTATTAAAGGGTTATTACTATAAGATTATAAAGTCTATAACTTATTATAATTATTATTTTATATATTTACTA : 7975
cen2-CR4 : TATTAAAAGCTTAT--TTTTTTTATTTATTATTAAAAGGTTATTACTATAAAATTATAAAGTTTATAATTTATTATAGTTATTATTTTATATAGTTATTA : 5114
cen2-CR5 : TATTAAAAGCTTATCCCTTTTTTATTTATTATTAAAAGGTTATTATTATAAAATTATAAAGTTTATAACTTATTATAATTATTATTTTATATATTTATTA : 6323
cen3-CR1 : TATTAAAAGCTTAT-TTTTTTTTATTTATTATTAAAAGGTTATTATTATAAAATTATAAAGTTTATAATTTATTATAATTATTATTTTATATATTTATTA : 6402
cen3-CR2 : ---------------------------------------------------------------------------------------------------- :    -
cen3-CR3 : ---------------------------------------------------------------------------------------------------- :    -
cen3-CR4 : TATTAAAAGCTTATCTTTTTTTTATTTATTATTAAAGGGTTATTATTATAAAATTATAAAGTTTATAACTTATTATAATTATTATTTTATATATTTACTA : 7860
cen4-CR1 : TATTAAAAGCTTATCCCTTTCTTATTTACTATTAAAAGGTTATTATTATAAAATTATAAAGTTTATAACTTATTATAATTATTATTTTATATATTTACTA : 5119
cen4-CR2 : TATTAAAAGCTTATCTTTTTTTTATTTATTATTAAAGGGTTATTATTATAAAATTATAAAGTTTATAATTTATTATAATTATTATTTTATATATTTACTA : 8036
cen4-CR3 : TATTAAAAGCTTAT--TTTTTTTATTTATTATTAAAAGGTTATTATTATAAAATTATAAGGTTTATAACTTATTATAATTATTATTTTATATATTTATTA : 4568
cen4-CR4 : TATTAAAAGCTTATCCTTTTCTTATTTATTATTAAAGGGTTATTATTATAAGATTATAAAGTTTATAACTTATTATAATTATTATTTTATATATTTACTA : 8126
cen4-CR5 : TATTAAAAGCTTAT-TTTTTTTTATTTATTATTAAAGGGTTATTATTATAAAAGTATAAAATTTATAATTTATTATAATTATTATTTTATATATTTACTA : 4725
cen5-CR1 : ---------------------------------------------------------------------------------------------------- :    -
cen6-CR1 : TATTAAAAGCTTATCCCTTTTTTATTTATTATTAAAGGGTTATTACTATAAGATTATAAAGTTTATAACTTATTATAATTATTATTTTATATATTTACTA : 8143
cen7-CR1 : ---------------------------------------------------------------------------------------------------- :    -
cen7-CR2 : TATTAAAAGCTTAT-TTTTTTTTATTTATTATTAAAAAGTTATTATTATAAAATTATAAAATTTATAATTTATTATAATTATTATTTTATATATTTACTA : 7925
cen7-CR3 : ---------------------------------------------------------------------------------------------------- :    -
           tattaaaagcttat   tt  ttattta tattaa    ttatta tataa   tataa  t tataa tta tata  tattattttatata tta ta

                    *      8320         *      8340         *      8360         *      8380         *      8400
cen1-CR1 : T-TTTTTTTTTAATAATTTCTTATCCTTTAATAAAAATAAACTTATTAAAGCTAGTATATAATATAAAAAAAT--AAAAAAAAAGAGAATAATTAAGGTT : 8074
cen1-CR2 : TCCTTCTTTTTAATAATTCCTTATCCTTTAATAAAAATAAACCTATTAAAGCTAGTATATAATATAAAAAAATAAAAAAAGAGAGAGAATAATTAAGGTT : 8188
cen1-CR3 : TTCTTCTTTTTAATAATTTCTTATCCTTTAGTAAAAATAAACCTATTAAAGCTAGTATATAATATAAGAAAGTAAAAAAAGAGAGAGAATAATTAAGGTT : 8233
cen1-CR4 : TCCTTCTTTTTAATAGTTCCTTATCCCTTAGTAAAAATAAACCTATTAAAGCTAGTATATAATATAAAGAAGTAAAAAGAGAGAGAGGATAATTAAAGTT : 7925
cen1-CR5 : TCCTTTTTTTTAATAATTCCTTATCCTTTAGTAAAAATAAACCTATTAAAGCTAGTATATAATATAAAAAAGTAAAAAAAAAGAGAGAATAACTAAAGTT : 7993
cen2-CR1 : TCCTTTTTTTTAATAATTCCTTATCTTTTAATAAAGATAAACTTATTAAAGCTAGTATATAATATAAAAAAGT-AAAAAAAAAAGAGAATAATTAAGGTT : 6969
cen2-CR2 : TCCTTCTTTTTAATAGTTTTTTATCTTTTAATAAAAATAAACTTATTAAAGCTAGTATATAATATAAAAAAGTAAAAAAAAAAAAAGAATAACTAAGGTT : 8240
cen2-CR3 : TCCTTCTTTTTAATAGTTCTTTATCCCTTAGTAAAGATAAACCTATTAAAGCTAGTATATAATATAAAAAAGTAAAAAGAGAGAGAGAATAACTAAGGTT : 8075
cen2-CR4 : TT-ATTTTTTTAATAATTCCTTATTTTTTAATAAAAATAAACTTATTAAAGCTAATATATAATATAAAAAAGT--AAAAAGAAAAAGAATAATTAAGGCT : 5211
cen2-CR5 : TTCTTCTTTTTAATAATTCTTTATCTTTTAAAAAAAATAAACTTATTAAAGCTAGTATATAATATAAAAAAGT-AAAAAAAAAAGAGAATAACTAAGGTT : 6422
cen3-CR1 : TCTTTTTTTTTAATAGTTTTTTATCTTTTAATAAAAATAAACTTATTAAAGCTAGTATATAATATAAAAAAGTAAAAAAAGAAAAAAAATAATTAAAGTT : 6502
cen3-CR2 : ---------------------------------------------------------------------------------------------------- :    -
cen3-CR3 : ---------------------------------------------------------------------------------------------------- :    -
cen3-CR4 : T-TTTTTTTTTAATAATTCCTTATCTTTTAATAAAAATAAACTTATTAAAGCTAGTATATAATATAAAAAAGT---AAAAGAAAAAGAATAATTAAAATT : 7956
cen4-CR1 : TCCTTCTTTTTAATAATTCCTTATCCTTTAATAAAAATAAACTTATTAAAGCTAGTATATAATATAAAAAAGTAAAAAAAAAAAAAGAATAACTAAAGTT : 5219
cen4-CR2 : TCTTTTTTTTTAATAATTCTTTATCCTTTAATAAAAATAAACTTATTAAAGTTAATATATAATATAAAGAAAT------AAAAAAAAAATAATTAAGGTT : 8130
cen4-CR3 : TT-TTTTTTTAAATAATTTTTTATTTTTTAATAAAAATAAATTTATTAAAGTTAGTATATAATATAAAAAAGT---AAAAAAAAAAGAATAATTAAGGCT : 4664
cen4-CR4 : TTCTTTTTTTTAATAGTTTCTTATCCTTTAATAAAAATAAATTTATTAAAGCTAGTATATAATATAAAAAAGTAAAAAAAAAAAAAGAATAATTAAAGTT : 8226
cen4-CR5 : TT-TTTTTTTTAATAATTTTTTATCTTTTAGTAAAAATAAACTTATTAAAGTTAATATATAATATAAAAAAGT-AAAAAAAAAAGAGAATAATTAAGGTT : 4823
cen5-CR1 : ---------------------------------------------------------------------------------------------------- :    -
cen6-CR1 : TCCTTCTTTTTAATAATTTCTTATCCTTTAATAAAAATAAACTTATTAAAGCTAGTATATAATATAAAAAAGTAAAAAAAAAAAGAGAATAATTAAAGTT : 8243
cen7-CR1 : ---------------------------------------------------------------------------------------------------- :    -
cen7-CR2 : T-TTTTTTTTTAATAATTTCTTATTTTTTAATAAAAATAAACTTATTAAAGCTAGTATATAATATAAAAAAGT-AAAAAAAAAAAAAAATACTTAAGGTT : 8023
cen7-CR3 : ---------------------------------------------------------------------------------------------------- :    -
           t   t tttt aata tt  ttat   tta  aaa ataaa  tattaaag ta tatataatataa  aa t      a a a a  ata  taa   t
```

```
                   *      8420         *      8440         *      8460         *      8480         *      8500
cen1-CR1 : ATAAAAAGTTAATATATAGTTATTAATTATTATTAAGTAGTTTAAAAGTTAATTATATAATTACTTTT-AAAAGTAAGTATTTATAAGTAATTATATTTA : 8173
cen1-CR2 : ATAAAAAGTTAGTATATAGTTATTAATTATTACTAAGTAGTTTAAGAGTTAATTATATAATTACTTTT-AAAAGTAGATGTTTATAAGTAACTATATTTA : 8287
cen1-CR3 : ATAAGAAGTTAATATATAGTTATTAGTTATTACTAAGTGGTTTAAGAGTTAATTATATAATTACTTTT-AAAAATAGGTGTTTATAAGTAATTATATTTA : 8332
cen1-CR4 : ATAAGAAGTTAGTATATAGTTATTAATTACTACTAAGTAGTCTAGGAGTTAATTATATAATTACTTTT-AAAAGTAAGTGTTTATAAGTAACTATATTTA : 8024
cen1-CR5 : ATAAAAAGTTAATATATAATTATTAATTACTACTAAGTAGTCTAAGAGTTAATTATATAATTACTTTT-AAAAGTAGGTGTTTATAAGTAATTATATTTA : 8092
cen2-CR1 : ATAAAAAGTTAATATATAATTATTAATTACTATTAAGTAGTTTAAAAGTTAATTATATAATTACTTTT-AAAAATAAGTATTTATAAGTAATTATATTTA : 7068
cen2-CR2 : ATAAAAAGTTAGTATATAATTATTAATTACTACTAAGTGGTCTAAAAGTTAATTATATAATTACTTTT-AAAGGTAGGTATTTATAAGTAATTATATTTA : 8339
cen2-CR3 : ATAAAAAGTTAATATATAATTATTAATTACTACTAAGTGGTTTAAAAGTTAATTATATAATTACTTTT-AAAGGTAGGTGTTTATAAGTAATTATATTTA : 8174
cen2-CR4 : AAAAAAAGTTAGTATATAGTTATTAATTACTATTAAGTAGTTTAAGGGTTAATTATATAATTACTTTTAAAAAGTAAGTATTTATAAGTAATTATATTTA : 5311
cen2-CR5 : ATAAAAAGTTAATATATAGTTATTAATTACTATTATATAGTTTAAAAGTTAATTATATAATTACTTTT-AAAAGTAAGTATTTATAAGTAATTATATTTA : 6521
cen3-CR1 : ATAAAAAGTTAATATATAATTATTAATTACTATTAAGTAATTTAAAAGTTAATTATATAATTACTTTT-AAAAATAAGTATTTATAAATAATTATATTTA : 6601
cen3-CR2 : ---------------------------------------------------------------------------------------------------- :    -
cen3-CR3 : ---------------------------------------------------------------------------------------------------- :    -
cen3-CR4 : ATAAAAAGTTAATATATAGCTATTAGTTATTATTAAGTAGTTTAAAGATTAATTATATAATTACTTTT-AAAAGTAAATATTTATAAATAATTATATTTA : 8055
cen4-CR1 : ATAAAAAGTTAATATATAATTATTAATTACTATTAAATAGTTTAAGAGTTAATTATATAATTACTTTT-AAAAGTAAGTATTTATAAGTAATTATATTTA : 5318
cen4-CR2 : ATAAAAAGTTAATATATAGTTATTAATTATTACTAAATAGTTTAAAAGTTAATTATATAATTACTTTT-AAAAGTAGGTGTTTATAAATAACTGTATTTA : 8229
cen4-CR3 : AAAAAAAGTTAATATATAGTTATTAATTATTATTAAGTAGTTTAAAAGTTAATTATATATTTACTTTT-AAAAGTAAGTATTTATAAATAATTATATTTA : 4763
cen4-CR4 : ATAAAAAGTTAATATATAGTTATTAATTATTACTAAGTAGTTTAAAAGTTAATTATATAATTACTTTT-AAAAGTAGGTGTTTATAAGTAATTATATTTA : 8325
cen4-CR5 : ATAAAAAGTTAATATATAGTTATTAATTATTACTAAGTAGTTTAAAAGTTAATTATATAATTACTTTT-AAAAGTAAGTATTTATAAATAATTATATTTA : 4922
cen5-CR1 : ---------------------------------------------------------------------------------------------------- :    -
cen6-CR1 : ATAAAAAGTTAATATATAATTATTAATTATTATTAAATAGTTTAAGAGTTAATTATATAATTACTTTT-AAAAGTAAGTATTTATAAGTAATTATATTTA : 8342
cen7-CR1 : ---------------------------------------------------------------------------------------------------- :    -
cen7-CR2 : ATAAAAAGTTAATATATAATTATTAATTATTATTAAGTAGTTTAAAGCTTAATTATATAATTACTTTT-AAAAGTAAGTGTTTATAAGTAATTATATTTA : 8122
cen7-CR3 : ---------------------------------------------------------------------------------------------------- :    -
           a aa aagtta tatata  tatta tta ta ta  t  t ta    ttaattatata ttactttt aaa  ta  t tttataa taa t tattta

                   *      8520         *      8540         *      8560         *      8580         *      8600
cen1-CR1 : TTTTT--AAACCTTTTTAAAATAAAAAAATAGTTTACTTTTTTTATAATTATAATAATATTATAAAGTTATAAGCACTTTATATTAAGGTTATTATAACT : 8271
cen1-CR2 : TTTTT--AAACCTCCTTAAAATAAAGGGATAGTTTACTCCTTTTATAGCTATAATAATACTATAAAGTTATAAGCACTTTATACTAAGGTTATTATAACC : 8385
cen1-CR3 : TTTTT--AAACCTTTTTAAAATAAAGGGATAGTTTACTTTTTTTATAGCTATAATAATGCTATAAAGTTATAAGCACTTTATGCTAAGGTTATTATAACC : 8430
cen1-CR4 : TTTTT--AAACCTCCTTAAAATAAAAAGATAGTTTACTTTTTTTATAGCTATAATAATGCTATAAAGTTATAAGCACTTTATGCTAAGGTTATTATAACC : 8122
cen1-CR5 : TTTTT--AAACCTTCTTAAAATAAAGGGATAGTTTACTCCTTTTATAGCTATAATAATACTATAAAGTTATAAGCACTTTATACTAAGGTTATTATAACC : 8190
cen2-CR1 : TTTTT--AAATCTTTTTAAAATAAAAGAATAGTTTACTTTTTTTATAATTATAATAATATTATAAAGTTATAAGCACTTTATATTAAGGTTATTATAACC : 7166
cen2-CR2 : TTTTT--AAACCTTTTTAAAATAAAGAGATAGTTTACTTTTTTTATAGCTATAATAGCGTTATAAAGTTATAAGCATTTTATATTAAAGTTATTATAACC : 8437
cen2-CR3 : TCTCT--AAACCTCCTTAAAATAAAGAAATAGTTTACTCTTTTTATAGCTATAATAACACTATAAAGTTATAAGCATTTTATACTAAGGTTATTATAACC : 8272
cen2-CR4 : TTTTTAAAAACCTTTTTAAAATAAAAAAATAATTTACT-TTTTTATAGCTATAATAATATTATAAAGTTATAAGTATTTTATATTAAAATTATTATAACC : 5410
cen2-CR5 : TTTTT--AAACCTTTTTAAAATAAAAAAATAGTTTAT--TTTTTATAGCTATAATAATATTATAAAGTTATAAGTATTTTATATTAAGGTTATTATAACT : 6617
cen3-CR1 : TTTTT--AAACCTTTTTAAAATAAAAAAATAGTTTACTTTTTTTATAATTATAATAATATTATAAAGTTATAAGCACTTTATATTAAGGTTATTATAACC : 6699
cen3-CR2 : ---------------------------------------------------------------------------------------------------- :    -
cen3-CR3 : ---------------------------------------------------------------------------------------------------- :    -
cen3-CR4 : TTTTT--AAACCTTTTTAAAATAAAAAAATAGTTTACTTTTTTTATAGCTATAATAATATTATAAAGTTATAAGCACTTTATATTAAGGTTATTATAACC : 8153
cen4-CR1 : TTTTT--AAACTTTTTTAAAATAAAAAAATAGTTTACTTTTTTTATAGCTATAATAATATTATAAAGTTATAAGTACTTTATATTAAGGTTATTATAACC : 5416
cen4-CR2 : TTTTT--AAACCTTTTTAAAATAAAAGGATAGTTTACTTTTTTTATAGCTATAATAATATTATAAAGTTATAAGCACTTTATACTAAGGTTATTATAACC : 8327
cen4-CR3 : TTTTTAAAAACCTTTTTAAAATAAAGAAATAATTTC---TTTTTATAATTATAATAATATTATAAAGTTATAAGCACTTTATATTAAAGTTATTATAATC : 4860
cen4-CR4 : TTTTT--AAACCTTTTTAAAATAAAGGGATAGTTTACTTTTTTTATAGCTATAATAATATTATAAAGTTATAAGCACTTTATACTAAGGTTATTATAACC : 8423
cen4-CR5 : TTTTT--AAATCTTTTTAAAATAAAAAGATAGTTTATT-TTTTTATAATTATAATAATATTATAAAGTTATAAGCATTTTATATTAAGGTTATTATAATT : 5019
cen5-CR1 : ---------------------------------------------------------------------------------------------------- :    -
cen6-CR1 : TTTTT--AAATCTTTTTAAAATAAAAGGATAATTTACTTTTTTTATAGCTATAATAATATTATAAAGTTATAAGCACTTTATATTAAGGTTATTATAACC : 8440
cen7-CR1 : ---------------------------------------------------------------------------------------------------- :    -
cen7-CR2 : TTTTT--AAACCTTTTTAAAATAAAAGAATAGTTTACTTTTTTTATAGCTATAATAATATTATAAAGTTATAAGTACTTTATATTAAGGTTATTATAACC : 8220
cen7-CR3 : ---------------------------------------------------------------------------------------------------- :    -
           t t t  aaa  t  ttaaaataaa   ata ttt     ttttata  tataata    tataaagttataag a tttat  taa  ttattataa
```

```
                   *      8620         *      8640         *      8660         *      8680         *      8700
cen1-CR1 : TTATATATATTTAATTTAACTTTATATTAAACTTTTTTTAAAAACTATGCCTTTTTATAAGGATTATATATTATAATTTTAATAATAATAAC-------- : 8363
cen1-CR2 : TTATATATATTTAATCTAACTTTATATTAAACTTTTTTTAAAAACTATGCCTTTTTATAAGGACTATATACTATAATCCTAATAA--------------- : 8470
cen1-CR3 : TTATATATATTTAATCTAACTTTATACTAAACTCCTTTTAAAGACTATGCCTTTTTATAAGGACTATATACTGTGATTTTAATATTAAGTTTTATAAAAA : 8530
cen1-CR4 : TTATATATATTTAATTTAACTTTATACTAAACTCCTTTTAAAGACTATGCCTTTTTATAAGGACTATATACTATAATCCTAACACTTACTATACTTAAAT : 8222
cen1-CR5 : TTATATATATTTAATCTAACTTTATACTAAACTCCTTTTAAAAACTATGCCTTTTTATAAGGACTATATACTATAATCCTAACATT-------------- : 8276
cen2-CR1 : TTATATATATTTAATTTAACTTTATATTAAACTTTTTTTAAAAACTATGCCTTTTTATAAAAACTATATATTATAATCTTAATAGATATTGAGAATGTCT : 7266
cen2-CR2 : TTATATATATTTAATTTAACTTTATACTAAACTCCTTTTAAAAACTATGCCTTTTTATAAGGACTATATACTATAATCCTAATAAAAATAATACTTTATA : 8537
cen2-CR3 : TTATATATATTTAGTCTGGCTTTATACTAAACTCCTTTTAAAAACTATGCCTTTTTATAAGGACTGCATACTATAATCCTAATAAATTACTATACTTATA : 8372
cen2-CR4 : TTATATATATTTAATTTAACTTTATATTAAACTCTTTTTAAAAACTATGCCTTTTTATTAAAACTATATACTATAATTCTAATA---------------- : 5494
cen2-CR5 : TTATATATATTTAATTTAACTTTATATTAAACTCTTTTTAAAAACTATGCCTTTTTATAAAAACTATATACTATAATCCTAATA-TATATTTAATTTTAA : 6716
cen3-CR1 : TTATATATATTTAATTTAATTTTATATTAAACTTCTTTTAAAGATTATACCTTTTTATAAAAACTATATATTATAATCCTAATA---------------- : 6783
cen3-CR2 : ---------------------------------------------------------------------------------------------------- :    -
cen3-CR3 : ---------------------------------------------------------------------------------------------------- :    -
cen3-CR4 : TTATATATATTTAATTTAACTTTATATTAAAGTCTTTTTAAGAACTATGCCTTTTTATAAAGACTATATATTATAATTTTAATATATACTTATATTATTA : 8253
cen4-CR1 : TTATATATATTTAATTTAACTTTATATTAAACTCCTTTTAAAGACTATGCTTTTTTATAAAGACTATATACTATAATCTTAATATATAATTACTTTTAAA : 5516
cen4-CR2 : TTATATATATTTAATTTAACTTTATATTAAACTCCTTTTAAAAACTATGCCTTTTTATAAGAACTATATACTATAATCTTAATAAATAATTTTAAAATAA : 8427
cen4-CR3 : TTATATATATTTAATTTAACTTTATATTAAACTCCTTTTAAAAACTATGCCTTTTTATTAAGACTATATACTATAATCTTAATAATAAAATTAATACTAA : 4960
cen4-CR4 : TTATATATATTTAATTTAATTTTATATTAAACTCCTTTTAAAAACTATGCTTTTTTATAAGGACTATATATTATAATTTTAATATAATTACT-------- : 8515
cen4-CR5 : TTATATATATTTAATTTAATTTTATATTAAACTCT----------------------------------------------------------------- : 5054
cen5-CR1 : ---------------------------------------------------------------------------------------------------- :    -
cen6-CR1 : TTATATATATTTAATCTAACTTTATATTAAACTCCTTTTAAAAACTATGCCTTTTTATAAGGACTGTATATTATAATCTTAATATTTTTATAAATAGC-- : 8538
cen7-CR1 : ---------------------------------------------------------------------------------------------------- :    -
cen7-CR2 : TTATATATATTTAATTTAACTTTATATTAAACTTTTTTTAAAAACTATACC-TTTTATAAGGACTATATATTATAATTTAAATAAAATAATATAAACTT- : 8318
cen7-CR3 : ---------------------------------------------------------------------------------------------------- :    -
           ttatatatattta t t   tttata taaa t

                   *      8720         *      8740         *      8760         *      8780         *      8800
cen1-CR1 : --------------------TTTTAACTTAATATAAA------------------------------------AAGTATT-----ATTATAAAGGCTATA : 8402
cen1-CR2 : -------------------------------------------------------------------------AATTATAAAAGCTATAAACTTATAATA : 8497
cen1-CR3 : TCTTTACTTTTAATAATTAATTTATAATTTTATAAAT------------------------------------AAATAATAAAAATATAATATTTTTTTT : 8594
cen1-CR4 : AGT------------------------------------------------------------------------------------------------- : 8225
cen1-CR5 : ---------------------------------------------------------------------------------------------------- :    -
cen2-CR1 : GTCAACGAAACAACTCCAATGAGCTTGCCTGACAAGA------------------------------------GAGCGCC-------------------- : 7310
cen2-CR2 : AATAGCCTTTATATA------------------------------------------------------------------------------------- : 8552
cen2-CR3 : CTAGTCCTTTTATA-------------------------------------------------------------------------------------- : 8386
cen2-CR4 : ---------------------------------------------------------------------------------------------------- :    -
cen2-CR5 : GTTTTTTTTTTATATTAATTTTCTTATAAATTTAATA------------------------------------AGTTATTACTTTTTTTATACTTTTTTT : 6780
cen3-CR1 : -------------------------------------------------------------------------AAAAGTAAGCTCGACTGGAGGCTGATA : 6810
cen3-CR2 : ---------------------------------------------------------------------------------------------------- :    -
cen3-CR3 : ---------------------------------------------------------------------------------------------------- :    -
cen3-CR4 : ATTTTTAAGCTAAATATTTATATATAATATAAATTAA------------------------------------TAATATTTGCTCGACTGGAGGCTGATA : 8317
cen4-CR1 : AATAAGTATTTATAAATAATTATATTTATTTTTAAAAACCTTTTTAAAATAAAAAAATAGTTTATTTTTTTATAATTATAATAATATTATAAAGTTATAA : 5616
cen4-CR2 : AAAAAAACTTTTTATTAATTTTATTATAATAAATATA------------------------------------TAAAAAGATATTATTTTAAAATTATTA : 8491
cen4-CR3 : AGTATTTACTAGTGCTGCCTTTACTACTGCCTTTATT------------------------------------ACTTTTACTACCTTTACTGCCTTTACT : 5024
cen4-CR4 : ---------------------------------------------------------------------------------------------------- :    -
cen4-CR5 : ---------------------------------------------------------------------------------------------------- :    -
cen5-CR1 : ---------------------------------------------------------------------------------------------------- :    -
cen6-CR1 : ---------------------------------------------------------------------------------------------------- :    -
cen7-CR1 : ---------------------------------------------------------------------------------------------------- :    -
cen7-CR2 : ---------------------------------------------------------------------------------------------------- :    -
cen7-CR3 : ---------------------------------------------------------------------------------------------------- :    -
```

```
                    *      8820         *      8840         *      8860
cen1-CR1 : TAAAATTACTAAAAAAGCTATAATTT---------------------------------------- : 8428
cen1-CR2 : ATAAACTTTATTAATATTATTATAAAT--------------------------------------- : 8524
cen1-CR3 : TATAATTTTATTTAATTTATAATAGTTTATA----------------------------------- : 8625
cen1-CR4 : ------------------------------------------------------------------ :    -
cen1-CR5 : ------------------------------------------------------------------ :    -
cen2-CR1 : ------------------------------------------------------------------ :    -
cen2-CR2 : ------------------------------------------------------------------ :    -
cen2-CR3 : ------------------------------------------------------------------ :    -
cen2-CR4 : -----TGATATTGAGAATGTCTGTCAACGAAACAACTCCAATGAGCTTGCCTGACAAGAGAGCGCC : 5555
cen2-CR5 : TAATTTGATATTGAGAATGTCTGTCAACGAAACAACTCCAATGAGCTTGCCTGACAAGAGAGCGCC : 6846
cen3-CR1 : TAAGGCTCCAGAATGCGGACTCGGCGCCTGAGGACATTATT------------------------- : 6851
cen3-CR2 : ------------------------------------------------------------------ :    -
cen3-CR3 : ------------------------------------------------------------------ :    -
cen3-CR4 : TAAGGCTCCAGAATGCGGACTCGGCGCCTGAGGACATTATT------------------------- : 8358
cen4-CR1 : GTATTTTATATTAAGGTTATTTTAACTTTATATATATTTAATTTAACTTTATATTAAAC------- : 5675
cen4-CR2 : TAAAAAAAGTAATAAAATATTTTTACTTTTATAATATAAATACTATATAA---------------- : 8541
cen4-CR3 : GCC--------------------------------------------------------------- : 5027
cen4-CR4 : ------------------------------------------------------------------ :    -
cen4-CR5 : ------------------------------------------------------------------ :    -
cen5-CR1 : ------------------------------------------------------------------ :    -
cen6-CR1 : ------------------------------------------------------------------ :    -
cen7-CR1 : ------------------------------------------------------------------ :    -
cen7-CR2 : ------------------------------------------------------------------ :    -
cen7-CR3 : ------------------------------------------------------------------ :    -
```
