# Supplementary material for: Trichoderma reesei complete genome sequence, repeat-induced point mutation, and partitioning of CAZyme gene clusters
Source: Biotechnol Biofuels. 2017 Jul 3;10:170. doi: 10.1186/s13068-017-0825-x (PMC5496416; doi:10.1186/s13068-017-0825-x)
Supplement: Supplementary file 5 — Additional file 5. Nucleotide sequences of the hgh alleles from all strains listed in Figure 7. [file 13068_2017_825_MOESM5_ESM.pdf]

Appendix A4: Nucleotide sequences of the *hgh* alleles from all strains listed in Figure 4.

>*blr1Δ::hph* (*MAT1-1*; F0)

ATGAAAAAGCCTGAACTCACCGCGACGTCTGTCGAGAAGTTCCTGATCGAAAAGTTCGACAGCGTCTCC
GACCTGATGCAGCTCTCGGAGGGCGAAGAATCTCGTGCTTTCAGCTTCGATGTAGGAGGGCGTGGATAT
GTCCTGCGGGTAAATAGCTGCGCCGATGGTTTCTACAAAGATCGTTATGTTTATCGGCACTTTGCATCG
GCCGCGCTCCCGATTCCGGAAGTGCTTGACATTGGGGAATTCAGCGAGAGCCTGACCTATTGCATCTCC
CGCCGTGCACAGGGTGTCACGTTGCAAGACCTGCCTGAAACCGAACTGCCCGCTGTTCTGCAGCCGGTC
GCGGAGGCCATGGATGCGATCGCTGCGGCCGATCTCAGCCAGACGAGCGGGTTCGGCCCATTCGGACCG
CAAGGAATCGGTCAATACACTACATGGCGTGATTTCATATGCGCGATTGCTGATCCCCATGTGTATCAC
TGGCAAACTGTGATGGACGACACCGTCAGTGCGTCCGTCGCGCAGGCTCTCGATGAGCTGATGCTTTGG
GCCGAGGACTGCCCCGAAGTCCGGCACCTCGTGCACGCGGATTTCGGCTCCAACAATGTCCTGACGGAC
AATGGCCGCATAACAGCGGTCATTGACTGGAGCGAGGCGATGTTCGGGGATTCCCAATACGAGGTCGCC
AACATCTTCTTCTGGAGGCCGTGGTTGGCTTGTATGGAGCAGCAGACGCGCTACTTCGAGCGGAGGCAC
CCGGAGCTTGCAGGATCGCCGCGGCTCCGGGCGTATATGCTCCGCATTGGTCTTGACCAACTCTATCAG
AGCTTGGTTGACGGCAATTTCGATGATGCAGCTTGCGCGCAGGGTCGATGCGACGCAATCGTCCGATCC
GGAGCCGGGACTGTCGGGCGTACACAAATCGCCCGCAGAAGCGCGGCCGTCTGGACCGATGGCTGTGTA
GAAGTACTCGCCGATAGTGGAAACCGACGCCCCAGCACTCGTCCGAGGGCAAAGGAATAG

>*blr1Δ::hph* (*MAT1-2*; F0)

ATGAAAAAGCCTGAACTCACCGCGACGTCTGTCGAGAAGTTCCTGATCGAAAAGTTCGACAGCGTCTCC
GACCTGATGCAGCTCTCGGAGGGCGAAGAATCTCGTGCTTTCAGCTTCGATGTAGGAGGGCGTGGATAT
GTCCTGCGGGTAAATAGCTGCGCCGATGGTTTCTACAAAGATCGTTATGTTTATCGGCACTTTGCATCG
GCCGCGCTCCCGATTCCGGAAGTGCTTGACATTGGGGAATTCAGCGAGAGCCTGACCTATTGCATCTCC
CGCCGTGCACAGGGTGTCACGTTGCAAGACCTGCCTGAAACCGAACTGCCCGCTGTTCTGCAGCCGGTC
GCGGAGGCCATGGATGCGATCGCTGCGGCCGATCTCAGCCAGACGAGCGGGTTCGGCCCATTCGGACCG
CAAGGAATCGGTCAATACACTACATGGCGTGATTTCATATGCGCGATTGCTGATCCCCATGTGTATCAC
TGGCAAACTGTGATGGACGACACCGTCAGTGCGTCCGTCGCGCAGGCTCTCGATGAGCTGATGCTTTGG
GCCGAGGACTGCCCCGAAGTCCGGCACCTCGTGCACGCGGATTTCGGCTCCAACAATGTCCTGACGGAC
AATGGCCGCATAACAGCGGTCATTGACTGGAGCGAGGCGATGTTCGGGGATTCCCAATACGAGGTCGCC
AACATCTTCTTCTGGAGGCCGTGGTTGGCTTGTATGGAGCAGCAGACGCGCTACTTCGAGCGGAGGCAC
CCGGAGCTTGCAGGATCGCCGCGGCTCCGGGCGTATATGCTCCGCATTGGTCTTGACCAACTCTATCAG
AGCTTGGTTGACGGCAATTTCGATGATGCAGCTTGCGCGCAGGGTCGATGCGACGCAATCGTCCGATCC
GGAGCCGGGACTGTCGGGCGTACACAAATCGCCCGCAGAAGCGCGGCCGTCTGGACCGATGGCTGTGTA
GAAGTACTCGCCGATAGTGGAAACCGACGCCCCAGCACTCGTCCGAGGGCAAAGGAATAG

>*blr1Δ* x *blr1Δ* (F1 #1)

ATGAAAAAGCCTGAACTCACCGCGACGTCTGTCGAGAAGTTCCTGATCGAAAAGTTCGACAGCGTCTCC

GACCTGATGCAGCTCTCGGAGGGCGAAGAATCTCGTGCTTTCAGCTTCGATATAGGAGGGCGTGGATAT
GTCCTGCGGGTAAATAGCTGCGCCGATGGTTTCTACAAAGATCGTTATGTTTATCGGCACTTTGCATCG
GCCGCGCTCCCGATTCCGGAAGTGCTTGACATTGGGGAATTCAGCGAGAGCCTGACCTATTGCATCTCC
CGCCGTGCACAGGGTGTCACGTTGCAAGACCTGCCTGAAACCGAACTGCCCGCTGTTCTGCAGCCGGTC
GCGGAGGCCATGGATGCGATCGCTGCGGCCGATCTCAGCCAGACGAGCGGGTTCGGCCCATTCGGACCG
TAAGGAATCGGTTAATACACTACATGGCGTGATTTTATATGCGCGATTGCTGATCCCCATATGTATTAC
TGGCAAACTGTGATGGACGACACTGTCAGTGCGTCCGTTACGCAGGCTCTCGATAAGCTGATGCTTTAG
GCCGAGGACTGCCCTGAAGTCCGGCACCTTGTGCATGCGGATTTCAGCTCTAATAATATCCTGATAGAT
AATGGCCGCATAACAGCGGTTATTAACTGGAGCGAGGCGATGTTTAGGGATTCCTAATACGAGGTCGCT
AACATCTTCTTCTAGAGGCCGTGGTTGGCTTATATGGAGCAGCAGATGCGCTACTTCGAGCGGAGGCAC
CTGGAGCTTGCAGGATCGCCGCGGCTCTGGGCGTATATGCTCTGCATTAGTCTTGACTAACTCTATTAG
AGCTTAGTTAACGGCAATTTTGATGATGCAGCTTAGGCGCAGGGTTAATGTAACGCAATCATCCGATCC
GGAGCCGGGACTGTTAGGCGTACATAAATCGCCTGCAGAAGCGCGGCCGTCTAGACTAATGGCTGTGTA
GAAGTACTTGCTAATAGTGGAAACCAACGCCCCAGCACTCATCCAAGGGCAAAGGAATAG

> *blr1Δ* x *blr1Δ* (F1 #2)
ATGAAAAAGCCTGAACTCACCGCGACGTCTGTCGAGAAGTTCCTGATCGAAAAGTTCGACAGCGTCTCC
GACCTGATGCAGCTCTCGGAGGGCGAAGAATCTCGTGCTTTCAGCTTCGATATAGGAGGGCGTGGATAT
GTCCTGCGGGTAAATAGCTGCGCCGATGGTTTCTACAAAGATCGTTATGTTTATCGGCACTTTGCATCG
GCCGCGCTCCCGATTCCGGAAGTGCTTGACATTGGGGAATTCAGCGAGAGCCTGACCTATTGCATCTCC
CGCCGTGCACAGGGTGTCACGTTGCAAGACCTGCCTGAAACCGAACTGCCCGCTGTTCTGCAGCCGGTC
GCGGAGGCCATGGATGCGATCGCTGCGGCCGATCTCAGCCAGACGAGCGGGTTCGGCCCATTCGGACCG
TAAGGAATCGGTTAATACACTACATGGCGTGATTTTATATGCGCGATTGCTGATCCCCATATGTATTAC
TGGCAAACTGTGATGGACGACACTGTCAGTGCGTCCGTTACGCAGGCTCTCGATAAGCTGATGCTTTAG
GCCGAGGACTGCCCTGAAGTCCGGCACCTTGTGCATGCGGATTTCAGCTCTAATAATATCCTGATAGAT
AATGGCCGCATAACAGCGGTTATTAACTGGAGCGAGGCGATGTTTAGGGATTCCTAATACGAGGTCGCT
AACATCTTCTTCTAGAGGCCGTGGTTGGCTTATATGGAGCAGCAGATGCGCTACTTCGAGCGGAGGCAC
CTGGAGCTTGCAGGATCGCCGCGGCTCTGGGCGTATATGCTCTGCATTAGTCTTGACTAACTCTATTAG
AGCTTAGTTAACGGCAATTTTGATGATGCAGCTTAGGCGCAGGGTTAATGTAACGCAATCATCCGATCC
GGAGCCGGGACTGTTAGGCGTACATAAATCGCCTGCAGAAGCGCGGCCGTCTAGACTAATGGCTGTGTA
GAAGTACTTGCTAATAGTGGAAACCAACGCCCCAGCACTCATCCAAGGGCAAAGGAATAG

> *blr1Δ* x *blr1Δ* (F1 #3)
ATGAAAAAGCCTGAACTCACCGCGACGTCTGTCGAGAAGTTCCTGATCGAAAAGTTCGACAGCGTCTCC
GACCTGATGCAGCTCTCGGAGGGCGAAGAATCTCGTGCTTTCAGCTTCGATGTAGGAGGGCGTGGATAT
GTCCTGCGGGTAAATAGCTGCGCCGATGGTTTCTACAAAGATCGTTATGTTTATCGGCACTTTGCATCG
GCCGCGCTCCCGATTCCGGAAGTGCTTGACATTGGGGAATTCAGCGAGAGCCTGACCTATTGCATCTCC
CGCCGTGCACAGGGTGTCACGTTGCAAGACCTGCCTGAAACCGAACTGCCCGCTGTTCTGCAGCCGGTC

GCGGAGGCCATGGATGCGATCGCTGCGGCCGATCTCAGCCAGACGAGCGGGTTCGGCCCATTCGGACCG
CAAGGAATCGGTCAATACACTACATAGCGTGATTTCATATGCGCGATTGCTGATCCCCATGTGTATTAC
TGGCAAACTGTGATAGACGACACCGTCAGTGCGTCCGTCACGCAGGCTCTTGATAAGCTGATGCTTTAG
GCCGAGGACTGCCCTGAAGTCCGGCACCTTGTGCACGCGGATTTCGGCTCTAACAATGTCCTAACGGAC
AATAGCTGCATAACAGCGGTCATTAACTAGAGCGAGGCGATATTCGGGGATTCCTAATACAAGGTCGCT
AATATCTTCTTCTAGAGGCTGTGGTTGGCTTATATAGAGCAGCAGACGCGCTACTTTAAGCAGAGGCAC
CTAGAGCTTGCAGGATTGCTGCGGCTCCGGGCGTATATGCTCCGCATTAGTCTTGACCAACTCTATCAG
AGCTTAGTTAACAGCAATTTTAATAATGCAGCTTAGGCGCAGGGTTGATGCGACGCAATCATCCAATCC
GGAGCCGGGACTGTCAGGCATATATAAATCGCCTGCAGAAGCGCAGCTGTCTAGACTAATGGCTGTGTA
GAAGTACTCGCTAATAGTAGAAACCGACGCCCCAGCACTTATCTAAGGGCAAAGGAATAA

> *blr1Δ* x *blr1Δ* (F1 #4)

ATGAAAAAGCCTGAACTCACCGCGACGTCTGTCGAGAAGTTCCTGATCGAAAAGTTCGACAGCGTCTCC
GACCTGATGCAGCTCTCGGAGGGCGAAGAATCTCGTGCTTTCAGCTTCGATGTAGGAGGGCGTGGATAT
GTCCTGCGGGTAAATAGCTGCGCCGATGGTTTCTACAAAGATCGTTATGTTTATCGGCACTTTGCATCG
GCCGCGCTCCCGATTCCGGAAGTGCTTGACATTGGGGAATTCAGCGAGAGCCTGACCTATTGCATCTCC
CGCCGTGCACAGGGTGTCACGTTGCAAGACCTGCCTGAAACCGAACTGCCCGCTGTTCTGCAGCCGGTC
GCGGAGGCCATGGATGCGATCGCTGCGGCCGATCTCAGCCAGACGAGCGGGTTCGGCCCATTCGGACCG
CAAGGAATCGGTCAATACACTACATAGCGTGATTTCATATGCGCGATTGCTGATCCCCATGTGTATTAC
TGGCAAACTGTGATAGACGACACCGTCAGTGCGTCCGTCACGCAGGCTCTTGATAAGCTGATGCTTTAG
GCCGAGGACTGCCCTGAAGTCCGGCACCTTGTGCACGCGGATTTCGGCTCTAACAATGTCCTAACGGAC
AATAGCTGCATAACAGCGGTCATTAACTAGAGCGAGGCGATATTCGGGGATTCCTAATACAAGGTCGCT
AATATCTTCTTCTAGAGGCTGTGGTTGGCTTATATAGAGCAGCAGACGCGCTACTTTAAGCAGAGGCAC
CTAGAGCTTGCAGGATTGCTGCGGCTCCGGGCGTATATGCTCCGCATTAGTCTTGACCAACTCTATCAG
AGCTTAGTTAACAGCAATTTTAATAATGCAGCTTAGGCGCAGGGTTGATGCGACGCAATCATCCAATCC
GGAGCCGGGACTGTCAGGCATATATAAATCGCCTGCAGAAGCGCAGCTGTCTAGACTAATGGCTGTGTA
GAAGTACTCGCTAATAGTAGAAACCGACGCCCCAGCACTTATCTAAGGGCAAAGGAATAA

> *blr1Δ* x *blr1* (F1 #4)

ATGAAAAAGCCTGAACTCACCGCGACGTCTGTCGAGAAGTTCCTGATCGAAAAGTTCGACAGCGTCTCC
GACCTGATGCAGCTCTCGGAGGGCGAAGAATCTCGTGCTTTCAGCTTCGATGTAGGAGGGCGTGGATAT
GTCCTGCGGGTAAATAGCTGCGCCGATGGTTTCTACAAAGATCGTTATGTTTATCGGCACTTTGCATCG
GCCGCGCTCCCGATTCCGGAAGTGCTTGACATTGGGGAATTCAGCGAGAGCCTGACCTATTGCATCTCC
CGCCGTGCACAGGGTGTCACGTTGCAAGACCTGCCTGAAACCGAACTGCCCGCTGTTCTGCAGCCGGTC
GCGGAGGCCATGGATGCGATCGCTGCGGCCGATCTCAGCCAGACGAGCGGGTTCGGCCTATTCGGACTG
CAAGGAATCGGTTAATACACTACATGGCGTGATTTTATATGCGCGATTGCTGATCCCTATGTGTATTAC
TGGCAAACTGTGATAGACGATACTGTCAGTGCGTCCGTTGCGCAGGCTCTTAATGAGCTAATGCTTTAG
GCCGAGGACTGCCCTGAAGTCCGGCACCTTGTGCATGCGGATTTCGGCTCTAATAATGTCCTGACGGAC

AATGGCCGCATAACAGCAGTCATTAACTGGAGCGAGGCGATGTTCGGGGATTCCTAATACGAGGTCGCT
AACATCTTCTTCTGGAGGCCGTGGTTGGCTTGTATGGAGCAATAGACGCGCTACTTTGAGCGGAGGCAC
CCGGAGCTTGCACGATTGCCGCGGCTCTGGGCGTATATGCTCTGCATTGGTCTTGACTAACTCTATTAG
AGCTTGGTTAACGGCAATTTTGATGATGCAGCTTAGGCGCAGGGTTGATGCGACGCAATTGTCTGATCC
GGAGCCGGGACTGTTAGGCGTATACAAATCGCCTGCAGAAGCGCGGCCGTCTGGACTGATAGCTGTGTA
GAAGTACTTGCCGATAGTGGAAACCGACGCCCCAGCACTTGTCCGAGGGTAAAGGAATAG

>*env1Δ* (*MAT1-1*; F0)

ATGAAAAAGCCTGAACTCACCGCGACGTCTGTCGAGAAGTTCCTGATCGAAAAGTTCGACAGCGTCTCC
GACCTGATGCAGCTCTCGGAGGGCGAAGAATCTCGTGCTTTCAGCTTCGATGTAGGAGGGCGTGGATAT
GTCCTGCGGGTAAATAGCTGCGCCGATGGTTTCTACAAAGATCGTTATGTTTATCGGCACTTTGCATCG
GCCGCGCTCCCGATTCCGGAAGTGCTTGACATTGGGGAATTCAGCGAGAGCCTGACCTATTGCATCTCC
CGCCGTGCACAGGGTGTCACGTTGCAAGACCTGCCTGAAACCGAACTGCCCGCTGTTCTGCAGCCGGTC
GCGGAGGCCATGGATGCGATCGCTGCGGCCGATCTCAGCCAGACGAGCGGGTTCGGCCCATTCGGACCG
CAAGGAATCGGTCAATACACTACATGGCGTGATTTCATATGCGCGATTGCTGATCCCCATGTGTATCAC
TGGCAAACTGTGATGGACGACACCGTCAGTGCGTCCGTCGCGCAGGCTCTCGATGAGCTGATGCTTTGG
GCCGAGGACTGCCCCGAAGTCCGGCACCTCGTGCACGCGGATTTCGGCTCCAACAATGTCCTGACGGAC
AATGGCCGCATAACAGCGGTCATTGACTGGAGCGAGGCGATGTTCGGGGATTCCCAATACGAGGTCGCC
AACATCTTCTTCTGGAGGCCGTGGTTGGCTTGTATGGAGCAGCAGACGCGCTACTTCGAGCGGAGGCAC
CCGGAGCTTGCAGGATCGCCGCGGCTCCGGGCGTATATGCTCCGCATTGGTCTTGACCAACTCTATCAG
AGCTTGGTTGACGGCAATTTCGATGATGCAGCTTGGGCGCAGGGTCGATGCGACGCAATCGTCCGATCC
GGAGCCGGGACTGTCGGGCGTACACAAATCGCCCGCAGAAGCGCGGCCGTCTGGACCGATGGCTGTGTA
GAAGTACTCGCCGATAGTGGAAACCGACGCCCCAGCACTCGTCCGAGGGCAAAGGAATAG

>*env1Δ* (*MAT1-2*; F0)

ATGAAAAAGCCTGAACTCACCGCGACGTCTGTCGAGAAGTTCCTGATCGAAAAGTTCGACAGCGTCTCC
GACCTGATGCAGCTCTCGGAGGGCGAAGAATCTCGTGCTTTCAGCTTCGATGTAGGAGGGCGTGGATAT
GTCCTGCGGGTAAATAGCTGCGCCGATGGTTTCTACAAAGATCGTTATGTTTATCGGCACTTTGCATCG
GCCGCGCTCCCGATTCCGGAAGTGCTTGACATTGGGGAATTCAGCGAGAGCCTGACCTATTGCATCTCC
CGCCGTGCACAGGGTGTCACGTTGCAAGACCTGCCTGAAACCGAACTGCCCGCTGTTCTGCAGCCGGTC
GCGGAGGCCATGGATGCGATCGCTGCGGCCGATCTCAGCCAGACGAGCGGGTTCGGCCCATTCGGACCG
CAAGGAATCGGTCAATACACTACATGGCGTGATTTCATATGCGCGATTGCTGATCCCCATGTGTATCAC
TGGCAAACTGTGATGGACGACACCGTCAGTGCGTCCGTCGCGCAGGCTCTCGATGAGCTGATGCTTTGG
GCCGAGGACTGCCCCGAAGTCCGGCACCTCGTGCACGCGGATTTCGGCTCCAACAATGTCCTGACGGAC
AATGGCCGCATAACAGCGGTCATTGACTGGAGCGAGGCGATGTTCGGGGATTCCCAATACGAGGTCGCC
AACATCTTCTTCTGGAGGCCGTGGTTGGCTTGTATGGAGCAGCAGACGCGCTACTTCGAGCGGAGGCAC
CCGGAGCTTGCAGGATCGCCGCGGCTCCGGGCGTATATGCTCCGCATTGGTCTTGACCAACTCTATCAG
AGCTTGGTTGACGGCAATTTCGATGATGCAGCTTGGGCGCAGGGTCGATGCGACGCAATCGTCCGATCC

GGAGCCGGGACTGTCGGGCGTACACAAATCGCCCGCAGAAGCGCGGCCGTCTGGACCGATGGCTGTGTA
GAAGTACTCGCCGATAGTGGAAACCGACGCCCCAGCACTCGTCCGAGGGCAAAGGAATAG

> *blr1Δ* x *env1Δ* (F1 #1, *blr1 Δ::hph*)

ATGAAAAAGCCTGAACTCACCGCGACGTCTGTCGAGAAGTTCCTGATCGAAAAGTTCGACAGCGTCTCC
GACCTGATGCAGCTCTCGGAGGGCGAAGAATCTCGTGCTTTCAGCTTCGATGTAGGAGGGCGTGGATAT
GTCCTGCGGGTAAATAGCTGCGCCGATGGTTTCTACAAAGATCGTTATGTTTATCGGCACTTTGCATCG
GCCGCGCTCCCGATTCCGGAAGTGCTTGACATTGGGGAATTCAGCGAGAGCCTGACCTATTGCATCTCC
CGCCGTGCACAGGGTGTCACGTTGCAAGACCTGCCTGAAACCGAACTGCCCGCTGTTCTGCAGCCGGTC
GCGGAGGCCATGGATGCGATCGCTGCGGCCGATCTCAGCCAGACGAGCGGGTTCGGCCCATTCGGACCG
CAAGGAATCGGTCAATACACTACATGGCGTGATTTCATATGCGCGATTGCTGATCCCCATGTGTATCAC
TGGCAAACTGTGATGGACGACACCGTCAGTGCGTCCGTCGCGCAGGCTCTTGATGAGCTGATGCTTTGG
GCCGAGGACTGCCCCGAAGTCCGGCACCTCGTGCACGCGGATTTCGGCTCTAATAATGTCCTGACGGAC
AATGGCCGCATAACAGCGGTTATTGACTGGAGCGAGGCGATGTTTGGGGATTCCTAATACGAGGTCGCT
AACATCTTCTTCTGGAGGCCGTGGTTGGCTTGTATGGAGCAGCAGACGCGCTACTTTGAGCGGAGGCAC
CCGGAGCTTGCAGGATCGCCGCGGCTCCGGGTGTATATGCTCCGCATTGGTCTTGACTAACTCTATCAG
AGCTTGGTTGACGGCAATTTCGATGATGCAGCTTGGGCGCAGGGTTGATGCGACGCAATTGTCTGATCC
GGAGCTGGGACTGTCGGGCGTATACAAATCGCCTGCAGAGGCGCGGCCGTCTGGACTGATGGCTGTGTA
GAAGTACTCGCCGATAGTGGAAACCGACGCCCCAGCACTTGTCCGAGGGCAAAGGAATAG

> *blr1Δ* x *env1Δ* (F1 #2, *blr1 Δ::hph*)

ATGAAAAAGCCTGAACTCACCGCGACGTCTGTCGAGAAGTTCCTGATCGAAAAGTTCGACAGCGTCTCC
GACCTGATGCAGCTCTCGGAGGGCGAAGAATCTCGTGCTTTCAGCTTCGATGTAGGAGGGCGTGGATAT
GTCCTGCGGGTAAATAGCTGCGCCGATGGTTTCTACAAAGATCGTTATGTTTATCGGCACTTTGCATCG
GCCGCGCTCCCGATTCCGGAAGTGCTTGACATTGGGGAATTCAGCGAGAGCCTGACCTATTGCATCTCC
CGCCGTGCACAGGGTGTCACGTTGCAAGACCTGCCTGAAACCGAACTGCCCGCTGTTCTGCAGCCGGTC
GCGGAGGCCATGGATGCGATCGCTGCGGCCGATCTCAGCCAGACGAGCGGGTTCGGCCCATTCGGACCG
CAAGGAATCGGTCAATACACTACATGGCGTGATTTCATATGCGCGATTGCTGATCCCCATGTGTATCAC
TGGCAAACTGTGATGGACGACACCGTCAGTGCGTCCGTCGCGCAGGCTCTTGATGAGCTGATGCTTTGG
GCCGAGGACTGCCCCGAAGTCCGGCACCTCGTGCACGCGGATTTCGGCTCTAATAATGTCCTGACGGAC
AATGGCCGCATAACAGCGGTTATTGACTGGAGCGAGGCGATGTTTGGGGATTCCTAATACGAGGTCGCT
AACATCTTCTTCTGGAGGCCGTGGTTGGCTTGTATGGAGCAGCAGACGCGCTACTTTGAGCGGAGGCAC
CCGGAGCTTGCAGGATCGCCGCGGCTCCGGGTGTATATGCTCCGCATTGGTCTTGACTAACTCTATCAG
AGCTTGGTTGACGGCAATTTCGATGATGCAGCTTGGGCGCAGGGTTGATGCGACGCAATTGTCTGATCC
GGAGCTGGGACTGTCGGGCGTATACAAATCGCCTGCAGAGGCGCGGCCGTCTGGACTGATGGCTGTGTA
GAAGTACTCGCCGATAGTGGAAACCGACGCCCCAGCACTTGTCCGAGGGCAAAGGAATAG

> *blr1Δ* x *env1Δ* (F1 #1, *env1Δ::hph*)

ATGAAAAAGCCTGAACTCACCGCGACGTCTGTCGAGAAGTTCCTGATCGAAAAGTTCGACAGCGTCTCC
GACCTGATGCAGCTCTCGGAGGGCGAAGAATCTCGTGCTTTCAGCTTCGATGTAGGAGGGCGTGGATAT
GTCCTGCGGGTAAATAGCTGCGCCGATGGTTTCTACAAAGATCGTTATGTTTATCGGCACTTTGCATCG
GCCGCGCTCCCGATTCCGGAAGTGCTTGACATTGGGGAATTCAGCGAGAGCCTGACCTATTGCATCTCC
CGCCGTGCACAGGGTGTCACGTTGCAAGACCTGCCTGAAACCGAACTGCCCGCTGTTCTGCAGCCGGTC
GCGGAGGCCATGGATGCGATCGCTGCGGCCGATCTCAGCCAGACGAGCGGGTTCGGCCCATTCGGACCG
CAAGGAATCGGTCAATACACTACATGGCGTGATTTCATATGCGCGATTGCTGATCCCCATGTGTATCAC
TGGCAAACTGTGATGGACGACACCGTCAGTGCGTCCGTCGCGCAGGCTCTCGATGAGCTGATGCTTTGG
GCCGAGGACTGCCCCGAAGTCCGGCACCTCGTGCACGCGGATTTCGGCTCCAACAATGTCCTGACGGAC
AATGGCCGCATAACAGCGGTCATTGACTGGAGCGAGGCGATGTTCGGGGATTCCCAATACGAGGTCGCC
AACATCTTCTTCTGGAGGCCGTGGTTGGCTTGTATGGAGCAGCAGACGCGCTACTTCGAGCGGAGGCAC
CCGGAGCTTGCAGGATCGCCGCGGCTCCGGGCGTATATGCTCCGCATTGGTCTTGACCAACTCTATCAG
AGCTTGGTTGACGGCAATTTCGATGATGCAGCTTGGGCGCAGGGTCGATGCGACGCAATCGTCCGATCC
GGAGCCGGGACTGTCGGGCGTACACAAATCGCCCGCAGAAGCGCGGCCGTCTGGACCGATGGCTGTGTA
GAAGTACTCGCCGATAGTGGAAACCGACGCCCCAGCACTCGTCCGAGGGCAAAGGAATAG

> *blr1Δ* x *env1Δ* (F1 #4, *env1Δ::hph*)

ATGAAAAAGCCTGAACTCACCGCGACGTCTGTCGAGAAGTTCCTGATCGAAAAGTTCGACAGCGTCTCC
GACCTGATGCAGCTCTCGGAGGGCGAAGAATCTCGTGCTTTCAGCTTCGATGTAGGAGGGCGTGGATAT
GTCCTGCGGGTAAATAGCTGCGCCGATGGTTTCTACAAAGATCGTTATGTTTATCGGCACTTTGCATCG
GCCGCGCTCCCGATTCCGGAAGTGCTTGACATTGGGGAATTCAGCGAGAGCCTGACCTATTGCATCTCC
CGCCGTGCACAGGGTGTCACGTTGCAAGACCTGCCTGAAACCGAACTGCCCGCTGTTCTGCAGCCGGTC
GCGGAGGCCATGGATGCGATCGCTGCGGCCGATCTCAGCCAGACGAGCGGGTTCGGCCCATTCGGACCG
CAAGGAATCGGTCAATACACTACATGGCGTGATTTCATATGCGCGATTGCTGATCCCCATGTGTATCAC
TGGCAAACTGTGATGGACGACACCGTCAGTGCGTCCGTCGCGCAGGCTCTCGATGAGCTGATGCTTTGG
GCCGAGGACTGCCCCGAAGTCCGGCACCTCGTGCACGCGGATTTCGGCTCCAACAATGTCCTGACGGAC
AATGGCCGCATAACAGCGGTCATTGACTGGAGCGAGGCGATGTTCGGGGATTCCCAATACGAGGTCGCC
AACATCTTCTTCTGGAGGCCGTGGTTGGCTTGTATGGAGCAGCAGACGCGCTACTTCGAGCGGAGGCAC
CCGGAGCTTGCAGGATCGCCGCGGCTCCGGGCGTATATGCTCCGCATTGGTCTTGACCAACTCTATCAG
AGCTTGGTTGACGGCAATTTCGATGATGCAGCTTGGGCGCAGGGTCGATGCGACGCAATCGTCCGATCC
GGAGCCGGGACTGTCGGGCGTACACAAATCGCCCGCAGAAGCGCGGCCGTCTGGACCGATGGCTGTGTA
GAAGTACTCGCCGATAGTGGAAACCGACGCCCCAGCACTCGTCCGAGGGCAAAGGAATAG

> *env1Δ* x *env1Δ* (F1 #1)

ATGAAAAAGCCTGAACTCACCGCGACGTCTGTCGAGAAGTTCCTGATCGAAAAGTTCGACAGCGTCTCC
GACCTGATGCAGCTCTCGGAGGGCGAAGAATCTCGTGCTTTCAGCTTCGATGTAGGAGGGCGTGGATAT
GTCCTGCGGGTAAATAGCTGCGCCGATGGTTTCTACAAAGATCGTTATGTTTATCGGCACTTTGCATCG
GCCGCGCTCCCGATTCCGGAAGTGCTTGACATTGGGGAATTCAGCGAGAGCCTGACCTATTGCATCTCC

CGCCGTGCACAGGGTGTCACGTTGCAAGACCTGCCTGAAACCGAACTGCCCGCTGTTCTGCAGCCGGTC

GCGGAGGCCATGGATGCGATCGCTGCGGCCGATCTCAGCCAGACGAGCGGGTTCGGCCCATTCGGACCG

CAAGGAATCGGTCAATACACTACATGGCGTGATTTCATATGCGCGATTGCTGATCCCCATGTGTATCAC

TGGCAAACTGTGATGGACGACACCGTCAGTGCGTCCGTCGCGCAGGCTCTCGATGAGCTGATGCTTTGG

GCCGAGGACTGCCCCGAAGTCCGGCACCTCGTGCACGCGGATTTCGGCTCCAACAATGTCCTGACGGAC

AATGGCCGCATAACAGCGGTCATTGACTGGAGCGAGGCGATGTTCGGGGATTCCCAATACGAGGTCGCC

AACATCTTCTTCTGGAGGCCGTGGTTGGCTTGTATGGAGCAGCAGACGCGCTACTTCGAGCGGAGGCAC

CCGGAGCTTGCAGGATCGCCGCGGCTCCGGGCGTATATGCTCCGCATTGGTCTTGACCAACTCTATCAG

AGCTTGGTTGACGGCAATTTCGATGATGCAGCTTGGGCGCAGGGTCGATGCGACGCAATCGTCCGATCC

GGAGCCGGGACTGTCGGGCGTACACAAATCGCCCGCAGAAGCGCGGCCGTCTGGACCGATGGCTGTGTA

GAAGTACTCGCCGATAGTGGAAACCGACGCCCCAGCACTCGTCCGAGGGCAAAGGAATAG

> ***env1Δ* x *env1Δ* (F1 #2)**

ATGAAAAAGCCTGAACTCACCGCGACGTCTGTCGAGAAGTTCCTGATCGAAAAGTTCGACAGCGTCTCC

GACCTGATGCAGCTCTCGGAGGGCGAAGAATCTCGTGCTTTCAGCTTCGATGTAGGAGGGCGTGGATAT

GTCCTGCGGGTAAATAGCTGCGCCGATGGTTTCTACAAAGATCGTTATGTTTATCGGCACTTTGCATCG

GCCGCGCTCCCGATTCCGGAAGTGCTTGACATTGGGGAATTCAGCGAGAGCCTGACCTATTGCATCTCC

CGCCGTGCACAGGGTGTCACGTTGCAAGACCTGCCTGAAACCGAACTGCCCGCTGTTCTGCAGCCGGTC

GCGGAGGCCATGGATGCGATCGCTGCGGCCGATCTCAGCCAGACGAGCGGGTTCGGCCCATTCGGACCG

CAAGGAATCGGTCAATACACTACATGGCGTGATTTCATATGCGCGATTGCTGATCCCCATGTGTATCAC

TGGCAAACTGTGATGGACGACACCGTCAGTGCGTCCGTCGCGCAGGCTCTCGATGAGCTGATGCTTTGG

GCCGAGGACTGCCCCGAAGTCCGGCACCTCGTGCACGCGGATTTCGGCTCCAACAATGTCCTGACGGAC

AATGGCCGCATAACAGCGGTCATTGACTGGAGCGAGGCGATGTTCGGGGATTCCCAATACGAGGTCGCC

AACATCTTCTTCTGGAGGCCGTGGTTGGCTTGTATGGAGCAGCAGACGCGCTACTTCGAGCGGAGGCAC

CCGGAGCTTGCAGGATCGCCGCGGCTCCGGGCGTATATGCTCCGCATTGGTCTTGACCAACTCTATCAG

AGCTTGGTTGACGGCAATTTCGATGATGCAGCTTGGGCGCAGGGTCGATGCGACGCAATCGTCCGATCC

GGAGCCGGGACTGTCGGGCGTACACAAATCGCCCGCAGAAGCGCGGCCGTCTGGACCGATGGCTGTGTA

GAAGTACTCGCCGATAGTGGAAACCGACGCCCCAGCACTCGTCCGAGGGCAAAGGAATAG

> ***env1Δ* x *env1Δ* (F1 #3)**

ATGAAAAAGCCTGAACTCACCGCGACGTCTGTCGAGAAGTTCCTGATCGAAAAGTTCGACAGCGTCTCC

GACCTGATGCAGCTCTCGGAGGGCGAAGAATCTCGTGCTTTCAGCTTCGATGTAGGAGGGCGTGGATAT

GTCCTGCGGGTAAATAGCTGCGCCGATGGTTTCTACAAAGATCGTTATGTTTATCGGCACTTTGCATCG

GCCGCGCTCCCGATTCCGGAAGTGCTTGACATTGGGGAATTCAGCGAGAGCCTGACCTATTGCATCTCC

CGCCGTGCACAGGGTGTCACGTTGCAAGACCTGCCTGAAACCGAACTGCCCGCTGTTCTGCAGCCGGTC

GCGGAGGCCATGGATGCGATCGCTGCGGCCGATCTCAGCCAGACGAGCGGGTTCGGCCCATTCGGACCG

CAAGGAATCGGTCAATACACTACATGGCGTGATTTCATATGCGCGATTGCTGATCCCCATGTGTATCAC

TGGCAAACTGTGATGGACGACACCGTCAGTGCGTCCGTCGCGCAGGCTCTCGATGAGCTGATGCTTTGG

GCCGAGGACTGCCCCGAAGTCCGGCACCTCGTGCACGCGGATTTCGGCTCCAACAATGTCCTGACGGAC

AATGGCCGCATAACAGCGGTCATTGACTGGAGCGAGGCGATGTTCGGGGATTCCCAATACGAGGTCGCC

AACATCTTCTTCTGGAGGCCGTGGTTGGCTTGTATGGAGCAGCAGACGCGCTACTTCGAGCGGAGGCAC

CCGGAGCTTGCAGGATCGCCGCGGCTCCGGGCGTATATGCTCCGCATTGGTCTTGACCAACTCTATCAG

AGCTTGGTTGACGGCAATTTCGATGATGCAGCTTGGGCGCAGGGTCGATGCGACGCAATCGTCCGATCC

GGAGCCGGGACTGTCGGGCGTACACAAATCGCCCGCAGAAGCGCGGCCGTCTGGACCGATGGCTGTGTA

GAAGTACTCGCCGATAGTGGAAACCGACGCCCCAGCACTCGTCCGAGGGCAAAGGAATAG

> *env1Δ* x *env1Δ* (F1 #4)

ATGAAAAAGCCTGAACTCACCGCGACGTCTGTCGAGAAGTTCCTGATCGAAAAGTTCGACAGCGTCTCC

GACCTGATGCAGCTCTCGGAGGGCGAAGAATCTCGTGCTTTCAGCTTCGATGTAGGAGGGCGTGGATAT

GTCCTGCGGGTAAATAGCTGCGCCGATGGTTTCTACAAAGATCGTTATGTTTATCGGCACTTTGCATCG

GCCGCGCTCCCGATTCCGGAAGTGCTTGACATTGGGGAATTCAGCGAGAGCCTGACCTATTGCATCTCC

CGCCGTGCACAGGGTGTCACGTTGCAAGACCTGCCTGAAACCGAACTGCCCGCTGTTCTGCAGCCGGTC

GCGGAGGCCATGGATGCGATCGCTGCGGCCGATCTCAGCCAGACGAGCGGGTTCGGCCCATTCGGACCG

CAAGGAATCGGTCAATACACTACATGGCGTGATTTCATATGCGCGATTGCTGATCCCCATGTGTATCAC

TGGCAAACTGTGATGGACGACACCGTCAGTGCGTCCGTCGCGCAGGCTCTCGATGAGCTGATGCTTTGG

GCCGAGGACTGCCCCGAAGTCCGGCACCTCGTGCACGCGGATTTCGGCTCCAACAATGTCCTGACGGAC

AATGGCCGCATAACAGCGGTCATTGACTGGAGCGAGGCGATGTTCGGGGATTCCCAATACGAGGTCGCC

AACATCTTCTTCTGGAGGCCGTGGTTGGCTTGTATGGAGCAGCAGACGCGCTACTTCGAGCGGAGGCAC

CCGGAGCTTGCAGGATCGCCGCGGCTCCGGGCGTATATGCTCCGCATTGGTCTTGACCAACTCTATCAG

AGCTTGGTTGACGGCAATTTCGATGATGCAGCTTGGGCGCAGGGTCGATGCGACGCAATCGTCCGATCC

GGAGCCGGGACTGTCGGGCGTACACAAATCGCCCGCAGAAGCGCGGCCGTCTGGACCGATGGCTGTGTA

GAAGTACTCGCCGATAGTGGAAACCGACGCCCCAGCACTCGTCCGAGGGCAAAGGAATAG

>*ku70Δ* (*MAT1-1*; F0)

ATGAAAAAGCCTGAACTCACCGCGACGTCTGTCGAGAAGTTTCTGATCGAAAAGTTCGACAGCGTCTCC

GACCTGATGCAGCTCTCGGAGGGCGAAGAATCTCGTGCTTTCAGCTTCGATGTAGGAGGGCGTGGATAT

GTCCTGCGGGTAAATAGCTGCGCCGATGGTTTCTACAAAGATCGTTATGTTTATCGGCACTTTGCATCG

GCCGCGCTCCCGATTCCGGAAGTGCTTGACATTGGGGAATTCAGCGAGAGCCTGACCTATTGCATCTCC

CGCCGTGCACAGGGTGTCACGTTGCAAGACCTGCCTGAAACCGAACTGCCCGCTGTTCTGCAGCCGGTC

GCGGAGGCCATGGATGCGATCGCTGCGGCCGACCTTAGCCAGACGAGCGGGTTCGGCCCATTCGGACCG

CAAGGAATCGGTCAATACACTACATGGCGTGATTTCATATGCGCGATTGCTGATCCCCATGTGTATCAC

TGGCAAACTGTGATGGACGACACCGTCAGTGCGTCCGTCGCGCAGGCTCTCGATGAGCTGATGCTTTGG

GCCGAGGACTGCCCCGAAGTCCGGCACCTCGTGCACGCGGATTTCGGCTCCAACAATGTCCTGACGGAC

AATGGCCGCATAACAGCGGTCATTGACTGGAGCGAGGCGATGTTCGGGGATTCCCAATACGAGGTCGCC

AACATCTTCTTCTGGAGGCCGTGGTTGGCTTGTATGGAGCAGCAGACGCGCTACTTCGAGCGGAGGCAT

CCGGAGCTTGCAGGATCGCCGCGGCTCCGGGCGTATATGCTCCGCATTGGTCTTGACCAACTCTATCAG

AGCTTGGTTGACGGCAATTTCGATGATGCAGCTTGAGCGCAGGGTCGATGCGACGCAATCGTCCGATCC
GGAGCCGGGACTGTCGGGCGTACACAAATCGCCCGCAGAAGCGCGGCCGTCTGGACCGATGGCTGTGTA
GAAGTACTCGCCGATAGTGGAAACCGACGCCCCAGCACTCGTCCGAGGGCAAAGGAATAG

>*ku70Δ* (*MAT1-2*; F0)

ATGAAAAAGCCTGAACTCACCGCGACGTCTGTCGAGAAGTTTCTGATCGAAAAGTTCGACAGCGTCTCC
GACCTGATGCAGCTCTCGGAGGGCGAAGAATCTCGTGCTTTCAGCTTCGATGTAGGAGGGCGTGGATAT
GTCCTGCGGGTAAATAGCTGCGCCGATGGTTTCTACAAAGATCGTTATGTTTATCGGCACTTTGCATCG
GCCGCGCTCCCGATTCCGGAAGTGCTTGACATTGGGGAATTCAGCGAGAGCCTGACCTATTGCATCTCC
CGCCGTGCACAGGGTGTCACGTTGCAAGACCTGCCTGAAACCGAACTGCCCGCTGTTCTGCAGCCGGTC
GCGGAGGCCATGGATGCGATCGCTGCGGCCGACCTTAGCCAGACGAGCGGGTTCGGCCCATTCGGACCG
CAAGGAATCGGTCAATACACTACATGGCGTGATTTCATATGCGCGATTGCTGATCCCCATGTGTATCAC
TGGCAAACTGTGATGGACGACACCGTCAGTGCGTCCGTCGCGCAGGCTCTCGATGAGCTGATGCTTTGG
GCCGAGGACTGCCCCGAAGTCCGGCACCTCGTGCACGCGGATTTCGGCTCCAACAATGTCCTGACGGAC
AATGGCCGCATAACAGCGGTCATTGACTGGAGCGAGGCGATGTTCGGGGATTCCCAATACGAGGTCGCC
AACATCTTCTTCTGGAGGCCGTGGTTGGCTTGTATGGAGCAGCAGACGCGCTACTTCGAGCGGAGGCAT
CCGGAGCTTGCAGGATCGCCGCGGCTCCGGGCGTATATGCTCCGCATTGGTCTTGACCAACTCTATCAG
AGCTTGGTTGACGGCAATTTCGATGATGCAGCTTGAGCGCAGGGTCGATGCGACGCAATCGTCCGATCC
GGAGCCGGGACTGTCGGGCGTACACAAATCGCCCGCAGAAGCGCGGCCGTCTGGACCGATGGCTGTGTA
GAAGTACTCGCCGATAGTGGAAACCGACGCCCCAGCACTCGTCCGAGGGCAAAGGAATAG

> *env1Δ* x *ku70Δ* (F1 #1, *env1Δ::hph*)

ATGAAAAAGCCTGAACTCACCGCGACGTCTGTCGAGAAGTTCCTGATCGAAAAGTTCGACAGCGTCTCC
GACCTGATGCAGCTCTCGGAGGGCGAAGAATCTCGTGCTTTCAGCTTCGATGTAGGAGGGCGTGGATAT
GTCCTGCGGGTAAATAGCTGCGCCGATGGTTTCTACAAAGATCGTTATGTTTATCGGCACTTTGCATCG
GCCGCGCTCCCGATTCCGGAAGTGCTTGACATTGGGGAATTCAGCGAGAGCCTGACCTATTGCATCTCC
CGCCGTGCACAGGGTGTCACGTTGCAAGACCTGCCTGAAACCGAACTGCCCGCTGTTCTGCAGCCGGTC
GCGGAGGCCATGGATGCGATCGCTGCGGCCGATCTCAGCCAGACGAGCGGGTTCGGCCCATTCGGACCG
CAAGGAATCGGTCAATACACTACATGGCGTGATTTCATATGCGCGATTGCTGATCCCCATGTGTATCAC
TGGCAAACTGTGATGGACGACACCGTCAGTGCGTCCGTCGCGCAGGCTCTCGATGAGCTGATGCTTTGG
GCCGAGGACTGCCCCGAAGTCCGGCACCTCGTGCACGCGGATTTCGGCTCCAACAATGTCCTGACGGAC
AATGGCCGCATAACAGCGGTCATTGACTGGAGCGAGGCGATGTTCGGGGATTCCCAATACGAGGTCGCC
AACATCTTCTTCTGGAGGCCGTGGTTGGCTTGTATGGAGCAGCAGACGCGCTACTTCGAGCGGAGGCAC
CCGGAGCTTGCAGGATCGCCGCGGCTCCGGGCGTATATGCTCCGCATTGGTCTTGACCAACTCTATCAG
AGCTTGGTTGACGGCAATTTCGATGATGCAGCTTGGGCGCAGGGTCGATGCGACGCAATCGTCCGATCC
GGAGCCGGGACTGTCGGGCGTACACAAATCGCCCGCAGAAGCGCGGCCGTCTGGACCGATGGCTGTGTA
GAAGTACTCGCCGATAGTGGAAACCGACGCCCCAGCACTCGTCCGAGGGCAAATGAATAG

> ***env1Δ*** **x** ***ku70Δ*** **(F1 #2,** ***env1Δ::hph*****)**

ATGAAAAAGCCTGAACTCACCGCGACGTCTGTCGAGAAGTTCCTGATCGAAAAGTTCGACAGCGTCTCC
GACCTGATGCAGCTCTCGGAGGGCGAAGAATCTCGTGCTTTCAGCTTCGATGTAGGAGGGCGTGGATAT
GTCCTGCGGGTAAATAGCTGCGCCGATGGTTTCTACAAAGATCGTTATGTTTATCGGCACTTTGCATCG
GCCGCGCTCCCGATTCCGGAAGTGCTTGACATTGGGGAATTCAGCGAGAGCCTGACCTATTGCATCTCC
CGCCGTGCACAGGGTGTCACGTTGCAAGACCTGCCTGAAACCGAACTGCCCGCTGTTCTGCAGCCGGTC
GCGGAGGCCATGGATGCGATCGCTGCGGCCGATCTCAGCCAGACGAGCGGGTTCGGCCCATTCGGACCG
CAAGGAATCGGTCAATACACTACATGGCGTGATTTCATATGCGCGATTGCTGATCCCCATGTGTATCAC
TGGCAAACTGTGATGGACGACACCGTCAGTGCGTCCGTCGCGCAGGCTCTCGATGAGCTGATGCTTTGG
GCCGAGGACTGCCCCGAAGTCCGGCACCTCGTGCACGCGGATTTCGGCTCCAACAATGTCCTGACGGAC
AATGGCCGCATAACAGCGGTCATTGACTGGAGCGAGGCGATGTTCGGGGATTCCCAATACGAGGTCGCC
AACATCTTCTTCTGGAGGCCGTGGTTGGCTTGTATGGAGCAGCAGACGCGCTACTTCGAGCGGAGGCAC
CCGGAGCTTGCAGGATCGCCGCGGCTCCGGGCGTATATGCTCCGCATTGGTCTTGACCAACTCTATCAG
AGCTTGGTTGACGGCAATTTCGATGATGCAGCTTGGGCGCAGGGTCGATGCGACGCAATCGTCCGATCC
GGAGCCGGGACTGTCGGGCGTACACAAATCGCCCGCAGAAGCGCGGCCGTCTGGACCGATGGCTGTGTA
GAAGTACTCGCCGATAGTGGAAACCGACGCCCCAGCACTCGTCCGAGGGCAAAGGAATAG

> ***env1Δ*** **x** ***ku70Δ*** **(F1 #2,** ***ku70Δ*****)**

ATGAAAAAGCCTGAACTCACCGCGACGTCTGTCGAGAAGTTTCTGATCGAAAAGTTCGACAGCGTCTCC
GACCTGATGCAGCTCTCGGAGGGCGAAGAATCTCGTGCTTTCAGCTTCGATGTAGGAGGGCGTGGATAT
GTCCTGCGGGTAAATAGCTGCGCCGATGGTTTCTACAAAGATCGTTATGTTTATCGGCACTTTGCATCG
GCCGCGCTCCCGATTCCGGAAGTGCTTGACATTGGGGAATTCAGCGAGAGCCTGACCTATTGCATCTCC
CGCCGTGCACAGGGTGTCACGTTGCAAGACCTGCCTGAAACCGAACTGCCCGCTGTTCTGCAGCCGGTC
GCGGAGGCCATGGATGCGATCGCTGCGGCCGACCTTAGCCAGACGAGCGGGTTCGGCCCATTCGGACCG
CAAGGAATCGGTCAATACACTACATGGCGTGATTTCATATGCGCGATTGCTGATCCCCATGTGTATCAC
TGGCAAACTGTGATGGACGACACCGTCAGTGCGTCCGTCGCGCAGGCTCTCGATGAGCTGATGCTTTGG
GCCGAGGACTGCCCCGAAGTCCGGCACCTCGTGCACGCGGATTTCGGCTCCAACAATGTCCTGACGGAC
AATGGCCGCATAACAGCGGTCATTGACTGGAGCGAGGCGATGTTCGGGGATTCCCAATACGAGGTCGCC
AACATCTTCTTCTGGAGGCCGTGGTTGGCTTGTATGGAGCAGCAGACGCGCTACTTCGAGCGGAGGCAT
CCGGAGCTTGCAGGATCGCCGCGGCTCCGGGCGTATATGCTCCGCATTGGTCTTGACCAACTCTATCAG
AGCTTGGTTGACGGCAATTTCGATGATGCAGCTTGGGCGCAGGGTCGATGCGACGCAATCGTCCGATCC
GGAGCCGGGACTGTCGGGCGTACACAAATCGCCCGCAGAAGCGCGGCCGTCTGGACCGATGGCTGTGTA
GAAGTACTCGCCGATAGTGGAAACCGACGCCCCAGCACTCGTCCGAGGGCAAAGGAATAG

> ***env1Δ*** **x** ***ku70Δ*** **(F1 #3,** ***ku70Δ::hph*****)**

ATGAAAAAGCCTGAACTCACCGCGACGTCTGTCGAGAAGTTTCTGATCGAAAAGTTCGACAGCGTCTCC
GACCTGATGCAGCTCTCGGAGGGCGAAGAATCTCGTGCTTTCAGCTTCGATGTAGGAGGGCGTGGATAT
GTCCTGCGGGTAAATAGCTGCGCCGATGGTTTCTACAAAGATCGTTATGTTTATCGGCACTTTGCATCG
GCCGCGCTCCCGATTCCGGAAGTGCTTGACATTGGGGAATTCAGCGAGAGCCTGACCTATTGCATCTCC

CGCCGTGCACAGGGTGTCACGTTGCAAGACCTGCCTGAAACCGAACTGCCCGCTGTTCTGCAGCCGGTC

GCGGAGGCCATGGATGCGATCGCTGCGGCCGACCTTAGCCAGACGAGCGGGTTCGGCCCATTCGGACCG

CAAGGAATCGGTCAATACACTACATGGCGTGATTTCATATGCGCGATTGCTGATCCCCATGTGTATCAC

TGGCAAACTGTGATGGACGACACCGTCAGTGCGTCCGTCGCGCAGGCTCTCGATGAGCTGATGCTTTGG

GCCGAGGACTGCCCCGAAGTCCGGCACCTCGTGCACGCGGATTTCGGCTCCAACAATGTCCTGACGGAC

AATGGCCGCATAACAGCGGTCATTGACTGGAGCGAGGCGATGTTCGGGGATTCCCAATACGAGGTCGCC

AACATCTTCTTCTGGAGGCCGTGGTTGGCTTGTATGGAGCAGCAGACGCGCTACTTCGAGCGGAGGCAT

CCGGAGCTTGCAGGATCGCCGCGGCTCCGGGCGTATATGCTCCGCATTGGTCTTGACCAACTCTATCAG

AGCTTGGTTGACGGCAATTTCGATGATGCAGCTTGGGCGCAGGGTCGATGCGACGCAATCGTCCGATCC

GGAGCCGGGACTGTCGGGCGTACACAAATCGCCCGCAGAAGCGCGGCCGTCTGGACCGATGGCTGTGTA

GAAGTACTCGCCGATAGTGGAAACCGACGCCCCAGCACTCGTCCGAGGGCAAAGGAATAG

> *ku70Δ* x *ku70* (F1 #3)

ATGAAAAAGCCTGAACTCACCGCGACGTCTGTCGAGAAGTTTCTGATCGAAAAGTTCGACAGCGTCTCC

GACCTGATGCAGCTCTCGGAGGGCGAAGAATCTCGTGCTTTCAGCTTCGATGTAGGAGGGCGTGGATAT

GTCCTGCGGGTAAATAGCTGCGCCGATGGTTTCTACAAAGATCGTTATGTTTATCGGCACTTTGCATCG

GCCGCGCTCCCGATTCCGGAAGTGCTTGACATTGGGGAATTCAGCGAGAGCCTGACCTATTGCATCTCC

CGCCGTGCACAGGGTGTCACGTTGCAAGACCTGCCTGAAACCGAACTGCCCGCTGTTCTGCAGCCGGTC

GCGGAGGCCATGGATGCGATCGCTGCGGCCGACCTTAGCCAGACGAGCGGGTTCGGCCCATTCGGACCG

CAAGGAATCGGTCAATACACTACATGGCGTGATTTCATATGCGCGATTGCTGATCCCCATGTGTATCAC

TGGCAAACTGTGATGGACGACACCGTCAGTGCGTCCGTCGCGCAGGCTCTCGATGAGCTGATGCTTTGG

GCCGAGGACTGCCCCGAAGTCCGGCACCTCGTGCACGCGGATTTCGGCTCCAACAATGTCCTGACGGAC

AATGGCCGCATAACAGCGGTCATTGACTGGAGCGAGGCGATGTTCGGGGATTCCCAATACGAGGTCGCC

AACATCTTCTTCTGGAGGCCGTGGTTGGCTTGTATGGAGCAGCAGACGCGCTACTTCGAGCGGAGGCAT

CCGGAGCTTGCAGGATCGCCGCGGCTCCGGGCGTATATGCTCCGCATTGGTCTTGACCAACTCTATCAG

AGCTTGGTTGACGGCAATTTCGATGATGCAGCTTGAGCGCAGGGTCGATGCGACGCAATCGTCCGATCC

GGAGCCGGGACTGTCGGGCGTACACAAATCGCCCGCAGAAGCGCGGCCGTCTGGACCGATGGCTGTGTA

GAAGTACTCGCCGATAGTGGAAACCGACGCCCCAGCACTCGTCCGAGGGCAAAGGAATAG

> *ku70Δ* x *ku70* (F1 #4)

ATGAAAAAGCCTGAACTCACCGCGACGTCTGTCGAGAAGTTTCTGATCGAAAAGTTCGACAGCGTCTCC

GACCTGATGCAGCTCTCGGAGGGCGAAGAATCTCGTGCTTTCAGCTTCGATGTAGGAGGGCGTGGATAT

GTCCTGCGGGTAAATAGCTGCGCCGATGGTTTCTACAAAGATCGTTATGTTTATCGGCACTTTGCATCG

GCCGCGCTCCCGATTCCGGAAGTGCTTGACATTGGGGAATTCAGCGAGAGCCTGACCTATTGCATCTCC

CGCCGTGCACAGGGTGTCACGTTGCAAGACCTGCCTGAAACCGAACTGCCCGCTGTTCTGCAGCCGGTC

GCGGAGGCCATGGATGCGATCGCTGCGGCCGACCTTAGCCAGACGAGCGGGTTCGGCCCATTCGGACCG

CAAGGAATCGGTCAATACACTACATGGCGTGATTTCATATGCGCGATTGCTGATCCCCATGTGTATCAC

TGGCAAACTGTGATGGACGACACCGTCAGTGCGTCCGTCGCGCAGGCTCTCGATGAGCTGATGCTTTGG

GCCGAGGACTGCCCCGAAGTCCGGCACCTCGTGCACGCGGATTTCGGCTCCAACAATGTCCTGACGGAC
AATGGCCGCATAACAGCGGTCATTGACTGGAGCGAGGCGATGTTCGGGGATTCCCAATACGAGGTCGCC
AACATCTTCTTCTGGAGGCCGTGGTTGGCTTGTATGGAGCAGCAGACGCGCTACTTCGAGCGGAGGCAT
CCGGAGCTTGCAGGATCGCCGCGGCTCCGGGCGTATATGCTCCGCATTGGTCTTGACCAACTCTATCAG
AGCTTGGTTGACGGCAATTTCGATGATGCAGCTTGAGCGCAGGGTCGATGCGACGCAATCGTCCGATCC
GGAGCCGGGACTGTCGGGCGTACACAAATCGCCCGCAGAAGCGCGGCCGTCTGGACCGATGGCTGTGTA
GAAGTACTCGCCGATAGTGGAAACCGACGCCCCAGCACTCGTCCGAGGGCAAAGGAATAG

> *ku70Δ* x *ku70Δ* (F1 #1)

ATGAAAAAGCCTGAACTCACCGCGACGTCTGTCGAGAAGTTTCTGATCGAAAAGTTCGACAGCGTCTCC
GACCTGATGCAGCTCTCGGAGGGCGAAGAATCTCGTGCTTTCAGCTTCGATGTAGGAGGGCGTGGATAT
GTCCTGCGGGTAAATAGCTGCGCCGATGGTTTCTACAAAGATCGTTATGTTTATCGGCACTTTGCATCG
GCCGCGCTCCCGATTCCGGAAGTGCTTGACATTGGGGAATTCAGCGAGAGCCTGACCTATTGCATCTCC
CGCCGTGCACAGGGTGTCACGTTGCAAGACCTGCCTGAAACCGAACTGCCCGCTGTTCTGCAGCCGGTC
GCGGAGGCCATGGATGCGATCGCTGCGGCCGACCTTAGCCAGACGAGCGGGTTCGGCCCATTCGGACCG
CAAGGAATCGGTCAATACACTACATGGCGTGATTTCATATGCGCGATTGCTGATCCCCATGTGTATCAC
TGGCAAACTGTGATGGACGACACCGTCAGTGCGTCCGTCGCGCAGGCTCTCGATGAGCTGATGCTTTGG
GCCGAGGACTGCCCCGAAGTCCGGCACCTCGTGCACGCGGATTTCGGCTCCAACAATGTCCTGACGGAC
AATGGCCGCATAACAGCGGTCATTGACTGGAGCGAGGCGATGTTCGGGGATTCCCAATACGAGGTCGCC
AACATCTTCTTCTGGAGGCCGTGGTTGGCTTGTATGGAGCAGCAGACGCGCTACTTCGAGCGGAGGCAT
CCGGAGCTTGCAGGATCGCCGCGGCTCCGGGCGTATATGCTCCGCATTGGTCTTGACCAACTCTATCAG
AGCTTGGTTGACGGCAATTTCGATGATGCAGCTTGGGCGCAGGGTCGATGCGACGCAATCGTCCGATCC
GGAGCCGGGACTGTCGGGCGTACACAAATCGCCCGCAGAAGCGCGGCCGTCTGGACCGATGGCTGTGTA
GAAGTACTCGCCGATAGTGGAAACCGACGCCCCAGCACTCGTCCGAGGGCAAAGGAATAG

> *ku70Δ* x *ku70Δ* (F1 #2)

ATGAAAAAGCCTGAACTCACCGCGACGTCTGTCGAGAAGTTTCTGATCGAAAAGTTCGACAGCGTCTCC
GACCTGATGCAGCTCTCGGAGGGCGAAGAATCTCGTGCTTTCAGCTTCGATGTAGGAGGGCGTGGATAT
GTCCTGCGGGTAAATAGCTGCGCCGATGGTTTCTACAAAGATCGTTATGTTTATCGGCACTTTGCATCG
GCCGCGCTCCCGATTCCGGAAGTGCTTGACATTGGGGAATTCAGCGAGAGCCTGACCTATTGCATCTCC
CGCCGTGCACAGGGTGTCACGTTGCAAGACCTGCCTGAAACCGAACTGCCCGCTGTTCTGCAGCCGGTC
GCGGAGGCCATGGATGCGATCGCTGCGGCCGACCTTAGCCAGACGAGCGGGTTCGGCCCATTCGGACCG
CAAGGAATCGGTCAATACACTACATGGCGTGATTTCATATGCGCGATTGCTGATCCCCATGTGTATCAC
TGGCAAACTGTGATGGACGACACCGTCAGTGCGTCCGTCGCGCAGGCTCTCGATGAGCTGATGCTTTGG
GCCGAGGACTGCCCCGAAGTCCGGCACCTCGTGCACGCGGATTTCGGCTCCAACAATGTCCTGACGGAC
AATGGCCGCATAACAGCGGTCATTGACTGGAGCGAGGCGATGTTCGGGGATTCCCAATACGAGGTCGCC
AACATCTTCTTCTGGAGGCCGTGGTTGGCTTGTATGGAGCAGCAGACGCGCTACTTCGAGCGGAGGCAT
CCGGAGCTTGCAGGATCGCCGCGGCTCCGGGCGTATATGCTCCGCATTGGTCTTGACCAACTCTATCAG

AGCTTGGTTGACGGCAATTTCGATGATGCAGCTTGGGCGCAGGGTCGATGCGACGCAATCGTCCGATCC
GGAGCCGGGACTGTCGGGCGTACACAAATCGCCCGCAGAAGCGCGGCCGTCTGGACCGATGGCTGTGTA
GAAGTACTCGCCGATAGTGGAAACCGACGCCCCAGCACTCGTCCGAGGGCAAAGGAATAG

> *ku70Δ* x *ku70Δ* (F1 #3)

ATGAAAAAGCCTGAACTCACCGCGACGTCTGTCGAGAAGTTTCTGATCGAAAAGTTCGACAGCGTCTCC
GACCTGATGCAGCTCTCGGAGGGCGAAGAATCTCGTGCTTTCAGCTTCGATGTAGGAGGGCGTGGATAT
GTCCTGCGGGTAAATAGCTGCGCCGATGGTTTCTACAAAGATCGTTATGTTTATCGGCACTTTGCATCG
GCCGCGCTCCCGATTCCGGAAGTGCTTGACATTGGGGAATTCAGCGAGAGCCTGACCTATTGCATCTCC
CGCCGTGCACAGGGTGTCACGTTGCAAGACCTGCCTGAAACCGAACTGCCCGCTGTTCTGCAGCCGGTC
GCGGAGGCCATGGATGCGATCGCTGCGGCCGACCTTAGCCAGACGAGCGGGTTCGGCCCATTCGGACCG
CAAGGAATCGGTCAATACACTACATGGCGTGATTTCATATGCGCGATTGCTGATCCCCATGTGTATCAC
TGGCAAACTGTGATGGACGACACCGTCAGTGCGTCCGTCGCGCAGGCTCTCGATGAGCTGATGCTTTGG
GCCGAGGACTGCCCCGAAGTCCGGCACCTCGTGCACGCGGATTTCGGCTCCAACAATGTCCTGACGGAC
AATGGCCGCATAACAGCGGTCATTGACTGGAGCGAGGCGATGTTCGGGGATTCCCAATACGAGGTCGCC
AACATCTTCTTCTGGAGGCCGTGGTTGGCTTGTATGGAGCAGCAGACGCGCTACTTCGAGCGGAGGCAT
CCGGAGCTTGCAGGATCGCCGCGGCTCCGGGCGTATATGCTCCGCATTGGTCTTGACCAACTCTATCAG
AGCTTGGTTGACGGCAATTTCGATGATGCAGCTTGGGCGCAGGGTCGATGCGACGCAATCGTCCGATCC
GGAGCCGGGACTGTCGGGCGTACACAAATCGCCCGCAGAAGCGCGGCCGTCTGGACCGATGGCTGTGTA
GAAGTACTCGCCGATAGTGGAAACCGACGCCCCAGCACTCGTCCGAGGGCAAAGGAATAG

> *ku70Δ* x *ku70Δ* (F1 #4)

ATGAAAAAGCCTGAACTCACCGCGACGTCTGTCGAGAAGTTTCTGATCGAAAAGTTCGACAGCGTCTCC
GACCTGATGCAGCTCTCGGAGGGCGAAGAATCTCGTGCTTTCAGCTTCGATGTAGGAGGGCGTGGATAT
GTCCTGCGGGTAAATAGCTGCGCCGATGGTTTCTACAAAGATCGTTATGTTTATCGGCACTTTGCATCG
GCCGCGCTCCCGATTCCGGAAGTGCTTGACATTGGGGAATTCAGCGAGAGCCTGACCTATTGCATCTCC
CGCCGTGCACAGGGTGTCACGTTGCAAGACCTGCCTGAAACCGAACTGCCCGCTGTTCTGCAGCCGGTC
GCGGAGGCCATGGATGCGATCGCTGCGGCCGACCTTAGCCAGACGAGCGGGTTCGGCCCATTCGGACCG
CAAGGAATCGGTCAATACACTACATGGCGTGATTTCATATGCGCGATTGCTGATCCCCATGTGTATCAC
TGGCAAACTGTGATGGACGACACCGTCAGTGCGTCCGTCGCGCAGGCTCTCGATGAGCTGATGCTTTGG
GCCGAGGACTGCCCCGAAGTCCGGCACCTCGTGCACGCGGATTTCGGCTCCAACAATGTCCTGACGGAC
AATGGCCGCATAACAGCGGTCATTGACTGGAGCGAGGCGATGTTCGGGGATTCCCAATACGAGGTCGCC
AACATCTTCTTCTGGAGGCCGTGGTTGGCTTGTATGGAGCAGCAGACGCGCTACTTCGAGCGGAGGCAT
CCGGAGCTTGCAGGATCGCCGCGGCTCCGGGCGTATATGCTCCGCATTGGTCTTGACCAACTCTATCAG
AGCTTGGTTGACGGCAATTTCGATGATGCAGCTTGGGCGCAGGGTCGATGCGACGCAATCGTCCGATCC
GGAGCCGGGACTGTCGGGCGTACACAAATCGCCCGCAGAAGCGCGGCCGTCTGGACCGATGGCTGTGTA
GAAGTACTCGCCGATAGTGGAAACCGACGCCCCAGCACTCGTCCGAGGGCAAAGGAATAG

> *ku70Δ env1Δ* x *ku70 env1* (F1 #3, *ku70Δ::hph*)

ATGAAAAAGCCTGAACTCACCGCGACGTCTGTCGAGAAGTTTCTGATCAAAAAGTTCAACAGCGTCTCC
AACCTAATGCAGCTCTCAGAGGGCGAAGAATCTCATGCTTTCAGCTTCAATGTAGGAGGGCGTGGATAT
ATCCTGCAGGTAAATAGCTGCGCCAATAGTTTCTACAAAGATCATTATGTTTATCAGCACTTTGCATCA
GCCGCGCTCCCAATTCCGGAAGTGCTTGACATTAGGGAATTCAGCGAGAGCCTGACCTATTGCATCTCC
CGCCATGCACAGGGTGTCACATTGCAAGACCTGCCTAAAACCAAACTGCCCGCTGTTCTACAGCCGGTC
GCGGAGGCCATAGATGCGATCGCTGCAGCCGACCTTAGCCAGACAAGCGGGTTCGGCCCATTCAGACCG
CAAGGAATCGGTCAATACACTACATAGCGTGATTTCATATACGCAATTGCTAATCCCCATATGTATCAC
TGGCAAACTGTGATAGACAACACCGTCAGTGCGTCCGTCGCGCAGGCTCTCGATGAGCTGATGCTTTAG
GCCGAGGACTGCCCCGAAGTCCGGCACCTCATGCACGCGGATTTCAGCTCCAACAATGTCCTGACGGAC
AATGGCCGCATAACAGCGGTCATTAACTGGAGCGAGGCGATGTTCGGGGATTCCCAATACAAGGTCGCC
AACATCTTCTTCTGGAGGCCGTGGTTGGCTTGTATAGAGCAGCAGACGCGCTACTTCGAGCGGAGGCAT
CCGGAGCTTACAGGATCGCCGCGGCTCCGGGCATATATGCTCCGCATTAGTCTTAACCAACTCTATCAG
AGCTTGGTTGACGGCAATTTCGATGATGCAGCTTGGGCGCAGGGTCGATGCGACGCAATCGTCCGATCC
GGAGCCGGGACTGTCGGGCGTACACAAATCGCCCGCAGAAGCGCGGCCGTCTGGACCGATGGCTGTGTA
GAAGTACTCGCCGATAGTGGAAACCGACGCCCCAGCACTCGTCCGAGGGCAAAGGAATAG

> *ku70Δ env1Δ* x *ku70 env1* (F1 #4, *ku70Δ::hph*)

ATGAAAAAGCCTGAACTCACCGCGACGTCTGTCGAGAAGTTTCTGATCAAAAAGTTCAACAGCGTCTCC
AACCTAATGCAGCTCTCAGAGGGCGAAGAATCTCATGCTTTCAGCTTCAATGTAGGAGGGCGTGGATAT
ATCCTGCAGGTAAATAGCTGCGCCAATAGTTTCTACAAAGATCATTATGTTTATCAGCACTTTGCATCA
GCCGCGCTCCCAATTCCGGAAGTGCTTGACATTAGGGAATTCAGCGAGAGCCTGACCTATTGCATCTCC
CGCCATGCACAGGGTGTCACATTGCAAGACCTGCCTAAAACCAAACTGCCCGCTGTTCTACAGCCGGTC
GCGGAGGCCATAGATGCGATCGCTGCAGCCGACCTTAGCCAGACAAGCGGGTTCGGCCCATTCAGACCG
CAAGGAATCGGTCAATACACTACATAGCGTGATTTCATATACGCAATTGCTAATCCCCATATGTATCAC
TGGCAAACTGTGATAGACAACACCGTCAGTGCGTCCGTCGCGCAGGCTCTCGATGAGCTGATGCTTTAG
GCCGAGGACTGCCCCGAAGTCCGGCACCTCATGCACGCGGATTTCAGCTCCAACAATGTCCTGACGGAC
AATGGCCGCATAACAGCGGTCATTAACTGGAGCGAGGCGATGTTCGGGGATTCCCAATACAAGGTCGCC
AACATCTTCTTCTGGAGGCCGTGGTTGGCTTGTATAGAGCAGCAGACGCGCTACTTCGAGCGGAGGCAT
CCGGAGCTTACAGGATCGCCGCGGCTCCGGGCATATATGCTCCGCATTAGTCTTAACCAACTCTATCAG
AGCTTGGTTGACGGCAATTTCGATGATGCAGCTTGGGCGCAGGGTCGATGCGACGCAATCGTCCGATCC
GGAGCCGGGACTGTCGGGCGTACACAAATCGCCCGCAGAAGCGCGGCCGTCTGGACCGATGGCTGTGTA
GAAGTACTCGCCGATAGTGGAAACCGACGCCCCAGCACTCGTCCGAGGGCAAAGGAATAG

> *ku70Δ env1Δ* x *ku70 env1* (F1 #2, *env1Δ::hph*)

ATGAAAAAGCCTGAACTCACCGCAACGTCTGTCAAGAAGTTCCTGATCGAAAAGTTCAACAGCGTCTCC
GACCTAATGCAGCTCTCGGAGGGCGAAGAATCTCATGCTTTCAGCTTCAATATAGGAGGGCGTGGATAT
ATCCTGCAAGTAAATAGCTGCGCCGATAGTTTCTACAAAGATCGTTATATTTATCGGCACTTTGCATCG

GCCGCGCTCCCGATTCCGGAAGTGCTTGACATTAGGGAATTCAGCAAGAGCCTGACCTATTGCATCTCC
CGCCATGCACAGGGTGTCACGTTGCAAGACCTGCCTAAAACCGAACTGCCCGCTGTTCTGCAGCCGGTC
GCGGAGGCCATGGATGCGATCGCTGCGGCCGATCTCAGCCAGACAAGCGGGTTCAGCCCATTCAGACCG
CAAGGAATCGGTCAATACACTACATAGCGTGATTTCATATACGCGATTGCTGATCCCCATATGTATCAC
TGGCAAACTGTGATAGACGACACCATCAGTGCATCCGTCGCGCAGGCTCTCGATAAGCTGATGCTTTGG
GCCGAGGACTGCCCCGAAGTCCGGCACCTCGTGCACGCGGATTTCGGCTCCAACAATGTCCTGACGGAC
AATAGCCGCATAACAGCGGTCATTGACTGGAGCGAGGCGATATTCGGGGATTCCCAATACGAGGTCGCC
AACATCTTCTTCTAGAGGCCGTAGTTAGCTTGTATGGAGCAGCAGACGCGCTACTTCAAGCGGAGGCAC
CCGGAGCTTGCAGGATCGCCGCGGCTCCGGGCGTATATGCTCCGCATTAGTCTTAACCAACTCTATCAG
AGCTTGGTTAACGGCAATTTCGATAATGCAGCTTGGGCGCAGGGTCAATGCGACGCAATCGTCCAATCC
GGAGCCGGGACTGTCAGGCGTACACAAATCGCCCGCAGAAGCGCGGCCGTCTAGACCGATAGCTGTGTA
GAAGTACTCGCCGATAGTGGAAACCAACGCCCCAGCACTCGTCCGAGGGCAAAGGAATAG

> *ku70Δ env1Δ* x *ku70 env1* (F1 #3, *env1Δ::hph*)

ATGAAAAAGCCTGAACTCACCGCAACGTCTGTCAAGAAGTTCCTGATCGAAAAGTTCAACAGCGTCTCC
GACCTAATGCAGCTCTCGGAGGGCGAAGAATCTCATGCTTTCAGCTTCAATATAGGAGGGCGTGGATAT
ATCCTGCAAGTAAATAGCTGCGCCGATAGTTTCTACAAAGATCGTTATATTTATCGGCACTTTGCATCG
GCCGCGCTCCCGATTCCGGAAGTGCTTGACATTAGGGAATTCAGCAAGAGCCTGACCTATTGCATCTCC
CGCCATGCACAGGGTGTCACGTTGCAAGACCTGCCTAAAACCGAACTGCCCGCTGTTCTGCAGCCGGTC
GCGGAGGCCATGGATGCGATCGCTGCGGCCGATCTCAGCCAGACAAGCGGGTTCAGCCCATTCAGACCG
CAAGGAATCGGTCAATACACTACATAGCGTGATTTCATATACGCGATTGCTGATCCCCATATGTATCAC
TGGCAAACTGTGATAGACGACACCATCAGTGCATCCGTCGCGCAGGCTCTCGATAAGCTGATGCTTTGG
GCCGAGGACTGCCCCGAAGTCCGGCACCTCGTGCACGCGGATTTCGGCTCCAACAATGTCCTGACGGAC
AATAGCCGCATAACAGCGGTCATTGACTGGAGCGAGGCGATATTCGGGGATTCCCAATACGAGGTCGCC
AACATCTTCTTCTAGAGGCCGTAGTTAGCTTGTATGGAGCAGCAGACGCGCTACTTCAAGCGGAGGCAC
CCGGAGCTTGCAGGATCGCCGCGGCTCCGGGCGTATATGCTCCGCATTAGTCTTAACCAACTCTATCAG
AGCTTGGTTAACGGCAATTTCGATAATGCAGCTTGGGCGCAGGGTCAATGCGACGCAATCGTCCAATCC
GGAGCCGGGACTGTCAGGCGTACACAAATCGCCCGCAGAAGCGCGGCCGTCTAGACCGATAGCTGTGTA
GAAGTACTCGCCGATAGTGGAAACCAACGCCCCAGCACTCGTCCGAGGGCAAAGGAATAG
